# Asymmetric isochalcogenourea-catalysed (4 + 2)-cycloadditions of *ortho*-quinone methides and allenoates^†^

**DOI:** 10.1039/d4ob01855a

**Published:** 2025-01-22

**Authors:** Anna Scheucher, Christoph Gross, Magdalena Piringer, Johanna Novacek, Armin R. Ofial, Mario Waser

**Affiliations:** a Institute of Organic Chemistry, Johannes Kepler University Linz, Altenbergerstrasse 69, 4040 Linz, Austria.; b Department Chemie, Ludwig-Maximilians-Universität München, Butenandtstr. 5-13, 81377 München, Germany; c Institute of Analytical and General Chemistry, Johannes Kepler University Linz, Altenbergerstrasse 69, 4040 Linz, Austria

## Abstract

Chiral isochalcogenoureas (*i*.*e*. isothioureas and isoselenoureas) catalyse the asymmetric (4 + 2)-cycloaddition of various allenoates with *ortho*-quinone methides. This approach provides straightforward access to different chromane derivatives with high enantioselectivities, good yields, and control of the configuration of the exocyclic double bond. Furthermore, some of the novel *ortho*-quinone methides used herein were successfully integrated into the Mayr reactivity scale by determining their electrophilicity parameter.

## Introduction

**Scheme 1:**
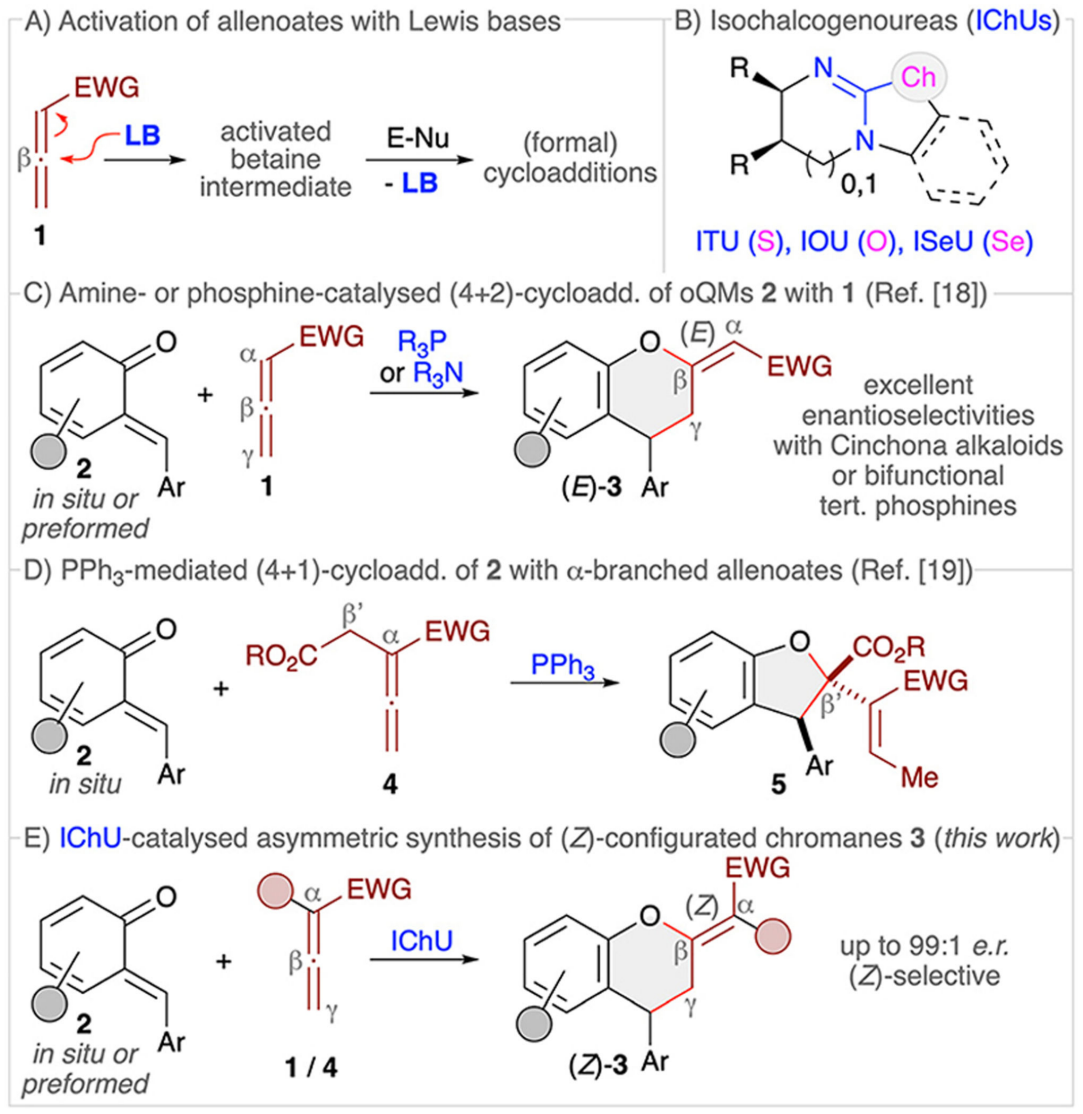
(A) General concept of LB-activation of allenoates; (B) isochalcogenoureas; (C) established amine- and phosphine-catalysed (4 + 2)-cycloadditions of *o*-quinone methides **2** with allenoates **1**; (D) our recently developed PPh_3_-mediated (4 + 1)-cycloaddition of α-branched allenoates **4;** (E) the herein investigated IChU-catalysed synthesis of chromanes **3**.

Allenoates 1 have been established as versatile reagents for various (formal) cycloaddition reactions over the course of the last three decades.^1,2^ Upon using chiral Lewis base (LB) organocatalysts,^3^ which form an activated betaine intermediate *via* addition to the β-carbon of the allenoate (Scheme 1A), reactions between allenoates and dipolar (vinylogous) acceptors (E-Nu) can lead to structurally diverse carbo- and heterocycles in a stereoselective manner.^4^ Remarkably, the nature of the catalyst usually has a strong influence on the reaction pathway, thus allowing for orthogonal outcomes depending on the used class of catalysts.^5–10^ Most commonly, (chiral) tertiary phosphines are the Lewis bases of choice for allenoate activations.^5,6^ Besides, also tertiary amines^7^ or N-heterocyclic carbenes (NHCs)^8^ have proven their potential for allenoate-based cycloadditions. Very recently, we have shown that chiral isochalcogenoureas (IChUs, Scheme 1B), *i*.*e*. isothioureas (ITUs)^11^ and isoselenoureas (ISeU),^12^ hold much potential for the activation of allenoates, too.^10^ Interestingly, in our studies we found that these easily accessible bench-stable Lewis bases allow for complementary reaction pathways as compared to the established phosphine- and amine-catalysed protocols.^9,10^ More specifically, so far we have investigated cycloaddition reactions between allenoates and four different classes of Michael acceptors and in all cases we exclusively observed (4 + 2)-heterocycloadditions leading to the formation of highly functionalised dihydropyrans with a (*Z*)-configured exocyclic double bond.^10^ In sharp contrast, phosphine catalysis often leads to (3 + 2)-carbocyclisations^2,13^ while tertiary amines can give analogous (4 + 2)-heterocycloadditions but with (*E*)-configured double bonds instead.^14^ Thus, IChUs represent a powerful alternative catalyst platform for asymmetric allenoate cycloadditions which provides an entry to chiral targets that are not easily accessible with the classically used Lewis base organocatalysts.

*ortho*-Quinone methides (*o*QMs, 2) are intensively investigated building blocks that can undergo various heterocycloaddition reactions.^15^ These reactive and often rather unstable compounds can either be formed *in situ* and then immediately be trapped by nucleophiles or dienophiles. Or, less frequently, *o*QMs have been preformed and used directly. (4 + 2)-Cycloadditions of *o*QMs^16^ can lead to highly functionalised chiral chromane^17^ derivatives 3, and it was recently shown that reactions between allenoates and *o*QMs in the presence of either chiral tertiary amines (*i*.*e*. Cinchona alkaloids) or chiral bifunctional tertiary phosphines can lead to the formation of chromanes 3 with an (*E*)-configured exocyclic double bond (Scheme 1C).^18^ Interestingly, we recently found that the reaction of α-alkyloxycarbonylmethyl-substituted allenoates 4 with *o*QMs 2 in the presence of triphenylphosphine resulted in the formation of dihydrobenzofurans 5 *via* a (4 + 1)-cycloaddition instead (Scheme 1D),^19^ thus underscoring the diversity of possible reaction pathways that allenoates can enter. Considering these recent results from other groups and ourselves, which demonstrate that cycloadditions of allenoates and *o*QMs can lead to various highly decorated aryl-fused oxygenated heterocycles straightforwardly, we were now wondering whether it is possible to carry out such reactions under IChU catalysis as well. Based on our recent observations,^10^ we concluded that this approach should give us predominately access to the (*Z*)-configured chromanes 3 instead of the already established (*E*)-configured ones.^18^ In addition, we were wondering if the presence of α- or γ-substituents will be tolerated without affecting the overall cycloaddition pathway too, in contrast to the mentioned differences when using phosphine catalysis (compare Scheme 1C and D).^18c,19^ Overall such an approach should thus provide an entry to the densely functionalised chiral products 3 in a highly selective manner (Scheme 1E).

## Results and discussion

### Electrophilic reactivities of *o*QMs

According to the general mechanism depicted in Scheme 1A, Lewis base addition to the electrophilic allenoates 1^6^ as well as the subsequent trapping of the nucleophilic betaine intermediate by sufficiently reactive electrophiles are the key bimolecular reactions that need to be understood to rationally optimise the cycloaddition reactions. In the context of this work, insight into the electrophilic reactivity of *ortho*-quinone methides 2 is crucial to define the scope of the IChU-catalysed synthesis of chromanes.

**Scheme 2:**
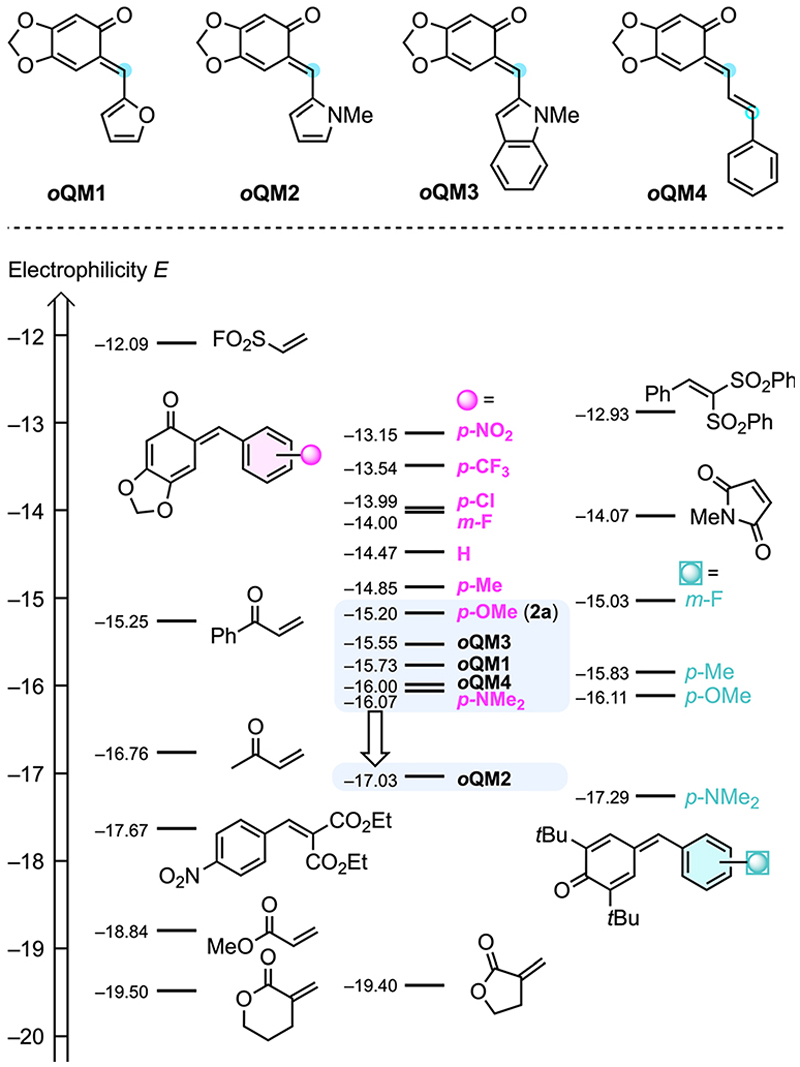
Comparing the Mayr electrophilicities *E* of ***o*QM1–*o*QM4** with those of aryl-substituted *o*QMs (such as 2a), *para*-quinone methides, and further Michael acceptors (with data from ref. 20 and 21c). Compounds are ordered according to their *E* parameters with increasing reactivity from bottom to the top. Top line: blue dots mark the electrophilic positions of ***o*QM1–*o*QM4**.

The Munich team recently synthesised a series of prototypical *o*QMs 2 formally derived from sesamol (= 3,4-methylendioxyphenol) and various acceptor- and donor-substituted benzaldehydes, studied their reactivities toward reference nucleophiles, and finally characterised the electrophilicities *E* of *o*QMs 2 on the Mayr reactivity scale.^20^ To cover further *o*QMs used in this work, we set out to include heteroaryl-substituted *o*QMs with furanyl **(*o*QM1)**, pyrrolyl **(*o*QM2)**, and indolyl **(*o*QM3)** moieties as well as with extended π-system **(*o*QM4)** (Scheme 2). The second-order rate constants *k*_2_ of addition reactions of carbanions (reference nucleophiles) to these *o*QMs in DMSO at 20 °C were determined by using (stopped-flow) photometric methods. Then, the electrophilicities *E* of ***o*QM1–*o*QM4** were calculated from the experimentally determined *k*_2_ and the reported nucleophilicity parameters (*N* and *s*_N_)^21^ of the reference nucleophiles according to the Mayr–Patz equation (see ESI, Section 1† for details).

The *p*-anisyl-substituted *o*QM 2a (*E* = −15.20)^20^ was used in the optimisation and screening studies of this work. Supplementing the Mayr electrophilicity scale^21c^ by the *ortho*-quinone methides ***o*QM1–*o*QM4** shows that all *o*QMs are located in a narrow reactivity range (Scheme 2). For ***o*QM1** (*E* = −15.73), ***o*QM3** (*E* = −15.55), and ***o*QM4** (*E* = −16.00) it can be anticipated that they may perform comparably well as **2a** or the only slightly less electrophilic *p*-(dimethylamino)-substituted *o*QM (*E* = −16.07).^20^ Allene ketones were successfully shown to undergo phosphine-catalysed (4 + 2) annulations with ***o*QM4**.^18c^ However, due to the low regioselectivity of the ambident ***o*QM4** in reactions with nucleophiles (see ESI, Section 12†), we excluded ***o*QM4** from further studies in this work. Similarly, low regioselectivities for the attack of C-nucleophiles at vinyl *para*-quinone methides were reported previously.^22^ The *N*-methylpyrrol-2-yl-substituted *o*QM2 is by almost two orders of magnitude less electrophilic than the standard *o*QM **2a**. Successful (4 + 2)-heterocycloadditions with ***o*QM2** would, therefore, significantly enhance the reactivity range of *o*QMs that could be used for the IChU-catalysed reactions with allenoates.

### Cycloadditions with preformed stabilised *o*QMs

We started our investigations on the allenoate cycloadditions by using the stabilised electron-rich benzodioxole-based *o*QMs 2. A first screening and optimisation of reaction conditions was carried out using *o*QM **2a** and the unbranched allenoate **1a** (Table 1).

**Fig. 1:**
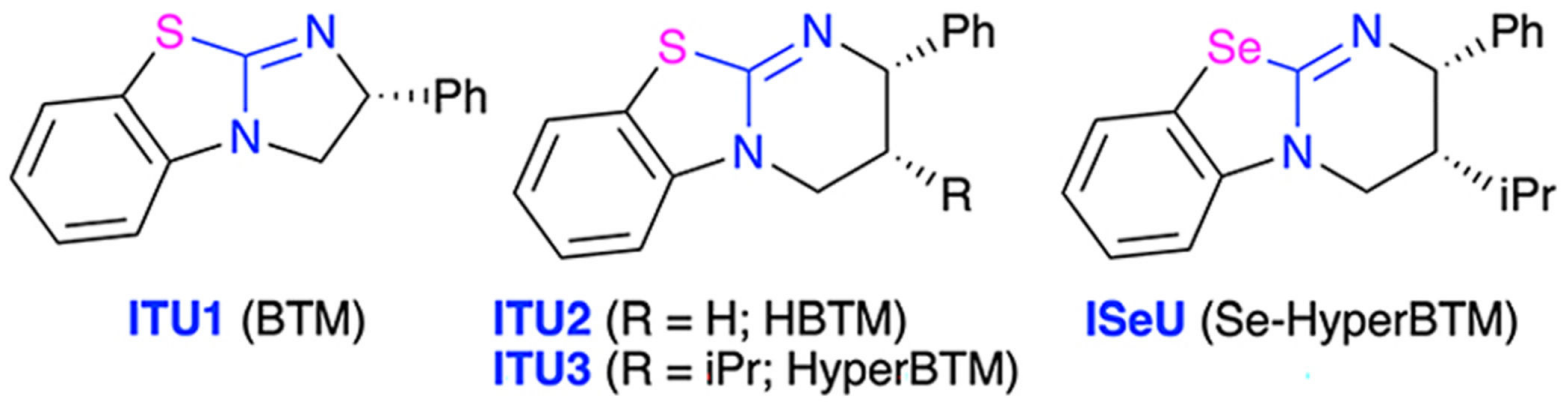
IChUs used herein.

Testing the four different catalysts depicted in Fig. 1 under conditions similar to those established for IChU-catalysed allenoate (4 + 2)-cycloadditions with various Michael acceptors,^10^ we observed a likewise reactivity trend herein as well (entries 1–4). While BTM (ITU1, entry 1) did not allow for any product formation, the 6-ring-based HBTM (ITU2, entry 2), HyperBTM (ITU3, entry 3), and its selenium-containing analogue (ISeU, entry 4) promoted the (4 + 2)-cycloaddition well. This difference in reactivity between the BTM motif and the HBTM/HyperBTM scaffold can most likely be rationalised by the lower nucleophilicity of 5-ring-based isothioureas,^23^ thus slowing down the initial addition to the allenoate (we recently showed that this step has a rather high activation barrier, which most likely also explains the need for higher reaction temperatures^10^). Interestingly, we not only observed the formation of the anticipated chromane **3a** [with the (*Z*)-isomer being the major one; the configuration of the double bond was assigned by NOESY NMR experiments], but also notable amounts of the chromene **6a** (originating from an initial α-attack of the allenoate to the benzylic position of the *o*QM). This observation is in sharp contrast to our previous studies, where analogous α-addition-based products were obtained in minute amounts only (if formed at all). Noteworthy, the enantioselectivity for the targeted (*Z*)-**3a** was very high, independent of the used catalyst (entries 2–4). On the other hand, the catalyst scaffold, as well as the reaction conditions (entries 2–10) had an influence on the product distribution. Overall, it turned out that HyperBTM (**ITU3**) is the Lewis base of choice. Using 20 mol% of this catalyst in toluene at 80 °C allows for around 60% (*Z*)-**3a** selectivity, besides approx. 10% of the (*E*)-diastereomer and slightly less than 30% of **6a** (entry 3). Interestingly, the two side-products (*E*)-**3a** (83 : 17 er) and **6a** (74 : 26 er) were obtained with significantly lower enantio-selectivities as compared to the major product (*Z*)-3a (99 : 1 er). Changing the solvent (entries 8 and 9), adding base (as exemplified for Cs_2_CO_3_; entry 10), and varying the temperature (entries 6 and 7) did not allow for any better results and lower catalyst loading (entry 5) resulted in a reduced yield too. Gratifyingly, the (*Z*)-isomer could easily be separated from the other two cycloaddition side-products by means of a simple silica gel column chromatography, thus giving (*Z*)-**3a** in a moderate isolated yield of 59% with excellent enantioselectivity (entry 3).

**Scheme 3:**
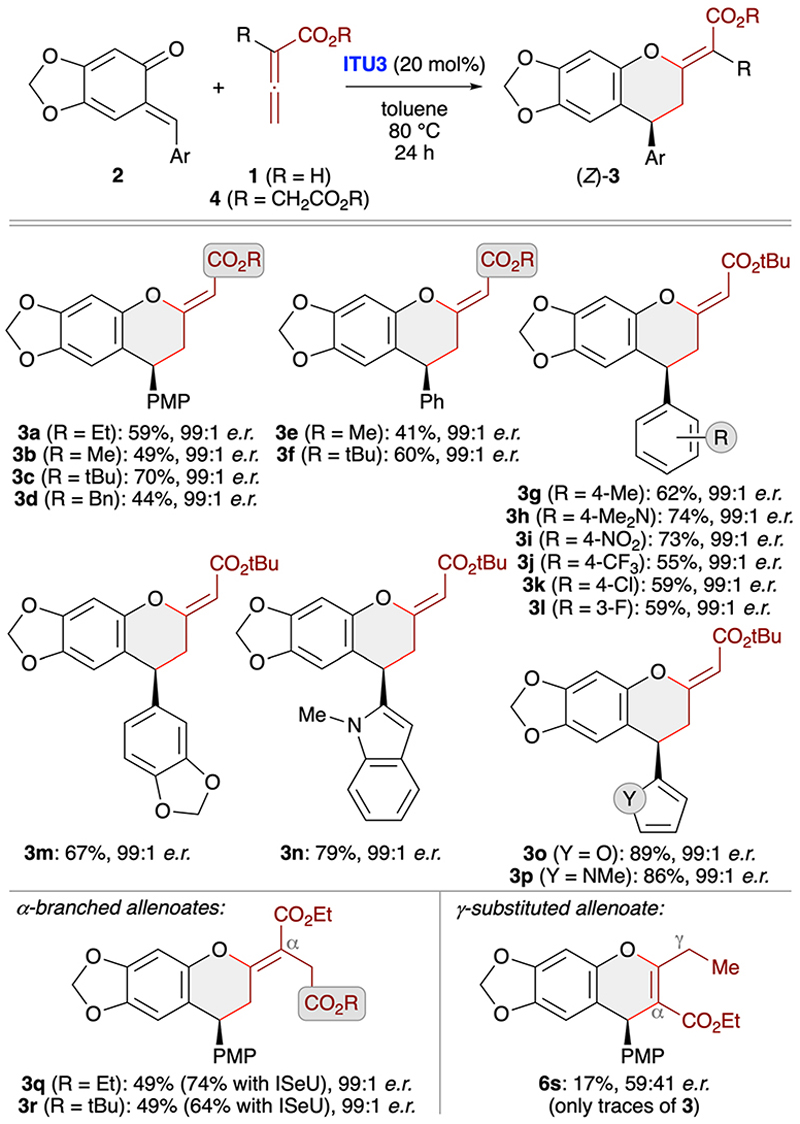
Asymmetric application scope testing various stabilised *o*QMs and different allenoates using (2*S*,3*R*)-HyperBTM (**ITU3**; conditions as specified in entry 3, Table 1).

With suited asymmetric conditions for the synthesis of (*Z*)-**3a** at hand, we next investigated the application scope by using various sesamol-derived *o*QMs 2 as well as different allenoates **1** and **4** (Scheme 3). Varying the ester group of allenoates **1** first (see products 3a–f) showed that *t*-butyl esters allow for the highest yields with reduced amounts of the α-addition products **6**. This can be explained by the higher steric shielding of the α-position, thus preventing formation of compounds **6** while making the γ-position more accessible. Accordingly, testing of different *o*QMs was then carried out with the *t*-butyl allenoate as the cycloaddition partner. As shown for products **3g–3p** various (hetero)aryl groups were well tolerated and in each case the level of enantioselectivity was very high. However, the method came to its limits when using γ-substituted allenoates. In this case the reaction was found to be rather messy and the only distinct product that could be obtained in trace amounts and with very low er was the chromene **6s** (formed *via* α-attack of the activated allenoate to QM). Remarkably when using α-branched allenoates 4 instead again the formation of the chromane skeleton **3** was the dominant transformation (see products **3q** and **3r**). This is in sharp contrast to our previous observations when reacting such allenoates with *in situ* generated *o*QMs (Scheme 1D)^19^ and OH-containing *para*-QMs^24^ in the presence of phosphine catalysts, thus underscoring the generality and functional group tolerance of the IChU-catalysed (4 + 2)-cycloaddition. Interestingly, herein the isoselenourea derivative **ISeU** allowed for higher yields and again the enantioselectivity was nearly perfect.

### Cycloadditions with *in situ* generated *o*QMs

When using less stabilised *o*QMs these transient species are usually generated *in situ* from different easily accessible precursors.^13^ One straightforward approach that should be compatible with our Lewis base-catalysis strategy relies on the use of α-(arylsulfonyl)methyl-substituted β-naphthols 7 under basic conditions, which leads to the formation of the corresponding *o*QMs *via* elimination of an arylsulfinic acid. This strategy was recently also utilised in our (4 + 1)-cycloaddition of allenoates **4** (Scheme 1D)^19^ and we thus set out to explore the possibility of applying this to IChU-catalysed allenoate cycloadditions. By starting from the precursor **7a** and the parent allenoate **1a** we first optimised the synthesis of product **3aa** (Table 2). We initially set the reaction temperature to 40 °C as we know from previous investigations with compounds 7 that higher temperatures usually lead to a relatively fast decomposition of the corresponding *o*QMs. First test reactions with the three isothiourea catalysts **ITU1–3** under basic conditions showed a similar reactivity trend as compared to the stabilised *o*QM **2a** (compare entries 1–3 of both tables). Again HyperBTM (**ITU3**) was best suited giving the targeted (*Z*)-**3aa** with moderate NMR yield and excellent enantioselectivity (entry 3). Interestingly, formation of the (*E*)-diastereoisomer and the α-addition product **6aa** was less pronounced as compared to the use of **2a**. Unfortunately, lower catalyst loading was again not well tolerated (entry 4). Interestingly however, in this case **ISeU** was found to be higher yielding (entry 5). Some further optimisation of reaction conditions was first carried out with **ITU3** (entries 6–8) showing that an excess of 3 equiv. of allenoate is beneficial, while increased or decreased temperature did not allow for any improvement (a few other solvents or bases were tested too but did not allow for any improvement). Finally, using **ISeU** in combination with 3 equiv. of **1a** and 1 equiv. of Cs_2_CO_3_ in toluene at 40 °C allowed for the synthesis of (*Z*)-**3aa** in 77% isolated yield with 99 : 1 er and only minor quantities of the other cycloaddition side products (entry 9).

**Scheme 4:**
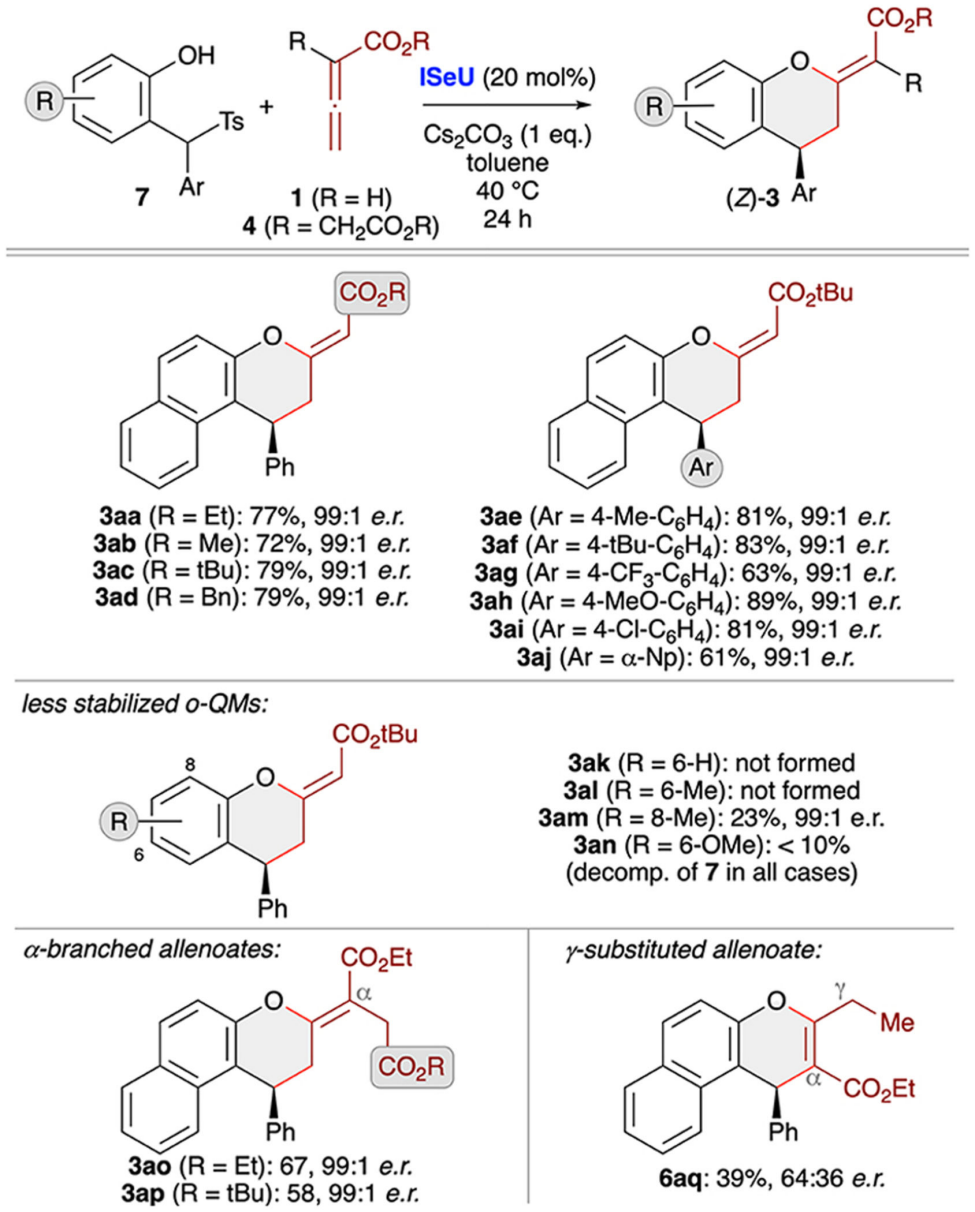
Asymmetric application scope using *in situ* generated *o*QMs (accessed from compounds 7) and different allenoates in the presence of (2*S*,3*R*)-SeHyperBTM (**ISeU**; conditions as detailed in entry 9, Table 2).

Investigating the asymmetric application scope for the cycloaddition starting from *o*QM precursors 7 (Scheme 4) showed that various naphthol-based derivatives are well tolerated, as exemplified for products **3aa–aj**. Unfortunately, however, the method comes to its limits when utilising simple phenol-based QM precursors that yield less stable and thus more easily decomposing *o*QMs. While products **3ak** and **3al** could not be accessed at all and **3an** was only detected in trace amounts, the 8-Me-containing **3am** could at least be obtained in low yield (but with very high enantioselectivity). In all these cases we observed a very pronounced formation of unidentified side-products originating from the decomposition of the *in situ* formed quinone methides. Using α-branched allenoates **4** allowed for the selective (4 + 2)-cycloaddition as well, as demonstrated for the synthesis of chromanes 3ao and **3ap**. Again, this result is in sharp contrast to our recent phosphine-catalysed (4 + 1)-cycloaddition protocol (Scheme 1D),^19^ underscoring the orthogonal catalytic potential of different Lewis bases for allenoate activation. Finally, analogous to the use of preformed *o*QMs (see product 6r, Scheme 3), the use of γ-branched allenoates resulted in the formation of the chromene skeleton herein as well, but with low yield and unsatisfying enantioselectivity only (product 6aq).

We have not been able to obtain crystals of the enantio-enriched products 3 that would have allowed for an unambiguous assignment of their absolute configuration by means of single crystal X-ray diffraction. Thus we recorded vibrational circular dichroism (VCD)^25^ spectra of both enantiomers of compound 3e and compared the experimental spectra with those calculated from DFT optimised structures which strongly supports the absolute configuration depicted in Scheme 3.^26^ This sense of configuration of the major enantiomer is in full accordance with our recent observations of IChU-catalysed allenoate-based (4 + 2)-cycloadditions where we always observed this orientation of the substituent on the stereogenic center in position 4 of tetrahydropyran ring when using the (2*S*,3*R*)-configured **ITU3** or **ISeU**.^10^ Furthermore, comparison of the optical rotation of our (*Z*)-configured products 3 with reported structurally similar (*E*)-configured ones^18^ support this sense of configuration as well and we therefore assigned all other products in analogy.

**Scheme 5:**
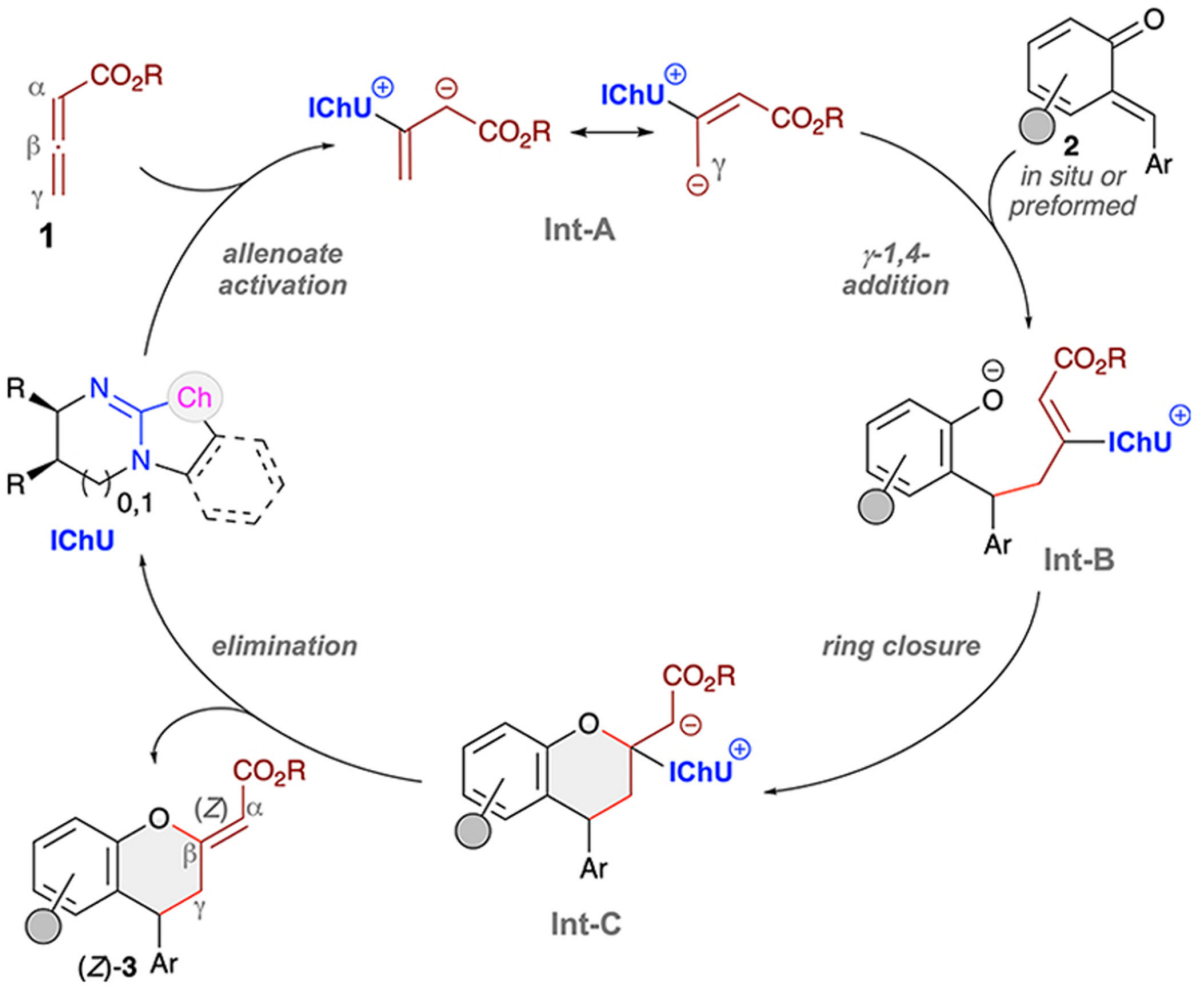
Mechanistic proposal.^10^

Mechanistically, we proposed that the herein reported stereoselective syntheses of chromanes 3 follow the established pathway for our recently introduced IChU-catalysed (4 + 2)-cycloadditions of allenoates with different Michael acceptors (Scheme 5).^10^ More specifically, the IChU first activates the allenoates **1** giving the resonance-stabilised **Int-A**. This chiral intermediate then undergoes 1,4-addition to the *o*QM 2 with its γ-carbon, giving **Int-B**. This is also the step that controls the configuration of products 3. As stated above, so far we have always observed the same sense of induction when using the same enantiomers of our catalysts, thus substantiating a very high level of catalyst control and a very well defined **Int-A. Int-B** then undergoes ring-closure and final IChU-elimination which also sets the configuration of the exocyclic double bond. Interestingly, while other Lewis base catalysts usually favour (*E*)-configurated double bonds in such transformations,^18^ IChUs show pronounced (*Z*)-selectivity, a kinetic phenomenon which we could also recently support by DFT calculations.^10a^

### Follow-up transformations

**Scheme 6:**
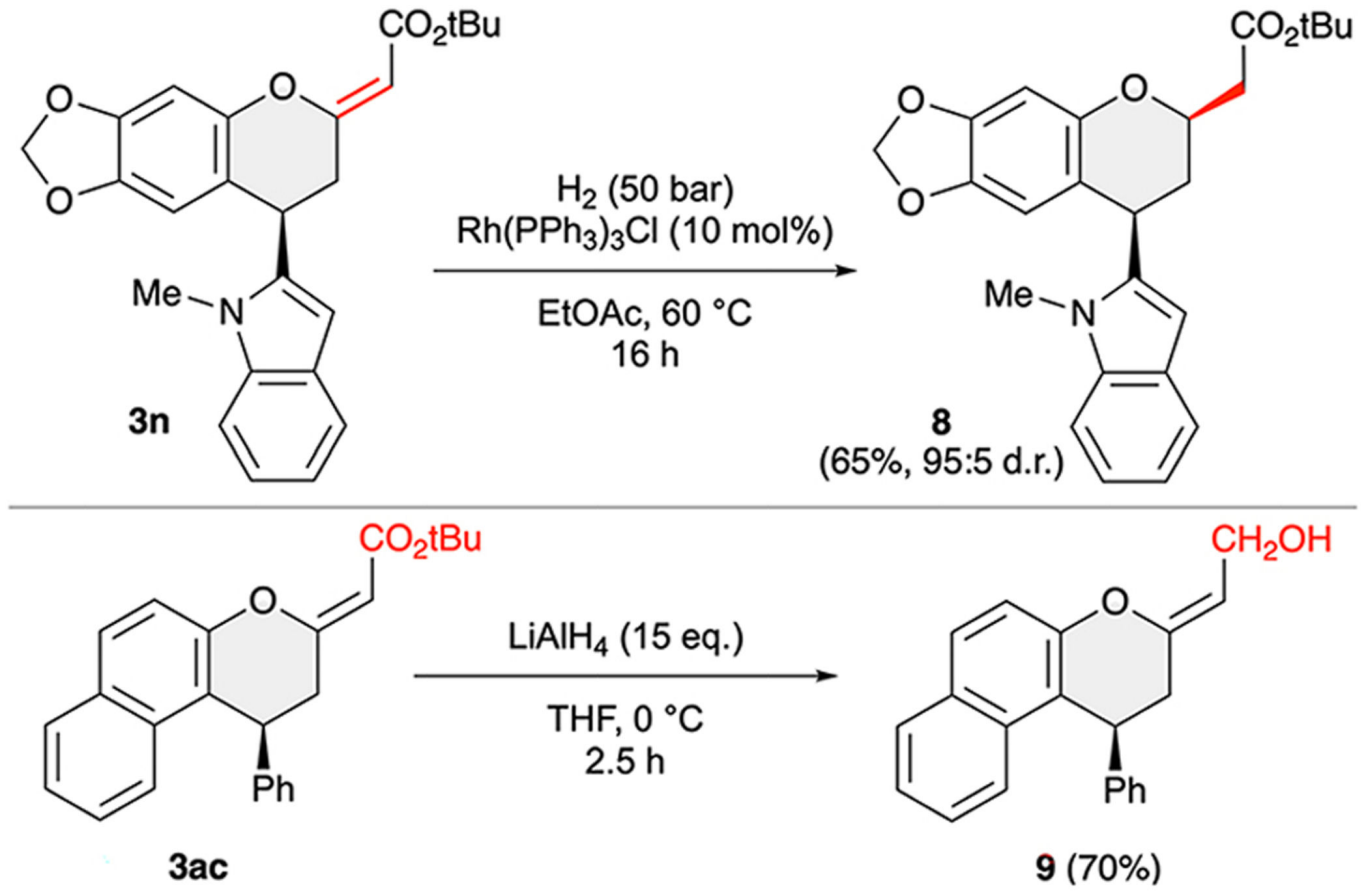
Reductive follow-up transformations of compounds 3.

Finally, we also carried out some test reactions to demonstrate the suitability of products 3 to serve as starting materials for further transformations. As outlined in Scheme 6, the exocyclic double bond can be selectively hydrogenated under homogeneous conditions by using Wilkinson’s catalyst. In this way product 8 was obtained with high diastereoselectivity; the *cis* configuration of the 2-acetyl group and the 4-indolyl substituent was determined by NOESY experiments. Furthermore, the ester functionality can be reduced to the primary alcohol 9 by using an excess of LiAlH_4_. It should be stated that neither of these two transformations have been optimised further, but in our opinion they represent a proof-of-concept to demonstrate the suitability of compounds 3 for further manipulations.

## Conclusions

Isothioureas (ITUs) and isoselenoureas (ISeUs) were successfully employed as chiral Lewis base catalysts for asymmetric (4 + 2)-cycloadditions of various allenoates with different *ortho*-quinone methides. This approach allows for the synthesis of chromanes 3 with (*Z*)-configured exocyclic double bonds in high enantioselectivities and good isolated yields. Accompanying VCD studies supported the assignment of the absolute configuration of the products. Furthermore, the electrophilicity parameters of some heteroaryl-substituted and π-extended *ortho*-quinone methides were successfully determined. These quantitative reactivity data hint to the reactivity range which the electrophilic component in the (4 + 2)-cycloadditions needs to cover. This knowledge should allow us to identify additional suitable reaction partners for such allenoate-based cycloadditions in the future, which can be selected based on their reported electrophilicity parameters *E*.

## Experimental details^26^

### General procedure using preformed *o*QMs 2

A flame dried N_2_-flushed flask was charged with **ITU3** (20 mol%), the respective *o*QM 2 (0.1 mmol, 1 equiv.) and toluene (5 mL, 0.02 mol L^−1^), directly followed by the addition of the allenoate **1** or **4** (0.15 mmol, 1.5 equiv.). The reaction mixture was then stirred at 80 °C for 24 h. After cooling, the mixture was filtered through a Na_2_SO_4_ plug. The solvent was removed under reduced pressure to yield the crude products **3a–q**. Purification *via* preparative TLC (heptane : EtOAc = 2 : 1) gave the products in the reported yields and enantiopurities.

### General procedure using *o*QM precursors 7

A N_2_-flushed and flame-dried flask was charged with ISeU (20 mol%), Cs_2_CO_3_ (0.1 mmol, 1 equiv.), the respective *o*QM precursor 7 (0.1 mmol, 1 equiv.) and toluene (5 mL, 0.02 mol L^−1^). The allenoate (0.3 mmol, 3 equiv.) was added and the mixture was heated to 40 °C and stirred for 24 h. After cooling to r.t., the mixture was filtered through a Na_2_SO_4_ plug and the solvent was removed under reduced pressure to give the crude products 3. Purification *via* preparative TLC (heptane : EtOAc = 2 : 1) gave the products in the reported yields and enantiopurities.

## Supplementary Material

SI

## Figures and Tables

**Table 1:** Optimisation of the (4 + 2)-cycloaddition of allenoate **1a** with *o*QM **2a** ^a^

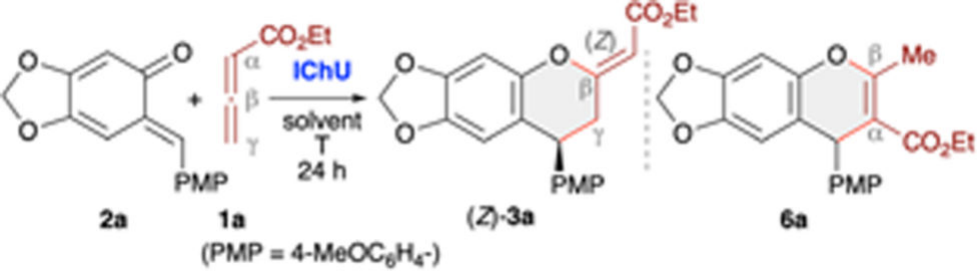							
Entry	IChU (mol%)	Solv.	*T* (°C)	(Z)-3a ^b^ (%)	(Z)-3a er^c^	(E)-3a ^b^ (%)	6a ^b^ (%)
1	**ITU1** (20%)	Tol.	80	—	—	—	—
2	**ITU2** (20%)	Tol.	80	48	99:1	8	32
**3**	**ITU3 (20%)**	**Tol.**	**80**	**61 (59)** ^d^	**99 : 1**	**7 (83 : 17)** ^c^	**28 (74 : 26)** ^[Table-fn TFN3]c^
4	**ISeU** (20%)	Tol.	80	59	99 : 1	13	26
5	**ITU3** (10%)	Tol.	80	54	99 : 1	9	28
6	**ITU3** (20%)	Tol.	40	52	99 : 1	7	20
7	**ITU3** (20%)	Tol.	120	51	99 : 1	8	25
8	**ITU3** (20%)	DCM	80	33	99 : 1	8	29
9	**ITU3** (20%)	THF	80	58	99 : 1	10	29
10	**ITU3** (20%)	Tol.^e^	80	29	99 : 1	4	26

a Unless otherwise stated, reactions were run for 24 h using 0.15 mmol 1a and 0.1 mmol **2a** in the presence of the given catalyst in the indicated solvent (*c* = 0.02 M with respect to **2a**) under N_2_ at the indicated temperature (tol. = toluene; DCM = dichloromethane; THF = tetrahydrofuran).

b Calculated from the ^1^H NMR spectrum of the crude product using mesitylene as an internal standard (IST).

c Enantiomeric ratio determined by HPLC using a chiral stationary phase.

d Isolated yield.

e With added Cs_2_CO_3_ (1 equiv.).

**Table 2:** Optimisation of the (4 + 2)-cycloaddition of allenoate 1a with *o*QM precursors 7a ^a^

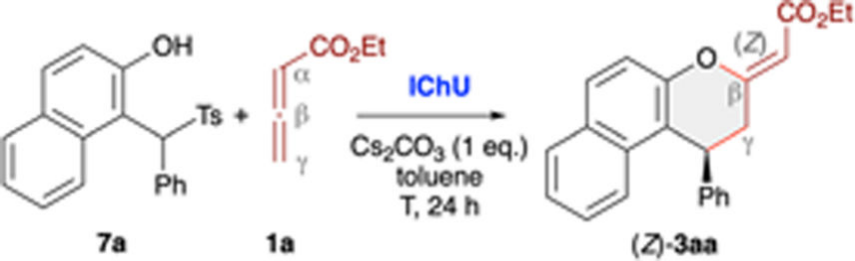						
Entry	IChU (mol%)	*T* (°C)	(*Z*)-3aa ^b^ (%)	(*Z*)-3aa er^c^	(*E*)-3aa ^b^ (%)	6aa^b^ (%)
1	**ITU1** (20%)	40	—	—	—	—
2	**ITU2** (20%)	40	29	97:3	8	5
3	**ITU3** (20%)	40	54	99 : 1	8	4
4	**ITU3** (10%)	40	19	98 : 2	5	3
5	**ISeU** (20%)	40	77	99 : 1	12	4
6	**ITU3** (20%)^d^	40	72	99 : 1	12	4
7	**ITU3** (20%)^d^	60	60	99 : 1	10	5
8	**ITU3** (20%)^d^	25	56	99 : 1	11	4
**9**	**ISeU (20%)** *^d^*	**40**	**79 (77)** *^e^*	**99 : 1**	**12 (63 :37)** ^c^	**3 (56 : 44)** ^c^

a Unless otherwise stated, reactions were run for 24 h using 0.15 mmol **1a** and 0.1 mmol 7a in the presence of the given catalyst in toluene under N_2_ at the indicated temperature.

b Calculated from the ^1^H NMR spectrum of the crude product using mesitylene as an internal standard (IST).

c Enantiomeric ratio determined by HPLC using a chiral stationary phase.

d Using 3 equiv. of allenoate **1a**.

e Isolated yield.

## Data Availability

The data supporting this article have been included as part of the ESI.† Raw data of the individual kinetic measurements that support the findings of this study are openly available in Open Data LMU at https://doi.org/10.5282/ubm/data.545.
