# Supplementary material for: Asymmetric isochalcogenourea-catalysed (4 + 2)-cycloadditions of ortho-quinone methides and allenoates
Source: Org Biomol Chem. Author manuscript; Available in PMC 2025 Jul 5. (PMC11619814; doi:10.1039/d4ob01855a)
Supplement: SI [file EMS206749-supplement-SI.pdf]

Supplementary Information (SI) for Organic & Biomolecular Chemistry.
This journal is © The Royal Society of Chemistry 2024

Electronic Supplementary Information (ESI)

Asymmetric Isochalcogenourea-Catalysed (4+2)-Cycloadditions of *ortho*-Quinone Methides and Allenoates

Anna Scheucher,[a] Christoph Gross,[b] Magdalena Piringer,[a] Johanna Novacek,[c] Armin R. Ofial,[b] and Mario Waser*[a]

[a]Institute of Organic Chemistry, Johannes Kepler University Linz, Altenbergerstraße 69, 4040 Linz, Austria
+43 732 2468 5411; mario.waser@jku.at

[b]Department Chemie, Ludwig-Maximilians-Universität München, Butenandtstraße 5–13, 81377 München, Germany

[c]Institute of Institute of Analytical and General Chemistry, Johannes Kepler University Linz, Altenbergerstraße 69, 4040 Linz, Austria

Contents

1. Determination of Electrophilicity Parameters *E* for *ortho*-Quinone Methides 3

2. General Information 10

3. Syntheses of Novel Starting Materials 13

3.1 Synthesis of *ortho*-quinone methides (*o*QM) (GP1–GP3) 13

3.2 General procedure for the synthesis of *o*QM precursors 7 (GP4) 14

4. (4+2)-Cycloadditions 15

4.1 General remarks 15

4.2 General procedure for the (4+2)-cycloaddition starting from preformed oQMs 2 (GP5) 15

4.3 General procedure for the (4+2)-cycloaddition starting from precursors 7 (GP6) 15

5. Characterization of Novel Starting Materials 16

6. Characterization of Products 3 23

6.1 7,8-Dihydro-6*H*-[1,3]dioxolo[4,5-g]chromanes obtained from oQMs 2 23

6.2 1,2-Dihydro-3*H*-benzo[f]chromanes from QM-precursors 7 33

7. Follow-Up Transformations 41

8. VCD Investigations 43

9. UV/Vis Spectra and Molar Absorption Coefficients of *o*QMs 54

10. Cyclic Voltammetry: Reduction Potentials of *o*QMs 59

11. Kinetics of the Reactions of *o*QMs with Carbanions (Reference Nucleophiles) 61

12. Products of Reactions of *o*QMs with C-Centered Nucleophiles (GP7) 73

13. References 76

14. Appendix: NMR Spectra and HPLC chromatograms 77

14.1 NMR spectra of the novel starting materials 77

14.2 NMR spectra of the (4+2)-cycloaddition products 92

14.3 NMR spectra of products of follow-up transformations 128

14.4 NMR spectra of products from reactions of oQMs with reference nucleophiles 130

14.5 HPLC chromatograms of the (4+2)-cycloaddition products 134

14.6 HPLC chromatogram of the follow up transformation 167

1. Determination of Electrophilicity Parameters *E* for *ortho*-Quinone Methides

Synthesis and characterisation of *o*QM1–*o*QM4

As shown in Fig. S1, the procedure for the synthesis of the heteroaryl-substituted ***o*QM1**, ***o*QM2**, and ***o*QM3** comprised a Friedel-Craft type acylation (*GP1*) to obtain the diarylketones **DAK**. Carbonate formation and subsequent reduction by sodium borohydride led to diaryl-substituted methanes **DAM** (*GP2*). Finally, oxidation of **DAM** with silver(I) oxide yielded stable, crystalline *o*QMs (*GP3*) (Fig. S1). We followed the reported procedure by Trauner et al. to prepare ***o*QM4** (Section 2).[1]

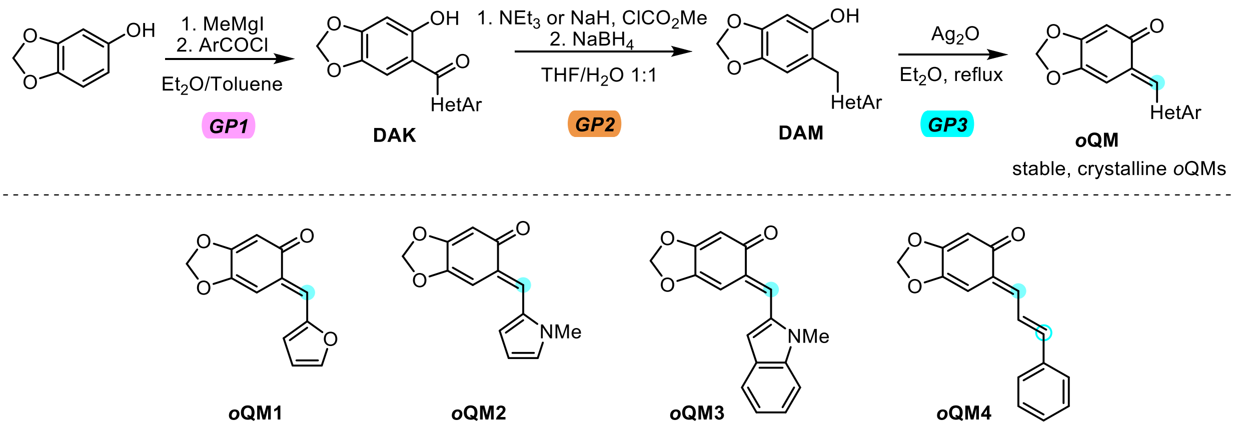

Fig. S1 Preparation of heteroaryl(HetAr)-substituted *o*QMs. Blue dots indicate the electrophilic positions of the *o*QMs.

Details of synthetic procedures for the preparation of *o*QMs are given in Section 3. Besides their spectroscopic characterization (Section 9 and Section 14.1), we also investigated the electrochemical reduction potentials of the *o*QMs (Section 10).

Electrophilicities of *o*QMs

In order to evaluate the Mayr electrophilicities *E* of ***o*QM1**–***o*QM4**, we measured the rate constants of the addition reactions of carbanionic reference nucleophiles **N1–N7** to the *o*QMs (Fig. S2).

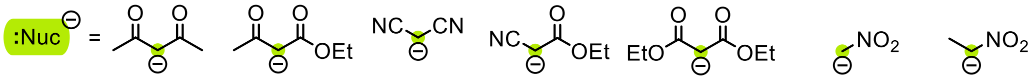

	N1	N2	N3	N4	N5	N6	N7
N =	17.64	18.82	19.36	19.62	20.22	20.71	21.54
s_N =	0.73	0.69	0.67	0.67	0.65	0.60	0.62

Fig. S2 Reference nucleophiles **N1**–**N7** (counterion: K^+) used to characterise the electrophilicity of *o*QMs (reactivity parameters N and s_N refer to DMSO solution).

The malononitrile-derived carbanion **N3** was selected to characterise typical reaction products of the kinetically investigated carbon-carbon bond-forming reactions (Section 12). We assume analogous reactions for all carbanion combinations with ***o*QM1**, ***o*QM2**, and ***o*QM3**, which feature the nucleophilic attack of the carbanions at the exocyclic CC π-bond of the *o*QMs as a common step (Fig. S3A). The unselective attack of **N3** at both electrophilic sites of ***o*QM4** (see Fig. S1) gave rise to two products (Section 12), analogous to previous investigations on the reactivities of vinyl *para*-quinone methides.[2]

The kinetics of *o*QM + carbanion reactions (Fig. S3A) were monitored photometrically at the absorbance maxima of the coloured *o*QMs by using stopped-flow or conventional UV-VIS spectroscopy. The carbanions were used in at least ten-fold excess over the *o*QMs, which allows for pseudo-first order reactions conditions. First-order rate constants k_{obs} (s^{-1}) were derived by least squares-fitting of the mono-exponential decay function $A = A_0 \exp(-k_{obs}t) + C$ to the time-dependent experimental absorbances A_t (Fig. S3B, raw data of the individual kinetic measurements that support the findings of this study are openly available in Open Data LMU at DOI: 10.5282/ubm/data.545.). The second order rate constants k_2^{exp} (M^{-1} s^{-1}) were then calculated as the slopes of the linear correlations of k_{obs} (s^{-1}) with four to five different carbanion concentrations (Fig. S3C). The second-order rate constants of all kinetically investigated *o*QM/carbanion combinations are listed in Table S1. Details of the individual kinetic measurements are given in Section 11.

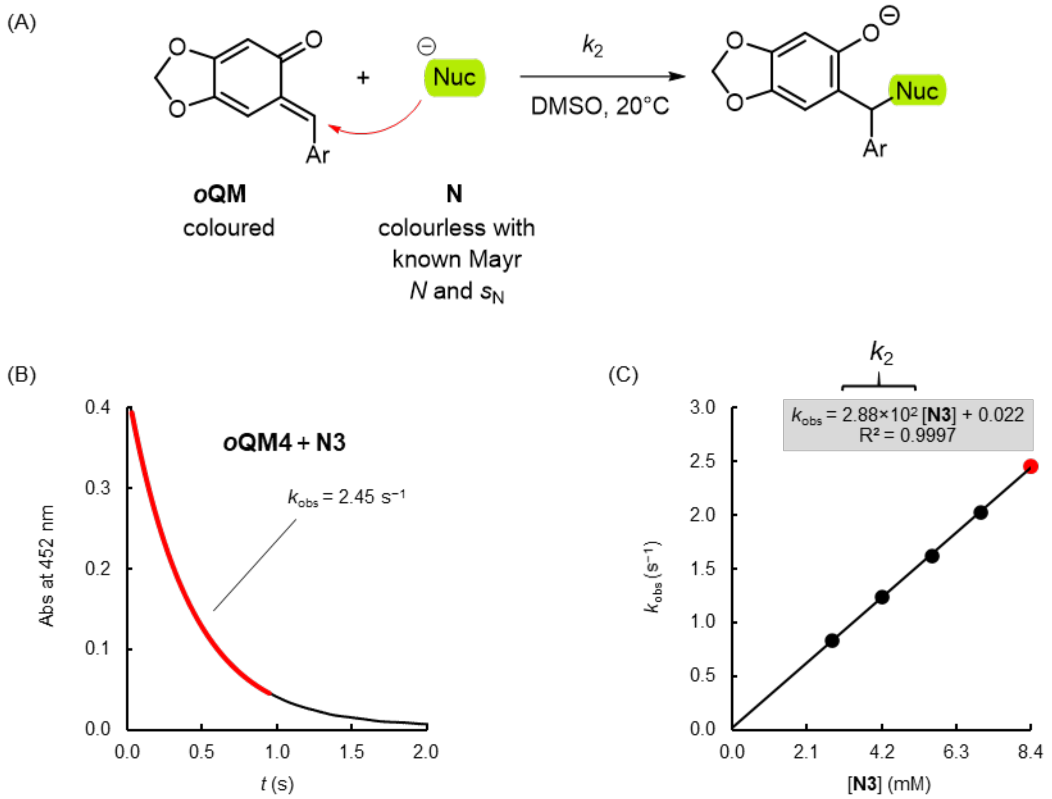

Fig. S3 (A) Carbon-carbon bond-forming reaction of an *o*QM with an anionic nucleophile. (B) Time-dependent decay of the *o*QM absorbance of ***o*QM4** ([***o*QM4**]$_0$ = 2.50 × 10^{-5} M) at 452 nm for the reaction with the carbanionic nucleophile **N3** ([**N3**]$_0$ = 2.40 × 10^{-3} M) in DMSO at 20°C and determination of the observed rate constant. (C) Linear correlation of experimentally determined first-order rate constants k_{obs} (s^{-1}) with the concentration of **N3** as required by the relationship k_{obs} = k_2[**N**]. The slope of the linear correlation corresponds to the second-order rate constant k_2 (M^{-1} s^{-1}).

Rearranging the Mayr-Patz equation (S1)

$\lg k_2(20\ °C) = s_N(N + E)$ (S1)

to equation (S2)

$(\lg k_2)/s_N = N + E$ (S2)

enables to depict the kinetic data of Table S1 as linear correlations (Figs. S4–S7) which illustrate the results of the least-square minimisations $\Delta^2 = (\lg k_2^{exp} - s_N(N + E))^2$ to calculate the electrophilicity E of the *o*QMs. The least-squares minimisations used the experimental second-order rate constants k_2^{exp} in DMSO at 20 °C from Table S1 and the reported nucleophile-specific reactivity parameters N and s_N of the reference nucleophiles **N1**–**N7** from ref.[3] as input in an MS Excel spreadsheet. The E parameter was defined as an adjustable variable and optimised for the individual *o*QMs by using the MS Excel Solver (GRG algorithm).

Table S1: Summary of experimentally determined second-order rate constants k_2^{exp} for the reactions of ***o*QM1**–***o*QM4** with carbanions **N1**–**N7** (reference nucleophiles) in DMSO at 20°C.

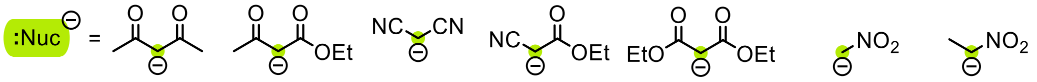

N1 **N2** **N3** **N4** **N5** **N6** **N7**

Nucleophiles	N (s_N)	k_2^{exp} (M^{-1} s^{-1})			
		***o*QM1**	***o*QM2**	***o*QM3**	***o*QM4**[a]
**N1**	17.64 (0.73)	3.84×10^1	7.92	6.00×10^1	n.d.
**N2**	18.82 (0.69)	1.08×10^2	1.28×10^1	1.57×10^2	6.27×10^1
**N3**	19.36 (0.67)	4.37×10^2	n.d.	6.50×10^2	2.40×10^2
**N4**	19.62 (0.67)	4.30×10^2	8.94×10^1	n.d.	2.88×10^2
**N5**	20.22 (0.65)	3.06×10^2	n.d.	4.89×10^2	3.32×10^2
**N6**	20.71 (0.60)	n.d.	8.04×10^1	n.d.	n.d.
**N7**	21.54 (0.62)	4.93×10^3	3.02×10^2	3.82×10^3	4.38×10^3
**Electrophilicity *E* of *o*QM**		**–15.73**	**–17.03**	**–15.55**	**–16.00**[a]

[a] Kinetic data for ***o*QM4** reflect the overall reactivity of the ambident electrophile.

The slopes in Figs. S4–S7 were set to unity as required by Equation (S1).

Fig. S8 shows that the electrophilicity parameters E of the heteroaryl-substituted ***o*QM1**–***o*QM3** correlate linearly with the electrochemical reduction potentials E_p^{red} (in MeCN).

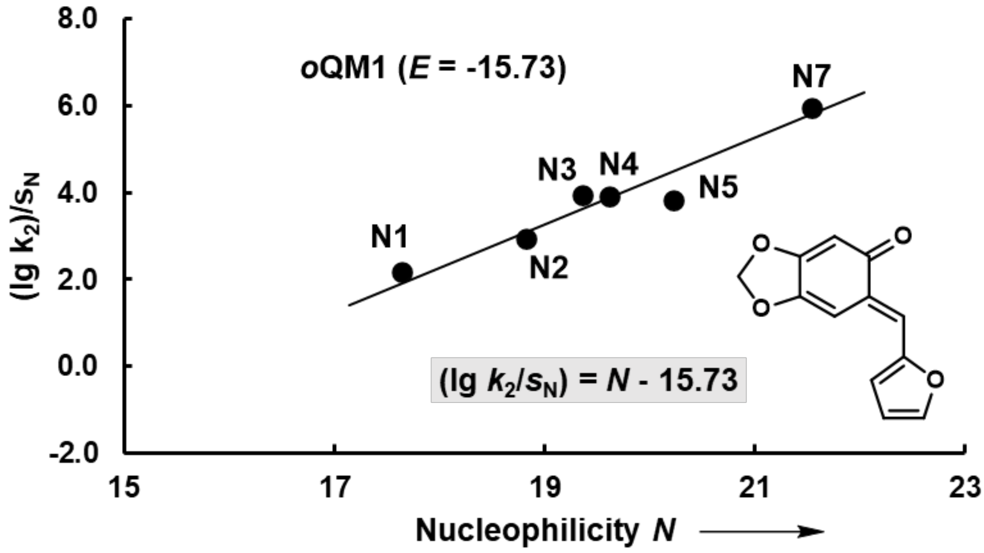

Fig. S4 Correlation of (lg k_2^{exp})/s_N vs. N for ***o*QM1**.

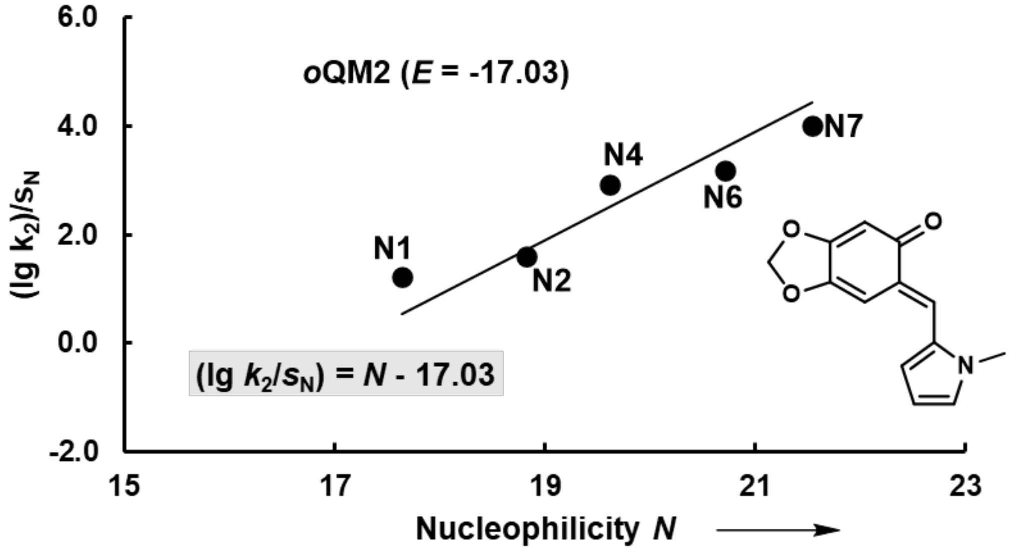

Fig. S5 Correlation of (lg k_2^{exp})/s_N vs. N for ***o*QM2**.

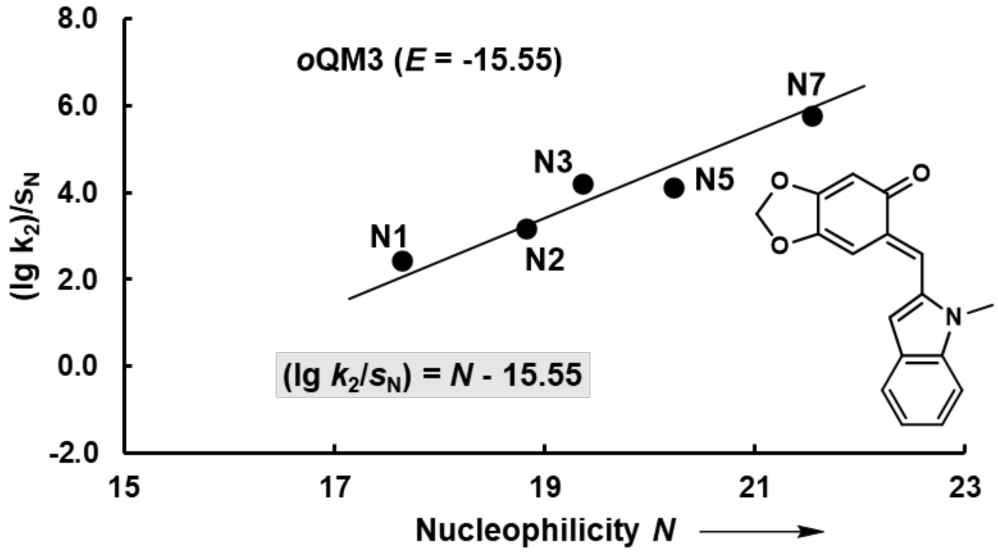

Fig. S6 Correlation of (lg k_2^{exp})/s_N vs. N for ***o*QM3**.

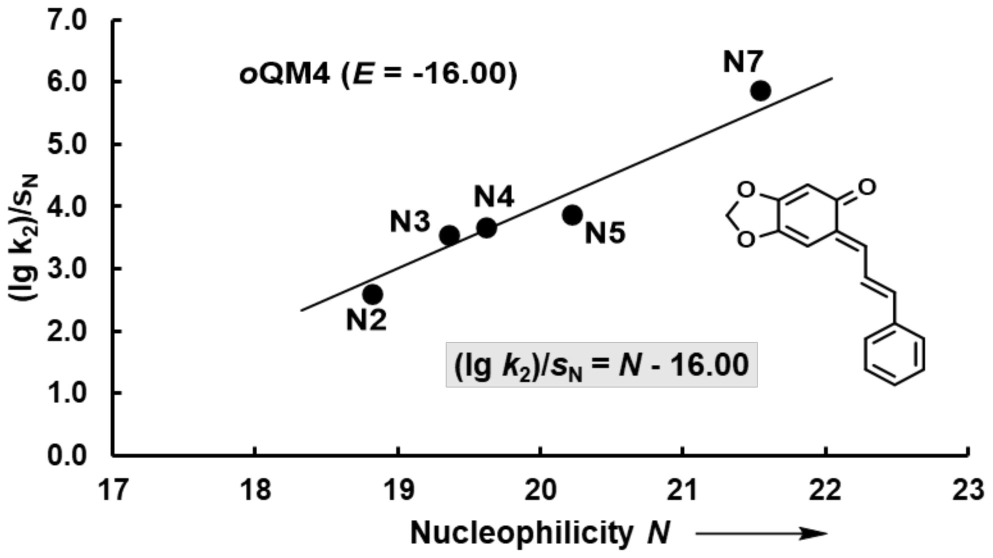

Fig. S7 Correlation of (lg k_2^{exp})/s_N vs. N for ***o*QM4**.

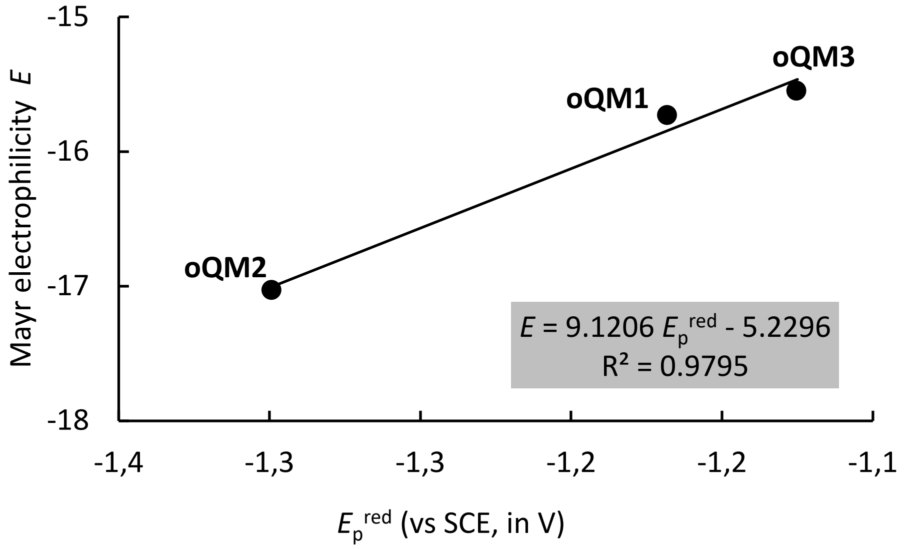

Fig. S8 Correlation of Mayr electrophilicity *E* vs. peak reduction potentials E_p^{red} of ***o*QM1**, ***o*QM2**, and ***o*QM3** (V vs. SCE in acetonitrile), values taken from Table S1 and Section 10.

2. General Information

At LMU München, nuclear magnetic resonance (NMR) spectra were recorded on 400, 600, and 800 MHz spectrometers. NMR signals were assigned based on information from additional 2D NMR experiments (COSY, gHSQC, gHMBC). Residual solvent signals were used as internal reference (δ_H 7.26 ppm, δ_C 77.16 ppm for $CDCl_3$, δ_H 2.50 ppm, δ_C 39.52 ppm for d_6-DMSO, δ_H 2.05 ppm, δ_C 29.84 ppm for d_6-acetone).

At JKU Linz, all NMR spectra were recorded using a Bruker Avance III 300 MHz spectrometer equipped with a broad band observe probe and a 16-slot sample changer, or a Bruker Avance DRX 500 MHz spectrometer. Both are property of the Austro Czech NMR Research Center "RERI uasb". The NMR spectra were referenced on the residual solvent peak of $CDCl_3$. (^{1}H NMR: δ = 7.26 ppm, ^{13}C NMR: δ = 77.16 ppm). NMR data are given as follows: the chemical shift (δ / ppm), integrals, multiplicity (s = singlet, d =doublet, t = triplet, q = quartet, m = multiplet) and coupling constants (J / Hz).

Infrared (IR) spectra were recorded on a Perkin Elmer Spectrum BX-59343 instrument with a Smiths Detection DuraSamplIR II Diamond ATR sensor or a Bruker Tensor 27 FT-IR instrument with a "Platinum" Diamond ATR sensor for detection in the range 4500–600 cm^{-1} as a film for liquids or neat for solids.

At LMU München, high resolution mass spectra (HRMS) were recorded on a Finnigan MAT 90, a Finnigan MAT 95, a JEOL MStation JMS 700, a Thermo Finnigan LTQ FT Ultra Fourier Transform ion cyclotron resonance, a Q Exactive GC Orbitrap GC/MS or a Thermo Fisher Scientific LTQ Orbitrap XL. For ionisation of the samples, either electron-impact ionization (EI) or electrospray ionization (ESI) was applied. At JKU Linz, HRMS were acquired in positive ionization mode using an Agilent 6520 Q-TOF mass spectrometer combined with an electrospray ionization (ESI) source.

Melting points were acquired using Büchi Melting Point B-560 devices and are not corrected.
Optical rotations were determined using a Schmidt+Haensch Unipol L 100 polarimeter, with $[\alpha]_D$ values reported in deg·cm^3·g^{-1}·dm^{-1} and concentrations (c) in g/100 mL.

Enantiomeric ratios (e.r.) were evaluated *via* HPLC analysis with a Dionex Summit, a Dionix Ultimate 3000 or a Shimadzu Prominence HPLC system, employing chiral stationary phases such as a CHIRAL ART Amylose-SA (4.6 mm × 250 mm, 5 µm), a CHIRAL ART Cellulose-SB (4.6 mm × 250 mm, 5 µm), CHIRAL ART Cellulose-SZ (4.6 mm × 250 mm, 5 µm), a CHIRALPAK® AD-H (4.6 mm × 250 mm, 5 µm), or a CHIRALCEL® OD-H (4.6 mm × 250 mm, 5 µm).

Semi-preparative HPLC was conducted on a Dionex Ultimate 3000 system, utilized with a Grace Alltima Silica 10 µm 250 × 10 mm column and variable wavelength detection.

Preparative column chromatography was performed using Davisil LC 60A 70– 200 MICRON silica gel, while Macherey-Nagel pre-coated TLC plates (silica gel 60, F254, 0.50 mm, SIL G-50) were used for preparative thin layer chromatography (TLC). The TLC plates were visualized under an UV lamp at 254 nm.

VCD spectra were recorded on a JASCO FVS-6000 spectrometer.

At LMU München, commercial reagents and dry solvents (stored over molecular sieves) were used without further purification as purchased from Sigma-Aldrich or Acros Organics. Diethyl ether and THF were dried over sodium and distilled. For thin-layer chromatography, silica gel plates with F-254 fluorescence indicator (Merck) were used. Purification by flash column chromatography was performed using Merck silica gel 60 (0.040–0.063 mm) with freshly distilled solvents. At JKU Linz, dry solvents were sourced from a mBRAUN SPS solvent purifier. All chemicals were obtained from commercial sources and used as received, unless specified otherwise. Solvents for extraction or chromatography were purchased as technical grade and distilled before use. Unless otherwise noted, the reactions were carried out under an inert atmosphere, using argon or nitrogen. **ITU1** ([885051-07-0]), **ITU2** ([1316861-19-4]), and **ITU3** ([1203507-02-1]) are commercially available from standard suppliers and were used in the purchased quality without any further purification or treatment. The selenium-based catalyst **ISeU** (Se-HyperBTM) was synthesized as reported previously.[4] The allenoates **1**[5] and **4** were synthesized in accordance to literature.[6,7]

1-Methyl-1*H*-pyrrole-2-carbonyl chloride (CG482). 1-Methylindole-2-carboxylic acid (1.48 g, 11.8 mmol) and DMF (5 drops) were dissolved in dry dichloromethane (30 mL) under argon atmosphere. Oxalyl chloride (1.96 g, 15.4 mmol) was added dropwise over 1 h at 0°C. The reaction mixture was stirred for another 16 h at room temperature. The solvent was removed under reduced pressure and the residue was dried under vacuum to yield a brown liquid (1.70 g, quantitative yield). ^{1}H NMR (400 MHz, $CDCl_3$): δ 7.27 (dd, J = 4.3, 1.8 Hz, 1 H), 6.98–6.97 (m, 1 H), 6.20 (dd, J = 4.3, 2.4 Hz, 1 H), 3.88 ppm (s, 3 H). The NMR spectroscopic data agree with those reported previously.[8]

1-Methyl-1*H*-indole-2-carbonyl chloride (CG490). 1-Methylindole-2-carboxylic acid (2.00 g, 11.4 mmol) and DMF (5 drops) were dissolved in dry dichloromethane (30 mL) under argon atmosphere. Oxalyl chloride (1.88 g, 14.8 mmol) was added dropwise over 1 h at 0°C. The reaction mixture was stirred for another 16 h at room temperature. The solvent was removed under reduced pressure, and the residue was dried under vacuum to yield an off-white powder (2.21 g, quantitative yield). ^{1}H NMR (400 MHz, $CDCl_3$): δ 7.74–7.71 (m, 1 H), 7.68 (s, 1 H), 7.48–7.44 (m, 1 H), 7.38–7.36 (m, 1 H), 7.22–7.18 (m, 1 H), 4.00 ppm (s, 3 H).

(*Z*)-6-((E)-3-Phenylallylidene)benzo[*d*][1,3]dioxol-5(6*H*)-one (*o*QM4) was synthesized according to a literature procedure.[1] NMR spectroscopic data agree with those described recently.[1]

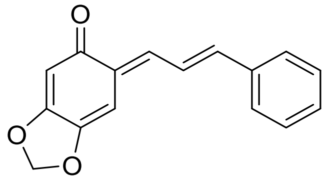

***o*QM4** (CG373)

^{1}H NMR (400 MHz, d_6-acetone): δ 7.73 (d, J = 6.7 Hz, 2 H), 7.67 (dd, J = 15.2, 12.3 Hz, 1 H), 7.46–7.35 (m, 4 H), 7.25 (d, J = 15.2 Hz, 1 H), 6.94 (s, 1 H), 6.05 (s, 2 H), 5.79 ppm (s, 1 H).

^{13}C{^{1}H} NMR (101 MHz, d_6-acetone): δ 184.5, 162.4, 146.6, 144.0, 140.1, 137.7, 131.5, 130.2, 129.7, 128.6, 124.6, 103.4, 101.5, 98.7 ppm.

IR (neat, ATR): $\tilde{\nu}$ 3054, 2917, 1618, 1591, 1524, 1427, 1365, 1227, 1208, 1093, 974, 957, 862, 811, 749, 690 cm^{-1}.

HRMS (EI): *m*/*z* calcd for $C_{16}H_{12}O_3^{\bullet+}$ [$M^{\bullet+}$]: 252.0781; found: 252.0779.

3. Syntheses of Novel Starting Materials

3.1 Synthesis of *ortho*-quinone methides (*o*QM) (GP1–GP3)

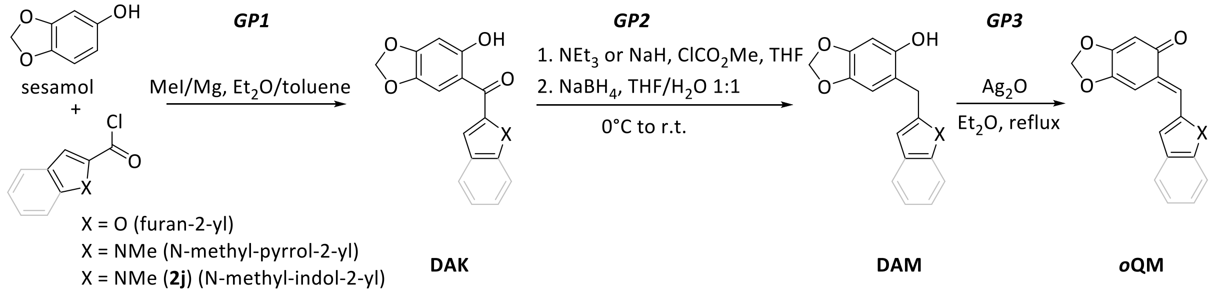

General Procedure (GP1). In this work, diarylketones (**DAK**) were prepared by following *General Procedure 1 (GP1)*, which is based on a literature procedure.[9] Magnesium turnings were flame-dried in a three-necked flask under vacuum and flushed with dry nitrogen. Methyl iodide was added to a suspension of magnesium and diethyl ether (10 mL) under reflux and the mixture was stirred until complete reaction of the magnesium. Sesamol, dissolved in diethyl ether (5 mL), was added dropwise, and the reaction mixture was stirred for 2 h. The solvent was removed under vacuum and the residue was suspended in dry toluene (30 mL). Aryl chloride was dissolved in toluene (5 mL) and added dropwise at 0°C over a period of 20 min. The reaction mixture was stirred for 20 h at room temperature (approx. 23 °C). The mixture was quenched with aq. saturated NH_4Cl solution (20 mL) and extracted with ethyl acetate (4 × 30 mL). The combined organic phases were washed with brine (2 × 30 mL) and dried over $MgSO_4$. The solvent was removed under reduced pressure and the crude residue was purified by silica gel chromatography and crystallised from *n*-hexane/dichloromethane mixtures to afford the **DAK**.

General Procedure (GP2). Diarylmethanes (**DAM**) were prepared from **DAK** according to *General Procedure 2 (GP2)*, which is based on a literature procedure.[9] In a flame-dried round-bottom flask under nitrogen ketones **DAK** were dissolved in dry THF. Triethylamine and methyl chloroformate were added and the reaction mixture was stirred for 1 h at 0°C.The mixture was filtered to remove precipitated triethylammonium chloride. Then the filtrate was reduced to half the volume by solvent evaporation at the rotary evaporator. The carbonate solution in THF was added to a mixture of $NaBH_4$ in water at 0°C, resulting in an approximately 1:1 mixture of THF/H_2O. The reaction mixture was stirred for 3 h at room temperature. The mixture was quenched with water (20 mL) and extracted with diethyl ether (4 × 30 mL). The combined organic phases were washed with brine (30 mL) and dried over $MgSO_4$. The solvent was removed under reduced pressure at the rotary evaporator. The crude product was purified by chromatography on silica gel to afford **DAM**.

General Procedure (GP3). *Ortho*-quinone methides (***o*QMs**) were prepared by following *General Procedure 3 (GP3)* in analogy to a procedure that was reported in Ref. [9]. **DAM** was dissolved in dry diethyl ether under argon atmosphere and treated with silver(I) oxide. The reaction mixture was stirred at 42°C for 5 h. Then the mixture was filtered. The volume of the filtrate was reduced to one third of the original volume by solvent evaporation at the rotary evaporator. Crystals formed upon cooling the remaining solution in a freezer (–18 °C). The crystalline ***o*QMs** were isolated by filtration.

3.2 General procedure for the synthesis of *o*QM precursors 7 (GP4)

For the synthesis of the *o*QM precursors **7** a two step literature procedure was applied.[10] Thereby, a flame-dried reaction flask was used under inert conditions and a suspension of magnesium turnings (2.5 equiv.) in dry THF (1.0 M) was added. A solution of aryl bromide (ArBr, 2.5 equiv.) in dry THF (0.6 M) was then added dropwise. Upon completion, the reaction was stirred at room temperature for 30 min, followed by refluxing for 1 h. The reaction mixture was subsequently cooled and a solution of the aldehyde (1 equiv.) in THF (0.8 M) was added dropwise. After stirring overnight, the reaction is quenched with saturated NH_4Cl solution and extracted 3 times with DCM. The organic layers are combined, washed with brine, dried over anhydrous Na_2SO_4, filtered and consequently evaporated to dryness in order to yield the crude product. The resulting product was then purified *via* column chromatography (heptane:EtOAc = 10:1 to 5:1).

For the second part of the reaction, $TolSO_2Na$ (1.15 equiv.) and TsOH (1.75 equiv.) are dissolved in DCM (0.2 M). The previously formed product was then added to the suspension while stirring gently. The reaction mixture was stirred for 2 h at room temperature and subsequently extracted with DCM (3 ×). The combined phases were washed with brine and dried over anhydrous Na_2SO_4. The solvent was then removed under reduced pressure and the product purified by column chromatography (heptane:EtOAc = 10:1 to 2:1) yielding the *o*QM precursors **7**.

4. (4+2)-Cycloadditions

4.1 General remarks

In all cases, the (4+2)-cycloaddition reactions predominantly yielded the (*Z*)-diastereomer and only traces of the (*E*)-diastereomers were formed. Besides this also in some cases noteworthy amounts of products **6** were found, originated from the α-attack of the allenoate on the benzylic position of the o-QM. Both side products could be easily removed by column chromatography. Diastereomeric ratios, conversions and screening yields were evaluated from the crude ^{1}H-NMR spectra using the internal standard mesitylene.

4.2 General procedure for the (4+2)-cycloaddition starting from preformed oQMs 2 (GP5)

The reaction was conducted under an inert nitrogen atmosphere in a flame dried Schlenk flask. First, the flask was charged with the catalyst **ITU3** (20 mol%), the respective o-QM (0.1 mmol, 1 equiv.) and dissolved in toluene (5 mL, 0.02 mol L^{-1}), directly followed by the addition of the allenoate **1** or **4** (0.15 mmol, 1.5 equiv.). Moreover, using Okamoto's achiral catalyst (DHPB 20 mol%), the corresponding racemic products were formed. The reaction mixture was then allowed to stir at 80 °C for 24 h. After cooling, the mixture was filtered through a Na_2SO_4 plug. The solvent was then removed under reduced pressure to yield the crude products **3a-q** and **6r**. Consequent purification *via* preparative TLC (heptane:EtOAc = 2:1) gave the products in high purities and moderate to good yields.

4.3 General procedure for the (4+2)-cycloaddition starting from precursors 7 (GP6)

The reaction was carried out in a flame-dried Schlenk flask under an inert nitrogen atmosphere. The flask was charged with the catalyst **ISeU** (20 mol%), Cs_2CO_3 (0.1 mmol, 1 equiv.), the respective o-QM precursor **7** (0.1 mmol, 1 equiv.) and subsequently suspended in toluene (5 mL, 0.02 mol L^{-1}). The allenoate (0.3 mmol, 3 equiv.) was added, the mixture was heated to 40 °C and stirred for 24 h. When using Okamoto's achiral catalyst (DHPB, 20 mol%) for **3aa-ah**, **3aj**, **6aq** or racemic **ITU3** (obtained by mixing both enantiomers of enantiopure **ITU3**) for **3ai**, **3am**, **3ao and 3ap**, the corresponding racemic products were synthesized (NOTE: *because of this manual mixing of the two enantiomers of ITU3 when preparing the "racemic" HPLC samples, these do not always show a perfect 50:50 ratio of the two enantiomers*). Upon cooling, the mixture was filtered through a Na_2SO_4 plug and the solvent was removed under reduced pressure to give the crude product **3**. The product was then purified *via* preparative TLC using (heptane:EtOAc = 2:1), resulting high purity and good yields. (NOTE: *One side product hereby was the addition product of the leaving group to the allenoate. This side product was often difficult to remove via simple column chromatography or prep. TLC and thus was removed by means of semi-preparative HPLC if necessary).*

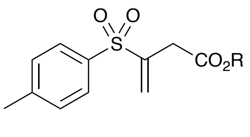

5. Characterization of Novel Starting Materials

Furan-2-yl(6-hydroxybenzo[*d*][1,3]dioxol-5-yl)methanone (DAK1) was prepared (*GP1*) from magnesium (0.40 g, 16 mmol), methyl iodide (3.85 g, 27.1 mmol), sesamol (1.50 g, 10.9 mmol), and 2-furoyl chloride (1.42 g, 10.9 mmol). Purification of the crude material by flash chromatography (silica gel, *n*-pentane:EtOAc = 19:1) gave **DAK1** as two yellow fractions. One pure fraction as yellow crystals (0.035 g) that was used for spectroscopic analysis and a crude product (2.40 g), which was directly used in the next step without further purification; m.p. 103 °C.

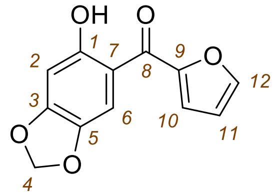

DAK1 (CG454_1)

^{1}H NMR (400 MHz, $CDCl_3$): δ 13.27 (s, 1 H, 1-OH), 7.79 (s, 1 H, 6-H), 7.70 (dd, J = 1.7, 0.8 Hz, 1 H, 12-H), 7.37 (dd, J = 3.6, 0.8 Hz, 1 H, 10-H), 6.61 (dd, J = 3.6, 1.8 Hz, 1 H, 11-H), 6.51 (s, 1 H, 2-H), 6.01 ppm (s, 2 H, 4-H).

^{13}C{^{1}H} NMR (101 MHz, $CDCl_3$): δ 183.0 (C_q, C-8), 164.3 (C_q, C-1), 154.6 (C_q, C-3), 152.6 (C_q, C-9), 146.7 (CH, C-12), 140.9 (C_q, C-5), 120.1 (CH, C-10), 112.5 (CH, C-11), 110.9 (C_q, C-7), 108.3 (CH, C-6), 102.1 (CH_2, C-4), 99.0 ppm (CH, C-2).

IR (neat, ATR): $\tilde{\nu}$ 3132, 2921, 1734, 1621, 1583, 1481, 1463, 1419, 1326, 1252, 1213, 1167, 1031, 1021, 927, 864, 827, 771, 752, 706 cm^{-1}.

HRMS (EI): *m*/*z* calcd for $C_{12}H_8O_5^{\bullet+}$ [$M^{\bullet+}$]: 232.0366; found: 232.0365.

(6-Hydroxybenzo[*d*][1,3]dioxol-5-yl)(1-methyl-1*H*-pyrrol-2-yl)methanone (DAK2) was prepared (*GP1*) from magnesium (0.34 g, 14 mmol), methyl iodide (3.34 g, 23.5 mmol), sesamol (1.30 g, 9.41 mmol), and pyrrole 2-carbonyl chloride (1.49 g, 10.4 mmol). Purification of the crude material by flash chromatography (silica gel, *n*-pentane:EtOAc = 19:1 → 9:1) and crystallisation (from *n*-hexane/CH_2Cl_2) gave **DAK2** (0.360 g, 16%) as yellow crystals; m.p. 104 °C.

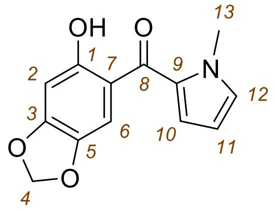

DAK2 (CG484)

R_f (silica, *n*-pentane/EtOAc = 19:1, UV) = 0.30

^{1}H NMR (400 MHz, $CDCl_3$): δ 12.77 (s, 1 H, 1-H), 7.34 (s, 1 H, 6-H), 6.90–6.89 (m, 1 H, 12-H), 6.78–6.76 (m, 1 H, 10-H), 6.51 (s, 1 H, 2-H), 6.20–6.18 (m, 1 H, 11-H), 5.98 (s, 2 H, 4-H), 3.93 ppm (s, 3 H, 13-H).

^{13}C {^1H} **NMR** (101 MHz, $CDCl_3$): *δ* 187.8 (C_q, C-8), 162.1 (C_q, C-1), 153.5 (C_q, C-3), 140.2 (C_q, C-5), 130.9 (CH, C-12), 129.7 (C_q, C-9), 121.1 (CH, C-10), 112.8 (C_q, C-7), 109.8 (CH, C-6), 108.4 (CH, C-11), 101.8 (CH_2, C-4), 98.9 (CH, C-2), 36.9 ppm (CH_3, C-13).

IR (neat, ATR): $\tilde{\nu}$ 2918, 1624, 1580, 1483, 1405, 1295, 1244, 1194, 1140, 1078, 1031, 931, 874, 863, 832, 732, 689 cm^{-1}.

HRMS (EI): *m*/*z* calcd for $C_{13}H_{11}NO_4^{\bullet+}$ [$M^{\bullet+}$]: 245.0683; found: 245.0683.

(6-Hydroxybenzo[*d*][1,3]dioxol-5-yl)(1-methyl-1*H*-indol-2-yl)methanone (DAK3) was prepared (*GP1*) from magnesium (0.37 g, 15 mmol), methyl iodide (3.60 g, 25.4 mmol), sesamol (1.40 g, 10.1 mmol), and indole 2-carbonyl chloride (2.16 g, 11.1 mmol, added portion by portion to the reaction mixture). Purification of the crude material by flash chromatography (silica gel, *n*-pentane:EtOAc = 19:1 → 9:1) and crystallisation (from *n*-hexane/CH_2Cl_2) gave **DAK3** (1.29 g, 43%) as yellow crystals; m.p. 179 °C.

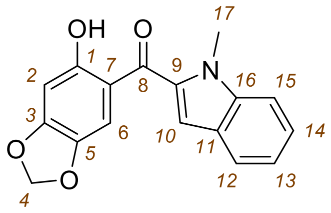

DAK3 (CG491)

R_f (silica, *n*-pentane/EtOAc = 19:1, UV) = 0.40.
^1H **NMR** (400 MHz, $CDCl_3$): *δ* 12.91 (s, 1 H, 1-H), 7.72–7.69 (m, 1 H, 12-H), 7.44–7.38 (m, 3 H, 6-H, 14-H, and 15-H), 7.21–7.17 (m, 1 H, 13-H), 7.00 (s, 1 H, 10-H), 6.56 (s, 1 H, 2-H), 6.01 (s, 2 H, 4-H), 3.98 ppm (s, 3 H, 17-H).

^{13}C{^1H} **NMR** (101 MHz, $CDCl_3$): *δ* 190.0 (C_q, C-8), 163.2 (C_q, C-1), 154.5 (C_q, C-3), 140.5 (C_q, C-5), 139.9 (C_q, C-16), 134.6 (C_q, C-9), 126.2 (C_q, C-11), 125.6 (CH, C-14), 122.7 (CH, C-12), 121.0 (CH, C-13), 113.1 (C_q, C-7), 112.1 (CH, C-10), 110.4 (CH, C-15), 110.0 (CH, C-6), 102.1 (CH_2, C-4), 98.9 (CH, C-2), 31.9 ppm (CH_3, C-17).

IR (neat, ATR): $\tilde{\nu}$ 2904, 1619, 1585, 1509, 1474, 1430, 1385, 1309, 1246, 1229, 1187, 1166, 1038, 939, 845, 734 cm^{-1}.

HRMS (EI): *m*/*z* calcd for $C_{17}H_{13}NO_4^{\bullet+}$ [$M^{\bullet+}$]: 295.0839; found: 295.0839.

6-(Furan-2-ylmethyl)benzo[*d*][1,3]dioxol-5-ol (DAM1) was prepared (*GP2*) from crude **DAK1** (2.40 g, approx. 10 mmol), triethylamine (1.57 g, 15.5 mmol), methyl chloroformate (1.12 g, 11.9 mmol) in THF (40 mL). After filtration, the solvent was removed under reduced pressure at the rotary evaporator and purification of the crude material by flash chromatography (silica gel, eluent: *n*-pentane:EtOAc = 8:2) yielded the carbonate [1.13 g, 3.89 mmol, R_f (silica, *n*-pentane/EtOAc 8:2, UV) = 0.2].

The carbonate (1.13 g, 3.89 mmol) was dissolved in THF (20 mL) and sodium borohydride (0.589 g, 15.6 mmol, in 20 mL water) was added. Purification of the crude material by flash chromatography (silica gel, eluent: *n*-pentane:EtOAc = 8:2) furnished **DAM1** (0.790 g, 93%, calculated from the carbonate) as a colourless oil.

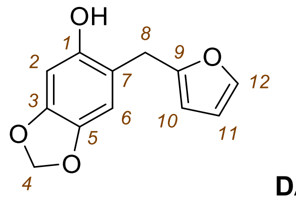

DAM1 (CG457_1)

R_f (silica, pentane/EtOAc 8:2, UV) = 0.60.

^{1}H NMR (600 MHz, $CDCl_3$): δ 7.34–7.33 (m, 1 H, 12-H), 6.62 (s, 1 H, 6-H), 6.44 (s, 1 H, 2-H), 6.30–6.29 (m, 1 H, 11-H), 6.06–6.05 (m, 1 H, 10-H), 5.89 (s, 2 H, 4-H), 5.00 (s, 1 H, 1-OH), 3.87 ppm (s, 2 H, 8-H).

^{13}C{^{1}H} NMR (151 MHz, $CDCl_3$): δ 153.7 (C_q, C-9), 148.4 (C_q, C-1), 147.1 (C_q, C-3), 141.8 (CH, C-12), 141.7 (C_q, C-5), 116.3 (C_q, C-7), 110.6 (CH, C-11), 109.7 (CH, C-6), 106.2 (CH, C-10), 101.2 (CH_2, C-4), 99.1 (CH, C-2), 29.3 ppm (CH_2, C-8).

HRMS (EI): *m*/*z* calcd for $C_{12}H_{10}O_4^{\bullet+}$ [$M^{\bullet+}$]: 218.0574; found: 218.0572.

6-((1-Methyl-1*H*-pyrrol-2-yl)methyl)benzo[*d*][1,3]dioxol-5-ol (DAM2) was prepared (*GP2*) from **DAK2** (0.360 g, 1.47 mmol), sodium hydride (60% dispersion in mineral oil, 0.088 g, 2.21 mmol), methyl chloroformate (0.160 g, 1.69 mmol) in THF (30 mL). After filtration, the solution was reduced to half of the volume by solvent evaporation at the rotary evaporator. Then, sodium borohydride (0.222 g, 5.87 mmol in 15 mL water) was added. Purification of the crude material by flash chromatography (silica gel, eluent: *n*-pentane:EtOAc = 8:2) furnished **DAM2** (0.312 g, 92%) as a colourless oil.

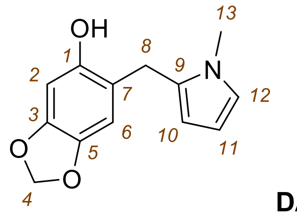

DAM2 (CG488)

R_f (silica, n-*pentane*/EtOAc 8:2, UV) = 0.40.

^{1}H NMR (400 MHz, $CDCl_3$): δ 6.61–6.60 (m, 1 H, 12-H), 6.56 (s, 1 H, 6-H), 6.40 (s, 1 H, 2-H), 6.07 (t, *J* = 3.1 Hz, 1 H, 11-H), 6.00–5.98 (m, 1 H, 10-H), 5.88 (s, 2 H, 4-H), 5.11 (s, 1 H, 1-OH), 3.86 (s, 2 H, 8-H), 3.45 ppm (s, 3 H, 13-H).

^{13}C{^{1}H} NMR (101 MHz, $CDCl_3$): δ 149.3 (C_q, C-1), 147.0 (C_q, C-3), 141.6 (C_q, C-5), 129.7 (C_q, C-9), 123.3 (CH, C-12), 116.4 (C_q, C-7), 109.5 (CH, C-6), 107.7 (CH, C-10), 107.2 (CH, C-11), 101.1 (CH_2, C-4), 99.2 (CH, C-2), 34.1 (CH_3, C-13), 29.0 ppm (CH_2, C-8).

IR (neat, ATR): $\tilde{\nu}$ 3438, 2894, 1633, 1503, 1483, 1444, 1297, 1168, 1037, 934, 854, 761, 713 cm^{-1}.

HRMS (EI): *m*/*z* calcd for $C_{13}H_{13}NO_3^{\bullet+}$ [$M^{\bullet+}$]: 231.0890; found: 231.0889.

6-((1-Methyl-1*H*-indol-2-yl)methyl)benzo[*d*][1,3]dioxol-5-ol (DAM3) was prepared (*GP2*) from **DAK3** (1.29 g, 4.37 mmol), triethylamine (0.796 g, 7.87 mmol), methyl chloroformate (0.620 g, 6.56 mmol) in THF (40 mL). After filtration, the solution was reduced to half of the volume by solvent evaporation at the rotary evaporator before sodium borohydride (1.32 g, 34.9 mmol in 20 mL water) was added. Purification of the crude material by flash chromatography (silica gel, eluent: *n*-pentane:EtOAc = 9:1→8:2) furnished **DAM3** (1.14 g, 93%) as an off-white solid; m.p. 134 °C.

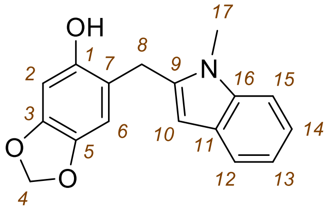

DAM3 (CG492)

^{1}H NMR (400 MHz, $CDCl_3$): δ 7.56–7.54 (m, 1 H, 12-H), 7.29–7.27 (m, 1 H, 15-H), 7.22–7.18 (m, 1 H, 14-H), 7.13–7.08 (m, 1 H, 13-H), 6.58 (s, 1 H, 6-H), 6.42 (s, 1 H, 2-H), 6.32 (q, *J* = 0.9 Hz, 1 H, 10-H), 5.90 (s, 2 H, 4-H), 4.89 (s, 1 H, 1-OH), 4.04 (s, 2 H, 8-H), 3.62 ppm (s, 3 H, 17-H).

^{13}C{^{1}H} NMR (101 MHz, $CDCl_3$): δ 148.7 (C_q, C-1), 147.1 (C_q, C-3), 141.9 (C_q, C-5), 138.2 (C_q, C-9), 138.2 (C_q, C-16), 127.8 (C_q, C-11), 121.4 (CH, C-14), 120.3 (CH, C-12), 119.7 (CH, C-13), 116.1 (C_q, C-7), 109.7 (CH, C-6), 109.1 (CH, C-15), 101.3 (CH_2, C-4), 100.7 (CH, C-10), 99.1 (CH, C-2), 29.9 (CH_3, C-17), 28.7 ppm (CH_2, C-8).

IR (neat, ATR): $\tilde{\nu}$ 3566, 2921, 1502, 1477, 1275, 1175, 1112, 1036, 929, 874, 860, 744 cm^{-1}.

HRMS (EI): *m*/*z* calcd for $C_{17}H_{15}NO_3^{\bullet+}$ [$M^{\bullet+}$]: 281.1046; found: 281.1047.

(*E*)-6-(Furan-2-ylmethylene)benzo[*d*][1,3]dioxol-5(6*H*)-one (*o*QM1) was prepared (*GP3*) from **DAM1** (0.750 g, 3.44 mmol) and silver(I) oxide (2.39 g, 10.3 mmol) in diethyl ether (80 mL) to yield ***o*QM1** (0.230 g, 31%) as red crystals; m.p. >127 °C (dec.).

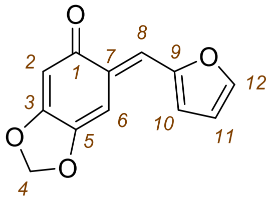

***o*QM1** (CG459)

^{1}H NMR (400 MHz, d_6-DMSO): δ 8.03 (d, *J* = 1.9 Hz, 1 H, 12-H), 7.34 (s, 1 H, 8-H), 7.23 (d, *J* = 3.5 Hz, 1 H, 10-H), 7.15 (s, 1 H, 6-H), 6.74 (dd, *J* = 3.5, 1.9 Hz, 1 H, 11-H), 6.08 (s, 2 H, 4-H), 5.92 ppm (s, 1 H, 2-H).

^{13}C{^{1}H} NMR (101 MHz, *d*$_6$-DMSO): *δ* 183.8 (C$_q$, C-1), 161.3 (C$_q$, C-3), 152.1 (C$_q$, C-9), 147.7 (CH, C-12), 146.1 (C$_q$, C-5), 126.0 (C$_q$, C-7), 123.8 (CH, C-8), 121.3 (CH, C-10), 113.5 (CH, C-11), 102.8 (CH$_2$, C-4), 100.3 (CH, C-2), 98.9 ppm (CH, C-6).

IR (neat, ATR): $\tilde{\nu}$ 3124, 1628, 1561, 1518, 1466, 1420, 1348, 1220, 1026, 1009, 945, 931, 849, 827, 746, 706 cm^{-1}.

HRMS (EI): *m*/*z* calcd for $C_{12}H_8O_4^{\bullet+}$ [M$^{\bullet+}$]: 216.0417; found: 216.0416.

(*E*)-6-((1-Methyl-1*H*-pyrrol-2-yl)methylene)benzo[*d*][1,3]dioxol-5(6*H*)-one (*o*QM2) was prepared (*GP3*) from **DAM2** (0.310 g, 1.34 mmol) and silver(I) oxide (0.932 g, 4.02 mmol) in diethyl ether (40 mL). The reaction mixture was filtered, and the filtration residue was washed with acetone (100 mL). The combined organic phases were evaporated to dryness. The red residue was dissolved in acetone (30 mL). The volume of the acetone solution was reduced to ca. 10 mL by solvent evaporation at the rotary evaporator, which led to precipitation of ***o*QM2** (0.121 g, 39%) as dark red crystals; m.p. >120 °C (dec.).

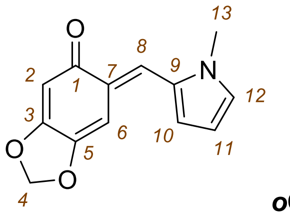

***o*QM2** (CG489)

^{1}H NMR (400 MHz, *d*$_6$-DMSO): *δ* 7.61 (s, 1 H, 8-H), 7.23 (br s, 1 H, 12-H), 6.96 (d, *J* = 4.5 Hz, 1 H, 10-H), 6.88 (s, 1 H, 6-H), 6.29–6.28 (m, 1 H, 11-H), 6.04 (s, 2 H, 4-H), 5.90 (s, 1 H, 2-H), 3.74 ppm (s, 3 H, 13-H).

^{13}C{^{1}H} NMR (101 MHz, *d*$_6$-DMSO): *δ* 183.3 (C$_q$, C-1), 160.8 (C$_q$, C-3), 145.1 (C$_q$, C-5), 129.9 (CH, C-12), 129.1 (C$_q$, C-9), 127.1 (CH, C-8), 124.3 (C$_q$, C-7), 116.1 (CH, C-10), 110.8 (CH, C-11), 102.3 (CH$_2$, C-4), 100.7 (CH, C-2), 99.1 (CH, C-6), 34.0 ppm (CH$_3$, C-13).

IR (neat, ATR): $\tilde{\nu}$ 3100, 1617, 1532, 1518, 1484, 1432, 1421, 1374, 1344, 1280, 1212, 1077, 1028, 944, 870, 841, 719, 704 cm^{-1}.

HRMS (pos. ESI): *m*/*z* calcd for $C_{13}H_{12}NO_3^+$ [M + H$^+$]: 230.0812; found: 230.0818.

(*E*)-6-((1-Methyl-1*H*-indol-2-yl)methylene)benzo[*d*][1,3]dioxol-5(6*H*)-one (*o*QM3) was prepared (*GP3*) from **DAM3** (1.14 g, 4.05 mmol) and silver(I) oxide (2.82 g, 12.2 mmol) in diethyl ether (80 mL). The reaction mixture was filtered, and the filtration residue was washed with acetone (500 mL). The combined organic phases were evaporated to dryness. The red residue was dissolved in acetone (150 mL). The volume of the acetone solution was reduced to ca. 50 mL by solvent evaporation at the rotary evaporator, which led to precipitation of ***o*QM3** (0.730 g, 65%) as claret-red crystals; m.p. >135 °C (dec.).

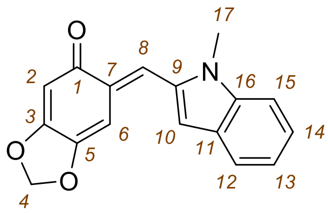

***o*QM3** (CG495)

^{1}**H NMR** (800 MHz, *d*$_6$-DMSO): *δ* 7.74 (s, 1 H, 8-H), 7.63 (d, *J* = 7.9 Hz, 1 H, 12-H), 7.53 (d, *J* = 8.4 Hz, 1 H, 15-H), 7.27–7.25 (m, 1 H, 14-H), 7.21 (s, 1 H, 10-H), 7.09–7.07 (m, 1 H, 13-H), 6.99 (s, 1 H, 6-H), 6.10 (s, 2 H, 4-H), 5.99 (s, 1 H, 2-H), 3.83 ppm (s, 3 H, 17-H).

^{13}**C{^{1}H} NMR** (201 MHz, *d*$_6$-DMSO): *δ* 183.6 (C$_q$, C-1), 161.5 (C$_q$, C-3), 147.1 (C$_q$, C-5), 138.7 (C$_q$, C-16), 134.3 (C$_q$, C-9), 129.3 (C$_q$, C-7), 127.7 (C$_q$, C-11), 126.5 (CH, C-8), 124.1 (CH, C-14), 121.5 (CH, C-12), 120.3 (CH, C-13), 110.3 (CH, C-15), 107.2 (CH, C-10), 102.9 (CH$_2$, C-4), 100.5 (CH, C-2), 98.6 (CH, C-6), 29.9 ppm (CH$_3$, C-17).

IR (neat, ATR): $\tilde{\nu}$ 2899, 1619, 1530, 1423, 1368, 1351, 1324, 1212, 1180, 1152, 1040, 947, 725, 702, 688 cm^{-1}.

HRMS (EI): *m*/*z* calcd for $C_{17}H_{13}NO_3^{\bullet+}$ [M$^{\bullet+}$]: 279.0890; found: 279.0897.

1-((4-(*tert*-Butyl)phenyl)(tosyl)methyl)naphthalen-2-ol (7a)

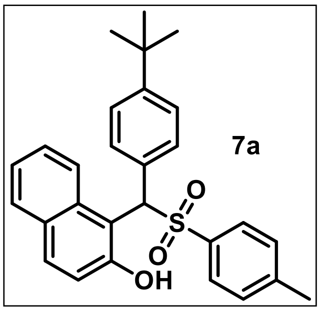

7a

The o-QM precursor **7a** was synthesized following the general procedure *GP4***.** After purification by means of column chromatography the product was obtained as a brown solid in an overall yield of 74%.

^{1}**H-NMR** (300 MHz, CDCl$_3$, 298 K) *δ* / ppm: 8.92 (s, 1H, O-**H**), 7.72 – 7.61 (m, 7H, Ar-**H**), 7.39 – 7.35 (m, 2H, Ar-**H**), 7.33 – 7.29 (m, 1H, Ar-**H**), 7.25 – 7.19 (m, 2H, Ar-**H**), 7.02 (d, J = 8.1 Hz, 2H, Ar-**H**), 6.49 (s, 1H, -C**H**), 2.22 (s, 3H, -C**H$_3$**), 1.29 (s, 9H, -C**H$_3$**).

^{13}**C-NMR** (75 MHz, CDCl$_3$, 298 K) *δ* / ppm = 154.7 (1 **C**), 151.9 (1 **C**), 145.2 (1 **C**), 134.5 (1 **C**), 133.0 (1 **C**), 131.8 (1 **C**), 129.7 (2 **C**), 129.4 (2 **C**), 129.4 (1 **C**), 129.0 (1 **C**), 128.6 (4 **C**), 127.3 (1 **C**), 125.9 (2 **C**), 123.3 (1 **C**), 121.1 (1 **C**), 110.9 (1 **C**), 70.0 (1 **C**), 34.7 (1 **C**), 31.3 (3 **C**), 21.6 (1 **C**).

HRMS (ESI-TOF): *m/z* calculated for $C_{28}H_{28}O_3S$: 467.1651 [M+Na]$^+$, found: 467.1654.

1-(Tosyl(4-(trifluoromethyl)phenyl)methyl)naphthalen-2-ol (7b)

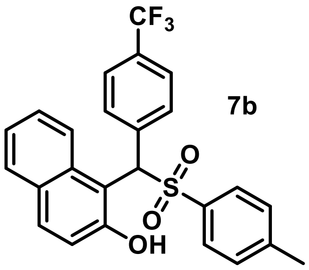

7b

The o-QM precursor **7b** was synthesized following the general procedure *GP4***.** After purification by means of column chromatography the product was obtained as brown solid in an overall yield of 32%.

^{1}H-NMR (300 MHz, $CDCl_3$, 298 K) δ / ppm: 8.75 (s, 1H, O-**H**), 7.82 (d, J = 8.2 Hz, 2H, Ar-**H**), 7.74 – 7.67 (m, 2H, Ar-**H**), 7.64 – 7.50 (m, 5H, Ar-**H**), 7.36 – 7.23 (m, 2H, Ar-**H**), 7.22 – 7.11 (m, 1H, Ar-**H**), 7.03 (d, J = 8.1 Hz, 2H, Ar-**H**), 6.54 (s, 1H, -C**H**), 2.23 (s, 3H, -C**H**$_3$).

^{13}C-NMR (75 MHz, CDCl3, 298 K) δ / ppm: 154.7, 145.6, 134.6 (d, J = 96.3 Hz), 132.9, 132.3, 130.2, 129.6, 129.5, 129.2, 128.7, 127.5, 125.8 (q, J = 3.8 Hz), 123.7, 124.0 (q, J = 278 Hz), 120.8 (q, J = 29.2 Hz), 110.7 (J = 5.8 Hz), 69.3, 21.6.

^{19}F-NMR (282 MHz, $CDCl_3$, 298 K) δ / ppm = -62.9 (3 F, C**F**$_3$).

HRMS (ESI-TOF): *m/z* calculated for $C_{25}H_{19}F_3O_3S$: 457.1080 $[M+H]^+$, found: 457.1082.

1-(Naphthalen-1-yl(tosyl)methyl)naphthalen-2-ol (7c)

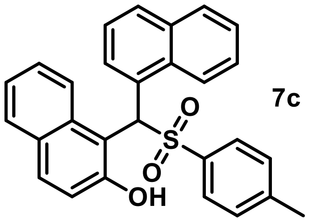

7c

The o-QM precursor **7c** was synthesized following the general procedure *GP4***.** After purification by means of column chromatography the product was obtained as light pink solid in an overall yield of 88%.

^{1}H-NMR (300 MHz, $CDCl_3$, 298 K) δ / ppm: 8.60 (d, J = 7.4 Hz, 1H, O-**H**), 7.91 – 7.75 (m, 3H, Ar-**H**), 7.73 – 7.66 (m, 4H, Ar-**H**), 7.62 – 7.54 (m, 3H, Ar-**H**), 7.39 – 7.34 (m, 1H, Ar-**H**), 7.31 – 7.20 (m, 3H, Ar-**H**), 7.14 (d, J = 8.9 Hz, 1H, Ar-**H**), 7.09 (s, 1H, -C**H**), 7.04 (d, J = 8.0 Hz, 2H, Ar-**H**), 2.26 (s, 3H, -C**H**$_3$).

^{13}C-NMR (75 MHz, CDCl3, 298 K) δ / ppm: 155.1, 145.4, 134.7, 134.1, 133.3, 132.0, 131.9, 129.8, 129.5, 129.4, 129.3, 129.1, 128.4, 127.3, 126.8, 125.6, 125.3, 123.3, 122.8, 121.1, 112.1, 66.3, 21.6.

HRMS (ESI-TOF): *m/z* calculated for $C_{28}H_{22}O_3S$: 461.1182 $[M+Na]^+$, found: 461.1182.

6. Characterization of Products 3

6.1 7,8-Dihydro-6*H*-[1,3]dioxolo[4,5-g]chromanes obtained from oQMs 2

Ethyl (S,Z)-2-(8-(4-methoxyphenyl)-7,8-dihydro-6H-[1,3]dioxolo[4,5-g]chromen-6-ylidene)acetate (3a)

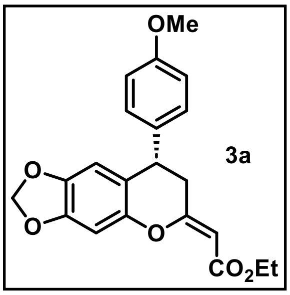

Product **3a** was synthesized on a 0.1 mmol scale following the general procedure *GP5*. After purification, the product was received as a yellow oil in an isolated yield of 59% and an e.r. of >99:1.

^{1}H-NMR (300 MHz, $CDCl_3$, 298 K) δ / ppm: 7.06 – 7.01 (m, 2H, Ar-**H**), 6.87 – 6.82 (m, 2H, Ar-**H**), 6.72 (s, 1H, Ar-**H**), 6.30 (s, 1H, Ar-**H**), 5.90 (s, 2H, -C**H**$_2$), 4.91 (s, 1H, -C=C**H**), 4.17 (qd, *J* = 7.1, 1.3 Hz, 2H, -C**H**$_2$), 3.99 (dd, *J* = 7.6, 5.2 Hz, 1H, -C**H**), 3.79 (s, 3H, -C**H**$_3$), 2.85 (dd, *J* = 14.7, 5.2 Hz, 1H, -C**H**$_2$), 2.70 (dd, *J* = 14.6, 7.6 Hz, 1H, -C**H**$_2$), 1.28 (t, *J* = 7.1 Hz, 3H, -C**H**$_3$).

^{13}C-NMR (75 MHz, $CDCl_3$, 298 K) δ / ppm: 164.9, 161.9, 158.9, 147.4, 146.3, 143.5, 133.8, 129.0, 118.2, 114.3, 107.6, 101.5, 99.2, 96.9, 59.7, 55.4, 39.4, 36.7, 14.5.

HRMS (ESI-TOF): *m/z* calculated for $C_{21}H_{20}O_6$: 369.1333 $[M+H]^+$, found: 369.1331.

$[\alpha]_D^{20}$(c = 1, $CHCl_3$, *e.r.* 99:1): +25.4.

HPLC (YMC-SB, *n*-hexane:IPA = 4:1, flow = 1.0 mL min^{-1}, T_{Column} = 10 °C, λ = 250 nm) t_r: 16.5 min (minor), 22.8 min (major).

Methyl (S,Z)-2-(8-(4-methoxyphenyl)-7,8-dihydro-6H-[1,3]dioxolo[4,5-g]chromen-6-ylidene)acetate (3b)

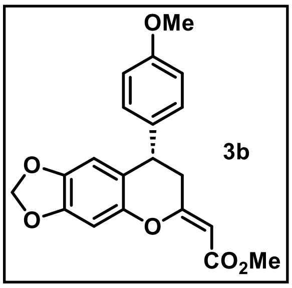

Product **3b** was synthesized on a 0.1 mmol scale following the general procedure *GP5***.** After purification, the product was received as a yellow viscous residue in an isolated yield of 49% and an e.r. of >99:1.

^{1}H-NMR (300 MHz, $CDCl_3$, 298 K) δ / ppm: 7.05 – 7.00 (m, 2H, Ar-**H**), 6.87 – 6.82 (m, 2H, Ar-**H**), 6.74 (s, 1H, Ar-**H**), 6.31 (s, 1H, Ar-**H**), 5.91 (d, *J* = 2.0 Hz, 1H, -C**H**$_2$), 5.90 (d, *J* = 2.0 Hz, 1H, -C**H**$_2$), 4.92 (s, 1H, -C=C**H**), 3.99 (dd, *J* = 7.4, 5.3 Hz, 1H, -C**H**), 3.79 (s, 3H, -C**H3**), 3.70 (s, 3H, -C**H3**), 2.85 (ddd, *J* = 14.7, 5.3, 1.0 Hz, 1H, -C**H**$_2$), 2.70 (ddd, *J* = 14.6, 7.4, 0.9 Hz, 1H, -C**H**$_2$).

^{13}C-NMR (75 MHz, $CDCl_3$, 298 K) δ / ppm: 165.3, 162.1, 158.9, 147.4, 146.2, 143.5, 133.8, 129.0, 118.2, 114.3, 107.6, 101.5, 99.2, 96.5, 55.4, 51.0, 39.4, 36.7.

HRMS (ESI-TOF): *m/z* calculated for $C_{20}H_{18}O_6$: 355.1176 $[M+H]^+$, found: 355.1182.

$[\alpha]_D^{20}$(c = 1, $CHCl_3$, *e.r.* 99:1): +12.6.

HPLC (YMC-SB, *n*-hexane:IPA = 4:1, flow = 1.0 mL min^{-1}, T_{Column} = 10 °C, λ = 272 nm) t_r: 21.2 min (minor), 29.2 min (major).

tert-Butyl (S,Z)-2-(8-(4-methoxyphenyl)-7,8-dihydro-6H-[1,3]dioxolo[4,5-g]chromen-6-ylidene)acetate (3c)

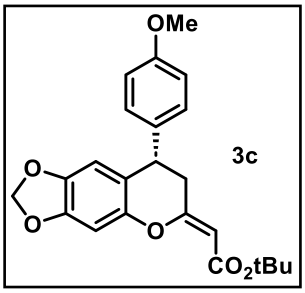

Product **3c** was synthesized on a 0.1 mmol scale following the general procedure *GP5*. After purification, the product was received as a reddish orange oil in an isolated yield of 70% and an e.r. of >99:1.

^{1}H-NMR (300 MHz, $CDCl_3$, 298 K) δ / ppm: 7.06 – 7.01 (m, 2H) , Ar-**H**, 6.87 – 6.83 (m, 2H, Ar-**H**), 6.73 (s, 1H, Ar-**H**), 6.28 (d, J = 0.8 Hz, 1H, Ar-**H**), 5.89 (s, 2H, -C**H₂**), 4.84 (s, 1H, -C=C**H**), 3.97 (dd, J = 7.9, 5.2 Hz, 1H, -C**H**), 3.79 (s, 3H, -C**H₃**), 2.80 (ddd, J = 14.6, 5.2, 1.0 Hz, 1H, -C**H₂**), 2.67 (ddd, J = 14.6, 8.0, 1.0 Hz, 1H, -C**H₂**), 1.50 (s, 9H, -C**H₃**).

^{13}C-NMR (75 MHz, $CDCl_3$, 298 K) δ / ppm: 164.5, 161.0, 158.8, 147.3, 146.4, 143.4, 133.9, 129.1, 118.3, 114.3, 107.6, 101.5, 99.2, 98.6, 79.7, 55.4, 39.5, 36.7, 28.5.

HRMS (ESI-TOF): *m/z* calculated for $C_{23}H_{24}O_6$: 419.1465 [M+Na]$^+$, found: 419.1465.

$[\alpha]_D^{20}$(c = 1, $CHCl_3$, *e.r.* 99:1): +41.3.

HPLC (YMC-SB, *n*-hexane:IPA = 4:1, flow = 1.0 mL min^{-1}, T_{Column} = 10 °C, λ = 250 nm) t_r: 14.2 min (minor), 17.0 min (major).

Benzyl (S,Z)-2-(8-(4-methoxyphenyl)-7,8-dihydro-6H-[1,3]dioxolo[4,5-g]chromen-6-ylidene)acetate (3d)

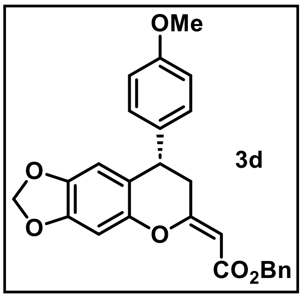

Product **3d** was synthesized on a 0.1 mmol scale following the general procedure *GP5*. After purification, the product was received as an orange viscous residue in an isolated yield of 44% and an e.r. of 99:1.

^{1}H-NMR (300 MHz, $CDCl_3$, 298 K) δ / ppm: 7.42 – 7.31 (m, 5H, Ar-**H**), 7.06 – 7.01 (m, 2H, Ar-**H**), 6.87 – 6.82 (m, 2H, Ar-**H**), 6.71 (s, 1H, Ar-**H**), 6.31 (s, 1H, Ar-**H**), 5.91 – 5.90 (m, 2H, -C**H₂**), 5.18 (dd, J = 15.3, 12.7 Hz, 2H, -C**H₂**), 4.98 (s, 1H, -C=C**H**), 3.99 (dd, J = 7.6, 5.2 Hz, 1H, -C**H**), 3.79 (s, 3H, -C**H₃**), 2.85 (ddd, J = 14.7, 5.3, 1.0 Hz, 1H, -C**H₂**), 2.71 (dd, J = 14.6, 7.6 Hz, 1H, -C**H₂**).

^{13}C-NMR (75 MHz, $CDCl_3$, 298 K) δ / ppm: 164.7, 162.5, 158.9, 147.4, 146.2, 143.5, 136.7, 133.7, 129.0, 128.6, 128.2, 128.1, 118.2, 114.4, 107.6, 101.5, 99.1, 96.5, 65.5, 55.4, 39.4, 36.7.

HRMS (ESI-TOF): *m/z* calculated for $C_{26}H_{22}O_6$: 453.1309 [M+Na]$^+$, found: 453.1305.

$[\alpha]_D^{20}$(c = 1, $CHCl_3$, *e.r.* 99:1): +10.5.

HPLC (YMC-SA, *n*-hexane:IPA = 4:1, flow = 1.0 mL min^{-1}, T_{Column} = 10 °C, *l* = 205 nm) t_r: 18.6 min (minor), 22.5 min (major).

Methyl (S,Z)-2-(8-phenyl-7,8-dihydro-6H-[1,3]dioxolo[4,5-g]chromen-6-ylidene)acetate (3e)

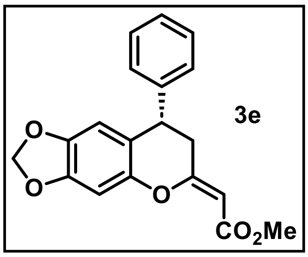

Product **3e** was synthesized on a 0.1 mmol scale following the general procedure *GP5*. After purification, the product was received as a yellow viscous residue in an isolated yield of 41% and an e.r. of >99:1

^{1}H-NMR (300 MHz, $CDCl_3$, 298 K) *δ* / ppm: 7.33 – 7.24 (m, 3H, Ar-**H**), 7.10 (d, J = 6.5 Hz, 2H, Ar-**H**), 6.74 (s, 1H, Ar-**H**), 6.30 (s, 1H, Ar-**H**), 5.89 (s, 2H, -C**H₂**), 4.91 (s, 1H, -C=C**H**), 4.03 (dd, J = 7.3, 5.4 Hz, 1H, -C**H**), 3.69 (s, 3H, -C**H₃**), 2.87 (dd, J = 14.7, 5.2 Hz, 1H, -C**H₂**), 2.72 (dd, J = 14.7, 7.3 Hz, 1H, -C**H₂**).

^{13}C-NMR (75 MHz, $CDCl_3$, 298 K) *δ* / ppm: 165.3, 161.9, 147.5, 146.3, 143.6, 141.8, 129.0, 128.0, 127.4, 117.7, 107.7, 101.6, 99.2, 96.5, 51.0, 40.2, 36.5.

HRMS (ESI-TOF): *m/z* calculated for $C_{19}H_{16}O_5$: 325.1071 $[M+H]^+$, found: 325.1072.

$[\alpha]_D^{20}$(c = 1, $CHCl_3$, *e.r.* 99:1): -2.0.

HPLC (YMC-SA, *n*-hexane:IPA = 4:1, flow = 1.0 mL min^{-1}, T_{Column} = 10 °C, *λ* = 250 nm) t_r: 10.3 min (minor), 11.9 min (major).

***tert*-Butyl (S,Z)-2-(8-phenyl-7,8-dihydro-6H-[1,3]dioxolo[4,5-g]chromen-6-ylidene)acetate (3f)**

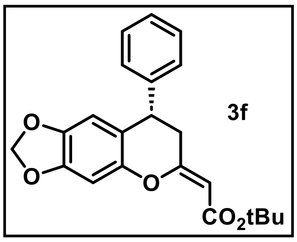

Product **3f** was synthesized on a 0.1 mmol scale following the general procedure *GP5*. After purification, the product was received as a yellow viscous residue in an isolated yield of 60% and an e.r. of >99:1.

^{1}H-NMR (300 MHz, $CDCl_3$, 298 K) *δ* / ppm: 7.35 – 7.23 (m, 3H, Ar-**H**), 7.14 – 7.10 (m, 2H, Ar-**H**), 6.74 (s, 1H, Ar-**H**), 6.28 (s, 1H, Ar-**H**), 5.90 (s, 2H, -C**H₂**), 4.84 (s, 1H, -C=C**H**), 4.02 (dd, *J* = 7.9, 5.3 Hz, 1H, -C**H**), 2.84 (dd, *J* = 14.7, 5.3 Hz, 1H, -C**H₂**), 2.71 (dd, *J* = 14.6, 7.9 Hz, 1H, -C**H₂**), 1.50 (s, 9H, -C**H₃**).

^{13}C-NMR (75 MHz, $CDCl_3$, 298 K) *δ* / ppm: 164.4, 160.8, 147.4, 146.5, 143.4, 141.9, 129.0, 128.1, 127.4, 117.9, 107.7, 101.5, 99.2, 98.7, 79.7, 40.3, 36.5, 28.5.

HRMS (ESI-TOF): *m/z* calculated for $C_{22}H_{22}O_5$: 389.1359 $[M+Na]^+$, found: 389.1358.

$[\alpha]_D^{20}$(c = 1, $CHCl_3$, *e.r.* 99:1): +25.5.

HPLC (YMC-SA, *n*-hexane:IPA = 10:1, flow = 0.5 mL min^{-1}, T_{Column} = 10 °C, *λ* = 250 nm) t_r: 14.7 min (major), 16.7 min (minor).

tert-Butyl (S,Z)-2-(8-(p-tolyl)-7,8-dihydro-6H-[1,3]dioxolo[4,5-g]chromen-6-ylidene)acetate (3g)

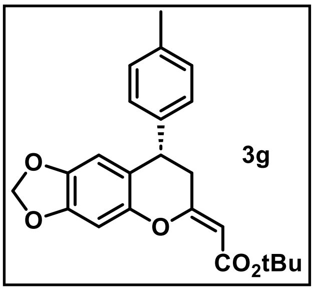

Product **3g** was synthesized on a 0.1 mmol scale following the general procedure *GP5*. After purification, the product was received as an orange oil in an isolated yield of 62% and an e.r. of >99:1

^{1}H-NMR (300 MHz, $CDCl_3$, 298 K) δ / ppm: 7.13 (d, *J* = 7.9 Hz, 2H, Ar-**H**), 7.01 (d, *J* = 8.1 Hz, 2H, Ar-**H**), 6.73 (s, 1H, Ar-**H**), 6.27 (d, *J* = 0.5 Hz, 1H, Ar-**H**), 5.89 (d, *J* = 1.8 Hz, 1H, -C**H$_2$**), 5.89 (d, *J* = 1.8 Hz, 1H, -C**H$_2$**), 4.85 (s, 1H, -C=C**H**), 3.98 (dd, *J* = 8.0, 5.2 Hz, 1H, -C**H**), 2.81 (ddd, *J* = 14.6, 5.3, 1.0 Hz, 1H, -C**H$_2$**), 2.69 (ddd, *J* = 14.6, 8.0, 1.0 Hz, 1H, -C**H$_2$**), 2.33 (s, 3H, -C**H$_3$**), 1.50 (s, 9H, -C**H$_3$**).

^{13}C-NMR (125 MHz, $CDCl_3$, 298 K) δ / ppm: 164.5, 161.0, 147.3, 146.4, 143.3, 138.9, 137.0, 129.6, 127.9, 118.2, 107.6, 101.5, 99.1, 98.5, 79.7, 39.9, 36.6, 28.5, 21.2.

HRMS (ESI-TOF): *m/z* calculated for $C_{23}H_{24}O_5$: 381.1697 $[M+H]^+$, found: 381.1696.

$[\alpha]_D^{20}$(c = 1, $CHCl_3$, *e.r.* 99:1): +41.7.

HPLC (CHIRALCEL® OD-H, *n*-hexane:IPA = 10:1, flow = 0.5 mL min^{-1}, T_{Column} = 10 °C, λ = 220 nm) t_r: 49.5 min (minor), 57.4 min (major).

tert-Butyl (S,Z)-2-(8-(4-(dimethylamino)phenyl)-7,8-dihydro-6H-[1,3]dioxolo[4,5-g]chromen-6-ylidene)acetate (3h)

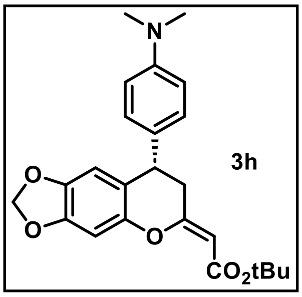

Product **3h** was synthesized on a 0.1 mmol scale following the general procedure *GP5*. After purification, the product was received as an orange viscous residue in an isolated yield of 74% and an e.r. of >99:1

^{1}H-NMR (300 MHz, $CDCl_3$, 298 K) δ / ppm: 7.02 – 6.97 (m, 2H, Ar-**H**), 6.72 (s, 1H, Ar-**H**), 6.71 – 6.67 (m, 2H, Ar-**H**), 6.30 (s, 1H, Ar-**H**), 5.88 (s, 2H, -C**H$_2$**), 4.85 (s, 1H, -C=C**H**), 3.92 (dd, *J* = 8.3, 5.2 Hz, 1H, -C**H**), 2.94 (s, 6H, -C**H$_3$**), 2.78 (ddd, *J* = 14.5, 5.3, 0.9 Hz, 1H, -C**H$_2$**), 2.68 (ddd, *J* = 14.6, 8.4, 1.0 Hz, 1H, -C**H$_2$**), 1.50 (s, 9H, -C**H$_3$**).

^{13}C-NMR (75 MHz, $CDCl_3$, 298 K) δ / ppm: 164.6, 161.6, 149.8, 147.2, 146.3, 143.3, 129.5, 128.7, 119.0, 113.0, 107.7, 101.4, 99.1, 98.3, 79.6, 40.8, 39.4, 36.7, 28.5.

HRMS (ESI-TOF): *m/z* calculated for $C_{24}H_{27}NO_5$: 410.1962 $[M+H]^+$, found: 410.1963.

$[\alpha]_D^{20}$(c = 1, $CHCl_3$, *e.r.* 99:1): +38.9.

HPLC (YMC-SB, *n*-hexane:IPA = 4:1, flow = 1.0 mL min^{-1}, T_{Column} = 10 °C, λ = 220 nm) t_r: 12.7 min (minor), 18.0 min (major).

tert-Butyl (S,Z)-2-(8-(4-nitrophenyl)-7,8-dihydro-6H-[1,3]dioxolo[4,5-g]chromen-6-ylidene)acetate (3i)

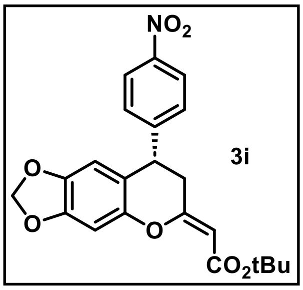

3i

Product **3i** was synthesized on a 0.1 mmol scale following the general procedure *GP5*. After purification, the product was received as an orange oil in an isolated yield of 73% and an e.r. of 99:1.

^{1}H-NMR (300 MHz, $CDCl_3$, 298 K) δ / ppm: 8.19 – 8.14 (m, 2H, Ar-**H**), 7.30 – 7.25 (m, 2H, Ar-**H**), 6.77 (s, 1H, Ar-**H**), 6.29 (s, 1H, Ar-**H**), 5.93 (d, J = 1.7 Hz, 1H, -C**H₂**), 5.93 (d, J = 1.7 Hz, 1H, -C**H₂**), 4.81 (s, 1H, -C=C**H**), 4.17 (t, J = 5.7 Hz, 1H, -C**H**), 2.94 (ddd, J = 14.7, 5.6, 1.3 Hz, 1H, -C**H₂**), 2.65 (dd, J = 14.8, 6.0 Hz, 1H, -C**H₂**), 1.48 (s, 9H, -C**H₃**).

^{13}C-NMR (75 MHz, $CDCl_3$, 298 K) δ / ppm: 164.0, 159.1, 149.7, 148.1, 147.3, 146.5, 143.7, 128.9, 124.2, 115.5, 107.4, 101.7, 99.6, 99.5, 80.0, 40.2, 36.1, 28.4.

HRMS (ESI-TOF): *m/z* calculated for $C_{22}H_{21}NO_7$: 434.1210 $[M+Na]^+$, found: 434.1205.

$[\alpha]_D^{20}$(c = 1, $CHCl_3$, *e.r.* 99:1): +0.4.

HPLC (YMC-SB, *n*-hexane:IPA = 6:1, flow = 1.0 mL min^{-1}, T_{Column} = 10 °C, λ = 272 nm) t_r: 38.8 min (minor), 44.0 min (major).

tert-Butyl (S,Z)-2-(8-(4-(trifluoromethyl)phenyl)-7,8-dihydro-6H-[1,3]dioxolo[4,5-g]chromen-6-ylidene)acetate (3j)

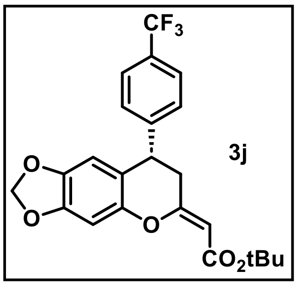

3j

Product **3j** was synthesized on a 0.1 mmol scale following the general procedure *GP5*. After purification, the product was received as a yellow viscous residue in an isolated yield of 55% and an e.r. of >99:1.

^{1}H-NMR (300 MHz, $CDCl_3$, 298 K) δ / ppm: 7.57 (d, J = 8.1 Hz, 2H, Ar-**H**), 7.24 (d, J = 8.0 Hz, 2H, Ar-**H**), 6.76 (s, 1H, Ar-**H**), 6.27 (s, 1H, Ar-**H**), 5.92 (d, J = 2.6 Hz, 1H, -C**H₂**), 5.91 (d, J = 2.6 Hz, 1H, -C**H₂**), 4.83 (s, 1H, -C=C**H**), 4.10 (t, J = 6.1 Hz, 1H, -C**H**), 2.89 (ddd, J = 14.6, 5.5, 1.2 Hz, 1H, -C**H₂**), 2.67 (dd, J = 14.9, 7.0 Hz, 1H, -C**H₂**), 1.49 (s, 9H, -C**H₃**).

^{13}C-NMR (75 MHz, $CDCl_3$, 298 K) δ / ppm: 164.2, 159.8, 147.8, 146.5, 146.2 (d, J = 1.4 Hz), 143.6, 129.7 (q, J = 32.5 Hz), 128.4, 125.9 (q, J = 3.7 Hz), 124.1 (q, J = 272 Hz), 116.5, 107.5, 101.6, 99.4, 99.3, 79.9, 40.2, 36.3, 28.5.

^{19}F-NMR (282 MHz, $CDCl_3$, 298 K) δ / ppm = -62.5 (3 F, C**F₃**).

HRMS (ESI-TOF): *m/z* calculated for $C_{23}H_{21}F_3O_5$: 457.1233 $[M+Na]^+$, found: 457.1233.

$[\alpha]_D^{20}$(c = 1, $CHCl_3$, *e.r.* 99:1): +18.9.

HPLC (YMC-SZ, *n*-hexane:IPA = 15:1, flow = 1.0 mL min^{-1}, T_{Column} = 10 °C, λ = 272 nm) t_r: 10.6 min (major), 14.4 min (minor).

***tert*-Butyl (S,Z)-2-(8-(4-chlorophenyl)-7,8-dihydro-6H-[1,3]dioxolo[4,5-g]chromen-6-ylidene)acetate (3k)**

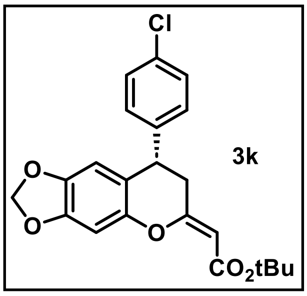

Product **3k** was synthesized on a 0.1 mmol scale following the general procedure *GP5*. After purification, the product was received as a yellow viscous residue in an isolated yield of 59% and an e.r. of >99:1.

^{1}H-NMR (300 MHz, $CDCl_3$, 298 K) δ / ppm: 7.31 – 7.27 (m, 2H, Ar-**H**), 7.07 – 7.02 (m, 2H, Ar-**H**), 6.74 (s, 1H, Ar-**H**), 6.27 (s, 1H, Ar-**H**), 5.91 (s, 2H, -C**H₂**), 4.83 (s, 1H, -C=C**H**), 4.01 (dd, *J* = 7.2, 5.3 Hz, 1H, -C**H**), 2.84 (ddd, *J* = 14.6, 5.3, 1.1 Hz, 1H, -C**H₂**), 2.64 (ddd, *J* = 14.6, 7.2, 0.9 Hz, 1H, -C**H₂**), 1.49 (s, 9H, -C**H₃**).

^{13}C-NMR (175 MHz, $CDCl_3$, 298 K) δ / ppm: 164.3, 160.2, 147.7, 146.5, 143.5, 140.6, 133.2, 129.4, 129.1, 117.1, 107.5, 101.6, 99.3, 99.0, 79.8, 39.8, 36.4, 28.5.

HRMS (ESI-TOF): *m/z* calculated for $C_{22}H_{21}ClO_5$: 423.0970 $[M+Na]^+$, found: 423.0969.

$[\alpha]_D^{20}$(c = 1, $CHCl_3$, *e.r.* 99:1): +21.3.

HPLC (YMC-SZ, *n*-hexane:IPA = 4:1, flow = 1.0 mL min^{-1}, T_{Column} = 10 °C, λ = 250 nm) t_r: 8.8 min (major), 11.4 min (minor).

***tert*-Butyl (S,Z)-2-(8-(3-fluorophenyl)-7,8-dihydro-6H-[1,3]dioxolo[4,5-g]chromen-6-ylidene)acetate (3l)**

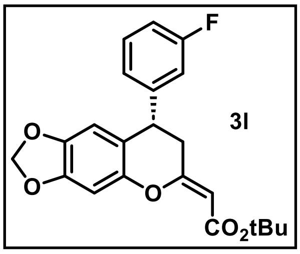

Product **3l** was synthesized on a 0.1 mmol scale following the general procedure *GP5*. After purification, the product was received as a reddish orange oil in an isolated yield of 59% and an e.r. of >99:1.

^{1}H-NMR (300 MHz, $CDCl_3$, 298 K) δ / ppm: 7.28 (td, *J* = 8.0, 6.0 Hz, 1H, Ar-**H**), 6.99 – 6.90 (m, 2H, Ar-**H**), 6.81 (dt, *J* = 9.8, 2.2 Hz, 1H, Ar-**H**), 6.74 (s, 1H, Ar-**H**), 6.29 (d, *J* = 0.5 Hz, 1H, Ar-**H**), 5.92 (s, 2H, -C**H₂**), 4.84 (s, 1H, -C=C**H**), 4.03 (dd, *J* = 7.3, 5.3 Hz, 1H, -C**H**), 2.85 (ddd, *J* = 14.7, 5.4, 1.1 Hz, 1H, -C**H₂**), 2.67 (ddd, *J* = 14.6, 7.3, 0.9 Hz, 1H, -C**H₂**), 1.49 (s, 9H, -C**H₃**).

^{13}C-NMR (75 MHz, $CDCl_3$, 298 K) δ / ppm: 164.3, 163.2 (d, *J* = 246.7 Hz), 160.2, 147.7, 146.5, 144.6 (d, *J* = 6.9 Hz), 143.5, 130.5 (d, *J* = 8.3 Hz), 123.7 (d, *J* = 2.9 Hz), 116.9, 115.0 (d, *J* = 21.7 Hz), 114.4 (d, *J* = 21.1 Hz), 107.6, 101.6, 99.3, 99.0, 79.8, 40.1 (d, *J* = 1.8 Hz), 36.3, 28.5.

^{19}F-NMR (282 MHz, $CDCl_3$, 298 K) δ / ppm: -112.4 (1 F, Ar-**F**).

HRMS (ESI-TOF): *m/z* calculated for $C_{22}H_{21}FO_5$: 407.1265 $[M+Na]^+$, found: 407.1269.

$[\alpha]_D^{20}$(c = 1, $CHCl_3$, *e.r.* 99:1): +19.3.

HPLC (YMC-SB, *n*-hexane:IPA = 4:1, flow = 1.0 mL min^{-1}, T_{Column} = 10 °C, λ = 272 nm) t_r: 10.5 min (major), 12.4 min (minor).

tert-Butyl (S,Z)-2-(8-(benzo[d][1,3]dioxol-5-yl)-7,8-dihydro-6H-[1,3]dioxolo[4,5-g]chromen-6-ylidene)acetate (3m)

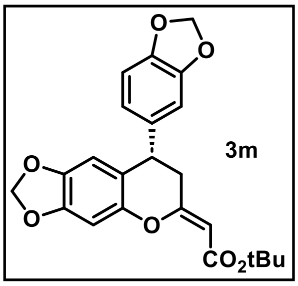

Product **3m** was synthesized on a 0.1 mmol scale following the general procedure *GP5*. After purification, the product was received as a white viscous residue in an isolated yield of 67% and an e.r. of >99:1.

^{1}H-NMR (300 MHz, $CDCl_3$, 298 K) δ / ppm: 6.76 – 6.72 (m, 2H, Ar-**H**), 6.60 – 6.57 (m, 2H, Ar-**H**), 6.30 (s, 1H, Ar-**H**), 5.94 (s, 2H, -C**H₂**), 5.90 (s, 2H, -C**H₂**), 4.85 (s, 1H, -C=C**H**), 3.95 (dd, *J* = 7.8, 5.2 Hz, 1H, -C**H**), 2.80 (dd, *J* = 14.7, 5.2 Hz, 1H, -C**H₂**), 2.64 (dd, *J* = 14.6, 7.8 Hz, 1H, -C**H₂**), 1.50 (s, 9H, -C**H₃**).

^{13}C-NMR (75 MHz, $CDCl_3$, 298 K) δ / ppm: 164.4, 160.8, 148.2, 147.5, 146.9, 146.4, 143.4, 135.8, 121.4, 117.9, 108.5, 108.2, 107.6, 101.5, 101.2, 99.2, 98.7, 79.8, 40.0, 36.7, 28.5.

HRMS (ESI-TOF): *m/z* calculated for $C_{23}H_{22}O_7$: 433.1258 $[M+Na]^+$, found: 433.1262.

$[\alpha]_D^{20}$(c = 1, $CHCl_3$, *e.r.* 99:1): +28.4.

HPLC (CHIRALPAK® AD-H, *n*-hexane:IPA = 4:1, flow = 1.0 mL min^{-1}, T_{Column} = 10 °C, λ = 272 nm) t_r: 15.3 min (major), 16.8 min (minor).

tert-Butyl (R,Z)-2-(8-(1-methyl-1H-indol-2-yl)-7,8-dihydro-6H-[1,3]dioxolo[4,5-g]chromen-6-ylidene)acetate (3n)

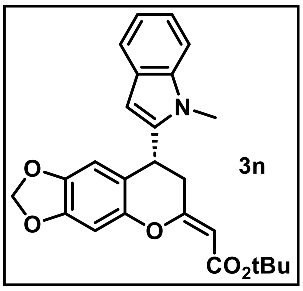

Product **3n** was synthesized on a 0.1 mmol scale following the general procedure *GP5*. After purification, the product was received as a white amorphous residue in an isolated yield of 79% and an e.r. of 99:1.

^{1}H-NMR (300 MHz, $CDCl_3$, 298 K) δ / ppm: 7.54 (d, *J* = 7.8 Hz, 1H, Ar-**H**), 7.34 (d, *J* = 8.1 Hz, 1H, Ar-**H**), 7.26 – 7.20 (m, 1H, Ar-**H**), 7.11 (ddd, *J* = 8.0, 7.0, 1.1 Hz, 1H, Ar-**H**), 6.77 (s, 1H, Ar-**H**), 6.27 (s, 1H, Ar-**H**), 6.19 (s, 1H, -C=C**H**), 5.91 (d, *J* = 3.9 Hz, 1H, -C**H₂**), 5.90 (d, *J* = 3.9 Hz, 1H, -C**H₂**), 4.91 (s, 1H, -C=C**H**), 4.28 (t, *J* = 6.9 Hz, 1H, -C**H**), 3.73 (s, 3H, -C**H₃**), 2.88 (d, *J* = 6.9 Hz, 2H, -C**H₂**), 1.51 (s, 9H, -C**H₃**).

^{13}C-NMR (75 MHz, $CDCl_3$, 298 K) δ / ppm: 164.3, 160.5, 147.7, 146.2, 143.6, 139.3, 137.8, 127.7, 121.7, 120.6, 119.9, 116.9, 109.2, 106.8, 101.6, 100.8, 99.4, 98.9, 79.8, 34.6, 32.6, 30.1, 28.5.

HRMS (ESI-TOF): *m/z* calculated for $C_{25}H_{25}NO_5$: 442.1629 $[M+Na]^+$, found: 442.1625.

$[\alpha]_D^{20}$(c = 1, $CHCl_3$, *e.r.* 99:1): +71.6.

HPLC (YMC-SB, *n*-hexane:IPA = 4:1, flow = 1.0 mL min^{-1}, T_{Column} = 10 °C, λ = 272 nm) t_r: 16.3 min (minor), 29.7 min (major).

tert-Butyl (R,Z)-2-(8-(furan-2-yl)-7,8-dihydro-6H-[1,3]dioxolo[4,5-g]chromen-6-ylidene)acetate (3o)

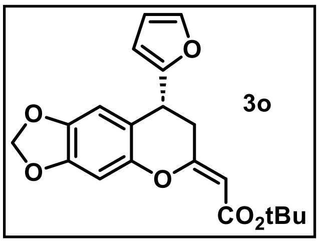

Product **3o** was synthesized on a 0.1 mmol scale following the general procedure *GP5*. After purification, the product was received as an orange oil in an isolated yield of 89% and an e.r. of >99:1.

^{1}H-NMR (300 MHz, $CDCl_3$, 298 K) δ / ppm: 7.35 (dd, J = 1.9, 0.8 Hz, 1H, -C=C**H**), 6.70 (s, 1H, Ar-**H**), 6.45 (s, 1H, Ar-**H**), 6.29 (dd, J = 3.2, 1.9 Hz, 1H, -C=C**H**), 6.02 (dt, J = 3.2, 0.8 Hz, 1H, -C=C**H**), 5.92 (d, J = 3.5 Hz, 1H, -C**H$_2$**), 5.92 (d, J = 3.5 Hz, 1H, -C**H$_2$**), 4.92 (s, 1H, -C=C**H**), 4.11 (t, J = 6.0 Hz, 1H, -C**H**), 2.90 (ddd, J = 14.8, 6.7, 0.8 Hz, 1H, -C**H$_2$**), 2.79 (ddd, J = 14.8, 5.3, 1.2 Hz, 1H, -C**H$_2$**), 1.49 (s, 9H, -C**H$_3$**).

^{13}C-NMR (75 MHz, $CDCl_3$, 298 K) δ / ppm: 164.4, 160.4, 154.1, 147.7, 146.2, 143.4, 142.3, 115.3, 110.4, 107.4, 107.3, 101.6, 99.4, 98.8, 79.8, 33.9, 33.1, 28.5.

HRMS (ESI-TOF): *m/z* calculated for $C_{20}H_{20}O_6$: 379.1152 $[M+Na]^+$, found: 379.1153.

$[\alpha]_D^{20}$(c = 1, $CHCl_3$, *e.r.* 99:1): +3.4.

HPLC (YMC-SB, *n*-hexane:IPA = 4:1, flow = 1.0 mL min^{-1}, T_{Column} = 10 °C, λ = 250 nm) t_r: 7.3 min (minor), 11.8 min (major).

tert-Butyl (R,Z)-(8-(1-methyl-1*H*-pyrrol-2-yl)-7,8-dihydro-6*H*-[1,3]dioxolo[4,5-*g*]chromen-6-ylidene)acetate (3p)

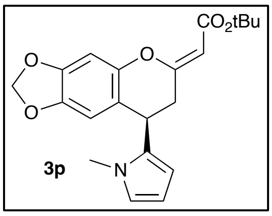

Product **3p** was synthesized on a 0.05 mmol scale following the general procedure *GP5*. After purification, the product was received as an orange oil in an isolated yield of 86% and an e.r. of 99:1.

^{1}H-NMR (300 MHz, $CDCl_3$, 298 K) δ / ppm: 6.71 (s, 1 H, Ar-**H**), 6.62 (dd, J_1 = 2.7 Hz, J_2 = 1.8 Hz, 1 H, Ar-**H**), 6.18 (d, J = 0.8 Hz, 1 H, Ar-**H**), 6.10 (dd, J_1 = 3.4 Hz, J_2 = 2.7 Hz, 1 H, Ar-**H**), 5.90 (d, J = 1.4 Hz, 1 H, O-C**H$_2$**-O-), 5.89 (d, J = 1.4 Hz, 1 H, O-C**H$_2$**-O-), 5.86 (dd, J_1 = 3.4 Hz, J_2 = 1.8 Hz, 1 H, Ar-**H**), 4.92 (s, 1 H, -C=C-**H**), 4.07 (dd, J_1 = 8.8 Hz, J_2 = 6.2 Hz, 1 H, -C**H**-CH$_2$-), 3.58 (s, 3 H, N-C**H$_3$**), 2.79-2.76 (m, 1 H, -CH-C**H$_2$**-), 1.51 (s, 9 H, -(C**H$_3$**)$_3$).

^{13}C-NMR (75 MHz, $CDCl_3$, 298 K) δ / ppm: 164.5, 161.5, 147.4, 146.1, 143.5, 131.7, 122.6, 118.5, 107.6, 107.1, 106.6, 101.5, 99.3, 98.3, 79.8, 34.9, 34.1, 32.0, 28.5 (3 C).

HRMS (ESI-TOF): *m/z* calculated for $C_{21}H_{23}NNaO_5^+$: 392.1468, found 392.1467.

$[\alpha]_D^{20}$(c = 1, $CHCl_3$, *e.r.* 99:1): +51.5.

HPLC (YMC-SB, *n*-hexane:IPA = 4:1, flow = 1.0 mL min^{-1}, T_{Column} = 10 °C, λ = 250 nm) t_r: 18.28 min (minor), 23.84 min (major).

Diethyl (S,Z)-2-(8-(4-methoxyphenyl)-7,8-dihydro-6H-[1,3]dioxolo[4,5-g]chromen-6-ylidene)succinate (3q)

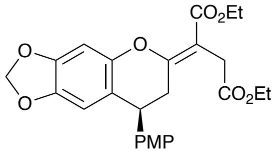

3q

Product **3q** was synthesized on a 0.1 mmol scale following the general procedure *GP5*. After purification, the product was received as a yellow oil in an isolated yield of 49% (74% with **ISeU**) and an e.r. of >99:1.

^{1}H-NMR (300 MHz, $CDCl_3$, 298 K) δ / ppm: 7.09 – 7.04 (m, 2H, Ar-**H**), 6.88 – 6.83 (m, 2H, Ar-**H**), 6.62 (s, 1H, Ar-**H**), 6.26 (d, J = 0.8 Hz, 1H, Ar-**H**), 5.89 (s, 2H, -C**H₂**), 4.25 (q, J = 7.1 Hz, 2H, -C**H₂**), 4.07 (dd, J = 7.1, 2.3 Hz, 2H, -C**H₂**), 4.11 – 3.98 (m, 1H, -C**H**), 3.79 (s, 3H, -C**H₃**), 3.22 (q, J = 17.2 Hz, 2H, -C**H₂**), 2.91 (dd, J = 14.9, 5.2 Hz, 1H, -C**H₂**), 2.79 (dd, J = 14.9, 8.5 Hz, 1H, -C**H₂**), 1.33 (t, J = 7.1 Hz, 3H, -C**H₃**), 1.20 (t, J = 7.1 Hz, 3H, -C**H₃**).

^{13}C-NMR (125 MHz, $CDCl_3$, 298 K) δ / ppm: 171.4 (2x), 166.6, 158.8, 147.3, 146.6, 143.1, 133.9, 129.1, 118.1, 114.3, 107.5, 102.8, 101.4, 98.8, 61.0, 60.5, 55.4, 39.4, 34.0, 33.1, 14.5, 14.3.

HRMS (ESI-TOF): *m/z* calculated for $C_{25}H_{26}O_8$: 477.1520 $[M+Na]^+$, found: 477.1519.

$[\alpha]_D^{20}$(c = 1, $CHCl_3$, *e.r.* 99:1): +84.2.

HPLC (YMC-SB, *n*-hexane:IPA = 4:1, flow = 1.0 mL min^{-1}, T_{Column} = 10 °C, λ = 250 nm) t_r: 14.3 min (minor), 18.4 min (major).

4-(*tert*-Butyl) 1-ethyl (S,Z)-2-(8-(4-methoxyphenyl)-7,8-dihydro-6H-[1,3]dioxolo[4,5-g]chromen-6-ylidene)succinate (3r)

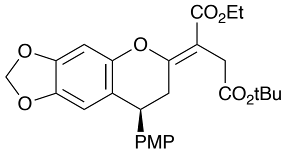

3r

Product **3r** was synthesized on a 0.1 mmol scale following the general procedure *GP5*. After purification, the product was received as a yellow oil in an isolated yield of 49% (64% with **ISeU**) and an e.r. of 99:1.

^{1}H-NMR (300 MHz, $CDCl_3$, 298 K) δ / ppm: 7.09 – 7.04 (m, 2H, Ar-**H**), 6.88 – 6.83 (m, 2H, Ar-**H**), 6.61 (s, 1H, Ar-**H**), 6.25 (d, J = 0.8 Hz, 1H, Ar-**H**), 5.88 (s, 2H, -C**H₂**), 4.24 (q, J = 7.1 Hz, 2H, -C**H₂**), 4.00 (dd, J = 8.7, 5.2 Hz, 1H, -C**H**), 3.79 (s, 3H, -C**H₃**), 3.13 (dd, J = 37.0, 17.6 Hz, 2H, -C**H₂**), 2.90 (dd, J = 14.9, 5.1 Hz, 1H, -C**H₂**), 2.78 (dd, J = 14.8, 8.7 Hz, 1H, -C**H₂**), 1.39 (s, 9H, -C**H₃**), 1.33 (t, J = 7.1 Hz, 3H, -C**H₃**).

^{13}C-NMR (75 MHz, $CDCl_3$, 298 K) δ / ppm: 170.6, 166.7, 158.9, 158.2, 147.3, 146.7, 143.1, 134.1, 129.1, 118.2, 114.3, 107.5, 103.5, 101.4, 98.8, 80.9, 60.4, 55.4, 39.5, 35.1, 33.0, 28.1, 14.5.

HRMS (ESI-TOF): *m/z* calculated for $C_{27}H_{30}O_8$: 505.1833 $[M+Na]^+$, found: 505.1830.

$[\alpha]_D^{20}$(c = 1, $CHCl_3$, *e.r.* 99:1): +68.2.

HPLC (YMC-SB, *n*-hexane:IPA = 6:1, flow = 1.0 mL min^{-1}, T_{Column} = 10 °C, λ = 250 nm) t_r: 10.4 min (minor), 13.3 min (major).

Ethyl (S)-6-ethyl-8-(4-methoxyphenyl)-8H-[1,3]dioxolo[4,5-g]chromene-7-carboxylate (6s)

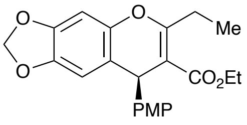

6s

Product **6s** was synthesized on a 0.1 mmol scale following the general procedure *GP5*. After purification, the product was received as a white viscous residue in an isolated yield of 17% and an e.r. of 59:41.

^{1}H-NMR (500 MHz, $CDCl_3$, 298 K) δ / ppm: 7.13 – 7.10 (m, 2H, Ar-**H**), 6.79 – 6.76 (m, 2H, Ar-**H**), 6.54 (s, 1H, Ar-**H**), 6.43 (d, J = 0.6 Hz, 1H, Ar-**H**), 5.89 (d, J = 1.4 Hz, 1H, -C**H$_2$**), 5.84 (d, J = 1.4 Hz, 1H, -C**H$_2$**), 4.84 (s, 1H, -C**H**), 4.13 – 4.03 (m, 2H, -C**H$_2$**), 3.75 (s, 3H, -C**H$_3$**), 2.92 – 2.78 (m, 2H, -C**H$_2$**), 1.25 (t, J = 7.5 Hz, 3H, -C**H$_3$**), 1.19 (t, J = 7.1 Hz, 3H, -C**H$_3$**).

^{13}C-NMR (125 MHz, $CDCl_3$, 298 K) δ / ppm 167.2, 164.5, 158.3, 146.8, 144.4, 144.2, 139.4, 128.8, 117.4, 113.9, 107.6, 105.1, 101.4, 98.0, 60.2, 55.3, 41.1, 26.1, 14.3, 12.1.

HRMS (ESI-TOF): *m/z* calculated for $C_{22}H_{22}O_6$: 383.1489 $[M+H]^+$, found: 383.1489.

$[\alpha]_D^{20}$(c = 1, $CHCl_3$, *e.r.* 59:41): +7.7.

HPLC (YMC-SZ, *n*-hexane:IPA = 100:1, flow = 1.0 mL min^{-1}, T_{Column} = 10 °C, λ = 272 nm) t_r: 10.7 min (major), 11.9 min (minor).

6.2 1,2-Dihydro-3*H*-benzo[f]chromanes from QM-precursors 7

Ethyl (S,Z)-2-(1-phenyl-1,2-dihydro-3H-benzo[f]chromen-3-ylidene)acetate (3aa)

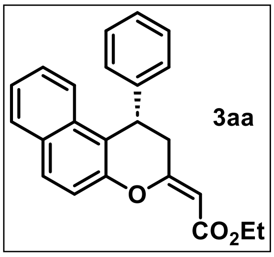

3aa

Product **3aa** was synthesized analogously to the general procedure *GP6* on a scale of 0.1 mmol. In order to remove the side product (arising from the addition of the sulfinate leaving group to the allenoate) the resulting mixture was additionally purified *via* semi-preparative column chromatography, to obtain the final product as a white amorphous residue in an isolated yield of 77% and an e.r. of 99:1.

^{1}H-NMR (300 MHz, $CDCl_3$, 298 K) δ / ppm: 7.84 – 7.80 (m, 2H, Ar-**H**), 7.74 – 7.71 (m, 1H, Ar-**H**), 7.45 (d, J = 9.0 Hz, 1H, Ar-**H**), 7.43 – 7.33 (m, 2H, Ar-**H**), 7.28 – 7.15 (m, 3H, Ar-**H**), 7.13 – 7.09 (m, 2H, Ar-**H**), 4.90 (d, J = 1.8 Hz, 1H, -C=C**H**), 4.80 (dd, J = 6.5, 1.9 Hz, 1H, -C**H**), 4.17 (qd, J = 7.1, 1.7 Hz, 2H, -C**H₂**), 3.17 (ddd, J = 14.6, 6.4, 1.8 Hz, 1H, -C**H₂**), 2.77 (dd, J = 14.6, 1.9 Hz, 1H, -C**H₂**), 1.28 (t, J = 7.2 Hz, 3H, -C**H₃**).

^{13}C-NMR (75 MHz, $CDCl_3$, 298 K) δ / ppm: 164.8, 160.4, 149.6, 142.1, 131.7, 130.6, 129.6, 128.9, 128.8, 127.7, 127.2, 124.6, 122.8, 118.3, 116.7, 98.5, 59.7, 36.8, 36.5, 14.5.

HRMS (ESI-TOF): *m/z* calculated for $C_{23}H_{20}O_3$: 345.1485 $[M+H]^+$, found: 345.1485.

$[\alpha]_D^{20}$(c = 1, $CHCl_3$, *e.r.* 99:1): -226.2.

HPLC (YMC-SB, *n*-hexane:IPA = 4:1, flow = 1.0 mL min^{-1}, T_{Column} = 10 °C, λ = 250 nm) t_r: 8.8 min (major), 25.4 min (minor).

Semi-preparative HPLC (Dionex Ultimate 3000, *n*-hexane with 7.5 % EtOAc, flow = 5.0 mL min^{-1}, λ = 230 nm) t_r: 17.5 min.

Methyl (S,Z)-2-(1-phenyl-1,2-dihydro-3H-benzo[f]chromen-3-ylidene)acetate (3ab)

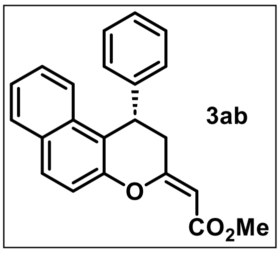

3ab

Product **3ab** was synthesized analogously to the general procedure *GP6* on a scale of 0.1 mmol. In order to remove the side product (arising from the addition of the sulfinate leaving group to the allenoate) the resulting mixture was additionally purified *via* semi-preparative column chromatography, to obtain the final product as a white amorphous residue in an isolated yield of 72% and an e.r. of 99:1.

^{1}H-NMR (300 MHz, $CDCl_3$, 298 K) δ / ppm: 7.84 – 7.80 (m, 2H, Ar-**H**), 7.74 – 7.71 (m, 1H, Ar-**H**), 7.46 (d, J = 9.0 Hz, 1H, Ar-**H**), 7.44 – 7.34 (m, 2H, Ar-**H**), 7.28 – 7.15 (m, 3H, Ar-**H**), 7.12 – 7.08 (m, 2H, Ar-**H**), 4.91 (d, J = 1.7 Hz, 1H, -C=C**H**), 4.80 (dd, J = 6.3, 1.9 Hz, 1H, -C**H**), 3.71 (s, 3H, -C**H₃**), 3.17 (ddd, J = 14.6, 6.4, 1.8 Hz, 1H, -C**H₂**), 2.78 (dd, J = 14.6, 1.9 Hz, 1H, -C**H₂**).

^{13}C-NMR (75 MHz, $CDCl_3$, 298 K) δ / ppm: 165.2, 160.6, 149.6, 142.0, 131.7, 130.6, 129.6, 128.9, 128.8, 127.7, 127.2, 124.6, 122.8, 118.3, 116.7, 98.1, 51.0, 36.8, 36.5.

HRMS (ESI-TOF): *m/z* calculated for $C_{22}H_{18}O_3$: 331.1329 [M+H]$^+$, found: 331.1327.

$[\alpha]_D^{20}$(c = 1, $CHCl_3$, *e.r.* 99:1): -285.9.

HPLC (YMC-SB, *n*-hexane:IPA = 10:1, flow = 1.0 mL min^{-1}, T_{Column} = 10 °C, λ = 272 nm) t_r: 16.1 min (major), 56.4 min (minor).

Semi-preparative HPLC (Dionex Ultimate 3000, *n*-hexane with 7.5 % EtOAc, flow = 5.0 mL min^{-1}, *l* = 230 nm) t_r: 20.1 min.

tert-Butyl (S,Z)-2-(1-phenyl-1,2-dihydro-3H-benzo[f]chromen-3-ylidene)acetate (3ac)

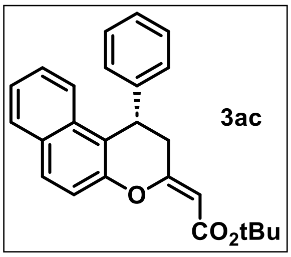

Product **3ac** was synthesized analogously to the general procedure *GP6* on a scale of 0.1 mmol. In order to remove the side product (arising from the addition of the sulfinate leaving group to the allenoate) the resulting mixture was additionally purified *via* semi-preparative column chromatography, to obtain the final product as a white amorphous residue in an isolated yield of 79% and an e.r. of 99:1.

^{1}H-NMR (300 MHz, $CDCl_3$, 298 K) δ / ppm: 7.82 – 7.79 (m, 2H, Ar-**H**), 7.73 – 7.70 (m, 1H, Ar-**H**), 7.46 (d, *J* = 9.0 Hz, 1H, Ar-**H**), 7.42 – 7.33 (m, 2H, Ar-**H**), 7.28 – 7.16 (m, 3H, Ar-**H**), 7.13 – 7.09 (m, 2H, Ar-**H**), 4.82 (d, *J* = 1.8 Hz, 1H, -C=C**H**), 4.78 (dd, *J* = 6.6, 1.9 Hz, 1H, -C**H**), 3.15 (ddd, *J* = 14.6, 6.4, 1.9 Hz, 1H, -C**H$_2$**), 2.73 (dd, *J* = 14.7, 1.9 Hz, 1H, -C**H$_2$**), 1.49 (s, 9H, -C**H$_3$**).

^{13}C-NMR (75 MHz, $CDCl_3$, 298 K) δ / ppm: 164.3, 159.4, 149.7, 142.3, 131.8, 130.5, 129.5, 128.9, 128.7, 127.7, 127.1, 124.5, 122.8, 118.5, 116.7, 100.3, 79.8, 36.8, 36.4, 28.5.

HRMS (ESI-TOF): *m/z* calculated for $C_{25}H_{24}O_3$: 395.1618 [M+Na]$^+$, found: 395.1619.

$[\alpha]_D^{20}$(c = 1, $CHCl_3$, *e.r.* 99:1): -167.0.

HPLC (YMC-SZ, *n*-hexane:IPA = 25:1, flow = 1.0 mL min^{-1}, T_{Column} = 10 °C, λ = 272 nm) t_r: 7.9 min (minor), 8.8 min (major).

Semi-preparative HPLC (Dionex Ultimate 3000, *n*-hexane with 7.5 % EtOAc, flow = 5.0 mL min^{-1}, *l* = 230 nm) t_r: 10.3 min.

Benzyl (S,Z)-2-(1-phenyl-1,2-dihydro-3H-benzo[f]chromen-3-ylidene)acetate (3ad)

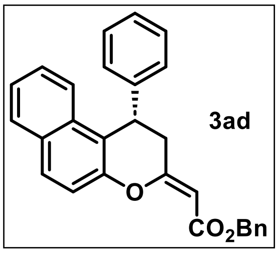

Product **3ad** was synthesized analogously to the general procedure *GP6* on a scale of 0.1 mmol. In order to remove the side product (arising from the addition of the sulfinate leaving group to the allenoate) the resulting mixture was additionally purified *via* semi-preparative column chromatography, to obtain the final product as a white amorphous residue in an isolated yield of 79% and an e.r. of >99:1.

^{1}H-NMR (300 MHz, $CDCl_3$, 298 K) *δ* / ppm: 7.84 – 7.80 (m, 2H, Ar-**H**), 7.74 – 7.71 (m, 1H, Ar-**H**), 7.44 (d, *J* = 9.1 Hz, 1H, Ar-**H**), 7.44 – 7.31 (m, 7H, Ar-**H**), 7.28 – 7.16 (m, 3H, Ar-**H**), 7.12 – 7.08 (m, 2H, Ar-**H**), 5.16 (dd, *J* = 16.4, 12.7 Hz, 2H, -C**H₂**), 4.96 (d, *J* = 1.7 Hz, 1H, -C=C**H**), 4.81 (dd, *J* = 6.5, 1.9 Hz, 1H, -C**H**), 3.18 (ddd, *J* = 14.6, 6.4, 1.8 Hz, 1H, -C**H₂**), 2.79 (dd, *J* = 14.6, 1.9 Hz, 1H, -C**H₂**).

^{13}C-NMR (75 MHz, $CDCl_3$, 298 K) *δ* / ppm: 164.5, 160.9, 149.6, 142.0, 136.7, 131.7, 130.6, 129.7, 129.0, 128.8, 128.6, 128.2, 128.1, 127.7, 127.2, 124.7, 122.8, 118.3, 116.8, 98.2, 65.6, 36.8, 36.5.

HRMS (ESI-TOF): *m/z* calculated for $C_{28}H_{22}O_3$: 407.1642 $[M+H]^+$, found: 407.1643.

$[\alpha]_D^{20}$(c = 1, $CHCl_3$, *e.r.* 99:1): -257.4.

HPLC (YMC-SB, *n*-hexane:IPA = 4:1, flow = 1.0 mL min^{-1}, T_{Column} = 10 °C, *λ* = 272 nm) t_r: 10.5 min (major), 35.3 min (minor).

Semi-preparative HPLC (Dionex Ultimate 3000, *n*-hexane with 7.5 % EtOAc, flow = 5.0 mL min^{-1}, *λ* = 230 nm) t_r: 15.9 min.

***tert*-Butyl (S,Z)-2-(1-(p-tolyl)-1,2-dihydro-3H-benzo[f]chromen-3-ylidene)acetate (3ae)**

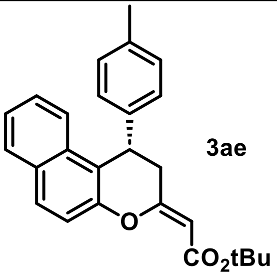

Product **3ae** was synthesized analogously to the general procedure *GP6* on a scale of 0.1 mmol. In order to remove the side product (arising from the addition of the sulfinate leaving group to the allenoate) the resulting mixture was additionally purified *via* semi-preparative column chromatography, to obtain the final product as a white amorphous residue in an isolated yield of 81% and an e.r. of >99:1.

^{1}H-NMR (300 MHz, $CDCl_3$, 298 K) *δ* / ppm: 7.81 – 7.78 (m, 2H, Ar-**H**), 7.73 – 7.70 (m, 1H, Ar-**H**), 7.45 (d, *J* = 9.0 Hz, 1H, Ar-**H**), 7.42 – 7.32 (m, 2H, Ar-**H**), 7.06 – 6.98 (m, 4H, Ar-**H**), 4.82 (d, *J* = 1.7 Hz, 1H, -C=C**H**), 4.74 (dd, 1H, -C**H**), 3.13 (ddd, *J* = 14.6, 6.4, 1.9 Hz, 1H, -C**H₂**), 2.71 (dd, *J* = 14.6, 1.9 Hz, 1H, -C**H₂**), 2.27 (s, 3H, -C**H₃**), 1.49 (s, 9H, -C**H₃**).

^{13}C-NMR (75 MHz, $CDCl_3$, 298 K) *δ* / ppm: 164.3, 159.6, 149.6, 139.3, 136.7, 131.8, 130.5, 129.6, 129.4, 128.7, 127.6, 127.1, 124.5, 122.8, 118.5, 116.9, 100.2, 79.7, 36.5, 36.5, 28.5, 21.2.

HRMS (ESI-TOF): *m/z* calculated for $C_{26}H_{26}O_3$: 409.1774 $[M+Na]^+$, found: 409.1773.

$[\alpha]_D^{20}$(c = 1, $CHCl_3$, *e.r.* 99:1): -237.5.

HPLC (YMC-SZ, *n*-hexane:IPA = 100:1, flow = 1.0 mL min^{-1}, T_{Column} = 10 °C, *λ* = 272 nm) t_r: 10.4 min (minor), 11.9 min (major).

Semi-preparative HPLC (Dionex Ultimate 3000, *n*-hexane with 5 % EtOAc, flow = 5.0 mL min^{-1}, *λ* = 230 nm) t_r: 14.7 min.

tert-Butyl (S,Z)-2-(1-(4-(tert-butyl)phenyl)-1,2-dihydro-3H-benzo[f]chromen-3-ylidene)acetate (3af)

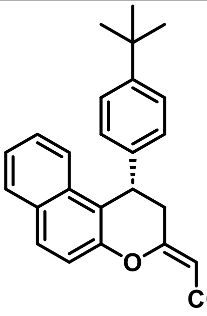

3af

Product **3af** was synthesized analogously to the general procedure *GP6* on a scale of 0.1 mmol. After purification, the final product was obtained as a yellow oil in an isolated yield of 83% and an e.r. of 99:1.

^{1}H-NMR (300 MHz, $CDCl_3$, 298 K) δ / ppm: 7.69 – 7.62 (m, 3H, Ar-**H**), 7.34 (d, J = 9.0 Hz, 1H, Ar-**H**), 7.32 – 7.21 (m, 2H, Ar-**H**), 7.15 – 7.11 (m, 2H, Ar-**H**), 6.94 – 6.90 (m, 2H, Ar-**H**), 4.74 (d, J = 1.7 Hz, 1H, -C=C**H**), 4.62 (dd, J = 6.4, 1.9 Hz, 1H, -C**H**), 3.01 (ddd, J = 14.7, 6.4, 1.9 Hz, 1H, -C**H₂**), 2.63 (dd, J = 14.7, 1.9 Hz, 1H, -C**H₂**), 1.40 (s, 9H, -C**H₃**), 1.14 (s, 9H, -C**H₃**).

^{13}C-NMR (75 MHz, $CDCl_3$, 298 K) δ / ppm: 164.4, 159.8, 149.7, 149.5, 139.1, 131.8, 130.5, 129.4, 128.7, 127.3, 127.0, 125.8, 124.5, 122.9, 118.5, 117.1, 100.1, 79.7, 36.2, 34.5, 31.4, 28.5.

HRMS (ESI-TOF): *m/z* calculated for $C_{29}H_{32}O_3$: 451.2244 [M+Na]$^+$, found: 451.2245.

$[\alpha]_D^{20}$(c = 1, $CHCl_3$, *e.r.* 99:1): -175.5.

HPLC (YMC-SB, *n*-hexane:IPA = 50:1, flow = 1.0 mL min^{-1}, T_{Column} = 10 °C, λ = 272 nm) t_r: 10.0 min (major), 34.8 min (minor).

tert-Butyl (S,Z)-2-(1-(4-(trifluoromethyl)phenyl)-1,2-dihydro-3H-benzo[f]chromen-3-ylidene)acetate (3ag)

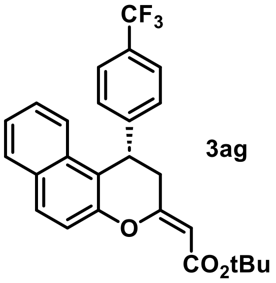

3ag

Product **3ag** was synthesized analogously to the general procedure *GP6* on a scale of 0.1 mmol. In order to remove the side product (arising from the addition of the sulfinate leaving group to the allenoate) the resulting mixture was additionally purified *via* semi-preparative column chromatography, to obtain the final product as a yellow oil in an isolated yield of 63% and an e.r. of 99:1.

^{1}H-NMR (300 MHz, $CDCl_3$, 298 K) δ / ppm: 7.85 – 7.81 (m, 2H, Ar-**H**), 7.65 – 7.62 (m, 1H, Ar-**H**), 7.51 (d, J = 8.1 Hz, 2H, Ar-**H**), 7.47 (d, J = 9.0 Hz, 1H, Ar-**H**), 7.44 – 7.35 (m, 2H, Ar-**H**), 7.24 (d, J = 8.1 Hz, 2H, Ar-**H**), 4.88 – 4.80 (m, 2H, -C**H**), 3.19 (ddd, J = 14.7, 6.4, 1.9 Hz, 1H, -C**H₂**), 2.72 (dd, J = 14.7, 1.9 Hz, 1H, -C**H₂**), 1.50 (s, 9H, -C**H₃**).

^{13}C-NMR (75 MHz, $CDCl_3$, 298 K) δ / ppm: 164.1, 158.6, 149.8, 146.3, 131.5, 130.6, 130.0, 129.5 (q, J = 32.7 Hz), 128.9, 128.1, 127.4, 125.9 (q, J = 3.7 Hz), 124.7, 124.1 (q, J = 271 Hz), 122.4, 118.5, 115.7, 100.7, 80.0, 36.7, 36.1, 28.5.

^{19}F-NMR (282 MHz, $CDCl_3$, 298 K) δ / ppm = -62.5 (3 F, -C**F₃**).

HRMS (ESI-TOF): *m/z* calculated for $C_{26}H_{23}F_3O_3$: 441.1672 [M+H]$^+$, found: 441.1674.

$[\alpha]_D^{20}$(c = 1, $CHCl_3$, *e.r.* 99:1): -37.4.

HPLC (CHIRALPAK® AD-H = 10:1, flow = 1.0 mL min^{-1}, T_{Column} = 10 °C, λ = 250 nm) t_r: 5.4 min (minor), 6.1 min (major).

Semi-preparative HPLC (Dionex Ultimate 3000, *n*-hexane with 15 % EtOAc, flow = 5.0 mL min^{-1}, *l* = 230 nm) t_r: 7.6 min.

tert-Butyl (S,Z)-2-(1-(4-methoxyphenyl)-1,2-dihydro-3H-benzo[f]chromen-3-ylidene)acetate (3ah)

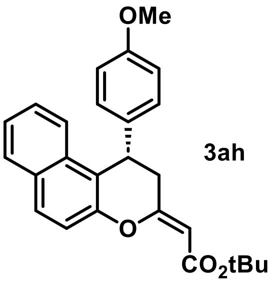

Product **3ah** was synthesized analogously to the general procedure *GP6* on a scale of 0.1 mmol. In order to remove the side product (arising from the addition of the sulfinate leaving group to the allenoate) the resulting mixture was additionally purified *via* semi-preparative column chromatography, to obtain the final product as a yellow oil in an isolated yield of 89% and an e.r. of 99:1.

^{1}H-NMR (300 MHz, $CDCl_3$, 298 K) δ / ppm: 7.81 – 7.78 (m, 2H, Ar-**H**), 7.71 (d, J = 7.9 Hz, 1H, Ar-**H**), 7.45 (d, J = 9.0 Hz, 1H, Ar-**H**), 7.45 – 7.29 (m, 2H, Ar-**H**), 7.05 – 7.00 (m, 2H, Ar-**H**), 6.80 – 6.75 (m, 2H, Ar-**H**), 4.83 (d, J = 1.7 Hz, 1H, -C=C**H**), 4.73 (d, J = 5.1 Hz, 1H, -C**H**), 3.74 (s, 3H, -C**H₃**), 3.12 (ddd, J = 14.5, 6.2, 1.8 Hz, 1H, -C**H₂**), 2.70 (dd, J = 14.6, 1.9 Hz, 1H, -C**H₂**), 1.50 (s, 9H, -C**H₃**).

^{13}C-NMR (75 MHz, $CDCl_3$, 298 K) δ / ppm: 164.3, 159.6, 158.6, 149.6, 134.3, 131.7, 130.5, 129.4, 128.8, 128.7, 127.1, 124.5, 122.8, 118.5, 117.0, 114.2, 100.2, 79.7, 55.3, 36.6, 36.0, 28.5.

HRMS (ESI-TOF): *m/z* calculated for $C_{26}H_{26}O_4$: 425.1723 [M+Na]$^+$, found: 425.1720.

$[\alpha]_D^{20}$(c = 1, $CHCl_3$, *e.r.* 99:1): -178.8.

HPLC (YMC-SB, *n*-hexane:IPA = 20:1, flow = 1.0 mL min^{-1}, T_{Column} = 10 °C, λ = 272 nm) t_r: 11.9 min (minor), 34.6 min (major).

Semi-preparative HPLC (Dionex Ultimate 3000, *n*-hexane with 15 % EtOAc, flow = 5.0 mL min^{-1}, λ = 230 nm) t_r: 7.9 min.

tert-Butyl (S,Z)-2-(1-(4-chlorophenyl)-1,2-dihydro-3H-benzo[f]chromen-3-ylidene)acetate (3ai)

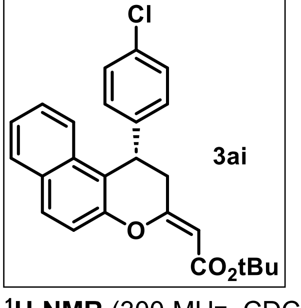

Product **3ai** was synthesized analogously to the general procedure *GP6* on a scale of 0.1 mmol. In order to remove the side product (arising from the addition of the sulfinate leaving group to the allenoate) the resulting mixture was additionally purified *via* semi-preparative column chromatography, to obtain the final product as a yellowish oil in an isolated yield of 81% and an e.r. of 99:1

^{1}H-NMR (300 MHz, $CDCl_3$, 298 K) δ / ppm: 7.83 – 7.80 (m, 2H, Ar-**H**), 7.67 – 7.64 (m, 1H, Ar-**H**), 7.45 (d, J = 9.1 Hz, 1H, Ar-**H**), 7.43 – 7.34 (m, 2H, Ar-**H**), 7.24 – 7.20 (m, 2H, Ar-**H**), 7.07 – 7.02 (m, 2H,

Ar-**H**), 4.83 (d, J = 1.7 Hz, 1H, -C=C**H**), 4.76 (dd, J = 6.1, 1.1 Hz, 1H, -C**H**), 3.15 (ddd, J = 14.6, 6.3, 1.9 Hz, 1H, -C**H₂**), 2.69 (dd, J = 14.6, 1.9 Hz, 1H, -C$\mathbf{H_2}$), 1.50 (s, 9H, -C$\mathbf{H_3}$).

^{13}C-NMR (75 MHz, $CDCl_3$, 298 K) δ / ppm: 164.1, 158.9, 149.7, 140.7, 133.0, 131.6, 130.6, 129.8, 129.1 (2x), 128.8, 127.3, 124.7, 122.5, 118.5, 116.1, 100.6, 79.9, 36.3, 36.3, 28.5.

HRMS (ESI-TOF): *m/z* calculated for $C_{25}H_{23}ClO_3$: 407.1408 [M+H]$^+$, found: 407.1408.

$[\alpha]_D^{20}$(c = 1, $CHCl_3$, *e.r.* 99:1): -204.6.

HPLC (YMC-SB, *n*-hexane:IPA = 20:1, flow = 1.0 mL min^{-1}, T_{Column} = 10 °C, λ = 272 nm) t_r: 10.4 min (major), 50.3 min (minor).

Semi-preparative HPLC (Dionex Ultimate 3000, *n*-hexane with 15 % EtOAc, flow = 5.0 mL min^{-1}, *l* = 230 nm) t_r: 6.7 min.

***tert*-Butyl (S,Z)-2-(1-(naphthalen-1-yl)-1,2-dihydro-3H-benzo[f]chromen-3-ylidene)acetate (3aj)**

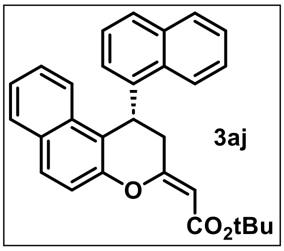

Product **3aj** was synthesized analogously to the general procedure *GP6* on a scale of 0.1 mmol. In order to remove the side product (arising from the addition of the sulfinate leaving group to the allenoate) the resulting mixture was additionally purified *via* semi-preparative column chromatography, to obtain the final product as a yellow oil in an isolated yield of 61% and an e.r. of 99:1

^{1}H-NMR (300 MHz, $CDCl_3$, 298 K) δ / ppm: 8.35 (d, J = 8.5 Hz, 1H, Ar-**H**), 7.96 (dd, J = 8.0, 0.8 Hz, 1H, Ar-**H**), 7.88 – 7.81 (m, 2H, Ar-**H**), 7.73 – 7.67 (m, 2H, Ar-**H**), 7.60 (ddd, J = 8.0, 6.8, 1.2 Hz, 1H, Ar-**H**), 7.52 (d, J = 8.9 Hz, 1H, Ar-**H**), 7.51 (d, J = 8.2 Hz, 1H, Ar-**H**), 7.36 – 7.24 (m, 2H, Ar-**H**), 7.19 (dd, J = 8.1, 7.4 Hz, 1H, Ar-**H**), 6.79 (dd, J = 7.3, 1.2 Hz, 1H, Ar-**H**), 5.60 (d, J = 6.5 Hz, 1H, -C**H**), 4.65 (d, J = 1.7 Hz, 1H, -C=C**H**), 3.27 (ddd, J = 14.5, 6.6, 1.8 Hz, 1H, -C$\mathbf{H_2}$), 2.90 (dd, J = 14.5, 1.7 Hz, 1H, -C$\mathbf{H_2}$), 1.46 (s, 9H, -C$\mathbf{H_3}$).

^{13}C-NMR (75 MHz, $CDCl_3$, 298 K) δ / ppm: 164.2, 159.5, 150.4, 136.9, 134.4, 131.7, 130.6, 130.4, 129.7, 129.7, 128.7, 127.9, 127.2, 126.8, 126.4, 125.9, 125.8, 124.6, 122.8, 122.3, 118.4, 116.7, 100.5, 79.7, 35.0, 32.6, 28.5.

HRMS (ESI-TOF): *m/z* calculated for $C_{29}H_{26}O_3$: 445.1774 [M+Na]$^+$, found: 445.1776.

$[\alpha]_D^{20}$(c = 1, $CHCl_3$, *e.r.* 99:1): -39.0.

HPLC (YMC-SB, *n*-hexane:IPA = 25:1, flow = 1.0 mL min^{-1}, T_{Column} = 10 °C, λ = 250 nm) t_r: 12.8 min (minor), 15.4 min (major).

Semi-preparative HPLC (Dionex Ultimate 3000, *n*-hexane with 7.5 % EtOAc, flow = 5.0 mL min^{-1}, *l* = 230 nm) t_r: 10.9 min.

tert-Butyl (S,Z)-2-(8-methyl-4-phenylchroman-2-ylidene)acetate (3am)

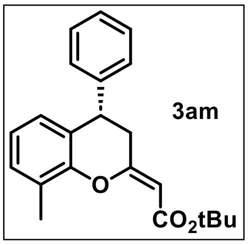

Product **3am** was synthesized analogously to the general procedure *GP6* on a scale of 0.1 mmol. After purification, the final product was obtained as a yellow oil in an isolated yield of 23% and an e.r. of 99:1.

^{1}H-NMR (300 MHz, $CDCl_3$, 298 K) δ / ppm: 7.35 – 7.23 (m, 3H, Ar-**H**), 7.15 – 7.08 (m, 3H, Ar-**H**), 6.84 (t, *J* = 7.5 Hz, 1H, Ar-**H**), 6.67 (d, *J* = 7.6 Hz, 1H, Ar-**H**), 4.88 (s, 1H, -C=C**H**), 4.13 (dd, *J* = 8.0, 5.3 Hz, 1H, -C**H**), 2.86 (ddd, *J* = 14.6, 5.4, 1.0 Hz, 1H, -C**H$_2$**), 2.76 (ddd, *J* = 14.6, 8.0, 1.0 Hz, 1H, -C**H$_2$**), 2.46 (s, 3H, -C**H$_3$**), 1.51 (s, 9H, -C**H$_3$**).

^{13}C-NMR (75 MHz, $CDCl_3$, 298 K) δ / ppm: 164.5, 160.4, 150.1, 142.0, 130.0, 128.9, 128.2, 127.3, 126.6, 126.2, 125.6, 122.5, 99.0, 79.7, 40.6, 36.3, 28.6, 16.2.

HRMS (ESI-TOF): *m/z* calculated for $C_{22}H_{24}O_3$: 373.1798 $[M+H]^+$, found: 373.1801.

$[\alpha]_D^{20}$(c = 1, $CHCl_3$, *e.r.* 99:1): +72.1.

HPLC (YMC-SB, *n*-hexane:IPA = 10:1, flow = 1.0 mL min^{-1}, T_{Column} = 10 °C, λ = 230 nm) t_r: 6.8 min (minor), 7.4 min (major).

Diethyl (S,Z)-2-(1-phenyl-1,2-dihydro-3H-benzo[f]chromen-3-ylidene)succinate (3ao)

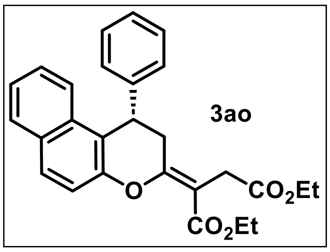

Product **3ao** was synthesized analogously to the general procedure *GP6* on a scale of 0.1 mmol. After purification, the final product was obtained as a colorless oil in an isolated yield of 67% and an e.r. of 99:1.

^{1}H-NMR (300 MHz, $CDCl_3$, 298 K) δ / ppm: 7.82 – 7.79 (m, 2H, Ar-**H**), 7.71 – 7.68 (m, 1H, Ar-**H**), 7.42 – 7.32 (m, 3H, Ar-**H**), 7.26 – 7.10 (m, 5H, Ar-**H**), 4.82 (dd, *J* = 6.1, 2.1 Hz, 1H, -C**H**), 4.27 (q, *J* = 7.1 Hz, 2H, -C**H$_2$**), 4.00 (qq, *J* = 7.1, 7.1, 7.1, 7.1 Hz, 2H, -C**H$_2$**), 3.24 – 3.14 (m, 2H, -C**H$_2$**), 3.01 – 2.90 (m, 2H, -C**H$_2$**), 1.35 (t, *J* = 7.1 Hz, 3H, -C**H$_3$**), 1.17 (t, *J* = 7.2 Hz, 3H, -C**H$_3$**).

^{13}C-NMR (75 MHz, $CDCl_3$, 298 K) δ / ppm: 171.2, 166.4, 156.8, 150.0, 142.2, 131.7, 130.3, 129.6, 128.8, 128.7, 127.5, 127.1, 124.4, 122.7, 118.2, 116.1, 105.2, 60.9, 60.6, 37.1, 33.9, 32.8, 14.4, 14.2.

HRMS (ESI-TOF): *m/z* calculated for $C_{27}H_{26}O_5$: 431.1853 $[M+H]^+$, found: 431.1854.

$[\alpha]_D^{20}$(c = 1, $CHCl_3$, *e.r.* 99:1): -82.8.

HPLC (YMC-SB, *n*-hexane:IPA = 10:1, flow = 1.0 mL min^{-1}, T_{Column} = 10 °C, λ = 272 nm) t_r: 15.8 min (major), 26.4 min (minor).

4-(*tert*-Butyl) 1-ethyl (S,Z)-2-(1-phenyl-1,2-dihydro-3H-benzo[f]chromen-3-ylidene)succinate (3ap)

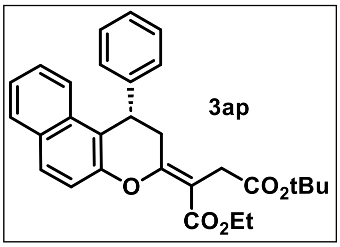

Product **3ap** was synthesized analogously to the general procedure *GP6* on a scale of 0.1 mmol. After purification, the final product was obtained as a white viscous residue in an isolated yield of 58% and an e.r. of 99:1.

^{1}H-NMR (300 MHz, $CDCl_3$, 298 K) δ / ppm: 7.81 – 7.78 (m, 2H, Ar-**H**), 7.71 – 7.68 (m, 1H, Ar-**H**), 7.41 – 7.31 (m, 3H, Ar-**H**), 7.25 – 7.10 (m, 5H, Ar-**H**), 4.82 (dd, *J* = 6.2, 2.1 Hz, 1H, -C**H**), 4.26 (q, *J* = 7.1 Hz, 2H, -C**H$_2$**), 3.20 – 3.09 (m, 2H, -C**H$_2$**), 2.97 (dd, *J* = 14.8, 6.1 Hz, 1H, -C**H$_2$**), 2.82 (d, *J* = 17.0 Hz, 1H, -C**H$_2$**), 1.37 (s, 9H, -C**H$_3$**), 1.35 (t, *J* = 7.1 Hz, 3H, -C**H$_3$**).

^{13}C-NMR (75 MHz, $CDCl_3$, 298 K) δ / ppm: 170.4, 166.4, 156.1, 150.0, 142.3, 131.8, 130.3, 129.5, 128.9, 128.7, 127.5, 127.1 (2x), 124.3, 122.7, 118.3, 116.1, 105.7, 80.9, 60.4, 37.2, 35.0, 32.6, 28.1, 14.5.

HRMS (ESI-TOF): *m/z* calculated for $C_{29}H_{30}O_5$: 481.1985 $[M+Na]^+$, found: 481.1988.

$[\alpha]_D^{20}$(c = 1, $CHCl_3$, *e.r.* 99:1): -40.3.

HPLC (YMC-SB, *n*-hexane:IPA = 10:1, flow = 1.0 mL min^{-1}, T_{Column} = 10 °C, λ = 272 nm) t_r: 7.3 min (major), 10.9 min (minor).

Ethyl (S)-3-ethyl-1-phenyl-1H-benzo[f]chromene-2-carboxylate (6aq)

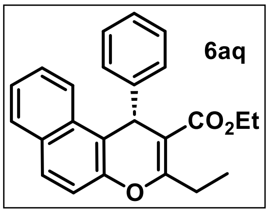

Product **6aq** was synthesized analogously to the general procedure *GP6* on a scale of 0.1 mmol. After purification, the final product was obtained as a colorless oil in an isolated yield of 39% and an e.r. of 67:33.

^{1}H-NMR (300 MHz, $CDCl_3$, 298 K) δ / ppm: 7.99 (d, *J* = 8.4 Hz, 1H, Ar-**H**), 7.78 – 7.72 (m, 2H, Ar-**H**), 7.46 – 7.30 (m, 5H, Ar-**H**), 7.21 – 7.16 (m, 2H, Ar-**H**), 7.11 – 7.05 (m, 1H, Ar-**H**), 5.66 (s, 1H, -C**H**), 2.99 (dq, *J* = 13.3, 7.4 Hz, 1H, -C**H$_2$**), 2.86 (dq, *J* = 13.6, 7.5 Hz, 1H, -C**H$_2$**), 1.34 (t, *J* = 7.1 Hz, 3H, -C**H$_3$**), 1.27 (t, *J* = 7.5 Hz, 3H, -C**H$_3$**).

^{13}C-NMR (75 MHz, $CDCl_3$, 298 K) δ / ppm: 167.2, 164.4, 148.0, 145.7, 131.4, 131.1, 128.8, 128.6, 128.5, 128.4, 126.9, 126.5, 124.7, 123.3, 117.4, 117.2, 106.8, 60.5, 38.6, 26.2, 14.4, 11.9.

HRMS (ESI-TOF): *m/z* calculated for $C_{24}H_{22}O_3$: 359.1642 $[M+H]^+$, found: 359.1643.

$[\alpha]_D^{20}$(c = 1, $CHCl_3$, *e.r.* 67:33): +23.9.

HPLC (YMC-SA, *n*-hexane:IPA = 10:1, flow = 1.0 mL min^{-1}, T_{Column} = 10 °C, λ = 272 nm) t_r: 4.4 min (major), 6.4 min (minor).

7. Follow-Up Transformations

***tert*-Butyl 2-(8-(1-methyl-1H-indol-2-yl)-7,8-dihydro-6H-[1,3]dioxolo[4,5-g]chromen-6-yl)acetate (8)**

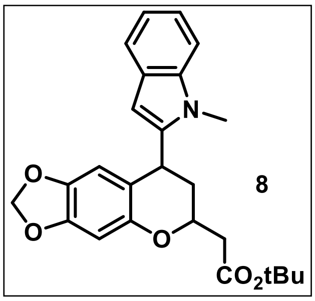

Following the procedure reported in the literature,[11] a sealed vial was charged with **3n** (42.5 mg, 0.101 mmol) and Wilkinson's catalyst (9.7 mg, 10 mol%). Next, the mixture was dissolved in EtOAc (0.05 M). After piercing the seal with a needle, the vial was placed in an autoclave and subsequently filled with H_2 to a total pressure of 50 bar. After stirring over night at 60 °C, the autoclave was cooled and flushed with air. The residue was concentrated to dryness and consequently purified *via* preparative TLC (heptane:EtOAc = 1:1), to obtain the pure product **8** as a light brownish amorphous solid in a yield of 65%.

^{1}H-NMR (300 MHz, $CDCl_3$, 298 K) *δ* / ppm: 7.56 (d, *J* = 7.7 Hz, 1H, Ar-**H**), 7.29 (d, *J* = 8.1 Hz, 1H, Ar-**H**), 7.23 – 7.17 (m, 1H, Ar-**H**), 7.13 – 7.08 (m, 1H, Ar-**H**), 6.40 (s, 1H, Ar-**H**), 6.36 (s, 1H, Ar-**H**), 6.29 (d, *J* = 0.5 Hz, 1H, Ar-**H**), 5.83 (dd, *J* = 4.7, 1.3 Hz, 2H, -C**H₂**), 4.57 – 4.45 (m, 2H, -C**H**), 3.53 (s, 3H, -C**H₃**), 2.74 (dd, *J* = 15.3, 7.1 Hz, 1H, -C**H₂**), 2.56 (dd, *J* = 15.3, 6.2 Hz, 1H, -C**H₂**), 2.32 (ddd, *J* = 13.4, 6.2, 1.9 Hz, 1H, -C**H₂**), 2.13 – 2.01 (m, 1H, -C**H₂**), 1.49 (s, 9H, -C**H₃**).

^{13}C-NMR (75 MHz, $CDCl_3$, 298 K) *δ* / ppm: 169.9, 149.5, 147.1, 142.1, 141.7, 138.1, 127.7, 121.4, 120.3, 119.7, 115.3, 109.1, 107.6, 101.4, 101.1, 98.8, 81.2, 73.3, 42.1, 35.5, 35.3, 30.5, 28.3.

HRMS (ESI-TOF): *m/z* calculated for $C_{25}H_{27}NO_5$: 422.1962 $[M+H]^+$, found: 422.1961.

(S,Z)-2-(1-phenyl-1,2-dihydro-3H-benzo[f]chromen-3-ylidene)ethan-1-ol (9)

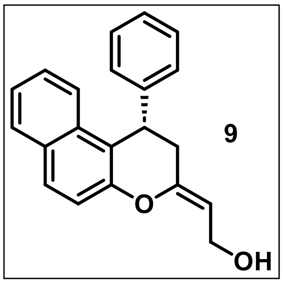

According to literature procedure,[12] a flame-dried reaction flask was charged with **3ac** (37.2 mg, 0.0999 mmol, e.r. 99: 1) and anhydrous THF (5 mL). The solution was cooled to 0 °C with an ice bath, followed by the addition of $LiAlH_4$ (56.9 mg, 1.50 mmol, 15 equiv.) in 5 portions to the reaction mixture. Afterwards, the suspension was stirred at rt. for 2.5 h under an argon atmosphere. After that, the reaction was carefully quenched by the addition of 5 mL 1 M NaOH and the phases were separated. Then the aqueous phase was extracted three times with EtOAc, the collected organic phases were dried with Na_2SO_4, filtered and evaporated to dryness. The crude product was purified by preparative TLC (heptane:EtOAc = 1:1) to give the desired alcohol as colourless viscous residue in a yield of 70% and an e.r. of 99:1.

^{1}H-NMR (300 MHz, $CDCl_3$, 298 K) *δ* / ppm: 7.80-7.76 (m, 2 H, Ar-**H**), 7.71-7.68 (m, 1 H, Ar-**H**), 7.40-7.27 (m, 3 H, Ar-**H**), 7.25-7.17 (m, 3 H, Ar-**H**), 7.11-7.08 (m, 2 H, Ar-**H**), 4.78 (dd, *J* = 5.6, 1.6 Hz 1 H, -C=C-**H**), 4.64 (td, *J* = 7.0, 1.9 Hz, 1 H, -C**H**), 4.42 (ddd, *J* = 12.3, 7.0, 1.6 Hz, 1 H, -C**H₂**), 4.31 (ddd, *J* = 12.3, 7.0, 1.1 Hz, 1 H, -C**H₂**), 3.10-3.03 (m, 1 H, -CH-C**H₂**), 2.67 (dd, *J* = 14.0, 1.9 Hz, 1 H, -CH-C**H₂**-).

^{13}C-NMR (75 MHz, $CDCl_3$, 298 K) *δ* / ppm: 150.4, 147.8, 143.5, 132.2, 130.0, 129.4, 128.6, 128.5, 127.9, 127.0, 126.8, 124.0, 122.8, 118.1, 116.1, 107.5, 56.5, 37.8, 35.2.

HRMS (ESI-TOF): *m/z* calculated for $C_{21}H_{19}O_2$: 303.1380 $[M+H]^+$, found 303.1382.

$[\alpha]_D^{20}$(c = 1, $CHCl_3$, *e.r.* 99:1): -64.5.

HPLC (YMC-SB, *n*-hexane:IPA = 1:1, flow = 1.0 mL min^{-1}, T_{Column} = 10 °C, λ = 272 nm) t_r: 4.59 min (major), 6.98 min (minor).

8. VCD Investigations

To assign the absolute configuration of products **3** we recorded VCD (vibrational circular dichroism) spectra of both enantiomers of compound **3e** (synthesized by using both enantiomers of **ITU3**) and compared the experimental spectra with those calculated from DFT optimized structures.

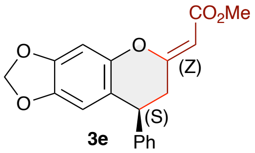

3e

VCD Measurements: The VCD spectra of (-)-3e and (+)-3e at a resolution of 4 cm−1 were recorded in CDCl3 solution using a Jasco FVS-6000 (MCT-V detector). A BaF2 cell of 200 µm pathlength and sample concentrations of 27 mg/mL (for each enantiomer) were used. The spectra were averaged for ~7 h (corresponding to 32768 scans). Baseline correction for IR was achieved by subtracting the spectrum of the solvent obtained under the same conditions, baseline correction for the VCD was achieved by subtracting the average of the spectra of the two enantiomers.[13]

Geometry optimizations and the computation of vibrational frequencies and VCD rotatory strengths were carried out with the Gaussian 16 software package[14] at DFT level using the ωB97X-D functional combined with the def2-TZVPPD basis set using an implicit solvation model (CPCM) for $CHCl_3$. The calculated vibrational frequencies were scaled by a factor of 0.9903.[15]

VCD curves were simulated from the calculated wavenumber and rotatory strength data using Lorentzian band shape and a half-width at half-height value of 4 cm^{-1}. The theoretical VCD curves of the **(*S*)-3e** were obtained as population-weighted sums of the calculated spectra of individual conformers, considering a Boltzmann distribution.

Results

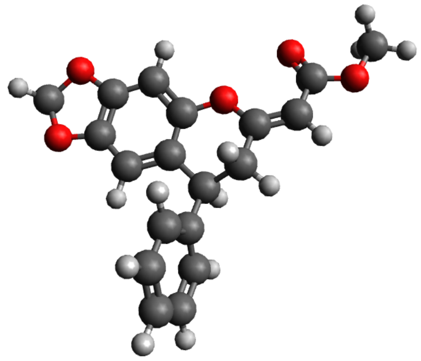

Figure S9: Conformer 1 of **(*S*)-3e** (12.3 %).

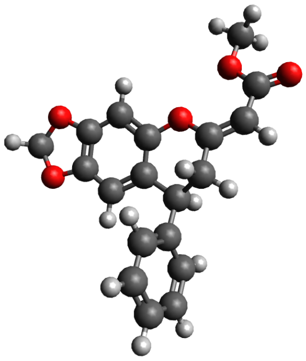

Figure S10: Conformer 2 of **(*S*)-3e** (67.1 %).

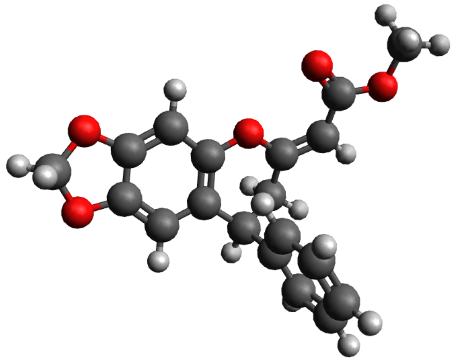

Figure S11: Conformer 3 of **(*S*)-3e** (3.2 %).

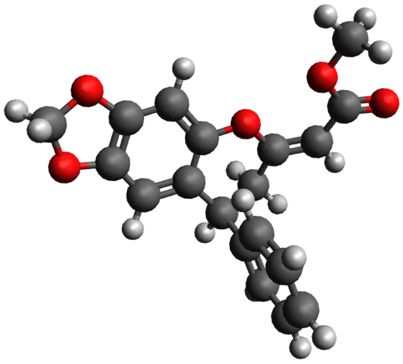

Figure S12: Conformer 4 of **(*S*)-3e** (17.5 %).

Table S2: Relative energy (in kJ/mol) and population (Boltzmann distribution) four lowest energy conformations of **(*S*)-3e**.

**conformer**	**ΔE**	**ΔG**	**population**
**Conformer 1**	3.53	4.21	12.3 %
**Conformer 2**	6.54	7.57	3.2 %
**Conformer 3**	0.00	0.00	67.1 %
**Conformer 4**	3.34	3.34	17.5 %

Table S3: Selected torsion angles of the four lowest energy conformations of **(*S*)-3e**.

**Torsion angle**	**Conformer 1**	**Conformer 2**	**Conformer 3**	**Conformer 4**
**C19-C20-O21-C23**	179.9°	-179.8°	-179.7°	179.6°
**C8-C19-C20-O21**	179.1°	-1.0°	178.9°	1.0°
**C4-C5-C10-C9**	30.4°	30.0°	-29.0°	-29.0°
**C5-C10-C9-C8**	-52.8°	-52.8°	52.8°	53.2°
**C10-C9-C8-O7**	45.9°	46.5°	-48.3°	-49.1°
**C5-C10-C14-C30**	-58.3°	-58.1°	-30.4°	-28.7°

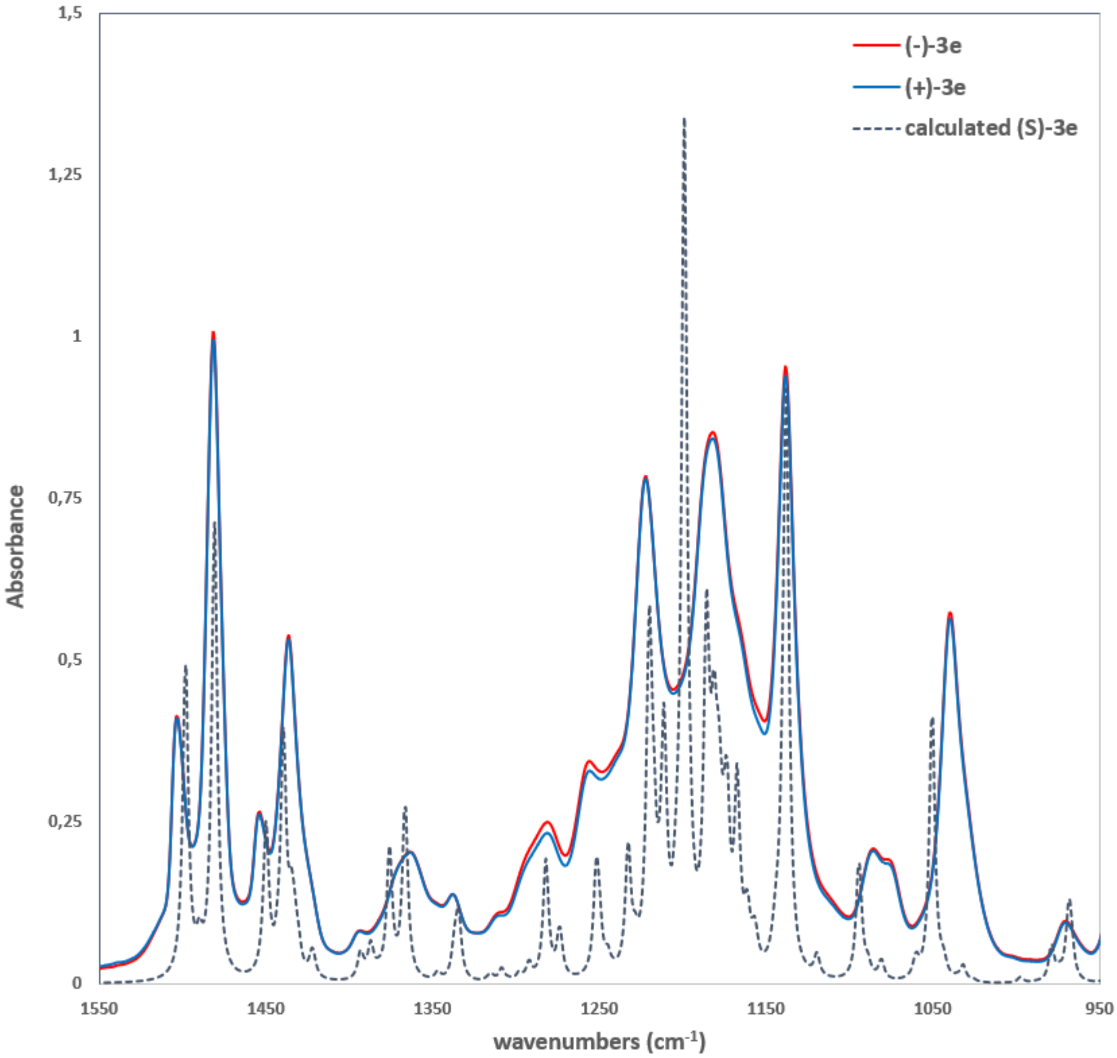

Figure S13: Measured (red & blue, solvent-subtracted) and calculated (dotted line) IR-spectra of **3e**.

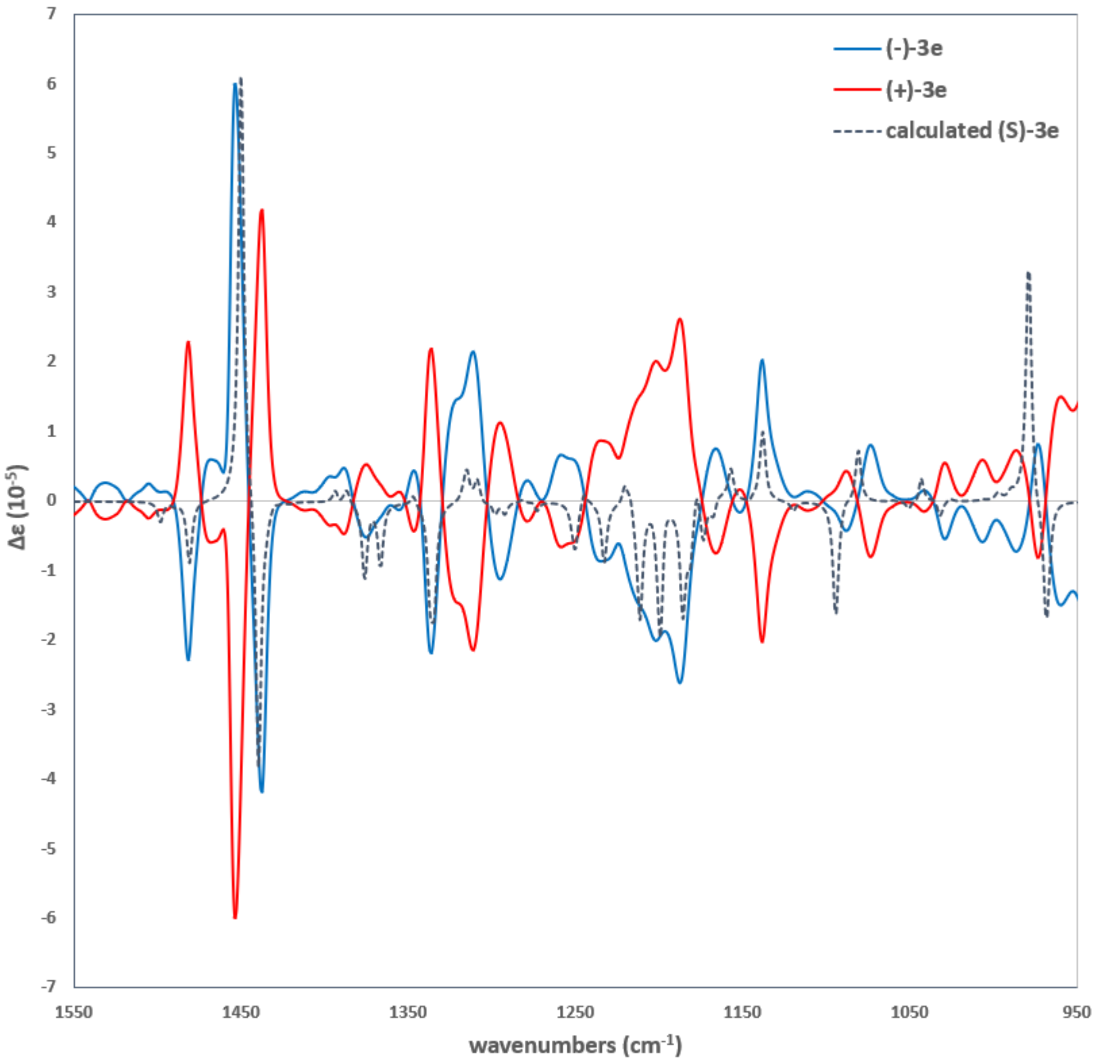

Figure S14: Measured (red & blue, baseline corrected) and calculated (dotted line) VCD-spectra of **3e**.

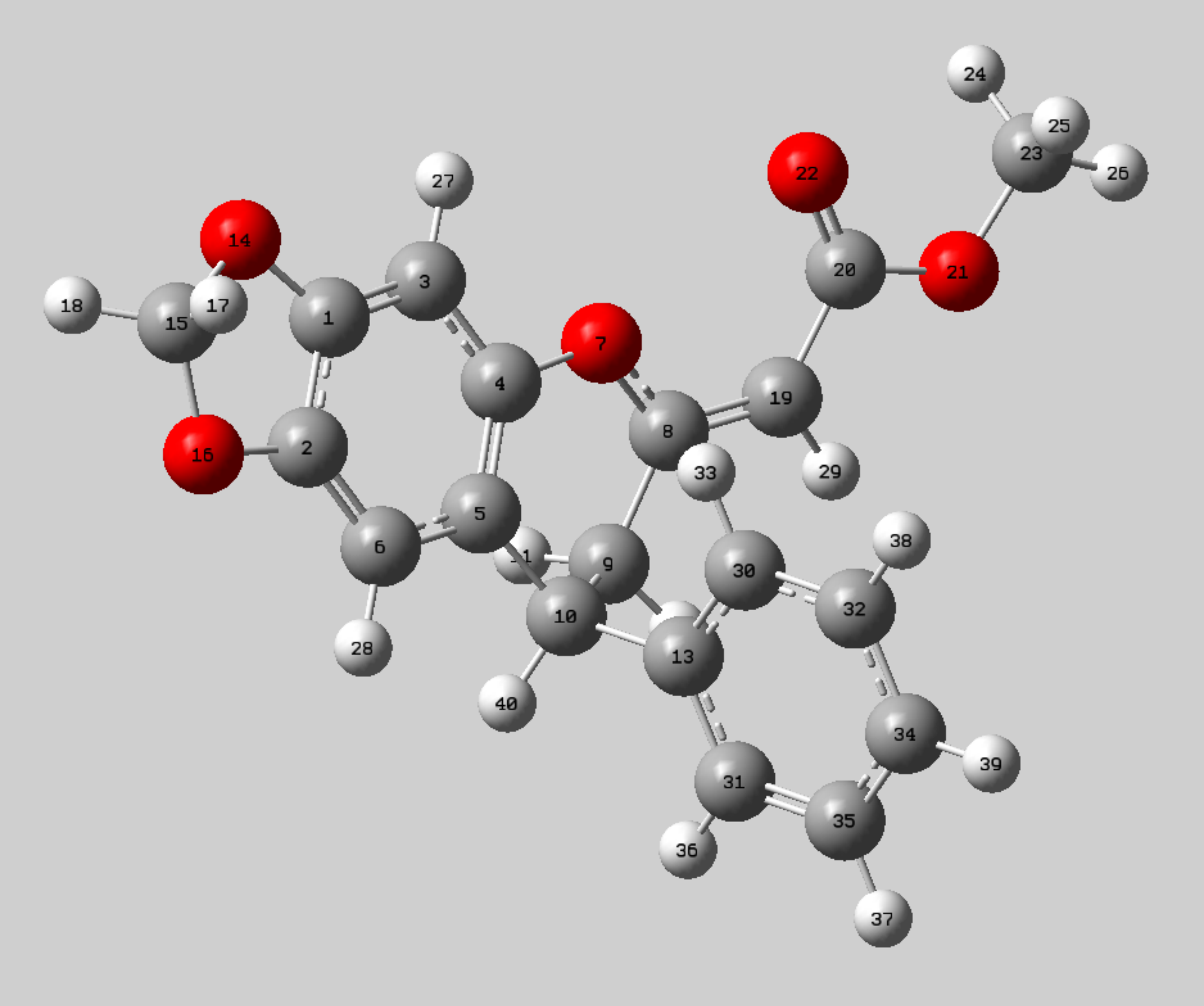

Figure S15: Labelling of atoms in **3e**.

Calculated thermodynamic and optimized geometry data of (*S*)-3e:

Conformer 1

Energy

E: -1109,8225506 Hartree, -2913838,71 kJ/mol
G: -1109,5567775 Hartree, -2913140,92 kJ/mol

Coordinates

C	-1.11461	3.00297	0.12445
C	-2.18330	2.19284	-0.22281
C	0.14603	2.50109	0.29906
C	0.29213	1.12534	0.10367
C	-0.76188	0.29099	-0.23411
C	-2.03940	0.84488	-0.40242
O	1.58963	0.67042	0.20995

C	1.85687	-0.64269	0.27754
C	0.69302	-1.55450	0.49518
C	-0.47605	-1.18285	-0.42532
H	1.00102	-2.58536	0.34125
H	0.37131	-1.45433	1.53604
C	-1.66584	-2.08347	-0.18424
O	-1.54600	4.28273	0.28552
C	-2.87657	4.29733	-0.22556
O	-3.32010	2.94772	-0.28440
H	-3.51658	4.86289	0.44674
H	-2.87099	4.72501	-1.23288
C	3.12000	-1.08825	0.18406
C	4.31325	-0.26610	-0.00326
O	5.40910	-1.04919	-0.03331
O	4.37356	0.93575	-0.12238
C	6.65344	-0.38061	-0.21333
H	6.83571	0.32064	0.59990
H	6.66846	0.15829	-1.15968
H	7.41076	-1.15862	-0.21344
H	0.99312	3.11525	0.56525
H	-2.88273	0.21879	-0.65614
H	3.27576	-2.15292	0.26021
C	-2.32715	-2.08650	1.04214
C	-2.11021	-2.94102	-1.18257
C	-3.40313	-2.92967	1.26364
H	-2.00621	-1.41402	1.82836
C	-3.83795	-3.78526	0.25933
C	-3.18924	-3.78810	-0.96486
H	-1.60863	-2.94730	-2.14260
H	-3.52164	-4.44847	-1.75491
H	-3.90645	-2.91804	2.22142
H	-4.67934	-4.44302	0.43187
H	-0.14951	-1.33668	-1.45804

Conformer 2

Energy

E: -1109,821403 Hartree, -2913835,69 kJ/mol
G: -1109,5554955 Hartree, -2913137,55 kJ/mol

Coordinates

C	-0.42347	3.04279	0.14649
C	-1.63431	2.47535	-0.21515
C	0.70310	2.28782	0.32653
C	0.56153	0.91280	0.12143
C	-0.64031	0.31876	-0.23035
C	-1.77263	1.12814	-0.40393
O	1.73513	0.19792	0.23461
C	1.72477	-1.14448	0.28295
C	0.39432	-1.79346	0.49267
C	-0.66813	-1.18127	-0.42851
H	0.47933	-2.86514	0.33374
H	0.09672	-1.63320	1.53317
C	-2.02084	-1.81366	-0.19329
O	-0.57995	4.38322	0.31459
C	-1.87496	4.67811	-0.20403
O	-2.58832	3.45072	-0.27915
H	-2.38865	5.35843	0.47036
H	-1.77275	5.10430	-1.20676
C	2.86089	-1.85276	0.18091
C	4.23831	-1.39431	0.00079
O	4.38940	-0.07110	-0.09455
O	5.17436	-2.16434	-0.06043
C	5.72368	0.39762	-0.27491
H	6.15082	-0.00019	-1.19415
H	6.35075	0.10749	0.56668
H	5.64937	1.47919	-0.33255
H	1.65666	2.71069	0.60498
H	-2.72570	0.69294	-0.66825
H	2.77198	-2.92623	0.24564
C	-2.67214	-1.67989	1.03112

C	-2.63128	-2.55839	-1.19436
C	-3.90131	-2.27957	1.24821
H	-2.22004	-1.09030	1.81940
C	-4.50209	-3.02421	0.24112
C	-3.86422	-3.16139	-0.98105
H	-2.13910	-2.66871	-2.15292
H	-4.32474	-3.73701	-1.77310
H	-4.39398	-2.16398	2.20459
H	-5.46299	-3.49172	0.40986
H	-0.37806	-1.39582	-1.46126

Conformer 3

Energy

E: -1109,82389 Hartree, -2913842,23 kJ/mol
G: -1109,55838 Hartree, -2913145,13 kJ/mol

Coordinates

C	-3.11052	-1.45746	-0.27142
C	-3.51920	-0.18912	0.11382
C	-1.81081	-1.86782	-0.15703
C	-0.91584	-0.92975	0.36651
C	-1.29936	0.33993	0.76144
C	-2.64292	0.72157	0.63443
O	0.39892	-1.34438	0.40102
C	1.32561	-0.62782	1.05961
C	0.81497	0.41298	2.00265
C	-0.26307	1.27477	1.32667
H	0.36565	-0.09485	2.86049
H	1.64148	1.01946	2.36327
C	0.34421	2.22338	0.30819
O	-4.18454	-2.16761	-0.70842
C	-5.24298	-1.21982	-0.82318
O	-4.86750	-0.07540	-0.06738
H	-5.36440	-0.94061	-1.87420
H	-6.15553	-1.64465	-0.41290
C	2.63243	-0.87168	0.87813

C	3.20460	-1.87338	-0.01917
O	4.54938	-1.82338	0.03525
O	2.61400	-2.65383	-0.72916
C	5.24541	-2.74458	-0.79841
H	4.99421	-3.77062	-0.53314
H	5.00106	-2.57658	-1.84642
H	6.30150	-2.56096	-0.62598
H	-1.47520	-2.85006	-0.45393
H	-2.96527	1.70595	0.94500
H	3.33070	-0.27354	1.44215
C	0.48293	1.88290	-1.03293
C	0.82857	3.45639	0.73653
C	1.09576	2.75267	-1.92402
H	0.10479	0.93447	-1.39253
C	1.57525	3.97791	-1.48690
C	1.43823	4.32893	-0.15115
H	0.72428	3.73693	1.77811
H	1.80342	5.28523	0.19964
H	1.19519	2.47069	-2.96391
H	2.04896	4.65741	-2.18278
H	-0.73248	1.87845	2.10397

Conformer 4

Energy

E: -1109,82271 Hartree, -2913839,12 kJ/mol
G: -1109,55711 Hartree, -2913141,79 kJ/mol

Coordinates

C	3.09176	-1.20586	0.17753
C	3.40806	0.10883	-0.13176
C	1.82705	-1.70391	0.02532
C	0.86819	-0.80670	-0.45542
C	1.15966	0.50784	-0.77511
C	2.47022	0.97984	-0.61142
O	-0.41109	-1.31536	-0.52832
C	-1.38730	-0.62910	-1.14987

C	-0.94417	0.49139	-2.03494
C	0.06086	1.39293	-1.30044
H	-0.44993	0.06213	-2.91075
H	-1.80838	1.05495	-2.37588
C	-0.62493	2.23533	-0.23898
O	4.21217	-1.85859	0.58672
C	5.19665	-0.84315	0.76623
O	4.74287	0.31094	0.07086
H	5.28904	-0.61542	1.83249
H	6.14121	-1.17600	0.34372
C	-2.67798	-0.95998	-0.99272
C	-3.28230	-2.01317	-0.17626
O	-2.42172	-2.74549	0.53506
O	-4.48066	-2.20098	-0.14547
C	-2.99062	-3.77155	1.34485
H	-3.67316	-3.34804	2.08001
H	-3.52774	-4.49313	0.73123
H	-2.15319	-4.25114	1.84219
H	1.56426	-2.72387	0.26234
H	2.72054	2.00119	-0.86345
H	-3.40006	-0.37549	-1.54160
C	-0.71976	1.82756	1.08691
C	-1.22786	3.43355	-0.61260
C	-1.40507	2.59703	2.01682
H	-0.25056	0.90507	1.40412
C	-2.00259	3.78793	1.63406
C	-1.91047	4.20589	0.31368
H	-1.15970	3.76603	-1.64181
H	-2.36832	5.13654	0.00521
H	-1.46891	2.26359	3.04421
H	-2.53365	4.38917	2.35990
H	0.48961	2.07207	-2.03802

9. UV/Vis Spectra and Molar Absorption Coefficients of *o*QMs

UV/Vis photometric measurements were carried out using a J&M TIDAS diode array spectrophotometer, which was controlled by TIDASDAQ3 (v3) software and connected to a Hellma 661.502-QX quartz Suprasil immersion probe (light path d = 5 mm) via fiber optic cables and standard SMA connectors.

Quinone methide solutions [9–14 mM] in dry DMSO were added stepwise to known volumes of the same solvent. The absorbances A of the *o*QM solutions were detected by using a *J&M* TIDAS diode array spectrophotometer (connected to a Hellma quartz probe with a path length d = 0.5 cm).

Molar absorption coefficients ε (M^{-1} cm^{-1}) were determined from the slopes of linear correlations of absorbance with *o*QM concentrations by assuming the validity of the Beer-Lambert law [Equation (S1)].

$$\lg (I_0/I) = A = \varepsilon\, d\, c \qquad \text{(S1)}$$

Solutions of ***o*QM1** in DMSO: Concentration-dependent absorbance *A* at λ_{max}.

V (L)	[***o*QM1**] (M)	*A* (445 nm)
0.02400	0	0.000
0.02402	1.12×10^{-5}	0.086
0.02405	2.79×10^{-5}	0.217
0.02410	5.57×10^{-5}	0.446
0.02415	8.33×10^{-5}	0.666
0.02420	1.11×10^{-4}	0.898
0.02425	1.38×10^{-4}	1.116
0.02430	1.66×10^{-4}	1.334

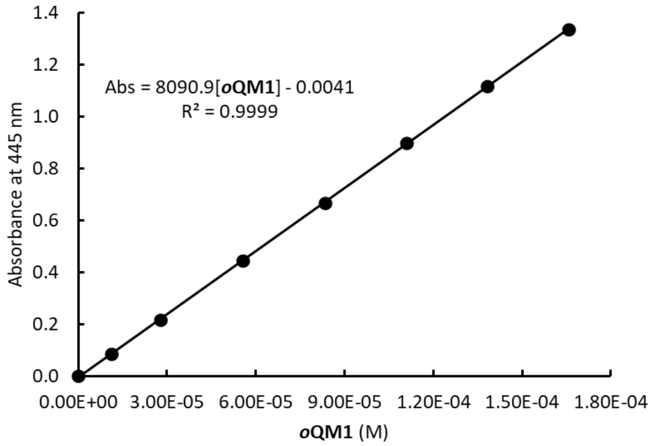

Concentration-dependent absorbance of ***o*QM1** used to determine *ε* (at λ_{max} = 445 nm, *d* = 0.5 cm).

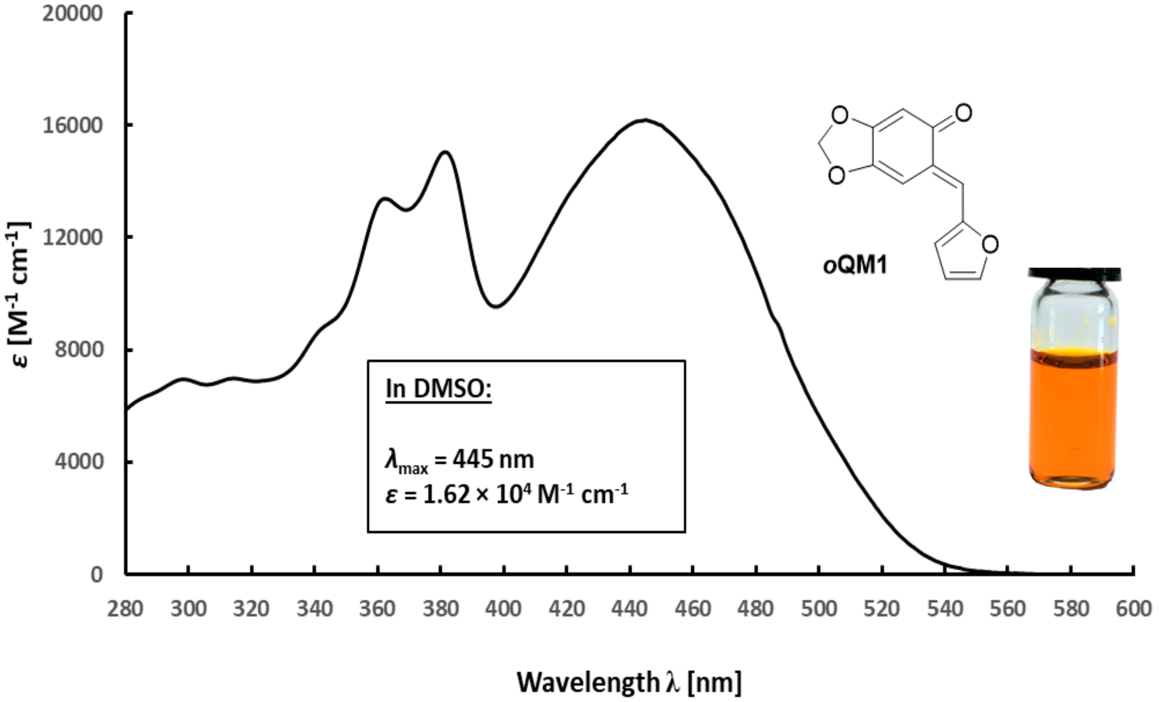

Solutions of ***o*****QM2** in DMSO: Concentration-dependent absorbance *A* at λ_{max}.

V (L)	[***o*****QM2**] (M)	*A* (482 nm)
0.02400	0	0.000
0.02402	8.72×10^{-6}	0.070
0.02405	2.18×10^{-5}	0.169
0.02410	4.34×10^{-5}	0.346
0.02415	6.50×10^{-5}	0.514
0.02420	8.65×10^{-4}	0.683
0.02425	1.08×10^{-4}	0.847
0.02430	1.29×10^{-4}	1.018

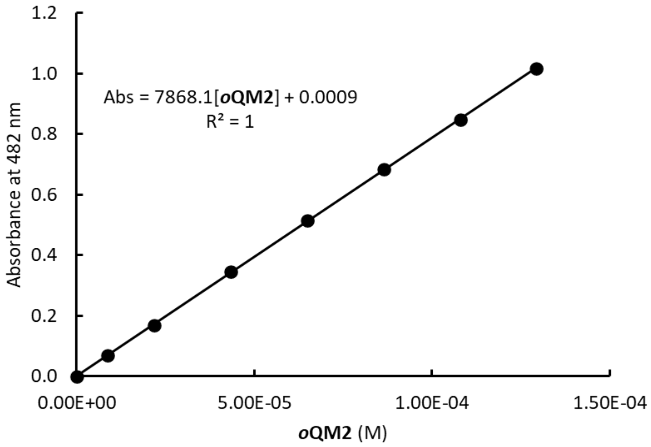

Concentration-dependent absorbance of ***o*****QM2** used to determine *ε* (at λ_{max} = 482 nm, *d* = 0.5 cm).

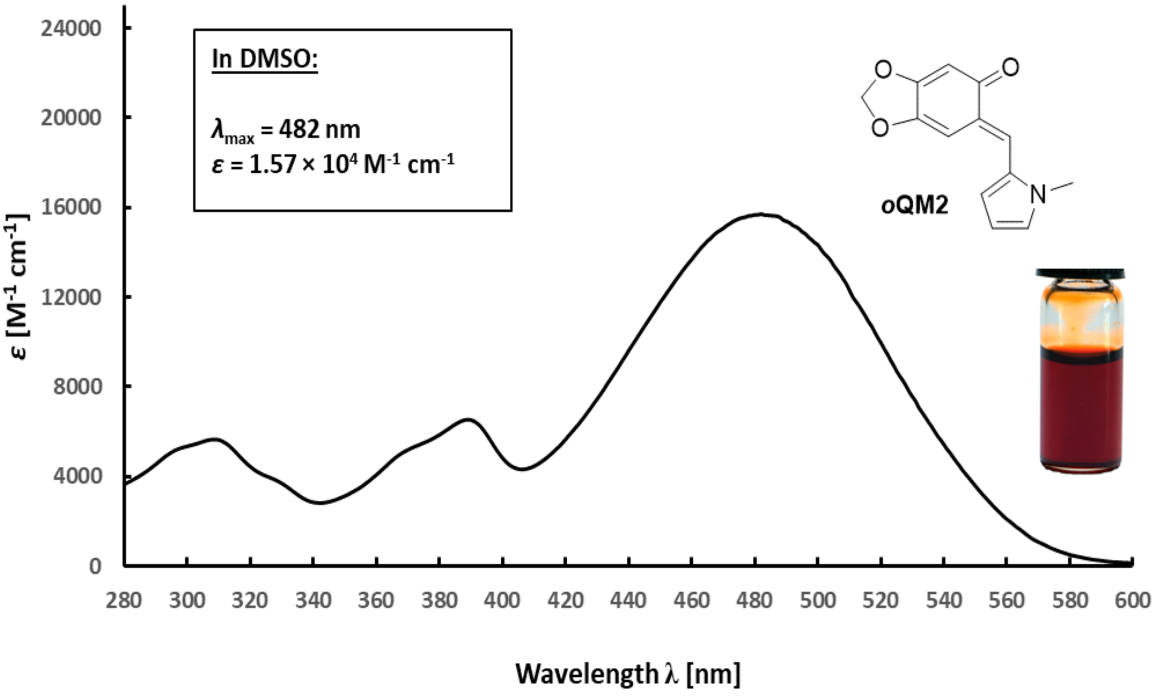

Solutions of ***o*QM3** in DMSO: Concentration-dependent absorbance *A* at λ_{max}.

V (L)	[***o*QM3**] (M)	*A* (485 nm)
0.02400	0	0.000
0.02402	7.45×10^{-6}	0.097
0.02404	1.49×10^{-5}	0.189
0.02406	2.23×10^{-5}	0.280
0.02408	2.97×10^{-5}	0.370
0.02410	3.71×10^{-5}	0.456
0.02412	4.45×10^{-5}	0.540
0.02414	5.19×10^{-5}	0.633
0.02416	5.93×10^{-5}	0.723
0.02418	6.66×10^{-5}	0.811
0.02420	7.40×10^{-5}	0.897

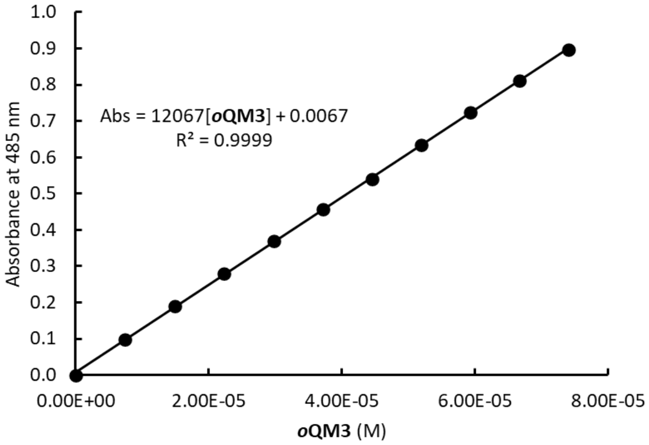

Concentration-dependent absorbance of ***o*QM3** used to determine *ε* (at λ_{max} = 485 nm, *d* = 0.5 cm).

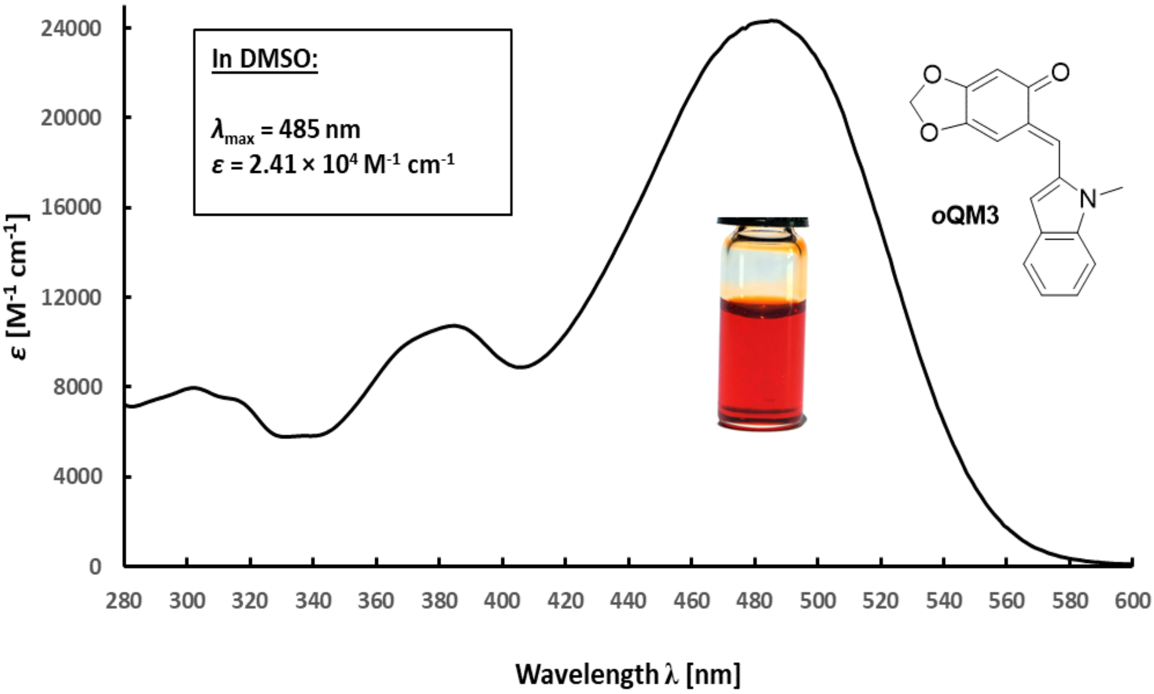

Solutions of ***o*QM4** in DMSO: Concentration-dependent absorbance *A* at λ_{max}.

V (L)	[***o*QM4**] (M)	*A* (452 nm)
0.02400	0	0.000
0.02405	1.98×10^{-5}	0.175
0.02410	3.95×10^{-5}	0.350
0.02415	5.91×10^{-5}	0.515
0.02420	7.86×10^{-5}	0.685
0.02425	9.81×10^{-5}	0.850
0.02430	1.17×10^{-4}	1.015

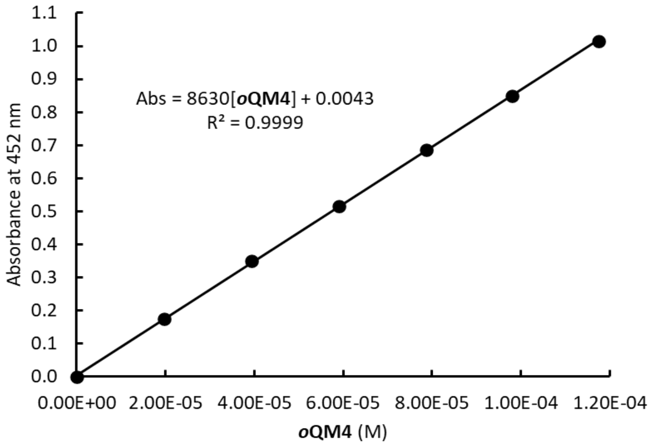

Concentration-dependent absorbance of ***o*QM4** used to determine *ε* (at λ_{max} = 452 nm, *d* = 0.5 cm).

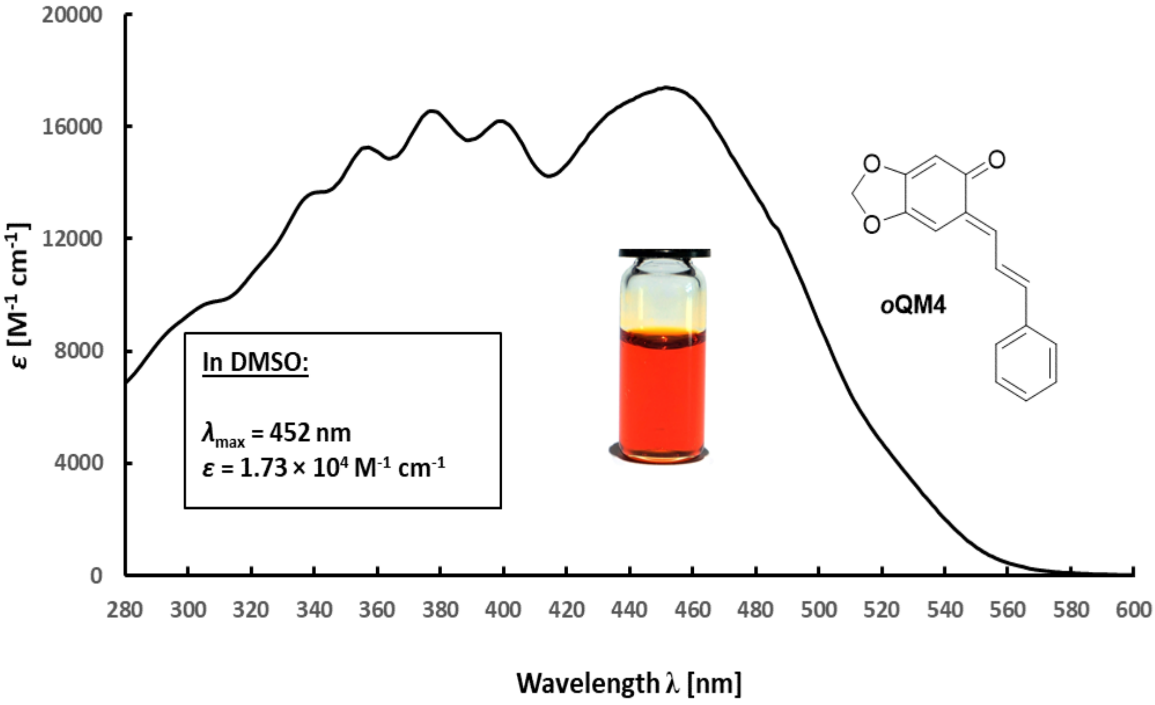

10. Cyclic Voltammetry: Reduction Potentials of *o*QMs

The reduction potentials of *o*QMs (E_p^{red}) were determined in acetonitrile on a CH Instruments 630E electrochemical analyser using a 2 mm diameter platinum working electrode, a platinum wire counter electrode and an Ag wire pseudo-reference electrode applying a scan rate of 0.1 V/s. Cyclic voltammetry measurements were performed in deaerated acetonitrile solutions containing 0.1 M tetra-*n*-butylammonium perchlorate, the *o*QMs ($c \approx 1 \times 10^{-3}$ M), and ferrocene ($c = 7.5 \times 10^{-4}$ M) as an internal standard. The $E_{1/2}$(fc^+/fc in MeCN) = +0.382 V [16] was used to calibrate E_p^{red} (***o*QM** in MeCN) vs SCE.

Only peak potentials E_p^{red} could be determined due to the non-reversibility of the *o*QM reduction.

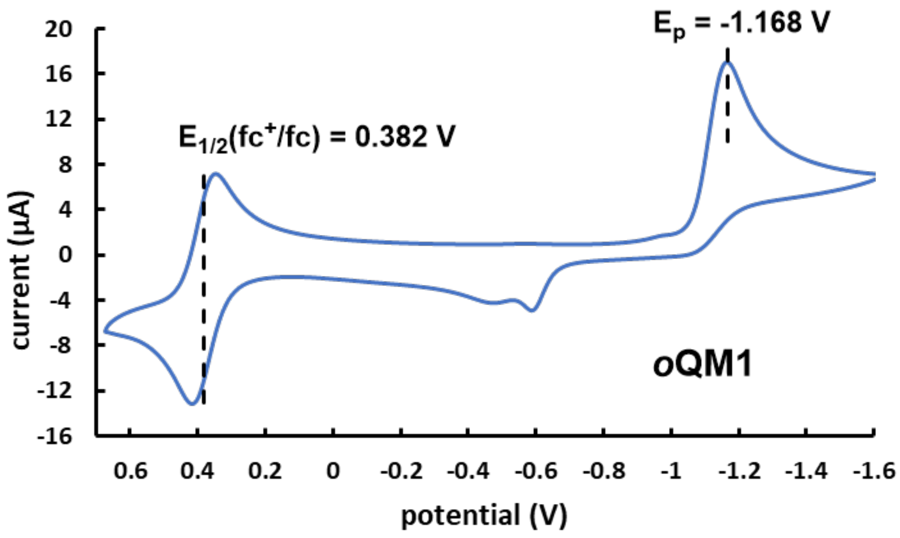

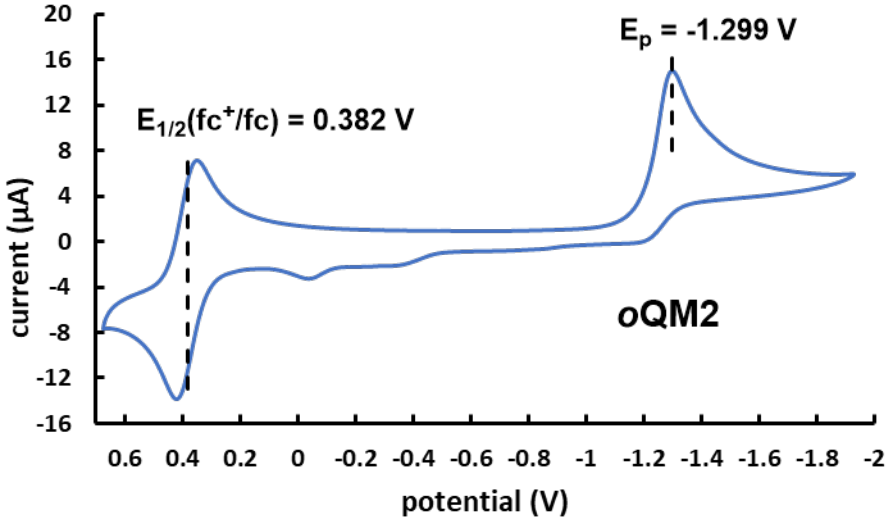

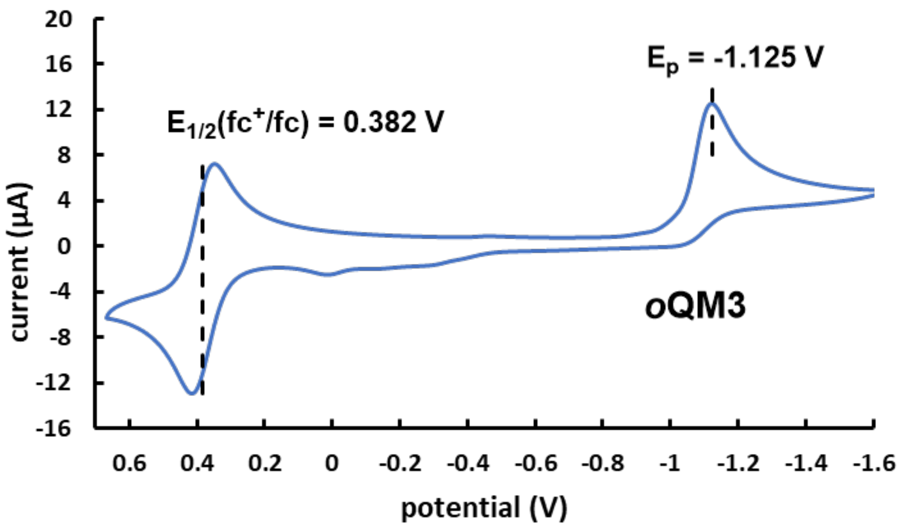

E_p = -1.125 V
$E_{1/2}(fc^+/fc)$ = 0.382 V
*o*QM3
current (µA)
potential (V)
20
16
12
8
4
0
-4
-8
-12
-16
0.6
0.4
0.2
0
-0.2
-0.4
-0.6
-0.8
-1
-1.2
-1.4
-1.6

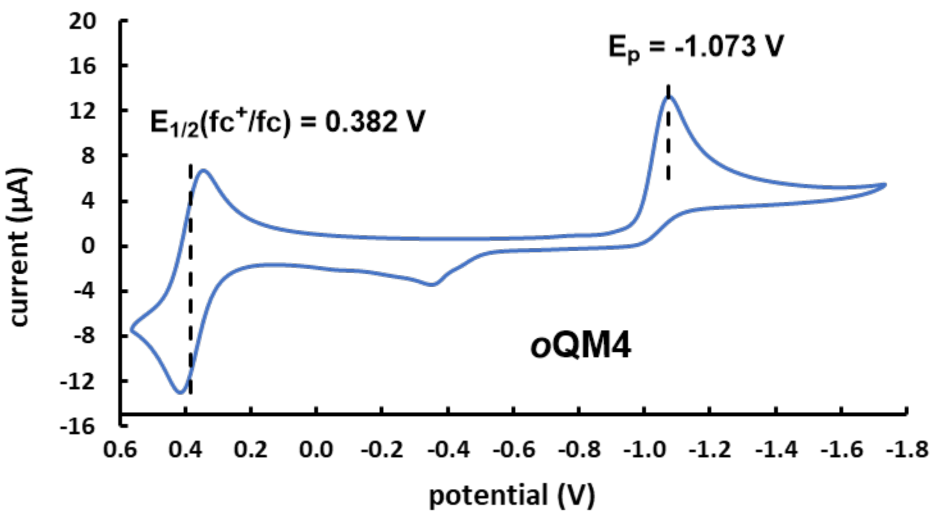

E_p = -1.073 V
$E_{1/2}(fc^+/fc)$ = 0.382 V
*o*QM4
current (µA)
potential (V)
20
16
12
8
4
0
-4
-8
-12
-16
0.6
0.4
0.2
0.0
-0.2
-0.4
-0.6
-0.8
-1.0
-1.2
-1.4
-1.6
-1.8

11. Kinetics of the Reactions of *o*QMs with Carbanions (Reference Nucleophiles)

For kinetic measurements the reactions of *o*QMs with carbanionic nucleophiles were monitored by UV/Vis photometry on AppliedPhotophysics SX.20 stopped-flow (SF) instruments as well as on a conventional J&M TIDAS diode array spectrophotometer, which was controlled by TIDASDAQ3 (v3) software and connected to a Hellma 661.502-QX quartz Suprasil immersion probe (light path d = 5 mm) via fiber optic cables and standard SMA connectors. The temperature (20.0 ± 0.2 °C) was maintained constant by using circulating bath cryostats.

All solutions were prepared by using dry DMSO (ThermoScientific, DMSO 99.7+%, extra dry, over molecular sieve, AcroSeal) and kept under an atmosphere of dry nitrogen. When carbanions were used as the nucleophiles, the kinetic measurements for each *o*QM/nucleophile combination were performed with or without added 18-crown-6 ether (18-c-6) and in some cases with additional CH-acid.

The raw data of kinetic measurements that support the findings of this study are openly available in Open Data LMU at DOI: 10.5282/ubm/data.545.

***o*QM1** + **N1** in DMSO (20 °C, SF method, detection at 445 nm) CG472

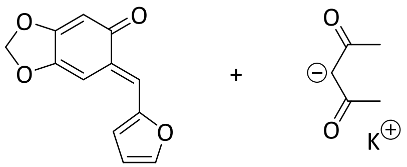

[***o*QM1**]$_0$ (M)	[**N1**]$_0$ (M)	[18-c-6]$_0$ (M)	k_{obs} (s^{-1})
3.70×10^{-5}	1.40×10^{-3}		6.40×10^{-2}
3.70×10^{-5}	2.10×10^{-3}	2.31×10^{-3}	9.41×10^{-2}
3.70×10^{-5}	2.80×10^{-3}		1.20×10^{-1}
3.70×10^{-5}	3.50×10^{-3}	3.85×10^{-3}	1.49×10^{-1}
3.70×10^{-5}	4.20×10^{-3}		1.71×10^{-1}

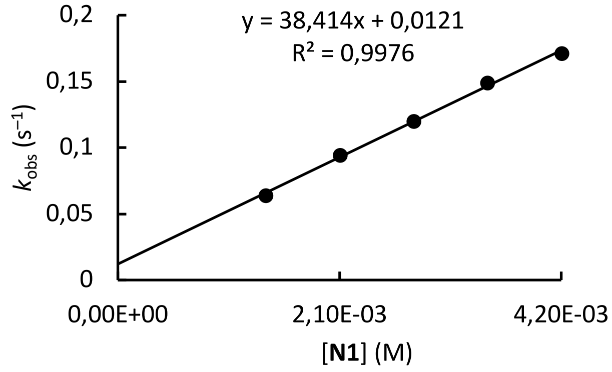

$\mathbf{k_2 = (3.84 \pm 0.11) \times 10^1\ M^{-1}\ s^{-1}}$

***o*QM1** + **N2** in DMSO (20 °C, SF method, detection at 445 nm) CG471

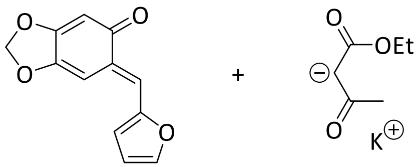

[***o*QM1**]$_0$ (M)	[**N2**]$_0$ (M)	[18-c-6]$_0$ (M)	k_{obs} (s^{-1})
3.83×10^{-5}	7.00×10^{-4}		9.51×10^{-2}
3.83×10^{-5}	1.05×10^{-3}	1.16×10^{-3}	1.37×10^{-1}
3.83×10^{-5}	1.40×10^{-3}		1.74×10^{-1}
3.83×10^{-5}	1.75×10^{-3}	1.93×10^{-3}	2.09×10^{-1}

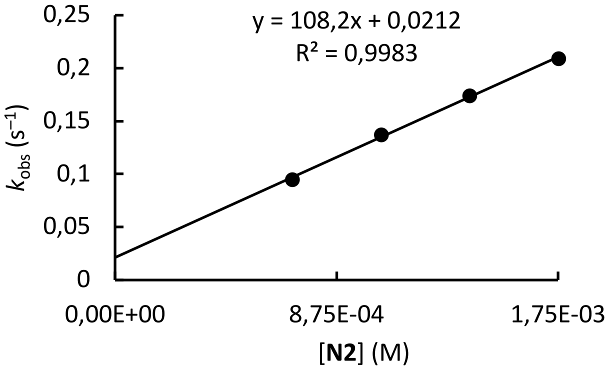

$\mathbf{k_2 = (1.08 \pm 0.03) \times 10^2\ M^{-1}\ s^{-1}}$

***o*QM1** + **N3** in DMSO (20 °C, SF method, detection at 445 nm) CG468

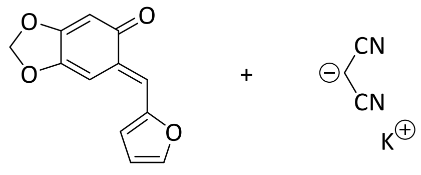

oQM1 **N3**

[***o*QM1**]$_0$ (M)	[**N3**]$_0$ (M)	[18-c-6]$_0$ (M)	k_{obs} (s^{-1})
2.96×10^{-5}	3.50×10^{-4}		1.72×10^{-1}
2.96×10^{-5}	5.25×10^{-4}	5.78×10^{-4}	2.49×10^{-1}
2.96×10^{-5}	7.00×10^{-4}		3.27×10^{-1}
2.96×10^{-5}	8.75×10^{-4}	9.63×10^{-4}	4.04×10^{-1}
2.96×10^{-5}	1.05×10^{-3}		4.77×10^{-1}

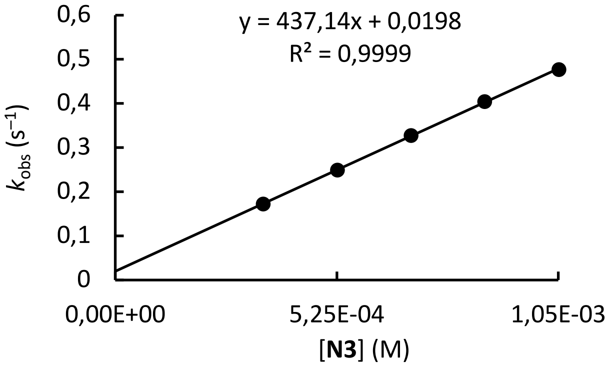

k_2 = (4.37 ± 0.03) × 10^2 M^{-1} s^{-1}

***o*QM1** + **N4** in DMSO (20 °C, SF method, detection at 445 nm) CG469

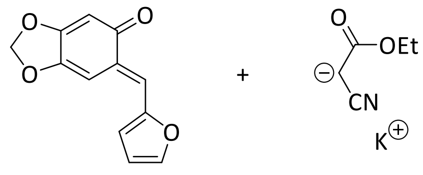

oQM1 **N4**

[***o*QM1**]$_0$ (M)	[**N4**]$_0$ (M)	[18-c-6]$_0$ (M)	k_{obs} (s^{-1})
2.96×10^{-5}	3.50×10^{-4}		1.80×10^{-1}
2.96×10^{-5}	5.25×10^{-4}	5.78×10^{-4}	2.68×10^{-1}
2.96×10^{-5}	7.00×10^{-4}		3.40×10^{-1}
2.96×10^{-5}	8.75×10^{-4}	9.63×10^{-4}	4.16×10^{-1}
2.96×10^{-5}	1.05×10^{-3}		4.82×10^{-1}

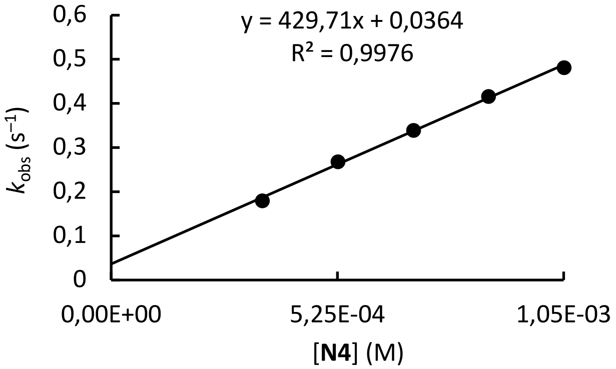

k_2 = (4.30 ± 0.12) × 10^2 M^{-1} s^{-1}

***o*QM1** + **N5** in DMSO (20 °C, SF method, detection at 445 nm) CG470

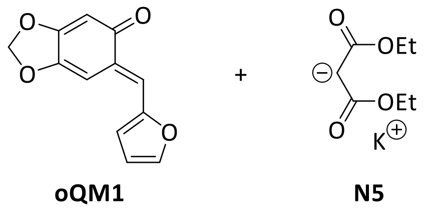

[***o*QM1**]$_0$ (M)	[**N5**]$_0$ (M)	[18-c-6]$_0$ (M)	k_{obs} (s^{-1})
2.96×10^{-5}	3.50×10^{-4}		1.23×10^{-1}
2.96×10^{-5}	5.25×10^{-4}	5.78×10^{-4}	1.81×10^{-1}
2.96×10^{-5}	7.00×10^{-4}		2.32×10^{-1}
2.96×10^{-5}	8.75×10^{-4}	9.63×10^{-4}	2.87×10^{-1}
2.96×10^{-5}	1.05×10^{-3}		3.38×10^{-1}

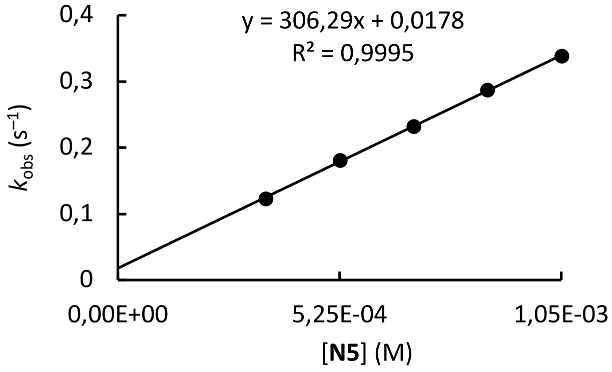

$k_2 = (3.06 \pm 0.04) \times 10^2\ M^{-1}\ s^{-1}$

***o*QM1** + **N7** in DMSO (20 °C, SF method, detection at 445 nm) CG473

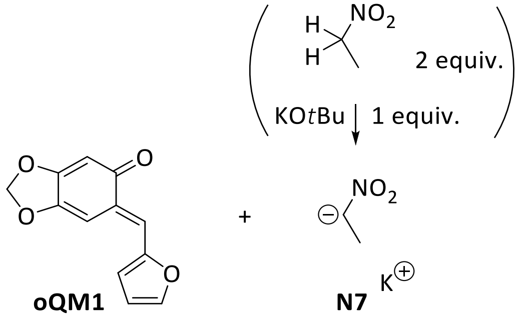

[***o*QM1**]$_0$ (M)	[**N7**]$_0$ (M)	[18-c-6]$_0$ (M)	k_{obs} (s^{-1})
3.46×10^{-5}	7.00×10^{-4}		3.74
3.46×10^{-5}	1.05×10^{-3}	1.16×10^{-3}	5.63
3.46×10^{-5}	1.40×10^{-3}		7.08
3.46×10^{-5}	1.75×10^{-3}	1.93×10^{-3}	9.01

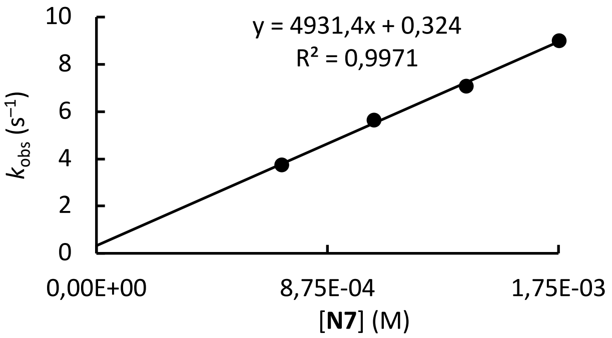

$k_2 = (4.93 \pm 0.19) \times 10^3\ M^{-1}\ s^{-1}$

***o*QM2** + **N1** in DMSO (20 °C, diode array detection at 482 nm) CG518

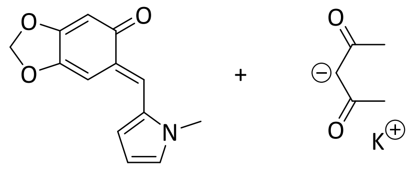

oQM2 **N1**

[***o*QM2**]$_0$ (M)	[**N1**]$_0$ (M)	[18-c-6]$_0$ (M)	k_{obs} (s^{-1})
4.90×10^{-5}	8.87×10^{-4}		6.94×10^{-3}
4.90×10^{-5}	1.32×10^{-3}	1.45×10^{-3}	1.06×10^{-2}
5.10×10^{-5}	1.77×10^{-3}		1.41×10^{-2}
5.10×10^{-5}	2.15×10^{-3}	2.37×10^{-3}	1.74×10^{-2}
4.97×10^{-5}	2.60×10^{-3}		2.04×10^{-2}

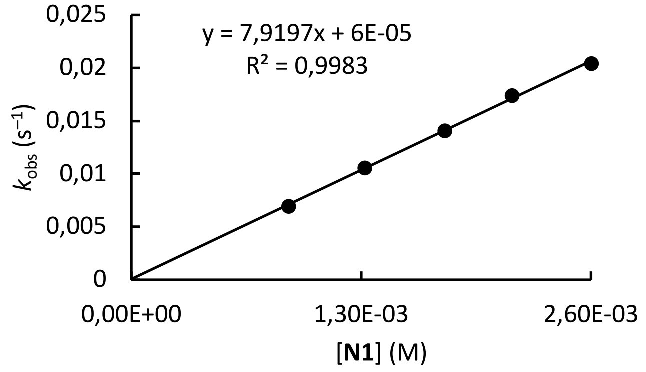

$\boldsymbol{k_2}$ **= (7.92 ± 0.19) M**$^{-1}$ **s**$^{-1}$

***o*QM2** + **N2** in DMSO (20 °C, SF method, detection at 482 nm) CG519

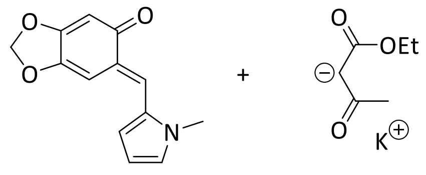

oQM2 **N2**

[***o*QM2**]$_0$ (M)	[**N2**]$_0$ (M)	[18-c-6]$_0$ (M)	k_{obs}[a] (s^{-1})
4.78×10^{-5}	7.50×10^{-4}		1.33×10^{-2}
4.78×10^{-5}	1.13×10^{-3}	1.24×10^{-3}	1.88×10^{-2}
4.78×10^{-5}	1.50×10^{-3}		2.28×10^{-2}
4.78×10^{-5}	1.88×10^{-3}	2.06×10^{-3}	2.84×10^{-2}
4.78×10^{-5}	2.25×10^{-3}		3.25×10^{-2}

[a] Only the first half-life time was used to determine k_{obs}.

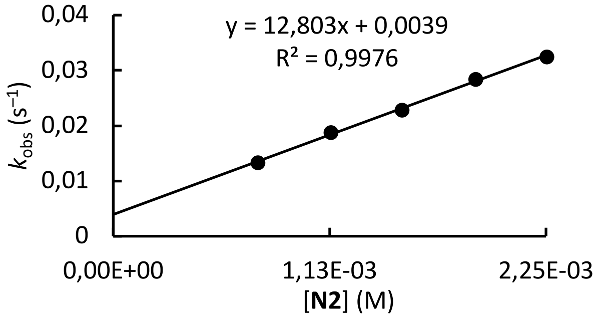

$\boldsymbol{k_2}$ **= (1.28 ± 0.04) × 10**^{1} **M**$^{-1}$ **s**$^{-1}$

***o*QM2** + **N4** in DMSO (20 °C, SF method, detection at 482 nm) CG521

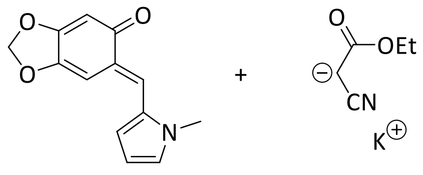

oQM2 **N4**

[***o*QM2**]$_0$ (M)	[**N4**]$_0$ (M)	[18-c-6]$_0$ (M)	k_{obs}[a] (s^{-1})
4.33×10^{-5}	3.75×10^{-4}		3.85×10^{-2}
4.33×10^{-5}	5.65×10^{-4}	6.19×10^{-4}	5.63×10^{-2}
4.33×10^{-5}	7.50×10^{-4}		7.49×10^{-2}
4.33×10^{-5}	9.40×10^{-4}	1.03×10^{-3}	8.98×10^{-2}
4.33×10^{-5}	1.13×10^{-3}		1.06×10^{-1}

[a] Only the first half-life time was used to determine k_{obs}.

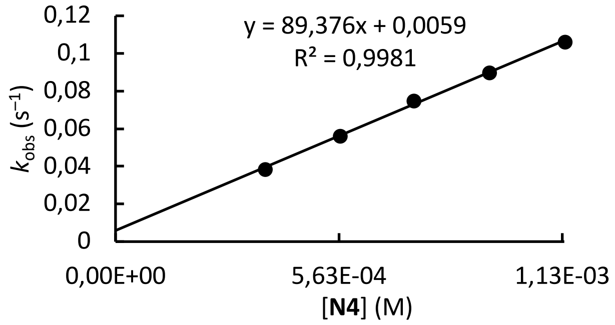

k_2 **= (8.94 ± 0.23) × 10¹ M⁻¹ s⁻¹**

***o*QM2** + **N6** in DMSO (20 °C, SF method, detection at 482 nm) CG520

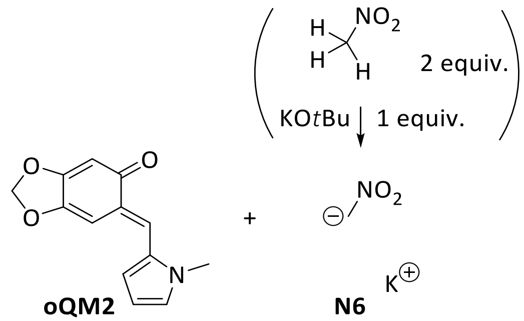

oQM2 **N6**

[***o*QM2**]$_0$ (M)	[**N6**]$_0$ (M)	[18-c-6]$_0$ (M)	k_{obs} (s^{-1})
4.90×10^{-5}	3.75×10^{-4}		6.72×10^{-2}
4.90×10^{-5}	5.65×10^{-4}	6.19×10^{-4}	7.80×10^{-2}
4.90×10^{-5}	7.50×10^{-4}		9.29×10^{-2}
4.90×10^{-5}	9.40×10^{-4}	1.03×10^{-3}	1.12×10^{-1}
4.90×10^{-5}	1.13×10^{-3}		1.26×10^{-1}

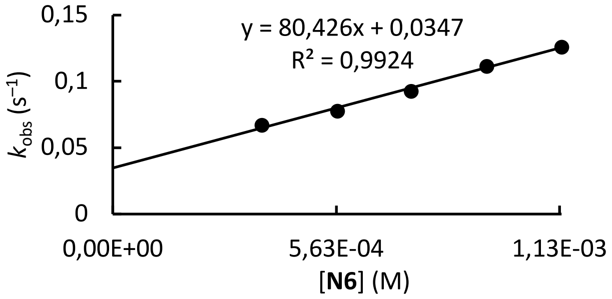

k_2 **= (8.04 ± 0.41) × 10¹ M⁻¹ s⁻¹**

oQM2 + **N7** in DMSO (20 °C, SF method, detection at 482 nm) CG517

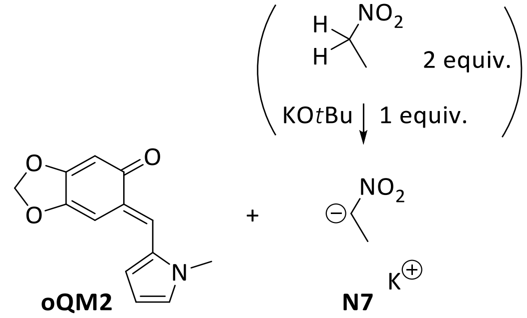

[**oQM2**]$_0$ (M)	[**N7**]$_0$ (M)	[18-c-6]$_0$ (M)	k_{obs} (s^{-1})
3.72×10^{-5}	3.75×10^{-4}		1.94×10^{-1}
3.72×10^{-5}	5.65×10^{-4}	6.19×10^{-4}	2.41×10^{-2}
3.72×10^{-5}	7.50×10^{-4}		2.82×10^{-2}
3.72×10^{-5}	9.40×10^{-4}	1.03×10^{-3}	3.62×10^{-1}
3.72×10^{-5}	1.13×10^{-3}		4.18×10^{-1}

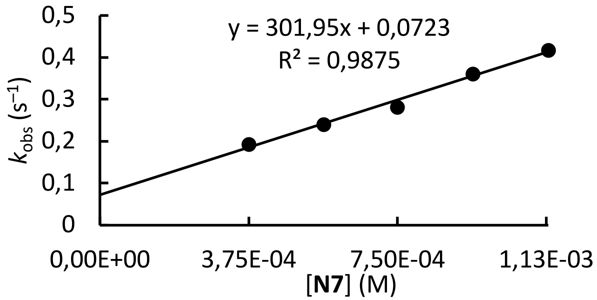

$k_2 = (3.02 \pm 0.20) \times 10^2\ M^{-1}\ s^{-1}$

oQM3 + **N1** in DMSO (20 °C, diode array detection at 485 nm) CG514

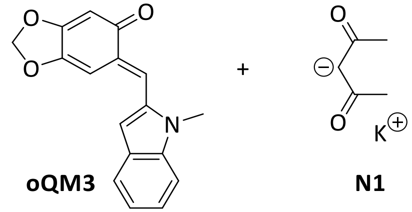

[**oQM3**]$_0$ (M)	[**N1**]$_0$ (M)	[18-c-6]$_0$ (M)	k_{obs} (s^{-1})
2.46×10^{-5}	4.97×10^{-4}		3.26×10^{-2}
2.34×10^{-5}	7.39×10^{-4}	8.12×10^{-4}	4.20×10^{-2}
2.50×10^{-5}	1.00×10^{-3}		5.98×10^{-2}
2.30×10^{-5}	1.22×10^{-3}	1.34×10^{-3}	7.36×10^{-2}
2.42×10^{-5}	1.48×10^{-3}		9.02×10^{-2}

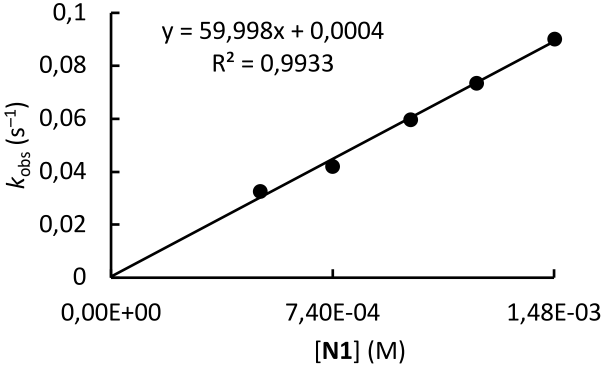

$k_2 = (6.00 \pm 0.28) \times 10^1\ M^{-1}\ s^{-1}$

***o*QM3** + **N2** in DMSO (20 °C, SF method, detection at 485 nm) CG513

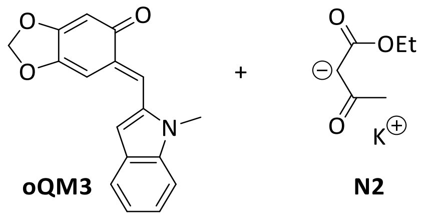

[**oQM3**]$_0$ (M)	[**N2**]$_0$ (M)	[18-c-6]$_0$ (M)	k_{obs} (s^{-1})
2.50×10^{-5}	5.00×10^{-4}		9.53×10^{-2}
2.17×10^{-5}	7.50×10^{-4}	8.25×10^{-4}	1.31×10^{-1}
2.01×10^{-5}	1.00×10^{-3}		1.69×10^{-1}
1.84×10^{-5}	1.25×10^{-3}	1.38×10^{-3}	2.15×10^{-1}
1.76×10^{-5}	1.50×10^{-3}		2.49×10^{-1}

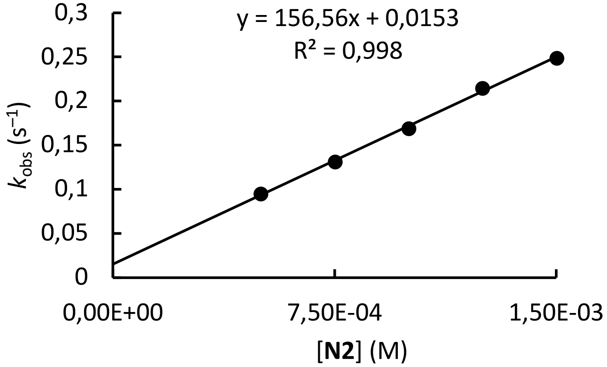

$\boldsymbol{k_2 = (1.57 \pm 0.04) \times 10^2\ M^{-1}\ s^{-1}}$

***o*QM3** + **N3** in DMSO (20 °C, SF method, detection at 485 nm) CG508

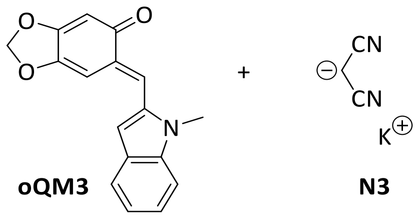

[***o*QM3**]$_0$ (M)	[**N3**]$_0$ (M)	[18-c-6]$_0$ (M)	k_{obs} (s^{-1})
2.09×10^{-5}	2.50×10^{-4}		2.62×10^{-1}
1.97×10^{-5}	3.75×10^{-4}	4.13×10^{-4}	3.59×10^{-1}
1.84×10^{-5}	5.00×10^{-4}		4.36×10^{-1}
1.76×10^{-5}	6.25×10^{-4}	6.88×10^{-4}	5.14×10^{-1}
1.62×10^{-5}	7.50×10^{-4}		5.91×10^{-1}

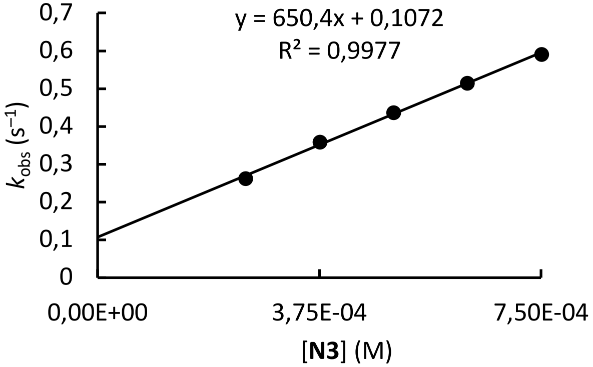

$\boldsymbol{k_2 = (6.50 \pm 0.18) \times 10^2\ M^{-1}\ s^{-1}}$

***o*QM3** + **N5** in DMSO (20 °C, SF method, detection at 485 nm) CG507

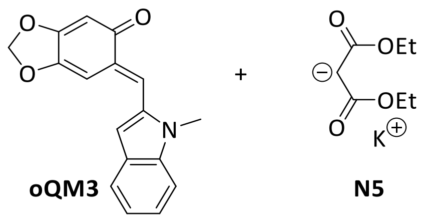

[***o*QM3**]$_0$ (M)	[**N5**]$_0$ (M)	[18-c-6]$_0$ (M)	k_{obs} (s^{-1})
2.58×10^{-5}	2.50×10^{-4}		1.73×10^{-1}
2.46×10^{-5}	3.75×10^{-4}	4.13×10^{-4}	2.44×10^{-1}
2.34×10^{-5}	5.00×10^{-4}		3.06×10^{-1}
2.17×10^{-5}	6.25×10^{-4}	6.88×10^{-4}	3.56×10^{-1}

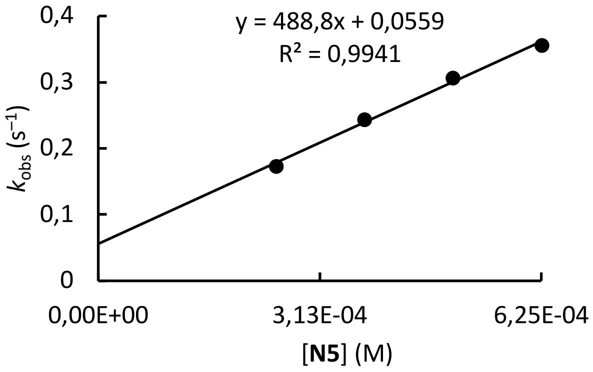

$\boldsymbol{k_2 = (4.89 \pm 0.27) \times 10^2\ M^{-1}\ s^{-1}}$

***o*QM3** + **N7** in DMSO (20 °C, SF method, detection at 485 nm) CG512

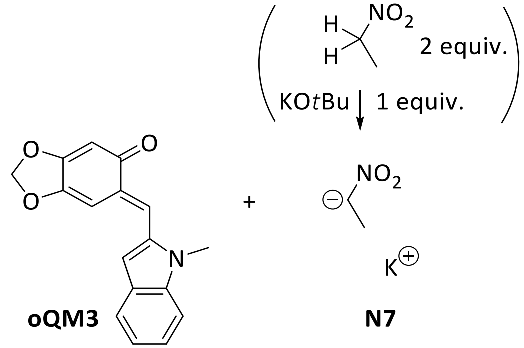

[***o*QM3**]$_0$ (M)	[**N7**]$_0$ (M)	[18-c-6]$_0$ (M)	k_{obs} (s^{-1})
2.42×10^{-5}	2.50×10^{-4}		9.85×10^{-1}
2.13×10^{-5}	3.75×10^{-4}	4.13×10^{-4}	1.47
1.93×10^{-5}	5.00×10^{-4}		1.94
1.68×10^{-5}	6.25×10^{-4}	6.88×10^{-4}	2.42

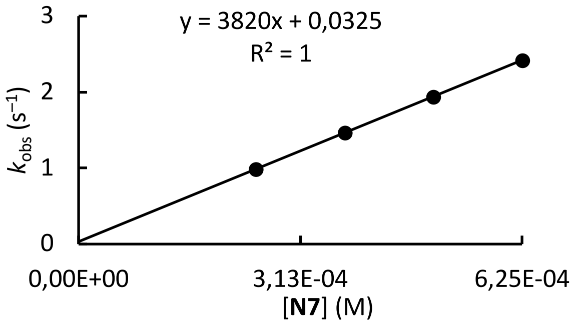

$\boldsymbol{k_2 = (3.82 \pm 0.02) \times 10^3\ M^{-1}\ s^{-1}}$

***o*QM4** + **N2** in DMSO (20 °C, SF method, detection at 452 nm) CG378

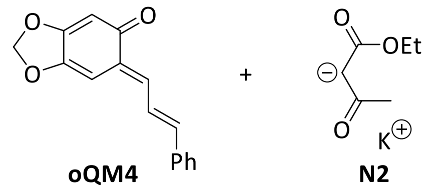

oQM4 **N2**

[***o*QM4**]$_0$ (M)	[**N2**]$_0$[a] (M)	[18-c-6]$_0$ (M)	k_{obs} (s^{-1})
6.55×10^{-5}	3.50×10^{-3}		2.32×10^{-1}
5.86×10^{-5}	5.25×10^{-3}	5.78×10^{-3}	3.55×10^{-1}
5.06×10^{-5}	7.00×10^{-3}		4.38×10^{-1}
4.60×10^{-5}	8.75×10^{-3}	9.63×10^{-3}	5.70×10^{-1}

[a] Additionally, the reaction mixtures contained an equimolar amount of the corresponding CH-acid.

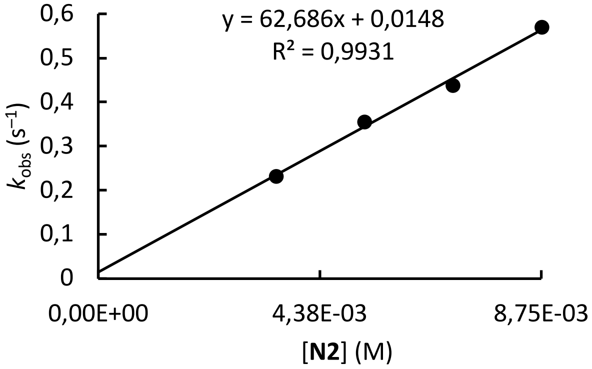

$\mathbf{k_2 = (6.27 \pm 0.37) \times 10^1\ M^{-1}\ s^{-1}}$

***o*QM4** + **N3** in DMSO (20 °C, SF method, detection at 452 nm) CG377

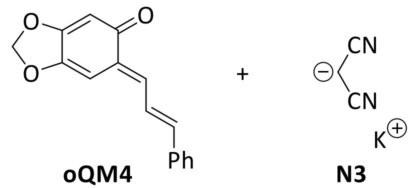

oQM4 **N3**

[***o*QM4**]$_0$ (M)	[**N3**]$_0$[a] (M)	[18-c-6]$_0$ (M)	k_{obs} (s^{-1})
5.98×10^{-5}	1.40×10^{-3}		3.37×10^{-1}
5.23×10^{-5}	2.10×10^{-3}	2.31×10^{-3}	5.02×10^{-1}
4.43×10^{-5}	2.80×10^{-3}		6.75×10^{-1}
3.85×10^{-5}	3.50×10^{-3}	3.85×10^{-3}	8.34×10^{-1}
3.33×10^{-5}	4.20×10^{-3}		1.01

[a] Additionally, the reaction mixtures contained an equimolar amount of the corresponding CH-acid.

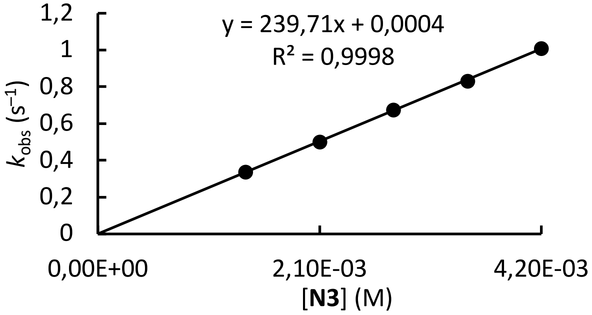

$\mathbf{k_2 = (2.40 \pm 0.02) \times 10^2\ M^{-1}\ s^{-1}}$

***o*QM4** + **N4** in DMSO (20 °C, SF method, detection at 452 nm) CG376

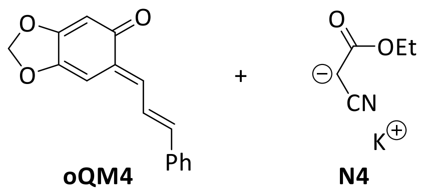

[***o*QM4**]$_0$ (M)	[**N4**]$_0$[a] (M)	[18-c-6]$_0$ (M)	k_{obs} (s^{-1})
3.62 × 10^{-5}	2.80 × 10^{-3}		8.30 × 10^{-1}
3.40 × 10^{-5}	4.20 × 10^{-3}	4.62 × 10^{-3}	1.24
3.05 × 10^{-5}	5.60 × 10^{-3}		1.62
2.70 × 10^{-5}	7.00 × 10^{-3}	7.70 × 10^{-3}	2.03
2.41 × 10^{-5}	8.40 × 10^{-3}		2.45

[a] Additionally, the reaction mixtures contained an equimolar amount of the corresponding CH-acid.

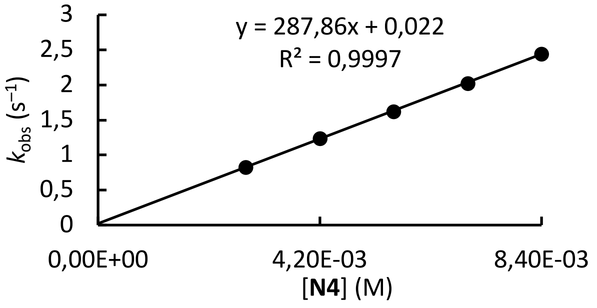

$\boldsymbol{k_2}$ **= (2.88 ± 0.03) × 10^2 M^{-1} s^{-1}**

***o*QM4** + **N5** in DMSO (20 °C, SF method, detection at 452 nm) CG379

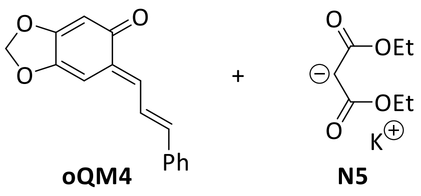

[***o*QM4**]$_0$ (M)	[**N5**]$_0$[a] (M)	[18-c-6]$_0$ (M)	k_{obs} (s^{-1})
3.10 × 10^{-5}	1.05 × 10^{-3}		3.50 × 10^{-1}
2.36 × 10^{-5}	1.58 × 10^{-3}	1.73 × 10^{-3}	5.45 × 10^{-1}
1.72 × 10^{-5}	2.10 × 10^{-3}		6.61 × 10^{-1}
1.26 × 10^{-5}	2.63 × 10^{-3}	2.89 × 10^{-3}	8.94 × 10^{-1}

[a] Additionally, the reaction mixtures contained an equimolar amount of the corresponding CH-acid.

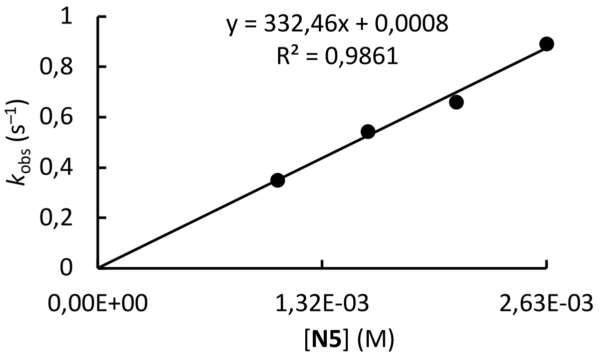

$\boldsymbol{k_2}$ **= (3.32 ± 0.28) × 10^2 M^{-1} s^{-1}**

***o*QM4** + **N7** in DMSO (20 °C, SF method, detection at 452 nm) CG375

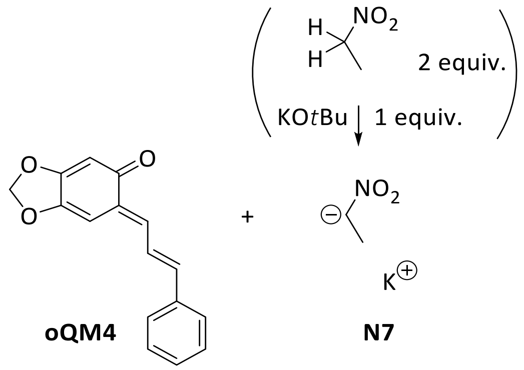

[***o*QM4**]$_0$ (M)	[**N7**]$_0$ (M)	[18-c-6]$_0$ (M)	k_{obs} (s^{-1})
3.68×10^{-5}	1.40×10^{-3}		5.23
3.40×10^{-5}	2.10×10^{-3}	2.31×10^{-3}	8.46
3.16×10^{-5}	2.80×10^{-3}		11.0
2.87×10^{-5}	3.50×10^{-3}	3.85×10^{-3}	14.6

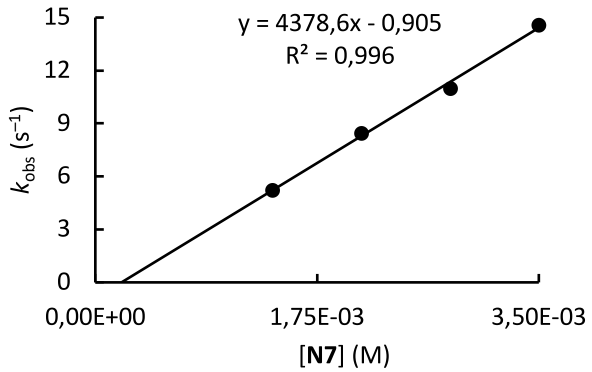

$\boldsymbol{k_2 = (4.38 \pm 0.20) \times 10^3\ M^{-1}\ s^{-1}}$

12. Products of Reactions of *o*QMs with C-Centered Nucleophiles (GP7)

General Procedure (GP7):

A solution of the nucleophile (1.0 to 1.1 equiv.) in d_6-DMSO (1.0 mL) was mixed with *o*QM in a standard GC vial by sonication. Then, the reaction mixture was left for the specified reaction time at room temperature (23 °C). Subsequently, the mixture was quenched with sat. aq. ammonium chloride solution (1 mL), diluted with water (5 mL) and extracted with diethyl ether (4 × 10 mL). The combined organic phases were washed with water (4 × 10 mL) and dried over $MgSO_4$. The solvent was removed under reduced pressure at the rotary evaporator. The residue was further purified either by preparative thin-layer chromatography (prepTLC) or crystallisation and characterised by spectroscopic methods.

6-Amino-8-(furan-2-yl)-8*H*-[1,3]dioxolo[4,5-*g*]chromene-7-carbonitrile (P1) was prepared according to *GP7* (reaction time: 3 h) from ***o*QM1** (20.0 mg, 0.093 mmol) and **N3** (10.6 mg, 0.102 mmol). The crude product was purified by crystallisation from *n*-pentane/CH_2Cl_2 to give **P1** (22.0 mg, 84%) as a brown solid; m.p. 201 °C.

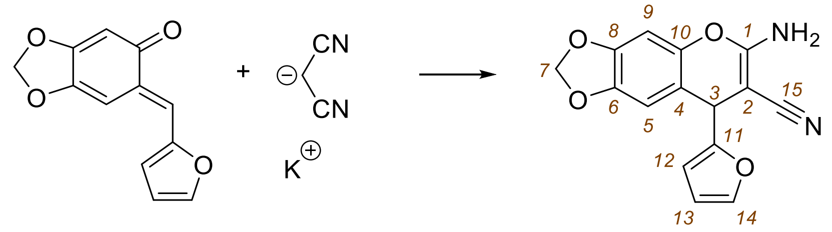

***o*QM1** **N3** **P1** CG585

^{1}H NMR (400 MHz, d_6-DMSO): δ 7.52 (dd, J = 1.9, 1.0 Hz, 1 H, 14-H), 6.93 (s, 2 H, 1-NH_2), 6.67 (s, 1 H, 9-H), 6.66 (s, 1 H, 5-H), 6.35 (dd, J = 3.2, 1.8 Hz, 1 H, 13-H), 6.17–6.16 (m, 1 H, 12-H), 6.02 (d, J = 1.1 Hz, 1 H, 7-H^a), 5.98 (d, J = 1.1 Hz, 1 H, 7-H^b), 4.77 ppm (s, 1 H, 3-H).

^{13}C{^{1}H} NMR (101 MHz, d_6-DMSO): δ 161.1 (C_q, C-1), 156.5 (C_q, C-11), 147.1 (C_q, C-8), 144.0 (C_q, C-6), 143.1 (C_q, C-10), 142.5 (CH, C-14), 120.3 (C_q, C-15), 112.9 (C_q, C-4), 110.3 (CH, C-13), 107.1 (CH, C-5), 105.7 (CH, C-12), 101.7 (CH_2, C-7), 97.8 (CH, C-9), 52.6 (C_q, C-2), 34.4 ppm (CH, C-3).

IR (neat, ATR): $\tilde{\nu}$ 3446, 3339, 2191, 1655, 1629, 1598, 1503, 1488, 1394, 1251, 1146, 1094, 1032, 1011, 926, 841, 756 cm^{-1}.

HRMS (EI): *m*/*z* calcd for $C_{15}H_{10}N_2O_4^{\bullet+}$ [$M^{\bullet+}$]: 282.0635; found: 282.0641.

6-Amino-8-(1-methyl-1*H*-indol-2-yl)-8*H*-[1,3]dioxolo[4,5-*g*]chromene-7-carbonitrile (P2) was prepared according to *GP7* (reaction time: 2 h) from ***o*QM3** (24.0 mg, 0.086 mmol) and **N3** (9.4 mg, 0.090 mmol). The crude product was purified by prepTLC (silica gel, eluent: *n*-pentane:EtOAc = 1:1) to give **P2** (20.0 mg, 67%) as a light brown solid; m.p. 185 °C.

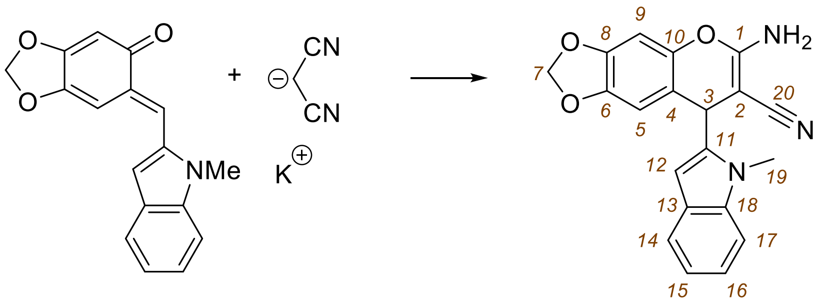

*o*QM3 N3 P2 CG504

R_f (silica, *n*-pentane/EtOAc 1:1, UV) = 0.50

¹H NMR (400 MHz, $CDCl_3$): *δ* 7.58–7.56 (m, 1 H, 14-H), 7.29–7.27 (m, 1 H, 17-H), 7.23–7.18 (m, 1 H, 16-H), 7.12–7.10 (m, 1 H, 15-H), 6.54 (s, 1 H, 9-H), 6.40 (s, 2 H, 5-H and 12-H), 5.93 (m, *J* = 1.3 Hz, 1 H, 7-H^a), 5.90 (m, *J* = 1.4 Hz, 1 H, 7-H^b), 5.06 (s, 1 H, 3-H), 4.70 (s, 2 H, 1-NH_2), 3.59 ppm (s, 3 H, 19-H).

¹³C{¹H} NMR (101 MHz, $CDCl_3$): *δ* 159.6 (C_q, C-1), 147.7 (C_q, C-8), 145.1 (C_q, C-6), 143.1 (C_q, C-10), 140.1 (C_q, C-11), 138.5 (C_q, C-18), 127.4 (C_q, C-13), 121.7 (CH, C-16), 120.6 (CH, C-14), 119.7 (CH, C-15), 119.6 (C_q, C-20), 112.9 (C_q, C-4), 109.1 (CH, C-17), 107.6 (CH, C-5), 102.8 (CH, C-12), 101.9 (CH_2, C-7), 98.1 (CH, C-9), 58.2 (C_q, C-2), 34.5 (CH, C-3), 30.2 ppm (CH_3, C-19).

IR (neat, ATR): $\tilde{\nu}$ 3450, 3338, 2196, 1666, 1603, 1502, 1484, 1407, 1244, 1187, 1147, 1091, 1032, 930, 849, 838, 785, 727 cm^{-1}.

HRMS (EI): *m/z* calcd for $C_{20}H_{15}N_3O_3^{\bullet+}$ [$M^{\bullet+}$]: 345.1108; found: 345.1014.

(*E*)-2-(3-(6-Hydroxybenzo[*d*][1,3]dioxol-5-yl)-1-phenylallyl)malononitrile (P3’) and (*E*)-6-amino-8-styryl-8*H*-[1,3]dioxolo[4,5-*g*]chromene-7-carbonitrile (P3) were obtained according to *GP7* (reaction time: 1 h) from **oQM4** (15.0 mg, 0.059 mmol) and **N3** (6.3 mg, 0.061 mmol). The crude product was purified by prepTLC (silica gel, eluent: *n*-pentane:EtOAc = 7:3) to yield the isomers **P3’** (5.3 mg, 28%) and **P3** (8.2 mg, 44%, known compound[17]).

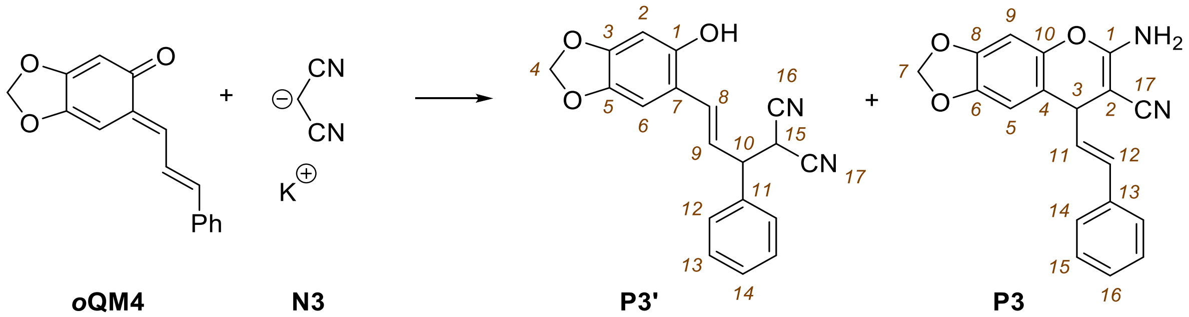

*o*QM4 N3 P3' P3

(*E*)-2-(3-(6-Hydroxybenzo[*d*][1,3]dioxol-5-yl)-1-phenylallyl)malononitrile (P3') (CG380F1)

^{1}H NMR (600 MHz, $CDCl_3$): δ 7.44–7.42 (m, 2 H, 13-H), 7.39–7.38 (m, 3 H, 12-H and 14-H), 6.92 (d, J = 15.7 Hz, 1 H, 8-H), 6.87 (s, 1 H, 6-H), 6.35 (s, 1 H, 2-H), 6.30 (dd, J = 15.7, 8.2 Hz, 1 H, 9-H), 5.92 (s, 2 H, 4-H), 4.94 (s, 1 H, 1-OH), 4.11–4.05 ppm (m, 2 H, 10-H and 15-H).

^{13}C{^{1}H} NMR (151 MHz, $CDCl_3$): δ 148.62 (C_q, C-3), 148.61, (C_q, C-1), 142.3 (C_q, C-5), 136.9 (C_q, C-11), 130.2 (CH, C-8), 129.6 (CH, C-13), 129.0 (CH, C-14), 127.8 (CH, C-12), 122.5 (CH, C-9), 115.4 (C_q, C-7), 111.9 (C_q, C-16 or C-17), 111.9 (C_q, C-16 or C-17), 105.8 (CH, C-6), 101.6 (CH_2, C-4), 98.5 (CH, C-2), 50.5 (CH, C-10), 30.7 ppm (CH, C-15).

IR (neat, ATR): $\tilde{\nu}$ 3436, 2903, 2257, 1627, 1503, 1486, 1445, 1258, 1172, 1037, 934, 909, 762, 731, 700 cm^{-1}.

HRMS (EI): *m*/*z* calcd for $C_{19}H_{14}N_2O_3^{\bullet+}$ [$M^{\bullet+}$]: 318.0999; found: 318.0998.

(*E*)-6-Amino-8-styryl-8*H*-[1,3]dioxolo[4,5-*g*]chromene-7-carbonitrile (P3) (CG380F2)

^{1}H NMR (600 MHz, $CDCl_3$): δ 7.38 (d, J = 7.9 Hz, 2 H, 14-H), 7.30 (t, J = 7.6 Hz, 2 H, 15-H), 7.23 (t, J = 7.3 Hz, 1 H, 16-H), 6.59 (s, 1 H, 5-H), 6.53 (d, J = 15.6 Hz, 1 H, 12-H), 6.49 (s, 1 H, 9-H), 6.09 (dd, J = 15.6, 8.7 Hz, 1 H, 11-H), 5.95 (d, J = 1.5 Hz, 1 H, 7-H^a), 5.94 (d, J = 1.5 Hz, 1 H, 7-H^b), 4.56 (s, 2 H, 1-NH_2), 4.20 ppm (d, J = 8.7 Hz, 1 H, 3-H).

^{13}C{^{1}H} NMR (151 MHz, $CDCl_3$): δ 159.5 (C_q, C-1), 147.5 (C_q, C-8), 144.9 (C_q, C-6), 143.0 (C_q, C-10), 136.5 (C_q, C-13), 131.5 (CH, C-11), 130.8 (CH, C-12), 128.7 (CH, C-15), 127.9 (CH, C-16), 126.8 (CH, C-14), 120.0 (C_q, C-17), 113.7 (C_q, C-4), 107.8 (CH, C-5), 101.9 (CH_2, C-7), 98.3 (CH, C-9), 58.5 (C_q, C-2), 39.1 ppm (CH, C-3).

IR (neat, ATR): $\tilde{\nu}$ 3453, 3348, 2189, 1674, 1602, 1502, 1482, 1406, 1245, 1186, 1149, 1036, 966, 936, 744, 698 cm^{-1}.

HRMS (EI): *m*/*z* calcd for $C_{19}H_{14}N_2O_3^{\bullet+}$ [$M^{\bullet+}$]: 318.0999; found: 318.0998.

13. References

[1] L. M. Bishop, M. Winkler, K. N. Houk, R. G. Bergman and D. Trauner, *Chem. Eur. J.*, 2008, **14**, 5405.

[2] A. Eitzinger, R. J. Mayer, N. Hampel, P. Mayer, M. Waser and A. R. Ofial, *Org. Lett.*, 2020, **22**, 2182.

[3] R. Lucius, R. Loos and H. Mayr, *Angew. Chem., Int. Ed.*, 2002, **41**, 91.

[4] C. M. Young, A. Elmi, D. J. Pascoe, R. K. Morris, C. McLaughlin, A. M. Woods, A. B. Frost, A. Houpliere, K. B. Ling, T. K. Smith, A. M. Z. Slawin, P. H. Willoughby, S. Cockroft, A. D. Smith, *Angew. Chem. Int. Ed.* **2020**, *59*, 3705–3710.

[5] L. S. Vogl, P. Mayer, R. Robiette, M. Waser, *Angew. Chem. Int. Ed.* **2024**, *63*, e202315345.

[6] M. G. Sankar, M. Garcia-Castro, C. Golz, C. Strohmann, K. Kumar, *Angew. Chem. Int. Ed.* **2016**, *55*, 9709–9713.

[7] R. Sinisi, J. Sun, G. C. Fu, Proc. Natl. Acad. Sci. USA 2010, 107, 20652–20654.

[8] O. A. Mukhina, N. N. B. Kumar, T. M. Arisco, R. A. Valiulin, G. A. Metzel and A. G. Kutateladze, *Angew. Chem., Int. Ed.*, **2011**, *50*, 9423.

[9] C. Gross, A. Eitzinger, N. Hampel, P. Mayer and A. R. Ofial, *Chem. Eur. J.*, **2024**, Accepted Article, e202403785 (DOI: 10.1002/chem.202403785).

[10] N. Meisinger, L. Roiser, U. Monkowius, M. Himmelsbach, R. Robiette, M. Waser, *Chem. Eur. J.* **2017**, *23*, 5137–5142.

[11] M. Piringer, M. Hofer, L. S. Vogl, P. Mayer, M. Waser, *Adv. Synth. Catal.* **2024**, *366*, 2115–2122.

[12] P. Chen, K. Wang, W. Guo, X. Liu, Y. Liu, C. Li, *Angew. Chem. Int. Ed.* **2017**, *56*, 3689–3693.

[13] L. A. Nafie, *Nat. Prod. Commun.* **2008**, *3*, 451-466.

[14] Gaussian 16, Revision C.01, M. J. Frisch, G. W. Trucks, H. B. Schlegel, G. E. Scuseria, M. A. Robb, J. R. Cheeseman, G. Scalmani, V. Barone, G. A. Petersson, H. Nakatsuji, X. Li, M. Caricato, A. V. Marenich, J. Bloino, B. G. Janesko, R. Gomperts, B. Mennucci, H. P. Hratchian, J. V. Ortiz, A. F. Izmaylov, J. L. Sonnenberg, D. Williams-Young, F. Ding, F. Lipparini, F. Egidi, J. Goings, B. Peng, A. Petrone, T. Henderson, D. Ranasinghe, V. G. Zakrzewski, J. Gao, N. Rega, G. Zheng, W. Liang, M. Hada, M. Ehara, K. Toyota, R. Fukuda, J. Hasegawa, M. Ishida, T. Nakajima, Y. Honda, O. Kitao, H. Nakai, T. Vreven, K. Throssell, J. A. Montgomery, Jr., J. E. Peralta, F. Ogliaro, M. J. Bearpark, J. J. Heyd, E. N. Brothers, K. N. Kudin, V. N. Staroverov, T. A. Keith, R. Kobayashi, J. Normand, K. Raghavachari, A. P. Rendell, J. C. Burant, S. S. Iyengar, J. Tomasi, M. Cossi, J. M. Millam, M. Klene, C. Adamo, R. Cammi, J. W. Ochterski, R. L. Martin, K. Morokuma, O. Farkas, J. B. Foresman, and D. J. Fox, Gaussian, Inc., Wallingford CT, 2019

[15] M. K. Kesharwani, B. Brauer, J. M. L. Martin, *J. Phys. Chem. A* **2015**, *119*, 1701-1714

[16] J. Ruiz Aranzaes, M.-C. Daniel and D. Astruc, *Can. J. Chem.*, 2006, **84**, 288.

[17] A. Adili, Z.-L. Tao, D.-F. Chen and Z.-Y. Han, *Org. Biomol. Chem.*, **2015**, *13*, 2247.

14. Appendix: NMR Spectra and HPLC chromatograms

14.1 NMR spectra of the novel starting materials

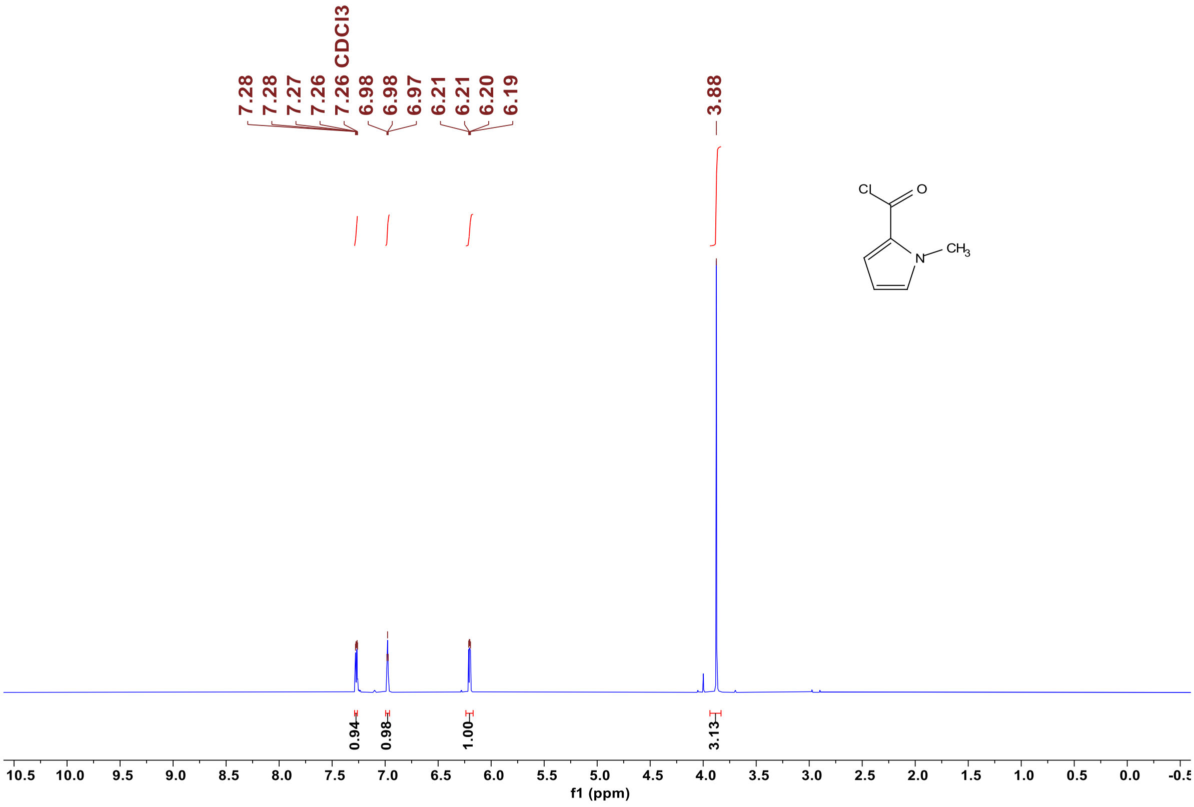

Fig. S16 ^{1}H NMR spectrum of 1-methyl-1*H*-pyrrole-2-carbonyl chloride in $CDCl_3$ (400 MHz) CG482

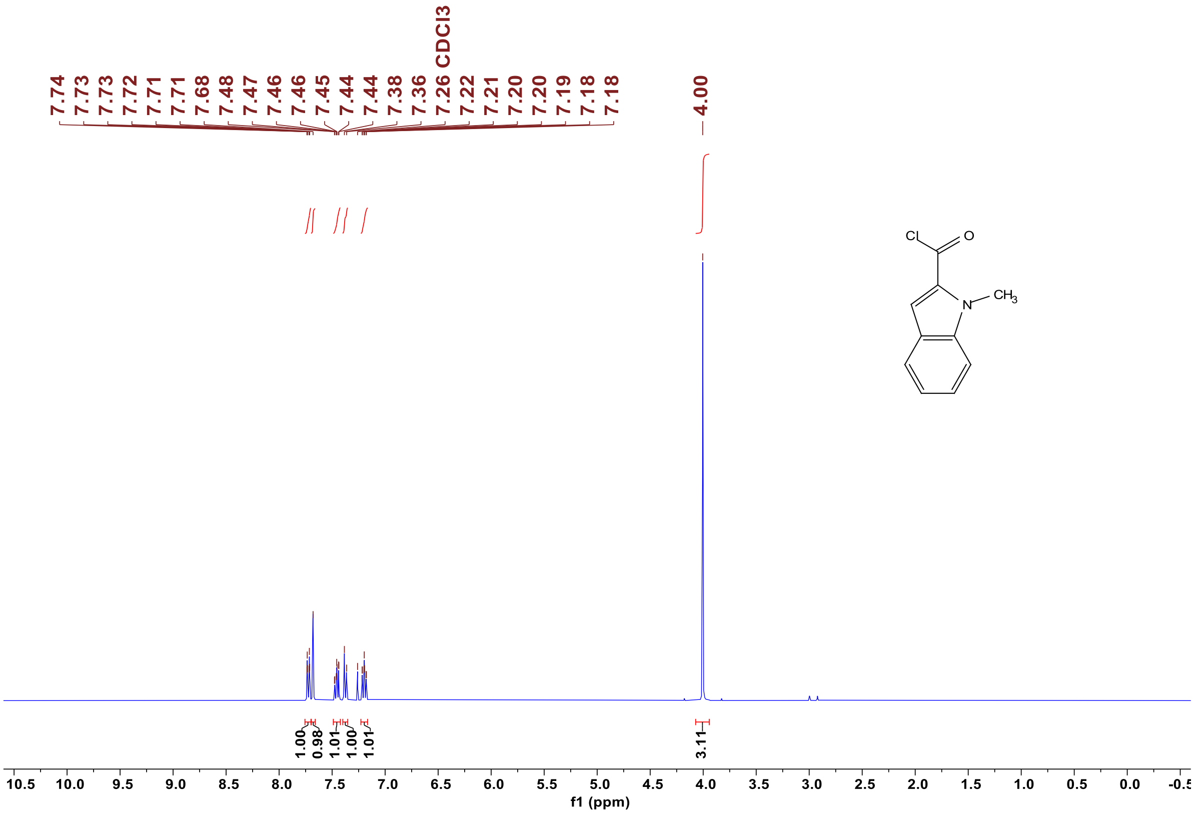

Fig. S17 ^{1}H NMR spectrum of 1-methyl-1*H*-indole-2-carbonyl chloride in $CDCl_3$ (400 MHz) CG490

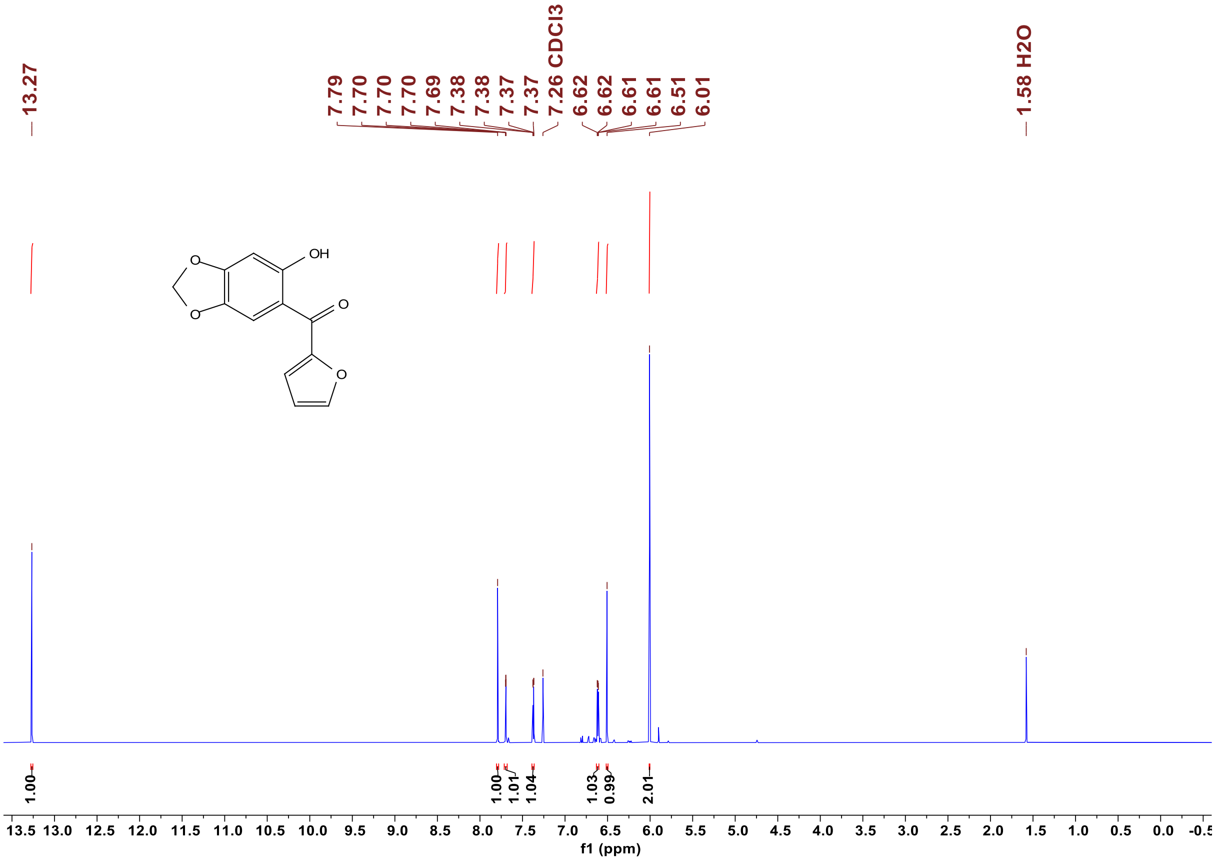

Fig. S18 ^{1}H NMR spectrum of **DAK1** in $CDCl_3$ (400 MHz) CG454_1

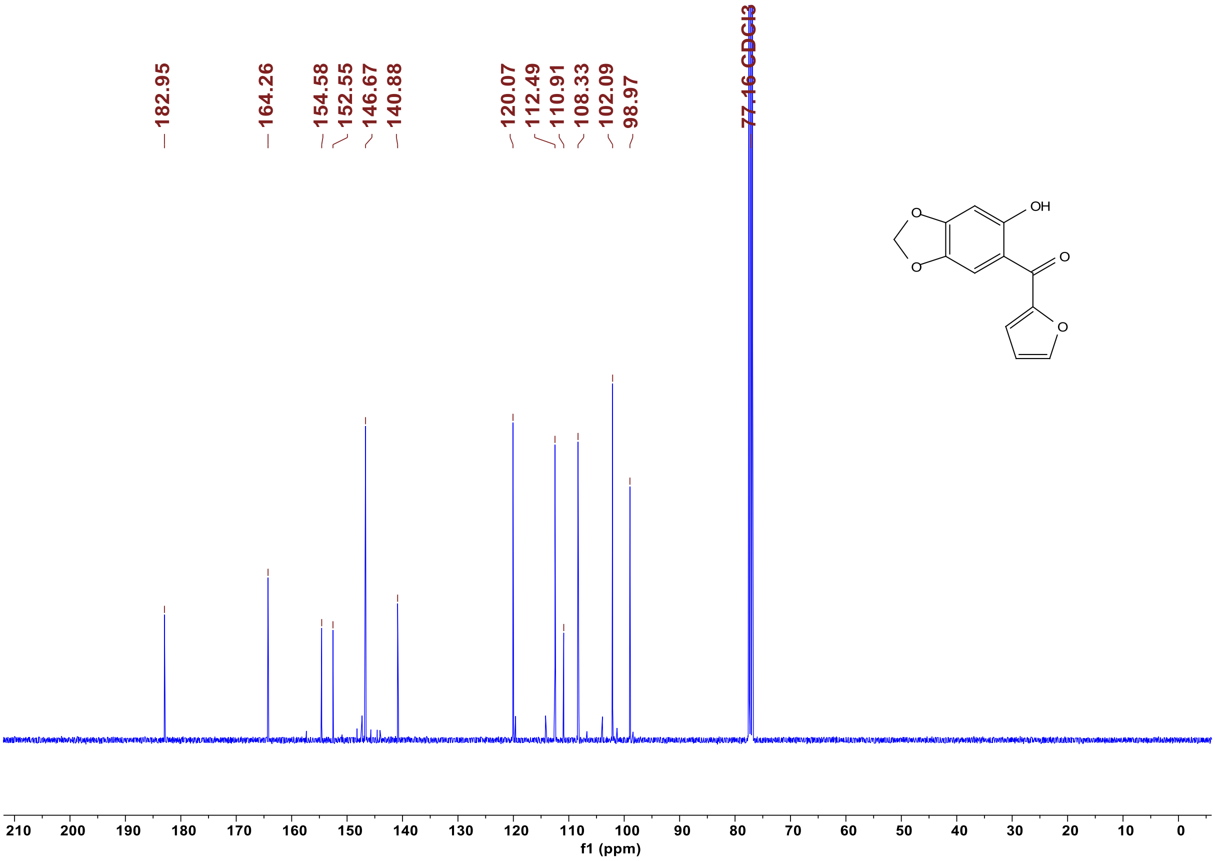

Fig. S19 $^{13}C\{^1H\}$ NMR spectrum of **DAK1** in $CDCl_3$ (101 MHz) CG454_1

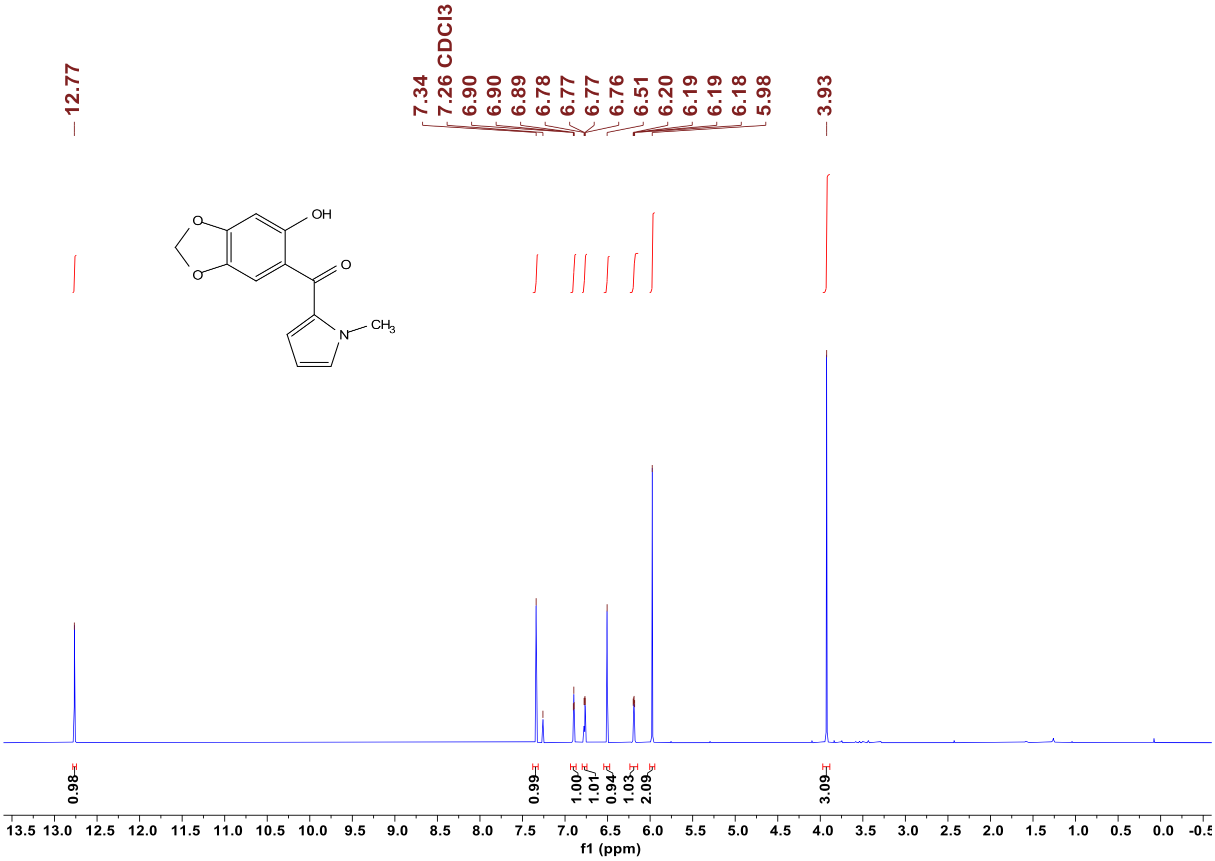

Fig. S9 ¹H NMR spectrum of **DAK2** in $CDCl_3$ (400 MHz) CG484

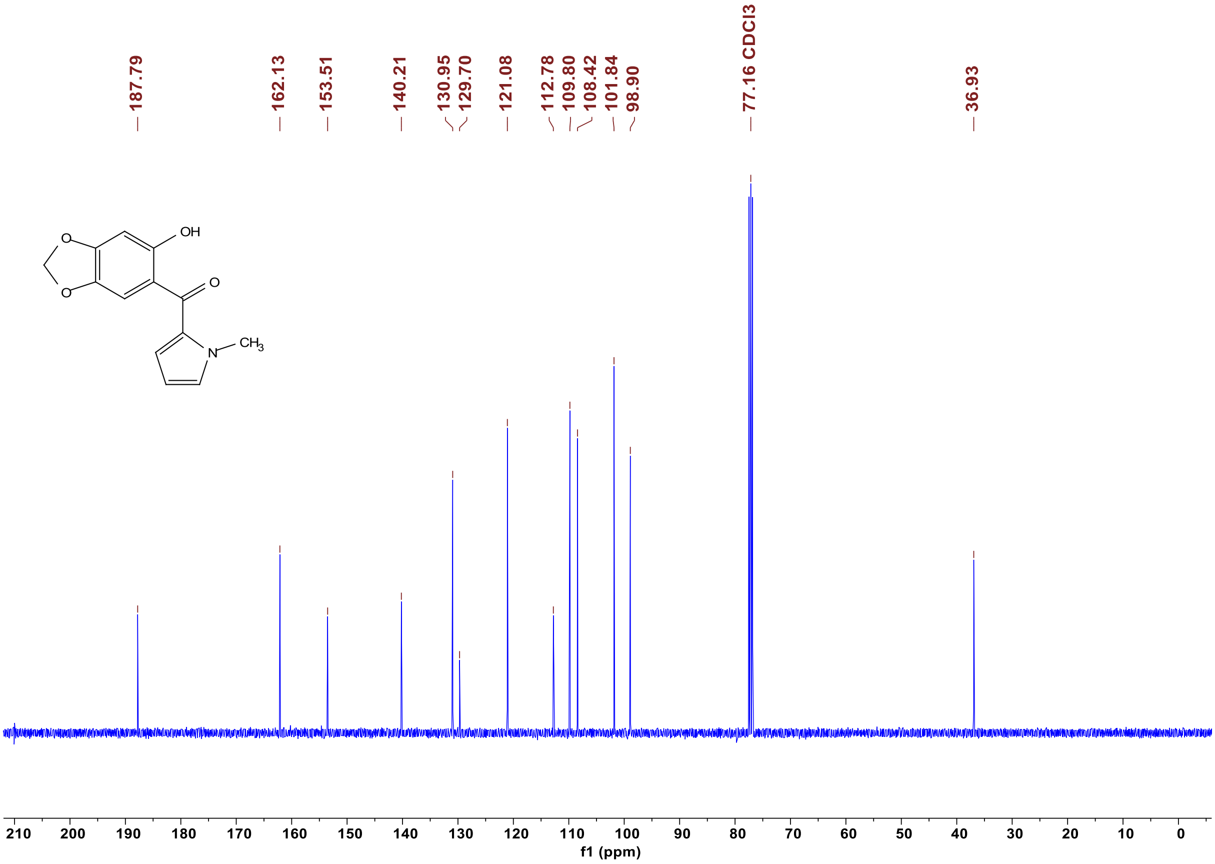

Fig. S20 $^{13}C\{^1H\}$ NMR spectrum of **DAK2** in $CDCl_3$ (101 MHz) CG484

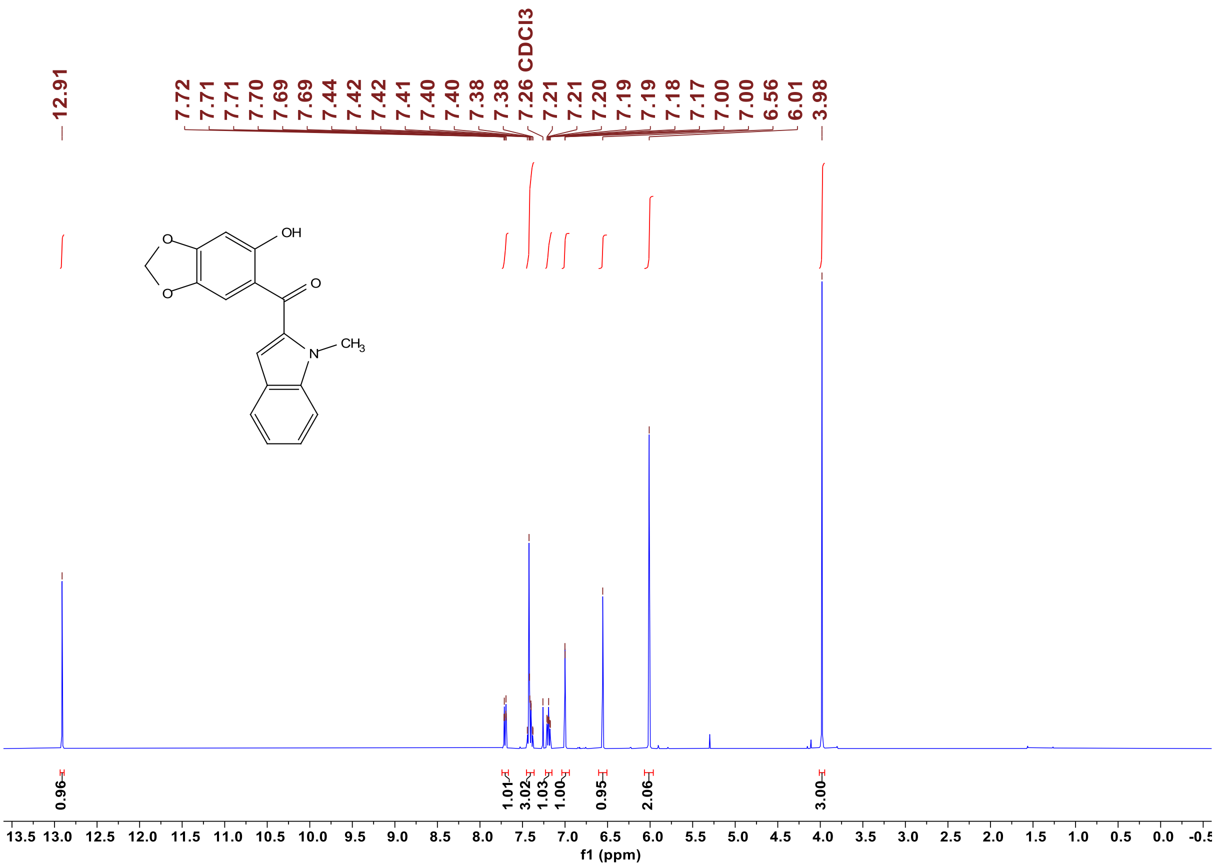

Fig. S21 ^{1}H NMR spectrum of **DAK3** in $CDCl_3$ (400 MHz) CG491

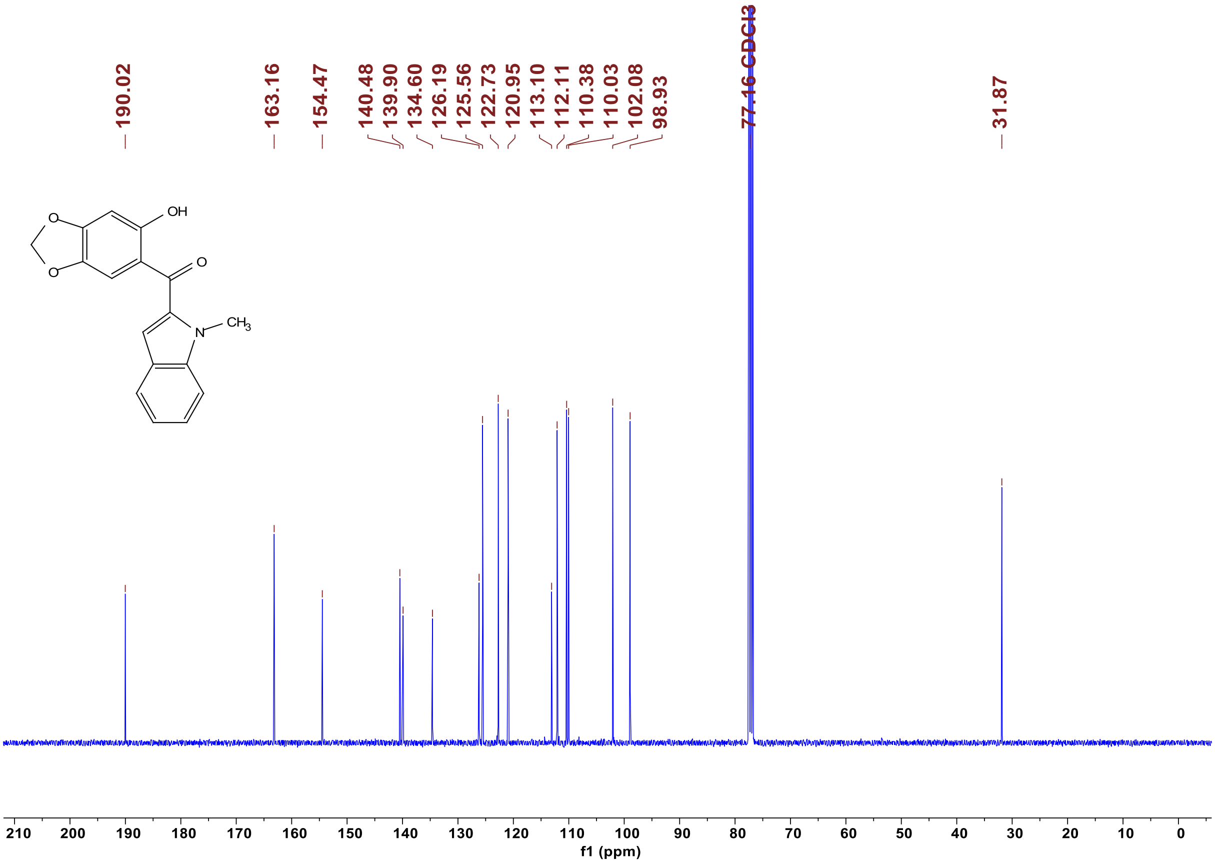

Fig. S22 ^{13}C{^{1}H} NMR spectrum of **DAK3** in $CDCl_3$ (101 MHz) CG491

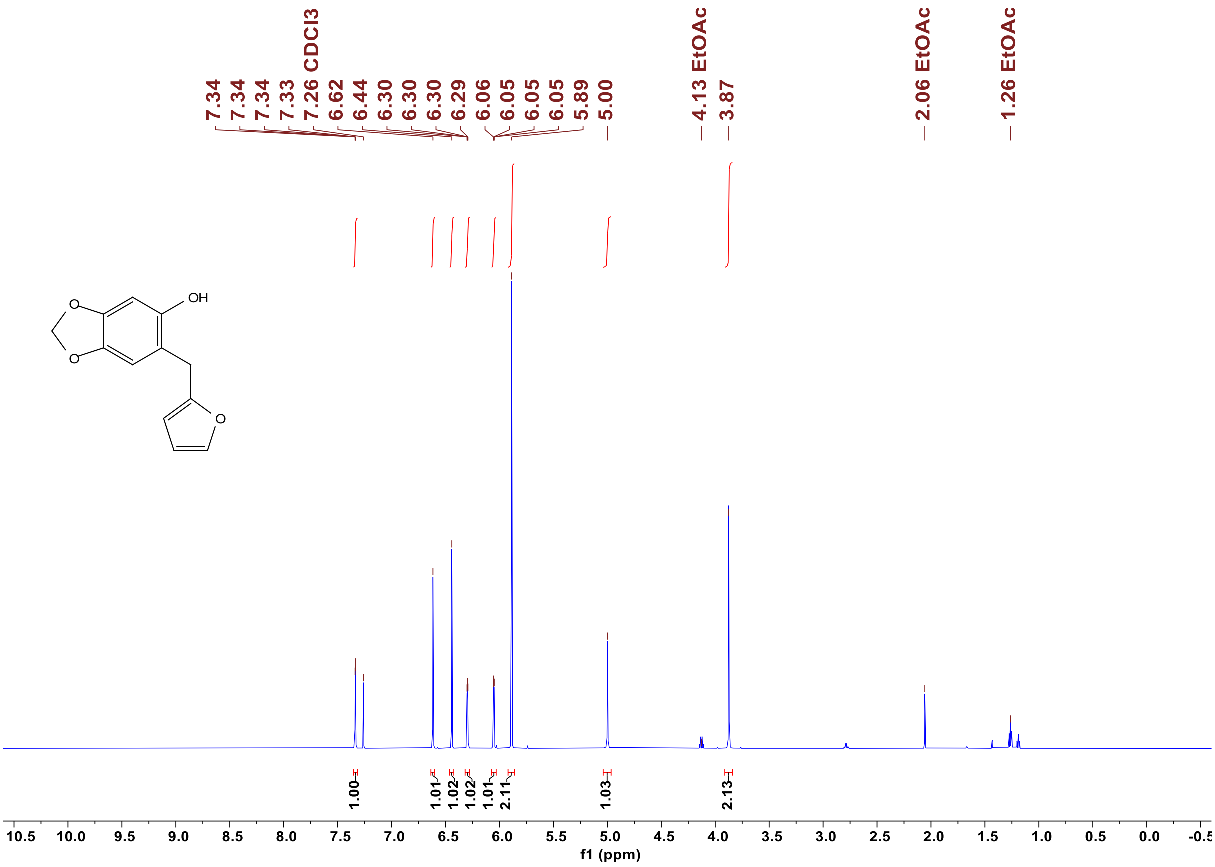

Fig. S23 ^{1}H NMR spectrum of **DAM1** in $CDCl_3$ (600 MHz) CG457

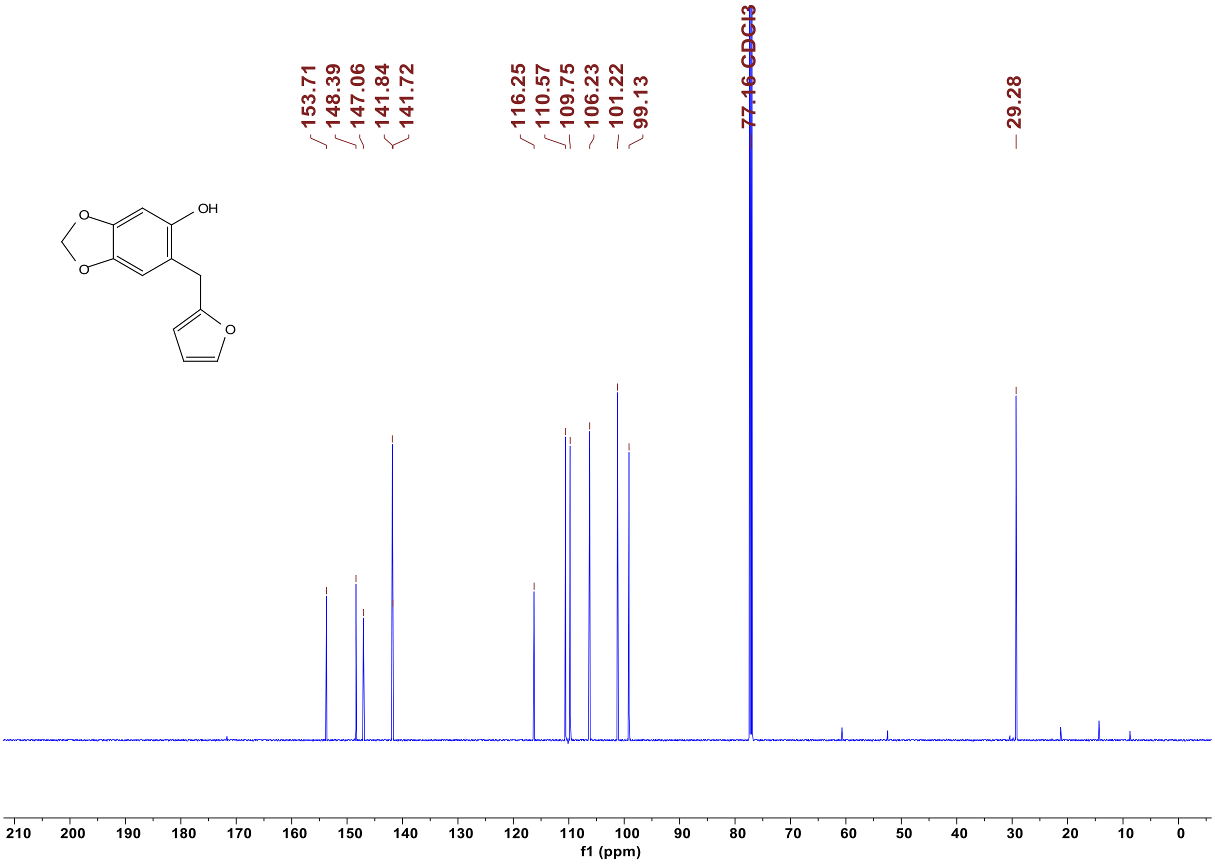

Fig. S24 $^{13}C\{^{1}H\}$ NMR spectrum of **DAM1** in $CDCl_3$ (151 MHz) CG457

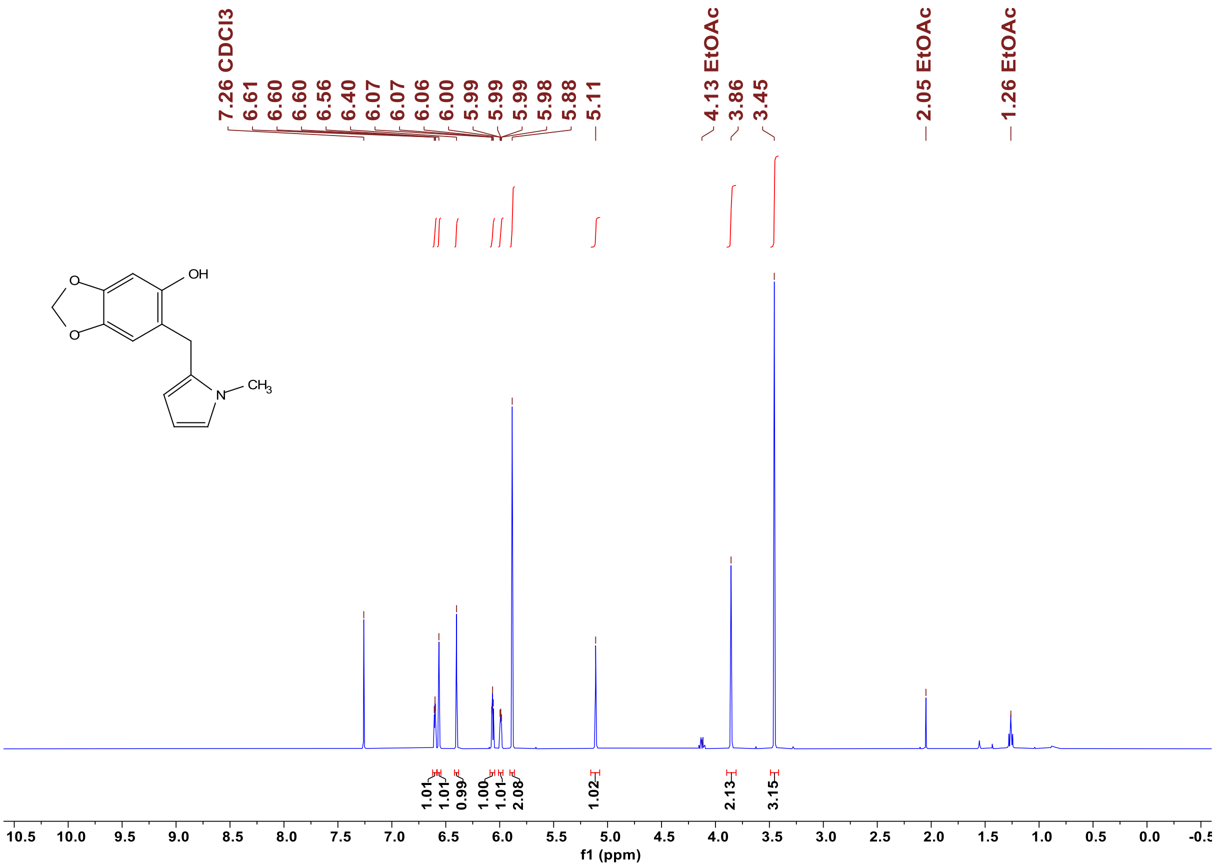

Fig. S25 ^{1}H NMR spectrum of **DAM2** in $CDCl_3$ (400 MHz) CG488_1

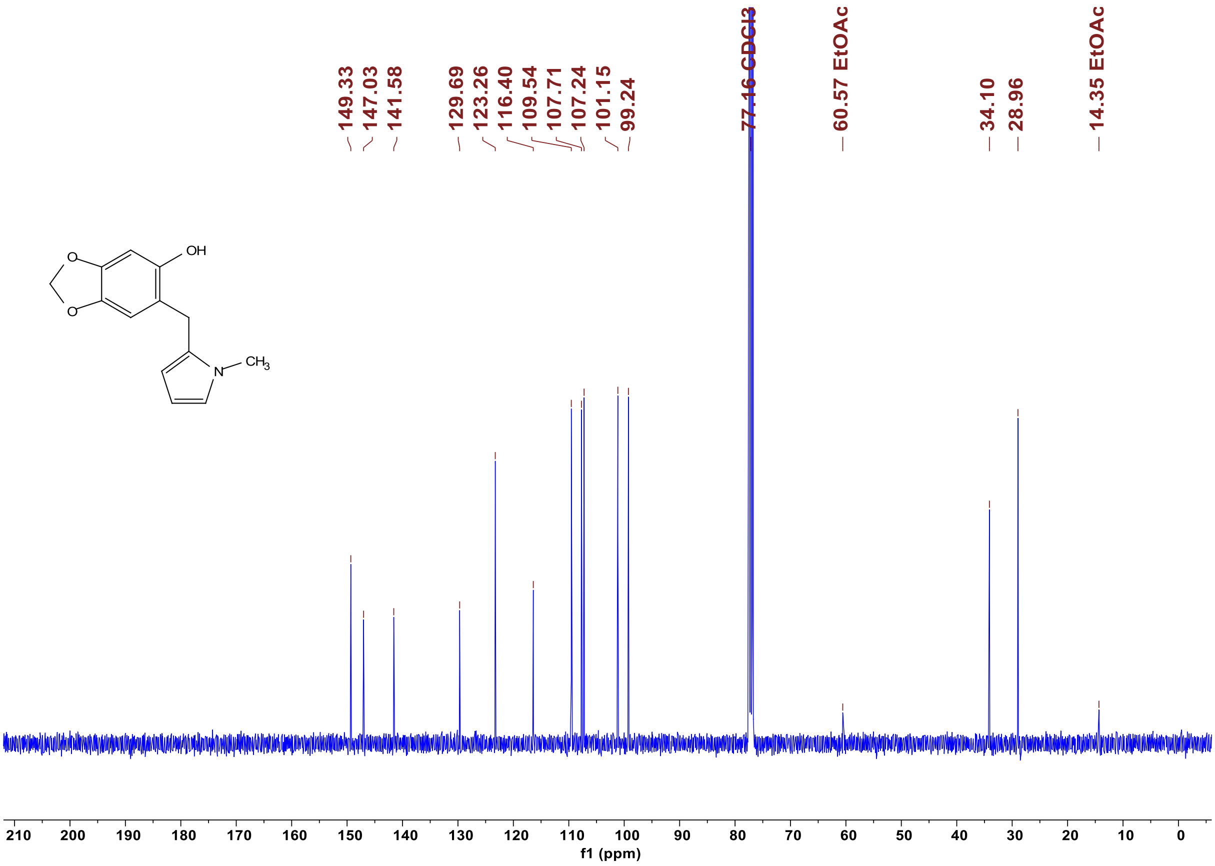

Fig. S26 $^{13}C\{^{1}H\}$ NMR spectrum of **DAM2** in $CDCl_3$ (101 MHz) CG488_1

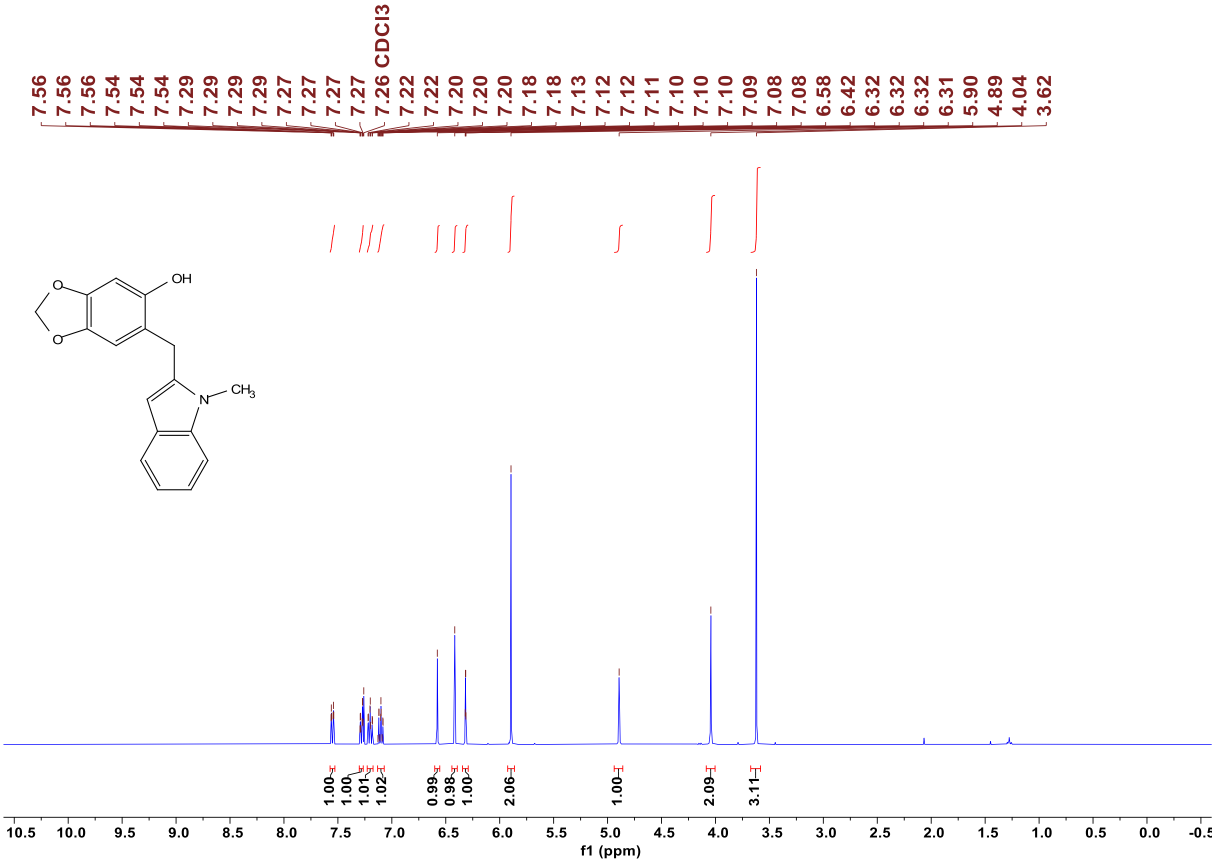

Fig. S27 ^{1}H NMR spectrum of **DAM3** in $CDCl_3$ (400 MHz) CG492

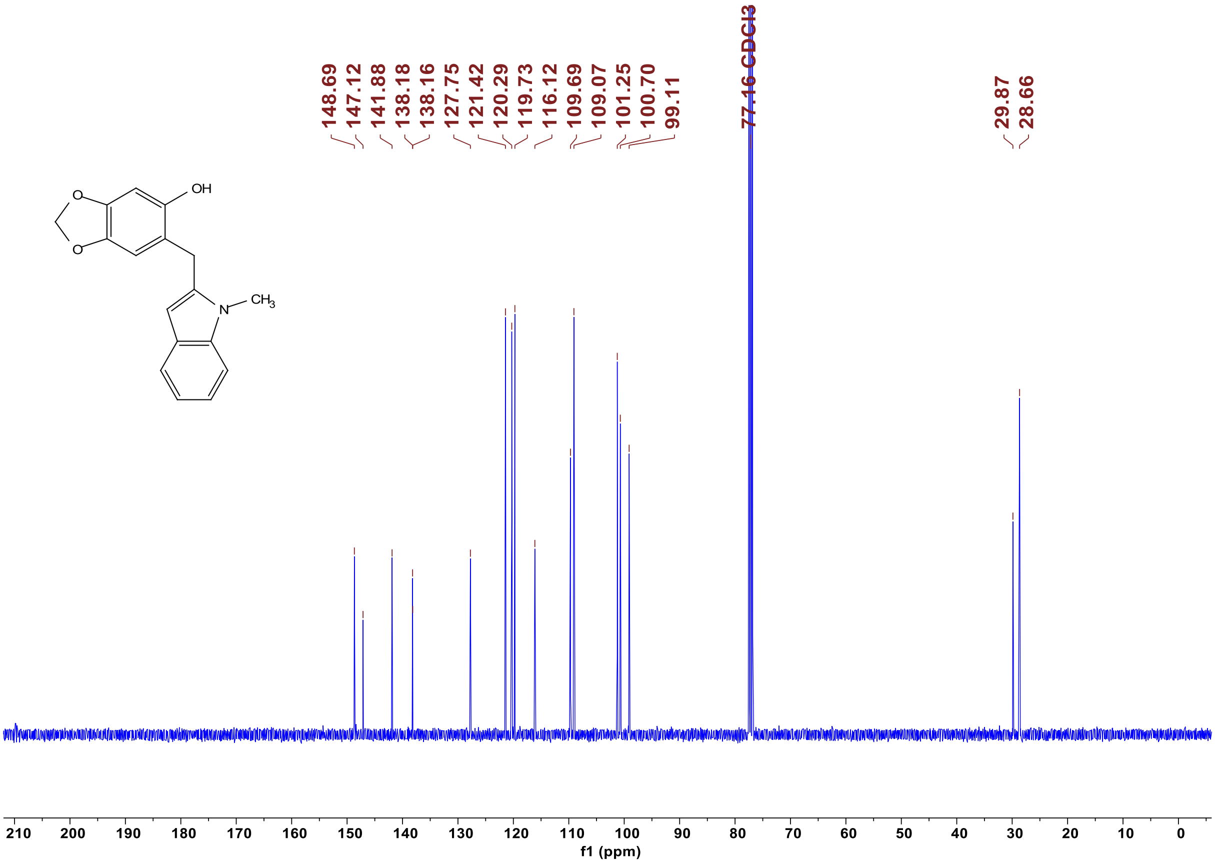

Fig. S28 $^{13}C\{^{1}H\}$ NMR spectrum of **DAM3** in $CDCl_3$ (101 MHz) CG492

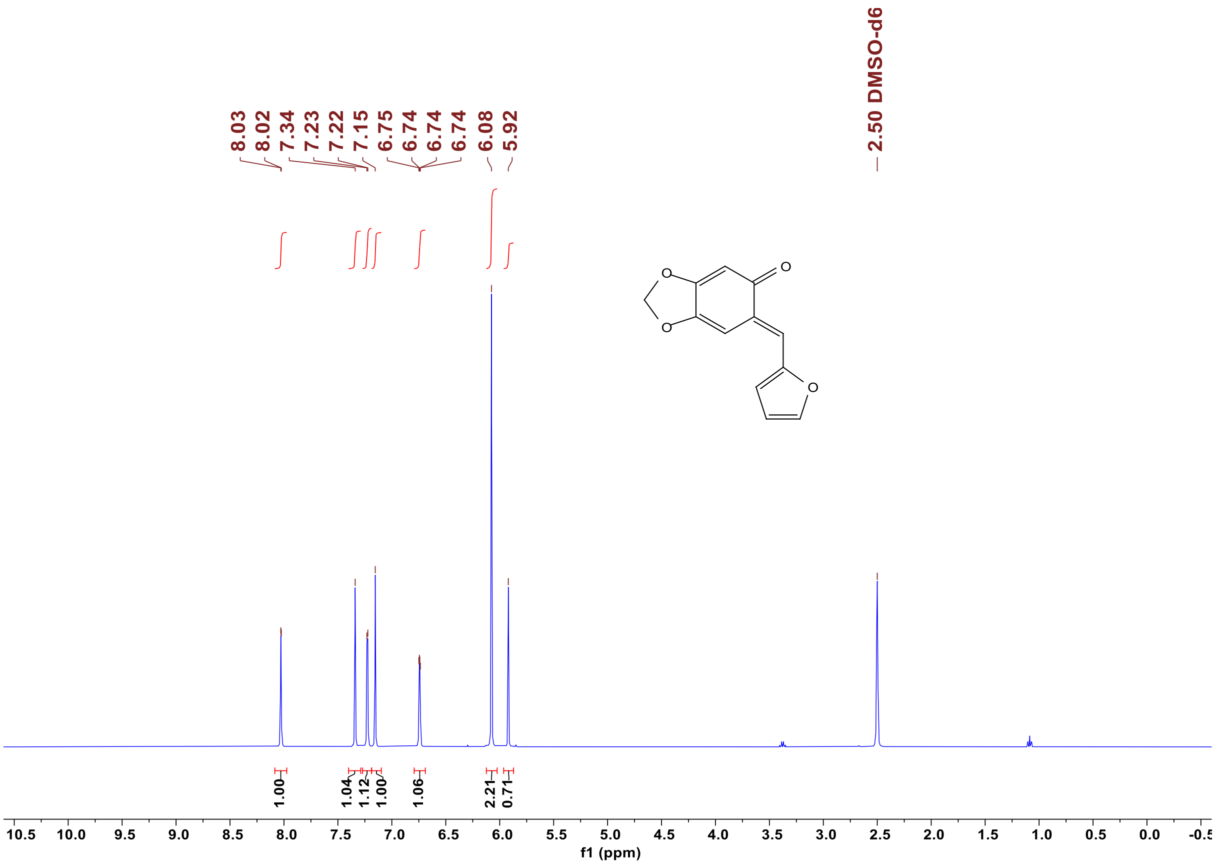

Fig. S29 ¹H NMR spectrum of ***o*QM1** in *d*6-DMSO (400 MHz) CG459

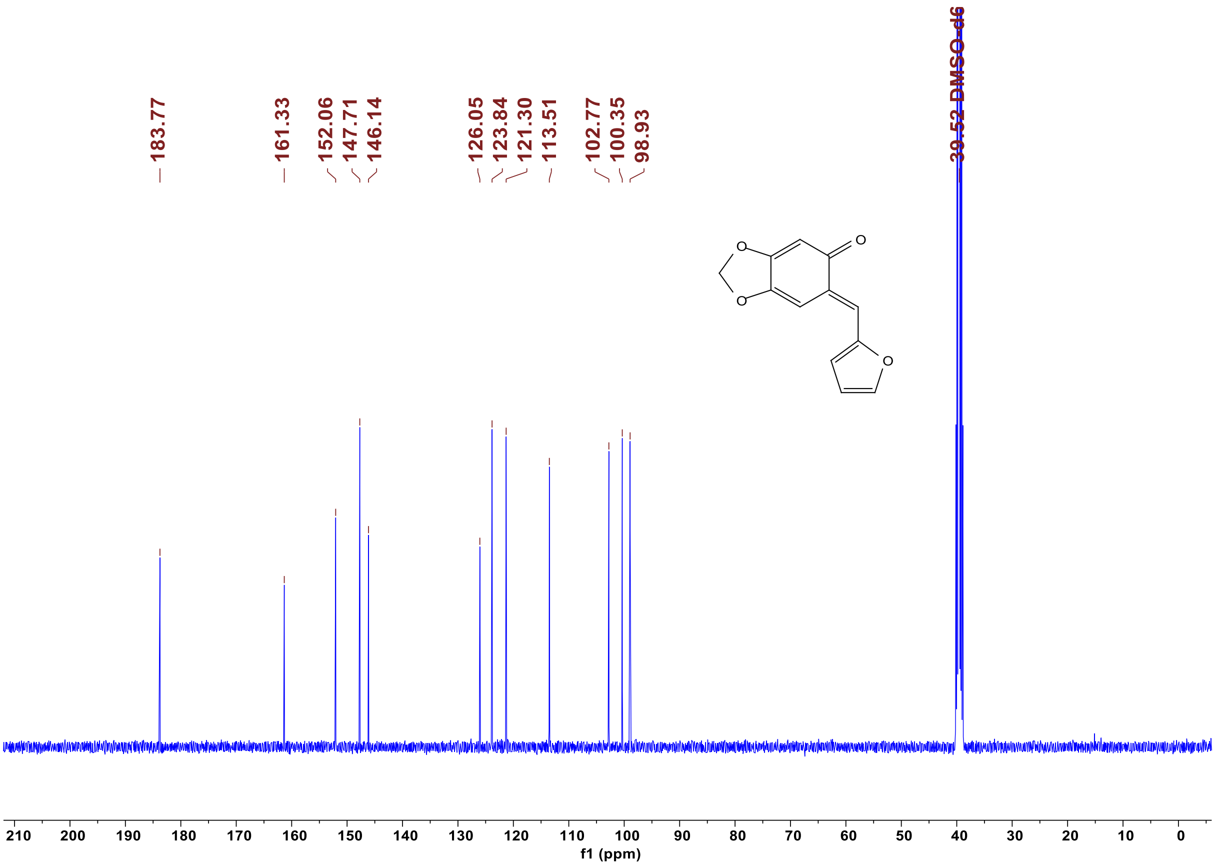

Fig. S30 ^{13}C{^{1}H} NMR spectrum of ***o*QM1** in *d*6-DMSO (101 MHz) CG459

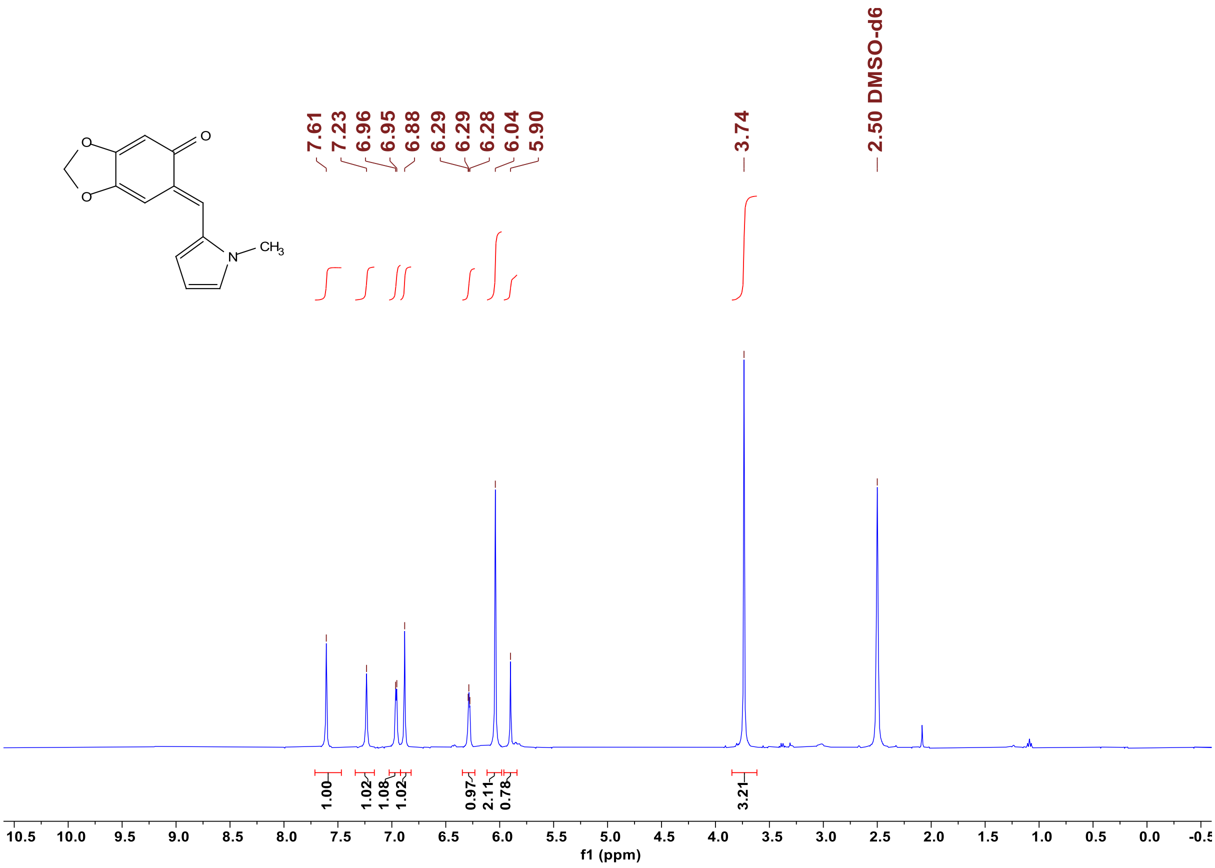

Fig. S31 ^{1}H NMR spectrum of ***o*QM2** in d_6-DMSO (400 MHz) CG489

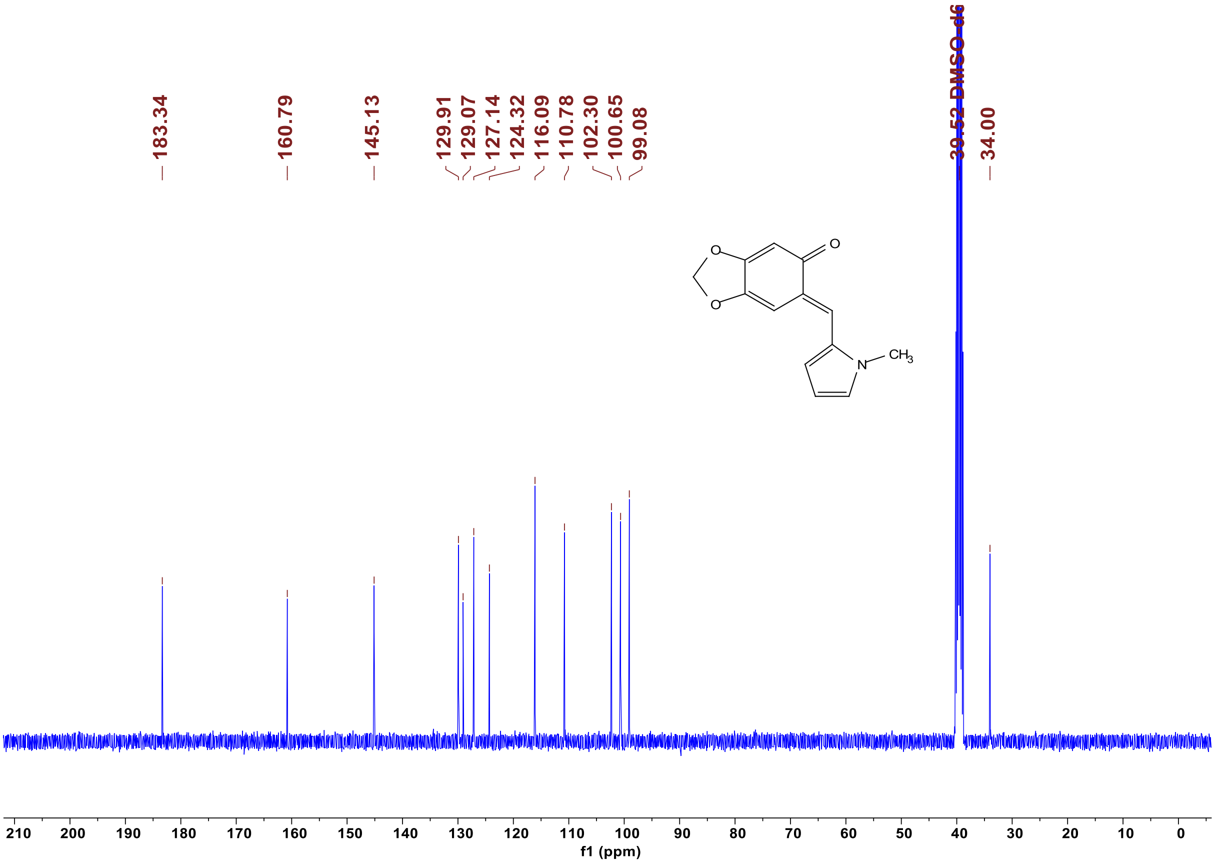

Fig. S32 $^{13}C\{^{1}H\}$ NMR spectrum of ***o*QM2** in d_6-DMSO (101 MHz) CG489

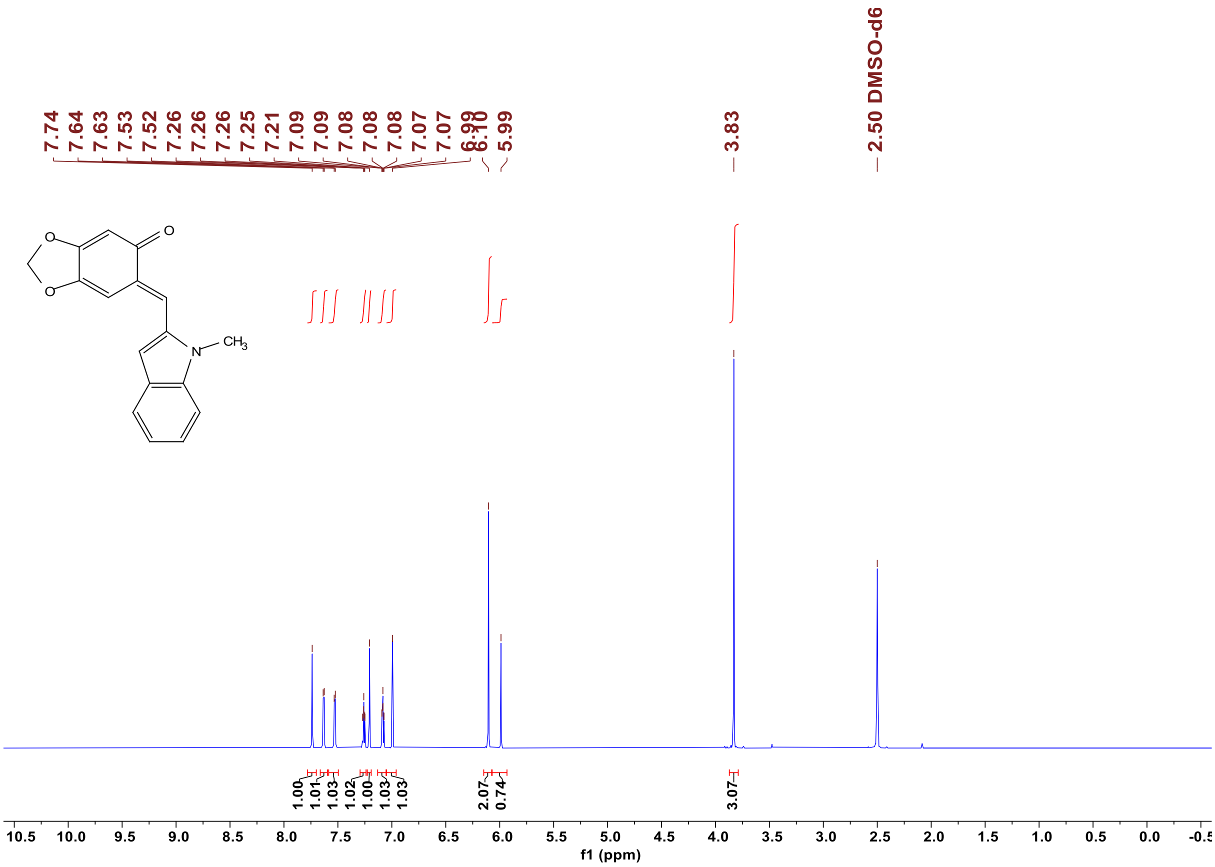

Fig. S33 ^{1}H NMR spectrum of ***o*QM3** in d_6-DMSO (800 MHz) CG495

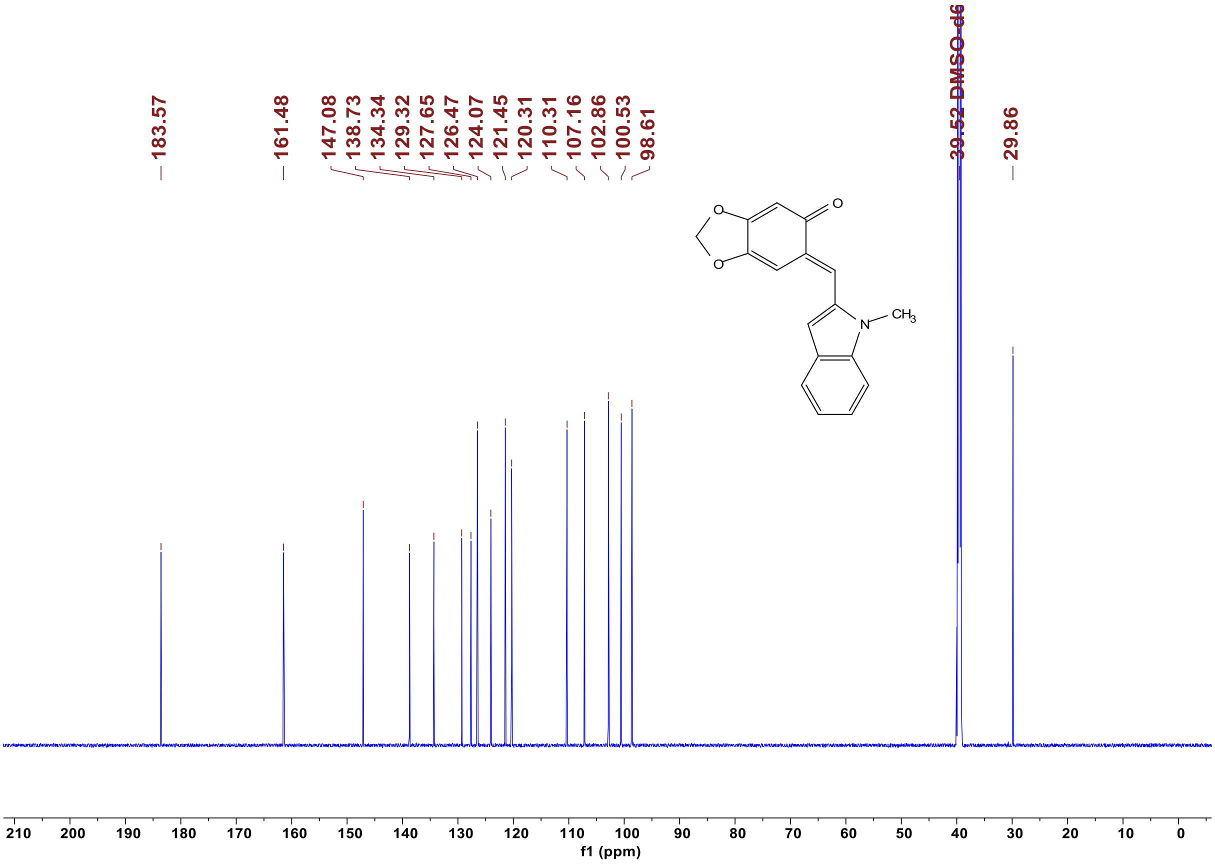

Fig. S34 $^{13}C\{^{1}H\}$ NMR spectrum of ***o*QM3** in d_6-DMSO (201 MHz) CG495

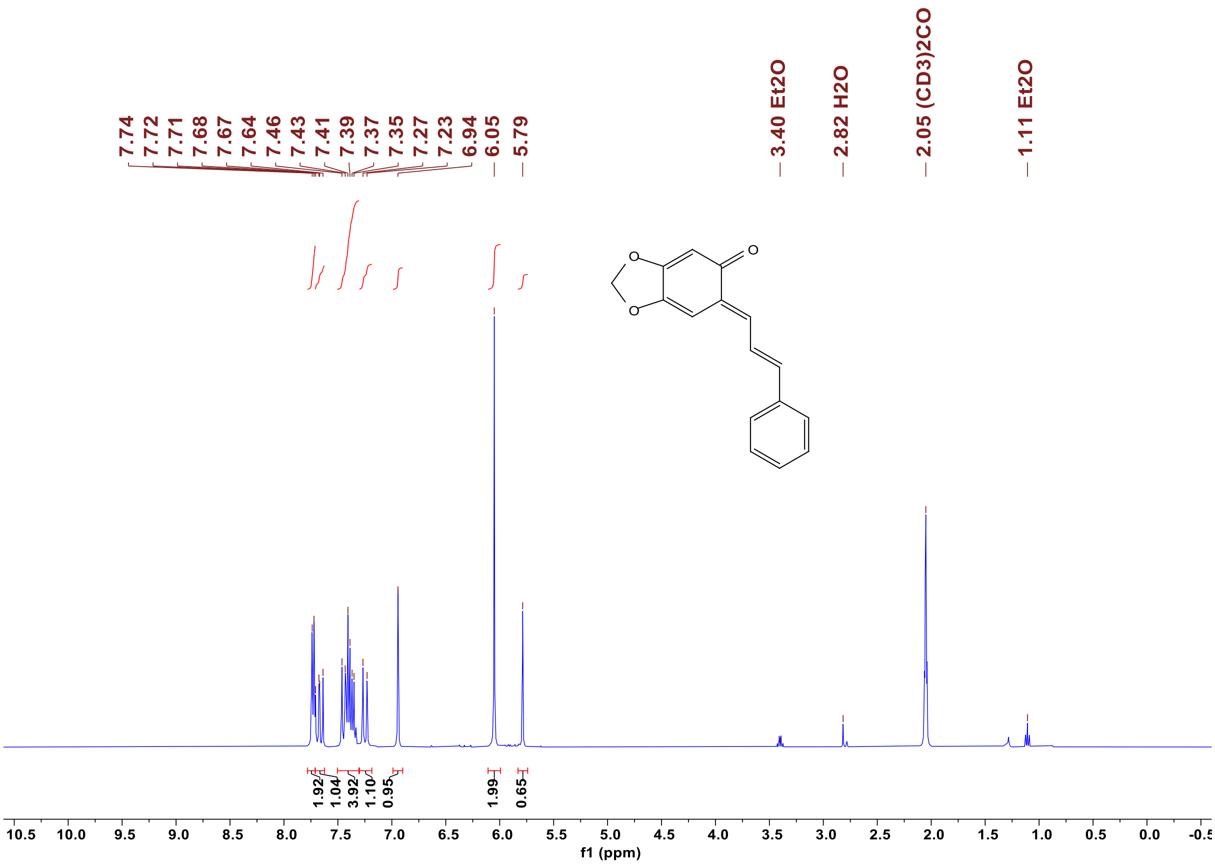

Fig. S35 ^{1}H NMR spectrum of ***o*QM4** in d_6-acetone (400 MHz) CG373

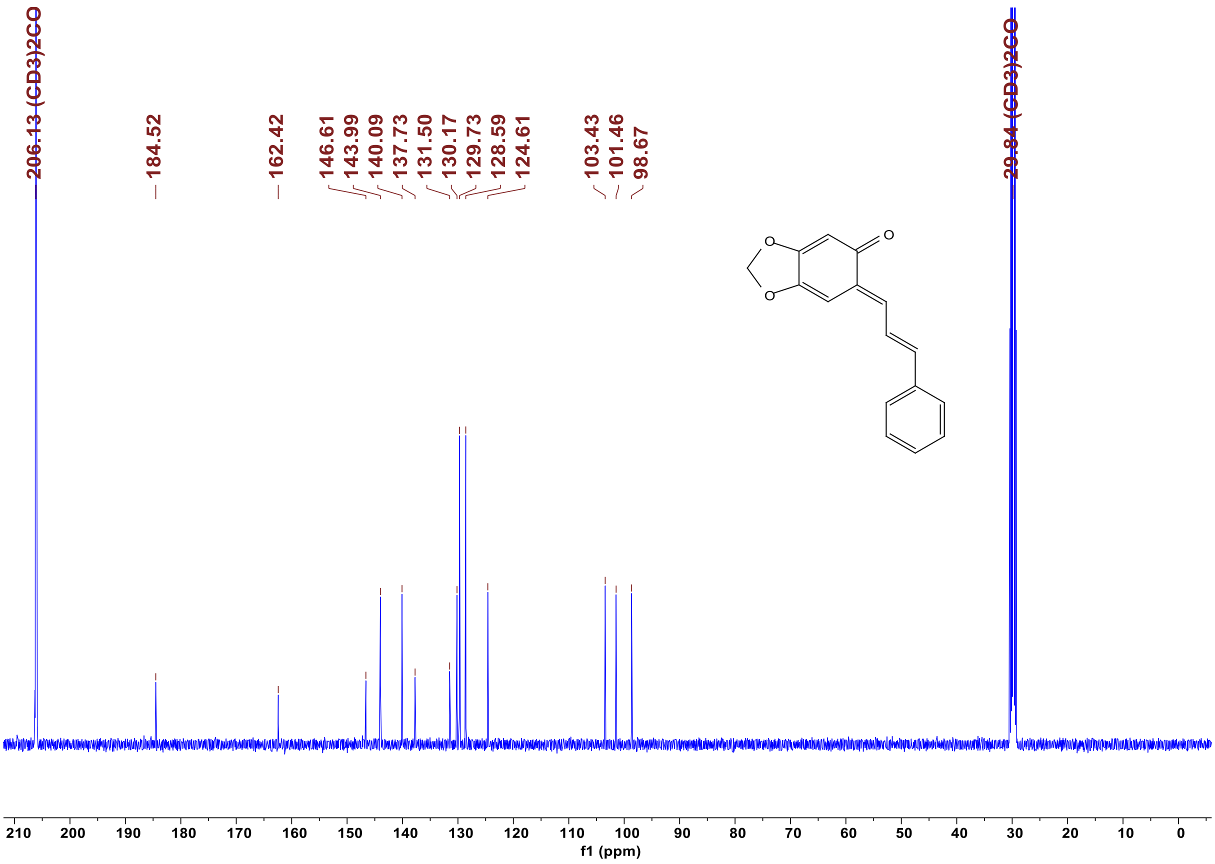

Fig. S36 ^{13}C{^{1}H} NMR spectrum of ***o*QM4** in d_6-acetone (101 MHz) CG373

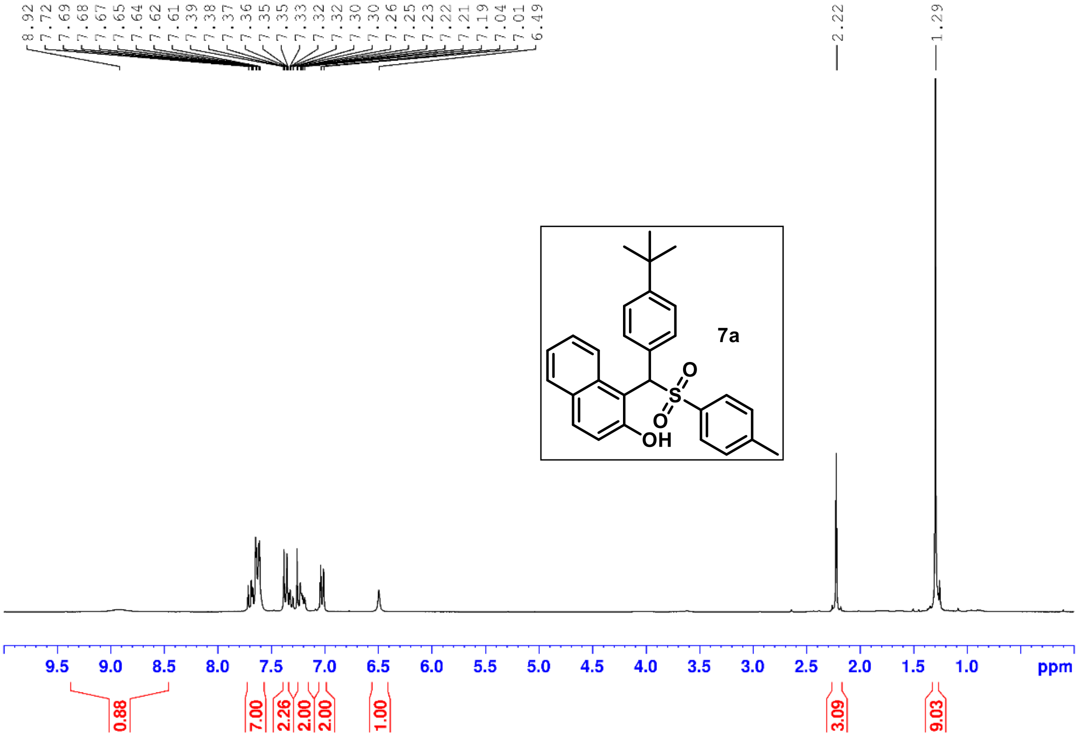
8.92 7.72 7.69 7.68 7.67 7.65 7.64 7.62 7.61 7.39 7.38 7.37 7.36 7.35 7.33 7.32 7.30 7.26 7.25 7.23 7.22 7.21 7.19 7.04 7.01 6.49
2.22
1.29
7a
O
S
O
OH
9.5 9.0 8.5 8.0 7.5 7.0 6.5 6.0 5.5 5.0 4.5 4.0 3.5 3.0 2.5 2.0 1.5 1.0 ppm
0.88 7.00 2.26 2.00 2.00 1.00 3.09 9.03

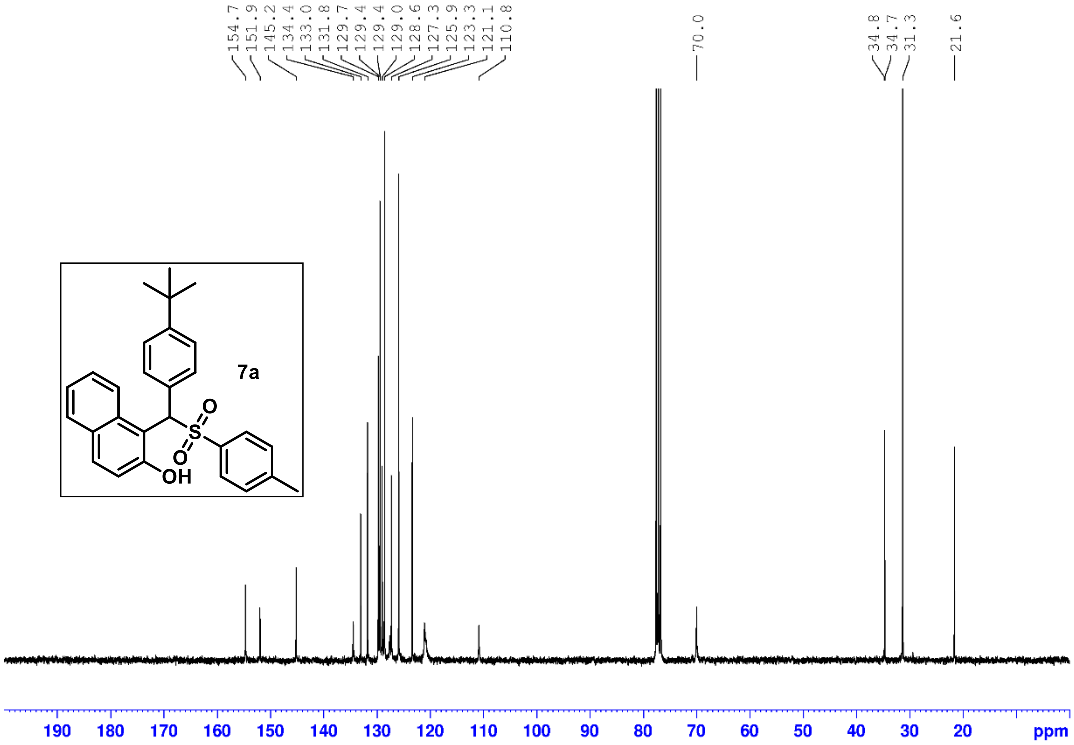
154.7 151.9 145.2 134.4 133.0 131.8 129.7 129.4 129.4 129.0 128.6 127.3 125.9 123.3 121.1 110.8
70.0
34.8 34.7 31.3
21.6
7a
O
S
O
OH
190 180 170 160 150 140 130 120 110 100 90 80 70 60 50 40 30 20 ppm

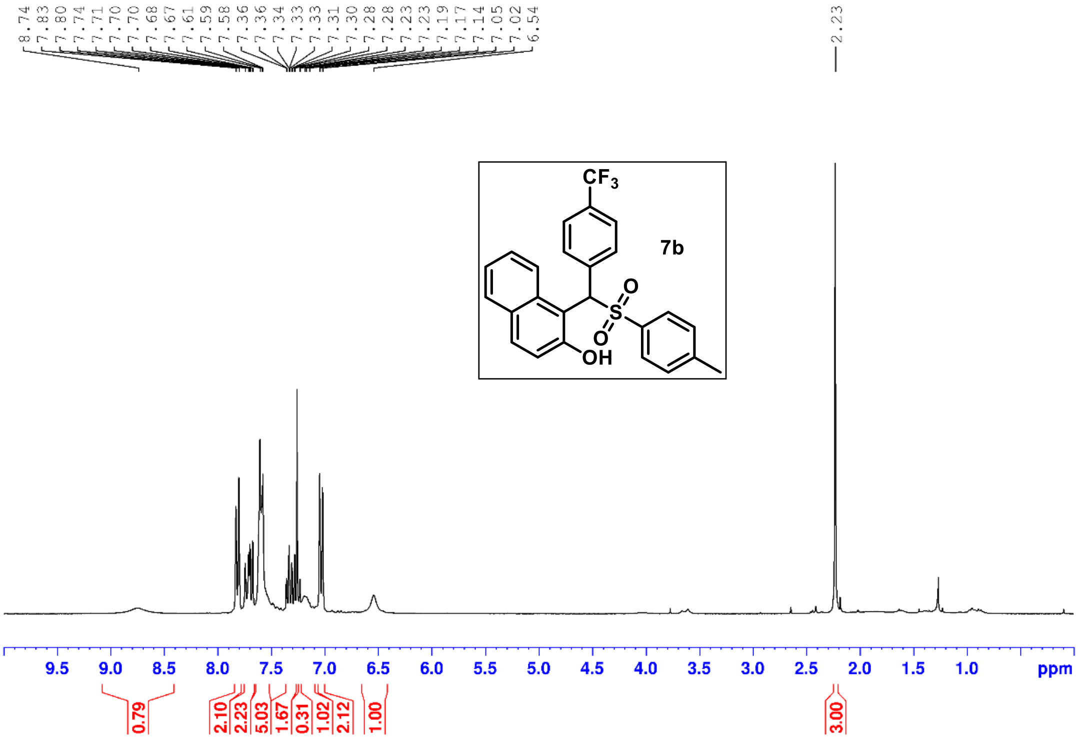

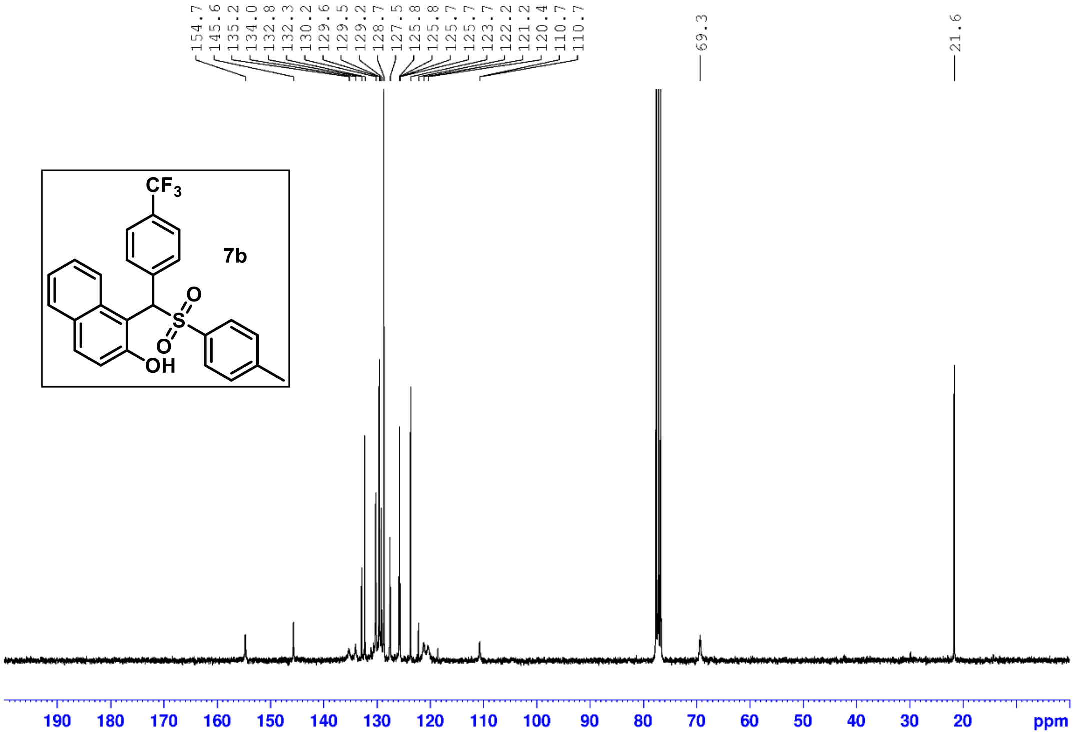

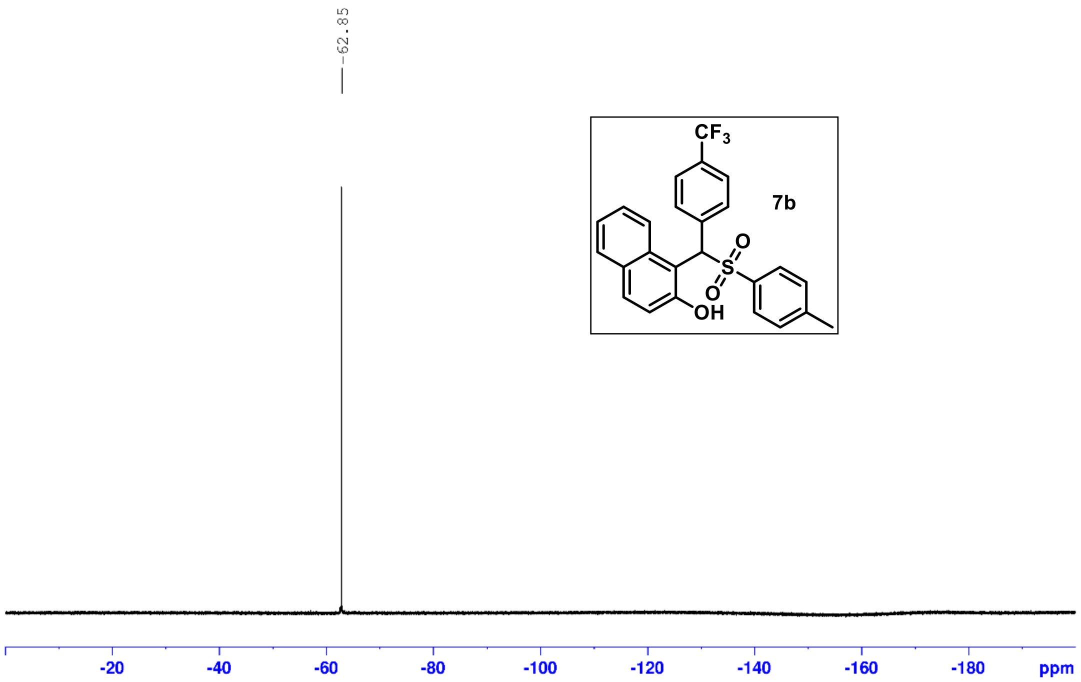

-62.85
CF3
7b
O
S
O
OH
-20
-40
-60
-80
-100
-120
-140
-160
-180
ppm

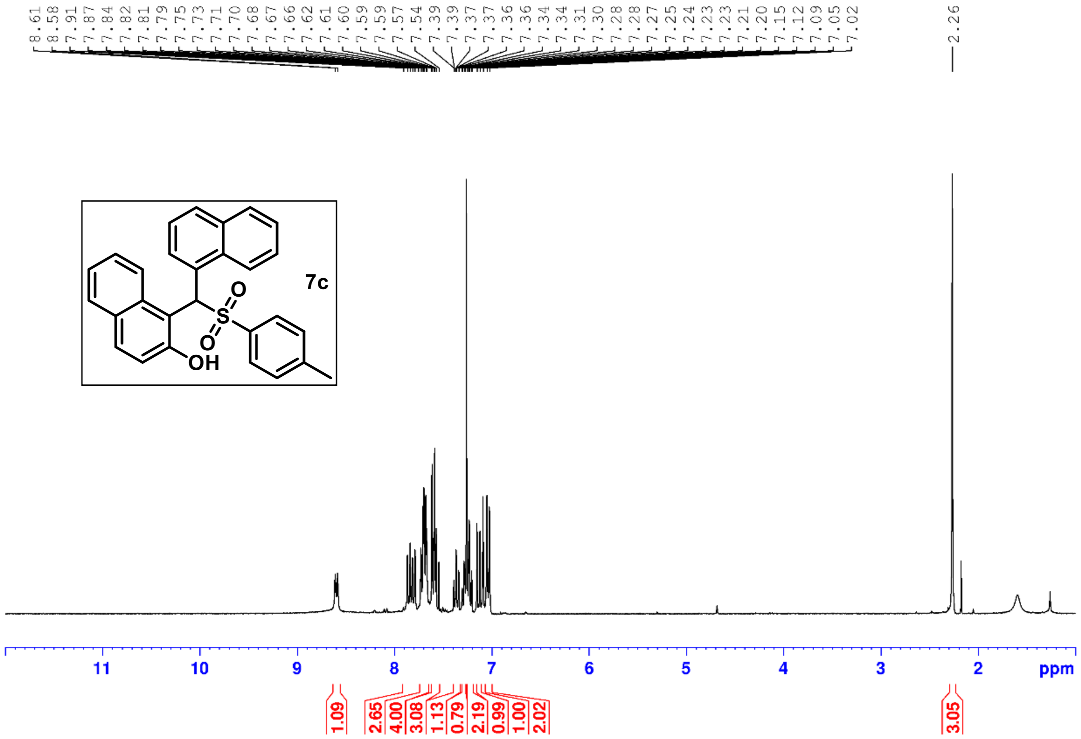
8.61 8.58 7.91 7.87 7.84 7.82 7.81 7.79 7.75 7.73 7.71 7.70 7.68 7.67 7.66 7.62 7.61 7.60 7.59 7.59 7.57 7.54 7.39 7.39 7.37 7.37 7.36 7.36 7.34 7.34 7.31 7.30 7.28 7.28 7.27 7.25 7.24 7.23 7.23 7.21 7.20 7.15 7.12 7.09 7.05 7.02
2.26
7c
O
S
O
OH
11 10 9 8 7 6 5 4 3 2 ppm
1.09 2.65 4.00 3.08 1.13 0.79 2.19 0.99 1.00 2.02
3.05

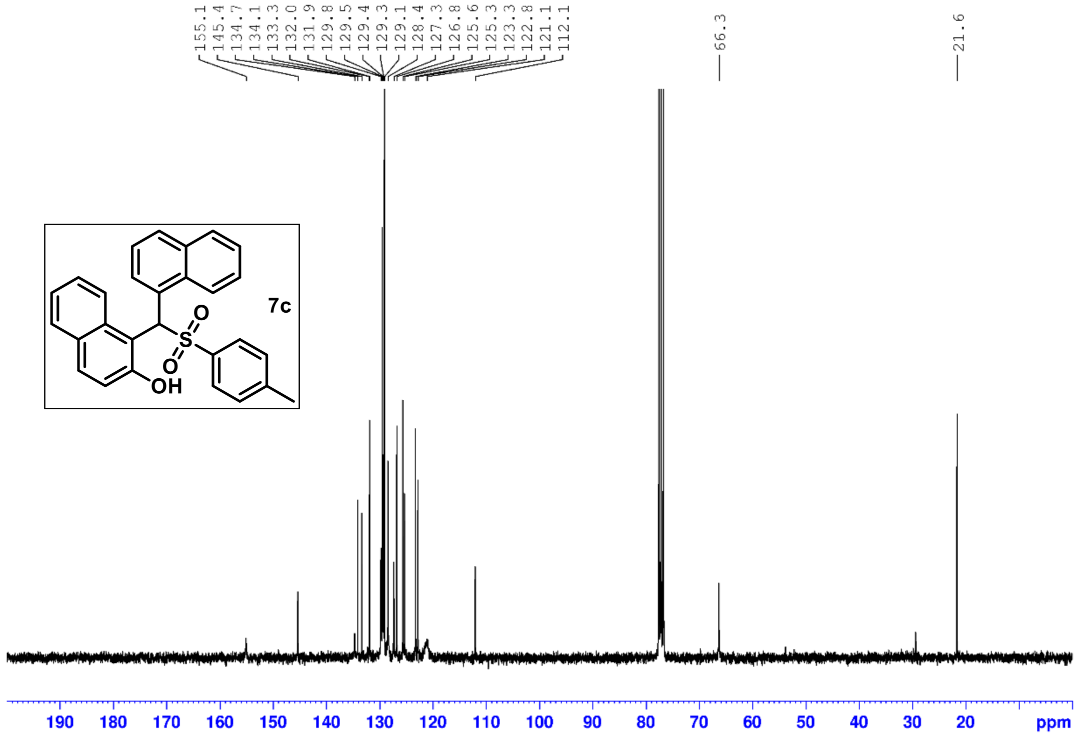
155.1 145.4 134.7 134.1 133.3 132.0 131.9 129.8 129.5 129.4 129.3 129.1 128.4 127.3 126.8 125.6 125.3 123.3 122.8 121.1 112.1
66.3
21.6
7c
O
S
O
OH
190 180 170 160 150 140 130 120 110 100 90 80 70 60 50 40 30 20 ppm

14.2 NMR spectra of the (4+2)-cycloaddition products

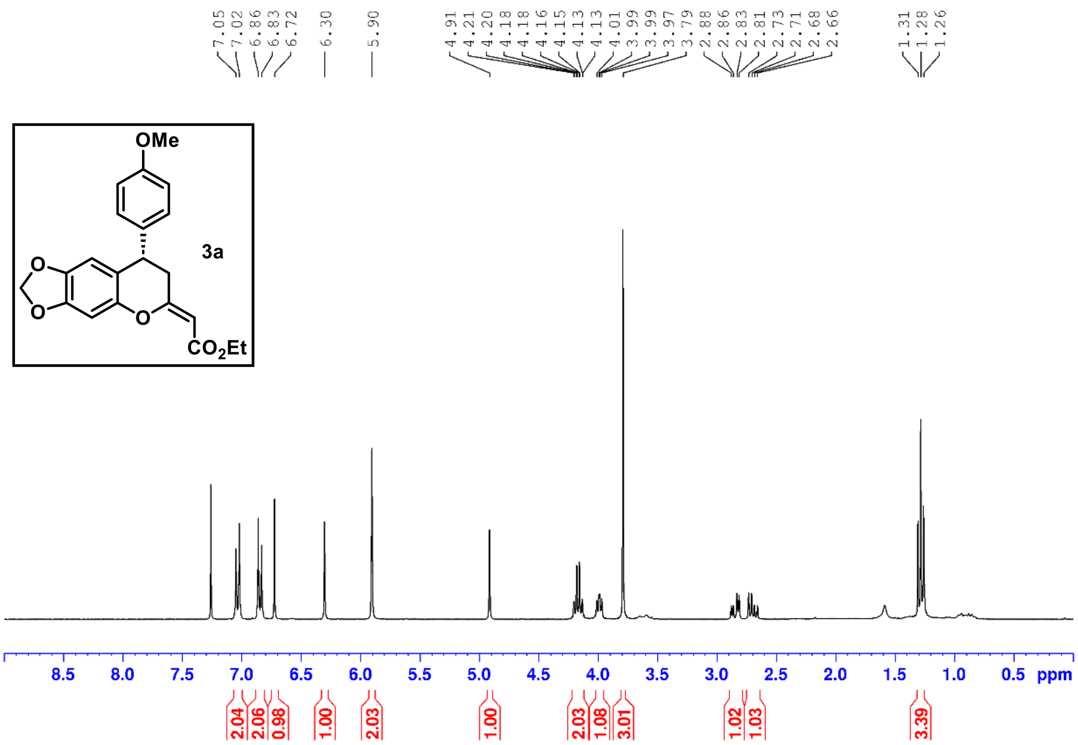

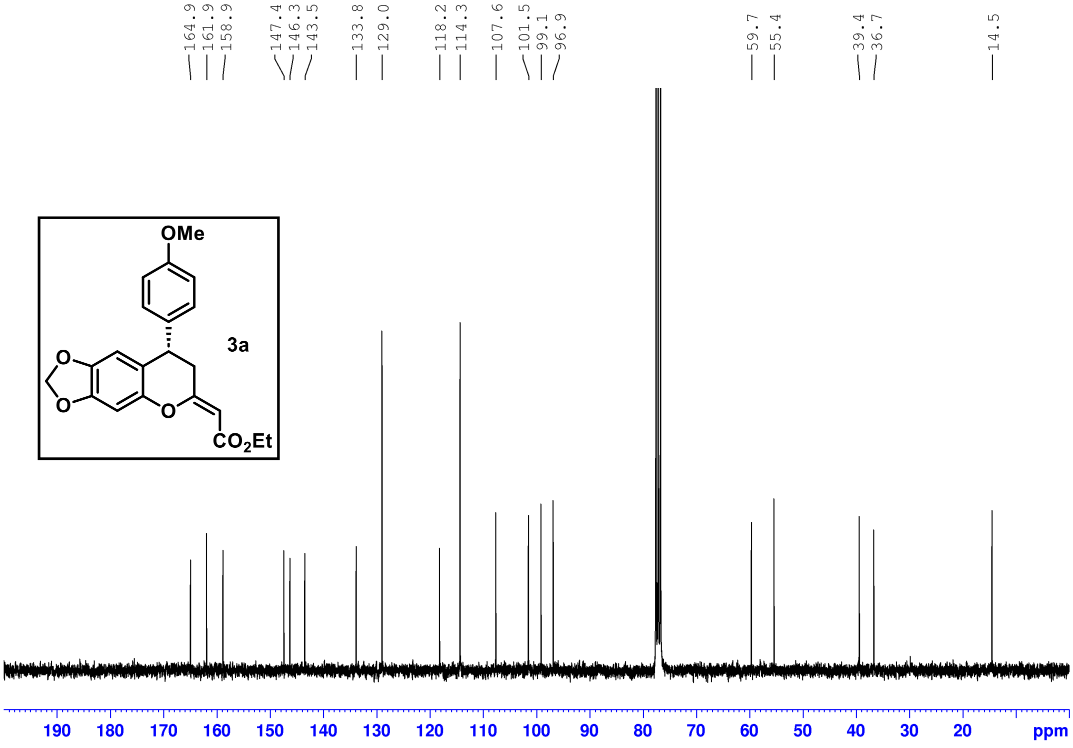

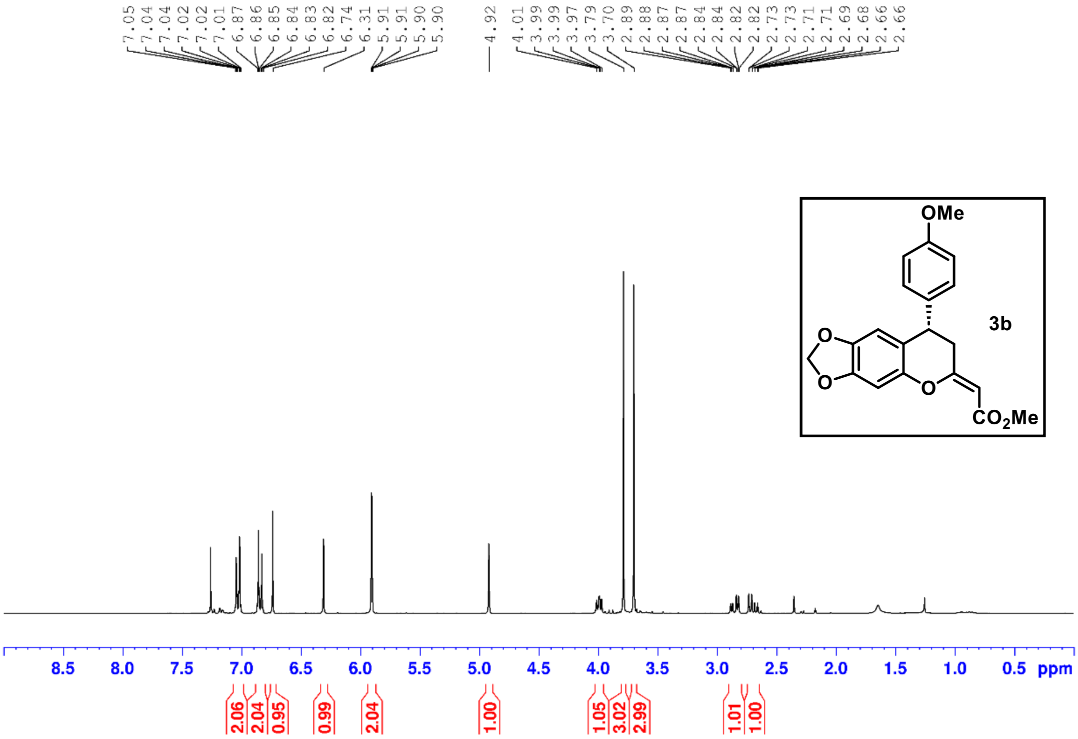
OMe
3b
O
O
O
CO2Me
8.5
8.0
7.5
7.0
6.5
6.0
5.5
5.0
4.5
4.0
3.5
3.0
2.5
2.0
1.5
1.0
0.5
ppm
2.06
2.04
0.95
0.99
2.04
1.00
1.05
3.02
2.99
1.01
1.00

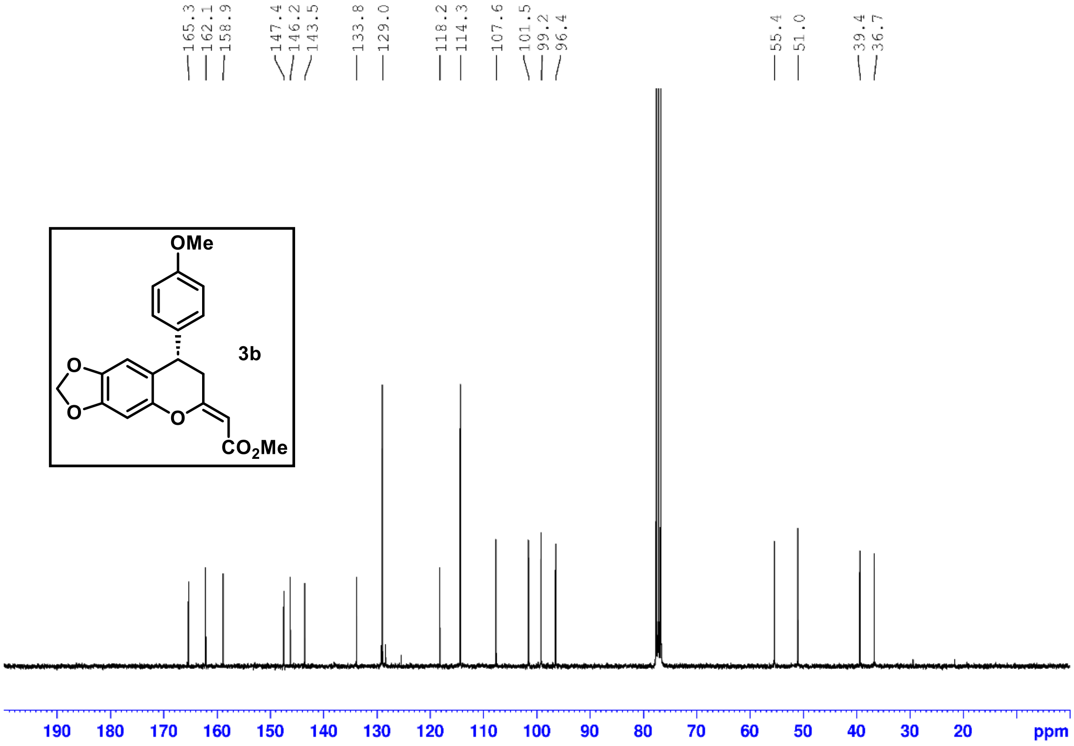
165.3
162.1
158.9
147.4
146.2
143.5
133.8
129.0
118.2
114.3
107.6
101.5
99.2
96.4
55.4
51.0
39.4
36.7
OMe
3b
O
O
O
CO2Me
190
180
170
160
150
140
130
120
110
100
90
80
70
60
50
40
30
20
ppm

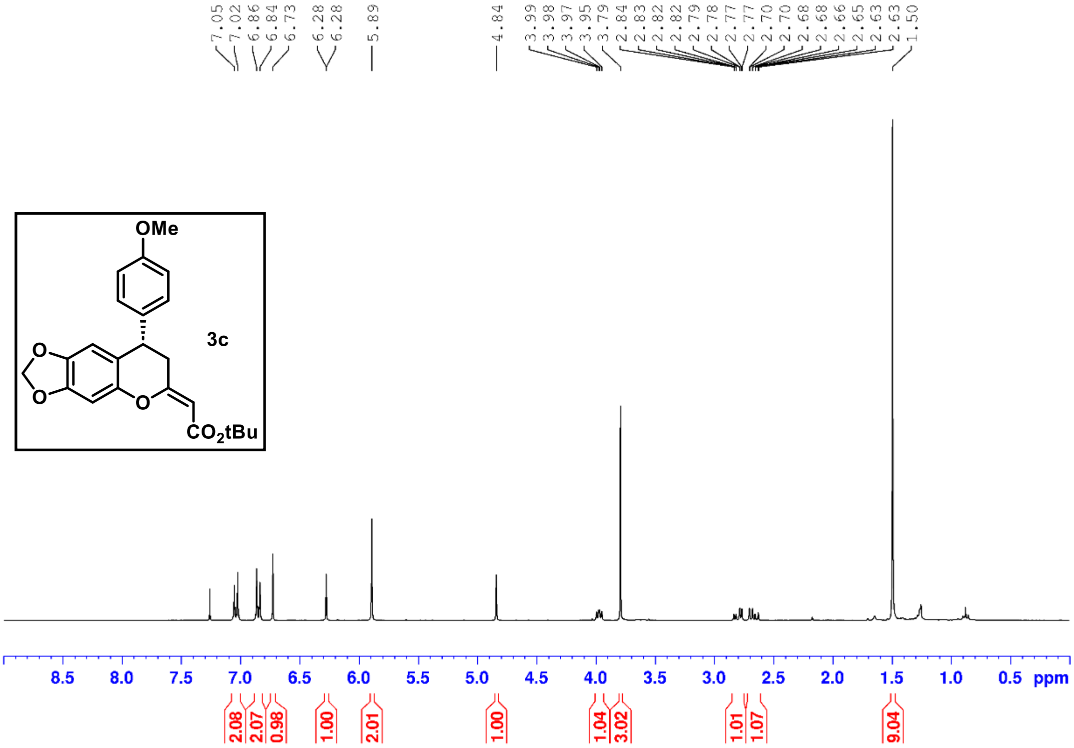
7.05
7.02
6.86
6.84
6.73
6.28
6.28
5.89
4.84
3.99
3.98
3.97
3.95
3.79
2.84
2.83
2.82
2.82
2.79
2.78
2.77
2.77
2.70
2.70
2.68
2.68
2.66
2.65
2.63
2.63
1.50
OMe
3c
O
O
O
CO2tBu
8.5
8.0
7.5
7.0
6.5
6.0
5.5
5.0
4.5
4.0
3.5
3.0
2.5
2.0
1.5
1.0
0.5
ppm
2.08
2.07
0.98
1.00
2.01
1.00
1.04
3.02
1.01
1.07
9.04

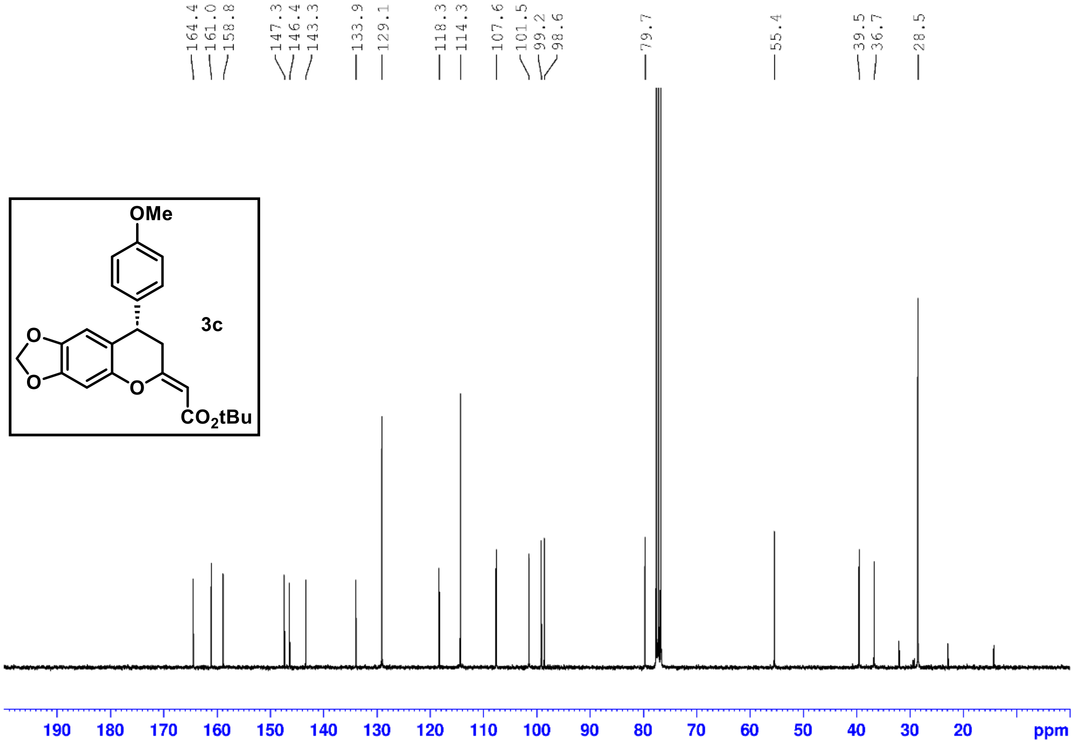
164.4
161.0
158.8
147.3
146.4
143.3
133.9
129.1
118.3
114.3
107.6
101.5
99.2
98.6
79.7
55.4
39.5
36.7
28.5
OMe
3c
O
O
O
CO2tBu
190
180
170
160
150
140
130
120
110
100
90
80
70
60
50
40
30
20
ppm

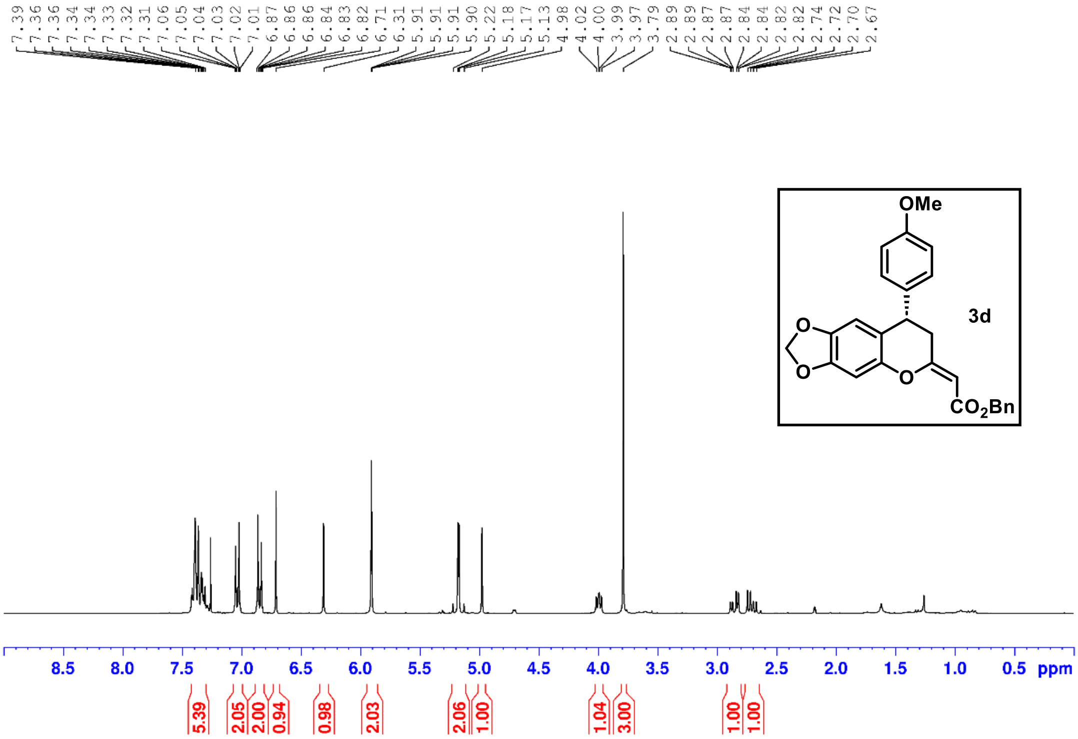
7.39 7.36 7.36 7.34 7.34 7.33 7.32 7.31 7.06 7.05 7.04 7.03 7.02 7.01 6.87 6.86 6.86 6.84 6.83 6.82 6.71 6.31 5.91 5.91 5.91 5.90 5.22 5.18 5.17 5.13 4.98 4.02 4.00 3.99 3.97 3.79 2.89 2.87 2.87 2.84 2.84 2.82 2.82 2.74 2.72 2.70 2.67
OMe
3d
O
O
O
CO2Bn
8.5 8.0 7.5 7.0 6.5 6.0 5.5 5.0 4.5 4.0 3.5 3.0 2.5 2.0 1.5 1.0 0.5 ppm
5.39 2.05 2.00 0.94 0.98 2.03 2.06 1.00 1.04 3.00 1.00 1.00

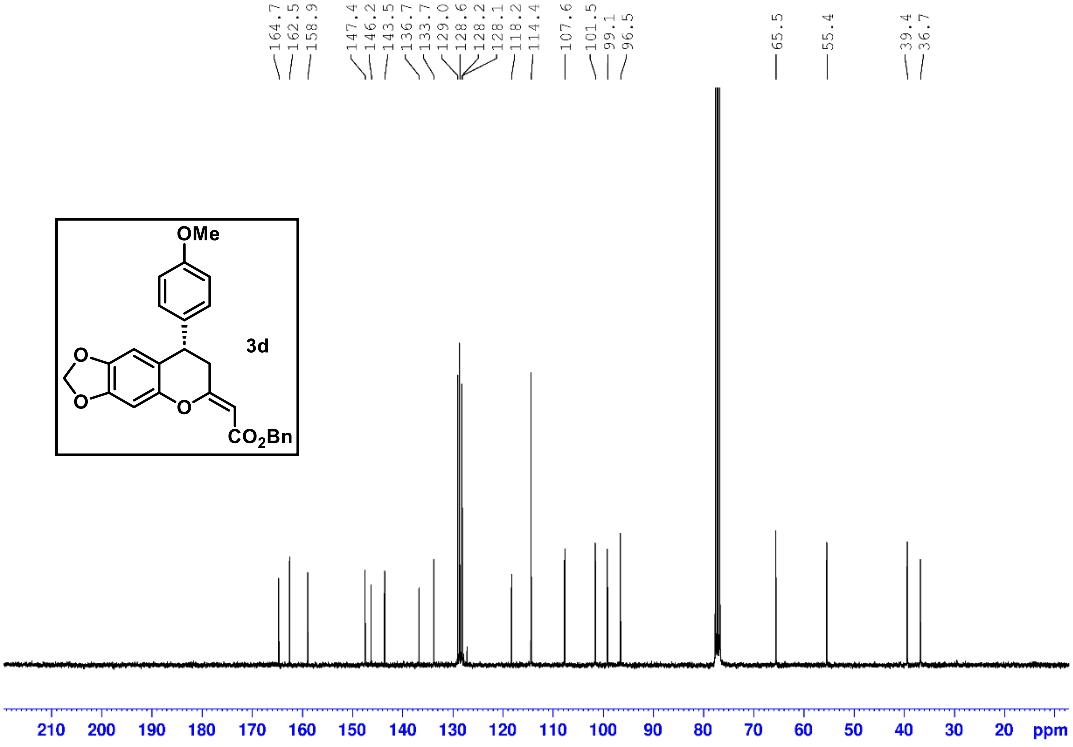
164.7 162.5 158.9 147.4 146.2 143.5 136.7 133.7 129.0 128.6 128.2 128.1 118.2 114.4 107.6 101.5 99.1 96.5 65.5 55.4 39.4 36.7
OMe
3d
O
O
O
CO2Bn
210 200 190 180 170 160 150 140 130 120 110 100 90 80 70 60 50 40 30 20 ppm

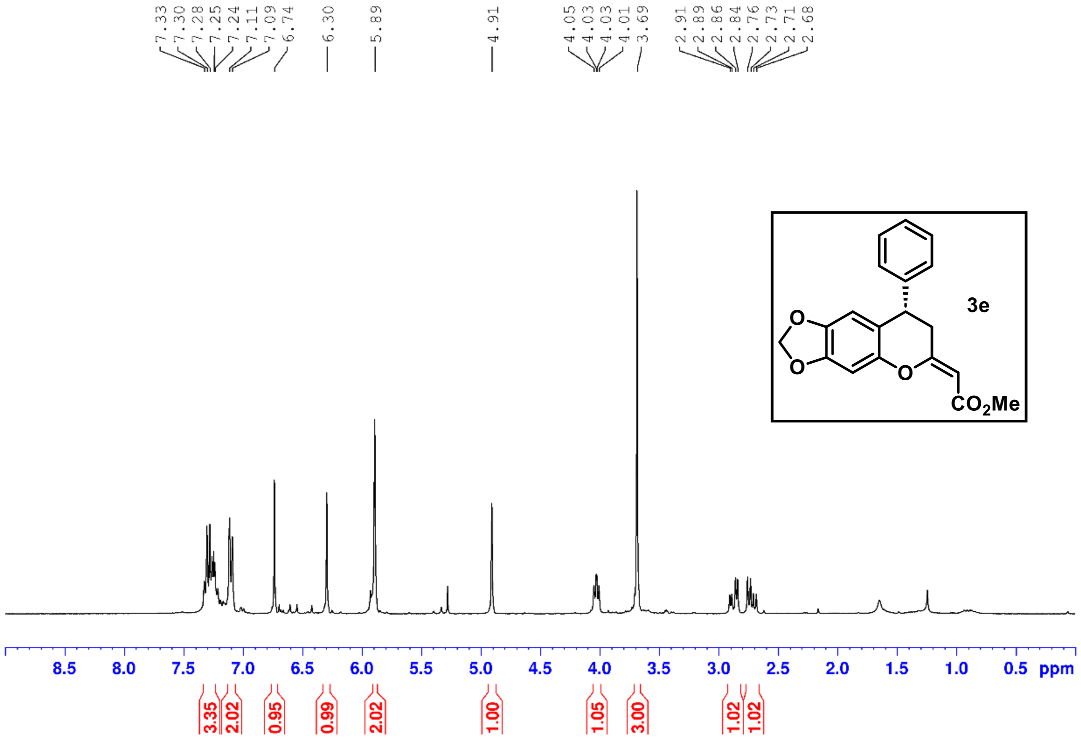
7.33 7.30 7.28 7.25 7.24 7.11 7.09 6.74
6.30
5.89
4.91
4.05 4.03 4.03 4.01 3.69
2.91 2.89 2.86 2.84 2.76 2.73 2.71 2.68
3e
CO_2Me
8.5 8.0 7.5 7.0 6.5 6.0 5.5 5.0 4.5 4.0 3.5 3.0 2.5 2.0 1.5 1.0 0.5 ppm
3.35 2.02 0.95 0.99 2.02 1.00 1.05 3.00 1.02 1.02

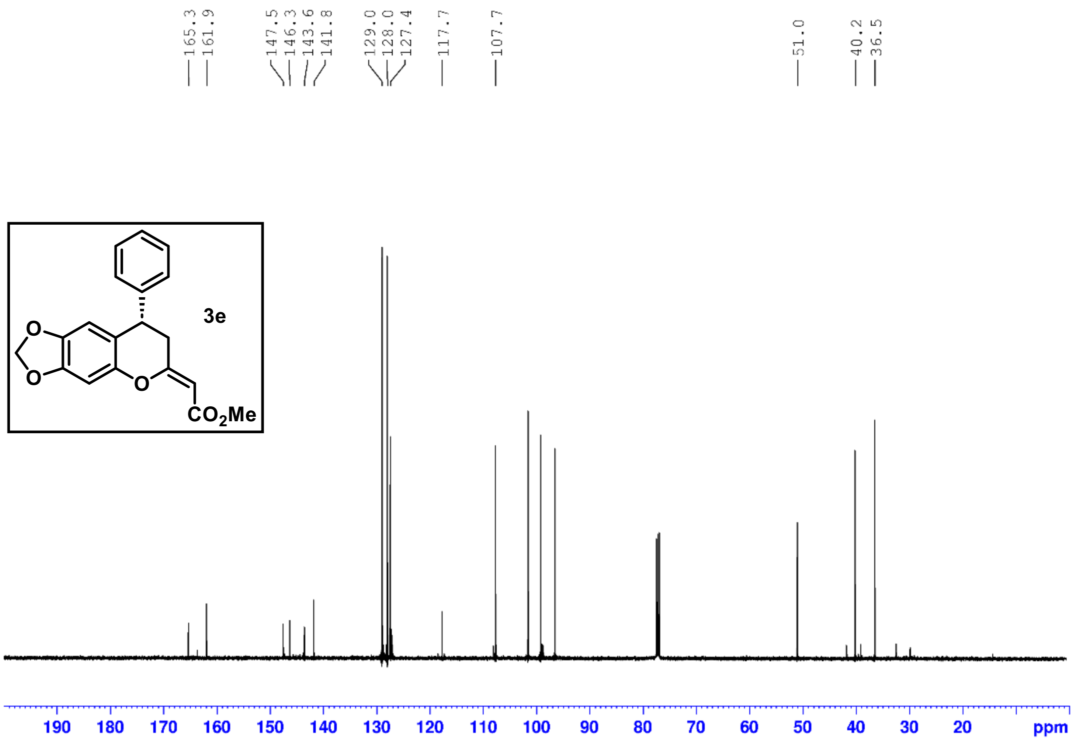
165.3 161.9
147.5 146.3 143.6 141.8
129.0 128.0 127.4
117.7
107.7
51.0
40.2 36.5
3e
CO_2Me
190 180 170 160 150 140 130 120 110 100 90 80 70 60 50 40 30 20 ppm

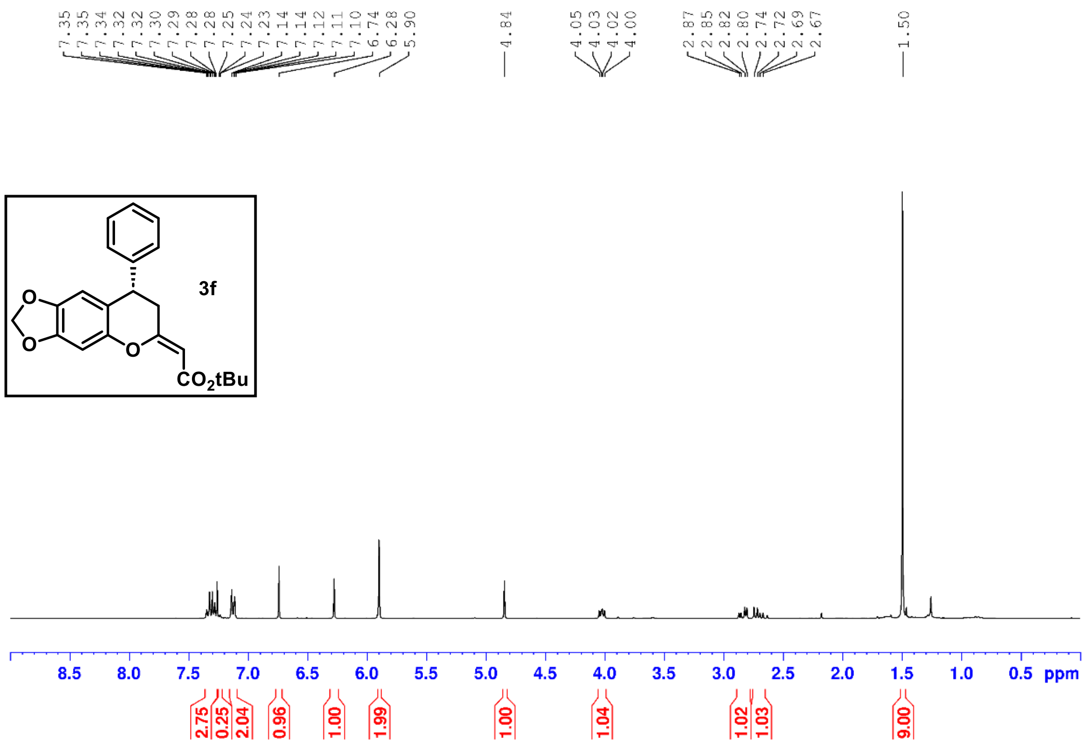

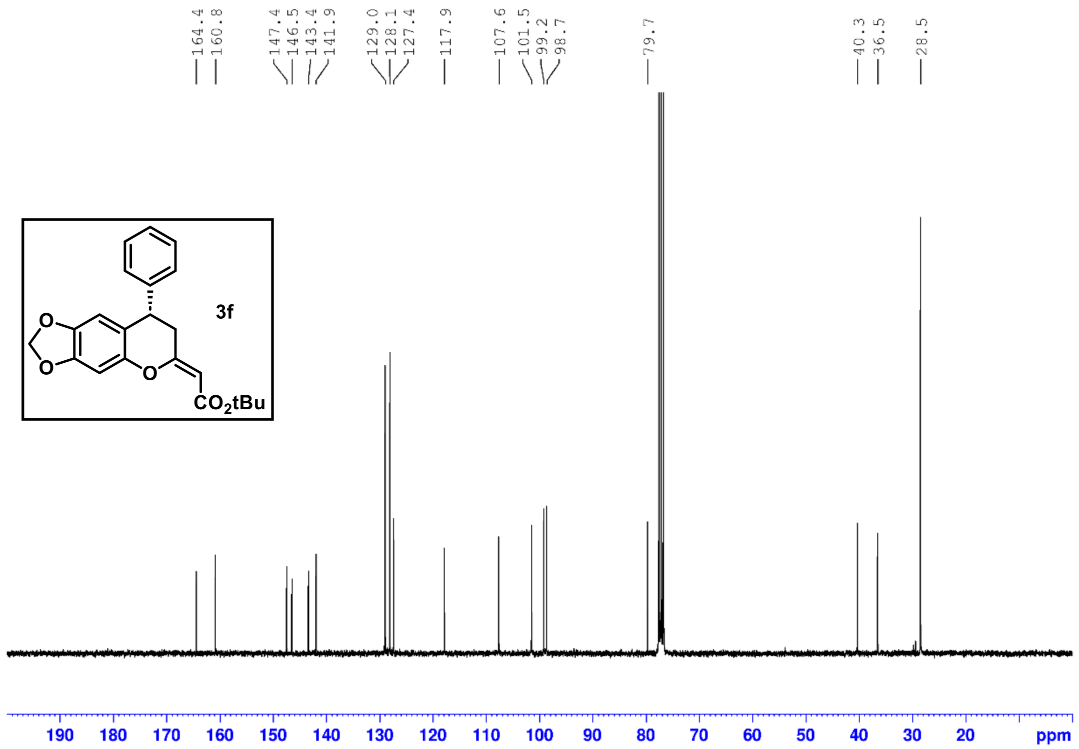

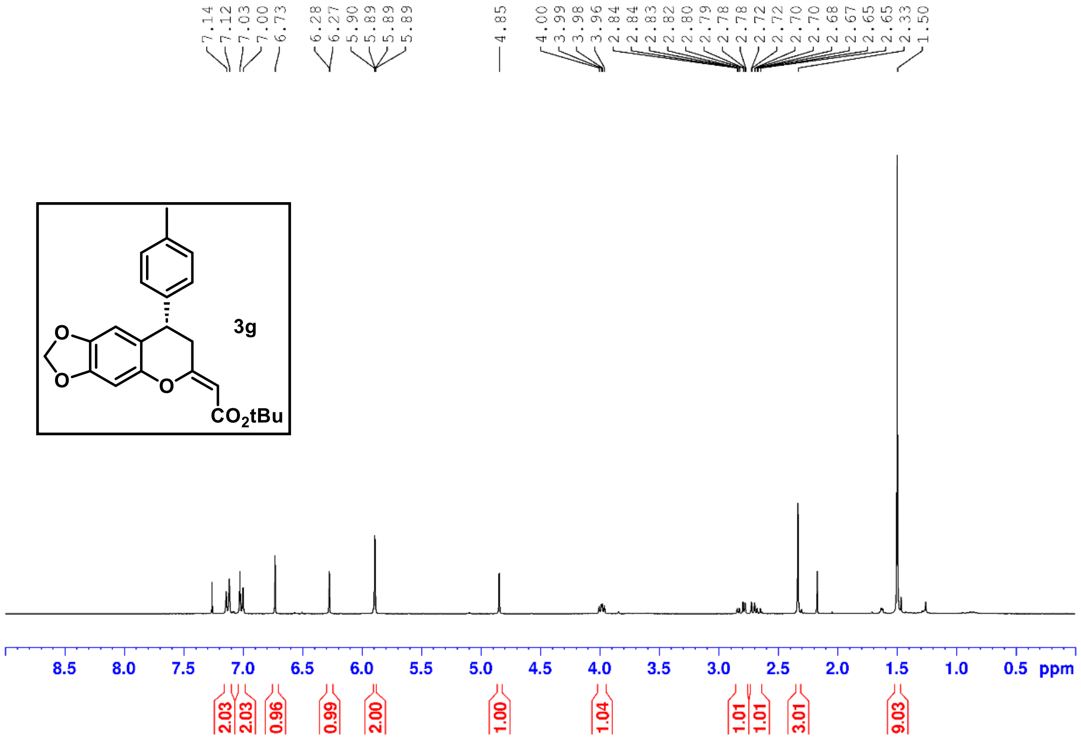
7.14
7.12
7.03
7.00
6.73
6.28
6.27
5.90
5.89
5.89
5.89
4.85
4.00
3.99
3.98
3.96
2.84
2.84
2.83
2.82
2.80
2.79
2.78
2.78
2.72
2.72
2.70
2.70
2.68
2.67
2.65
2.65
2.33
1.50
3g
O
O
O
CO_2tBu
8.5
8.0
7.5
7.0
6.5
6.0
5.5
5.0
4.5
4.0
3.5
3.0
2.5
2.0
1.5
1.0
0.5
ppm
2.03
2.03
0.96
0.99
2.00
1.00
1.04
1.01
1.01
3.01
9.03

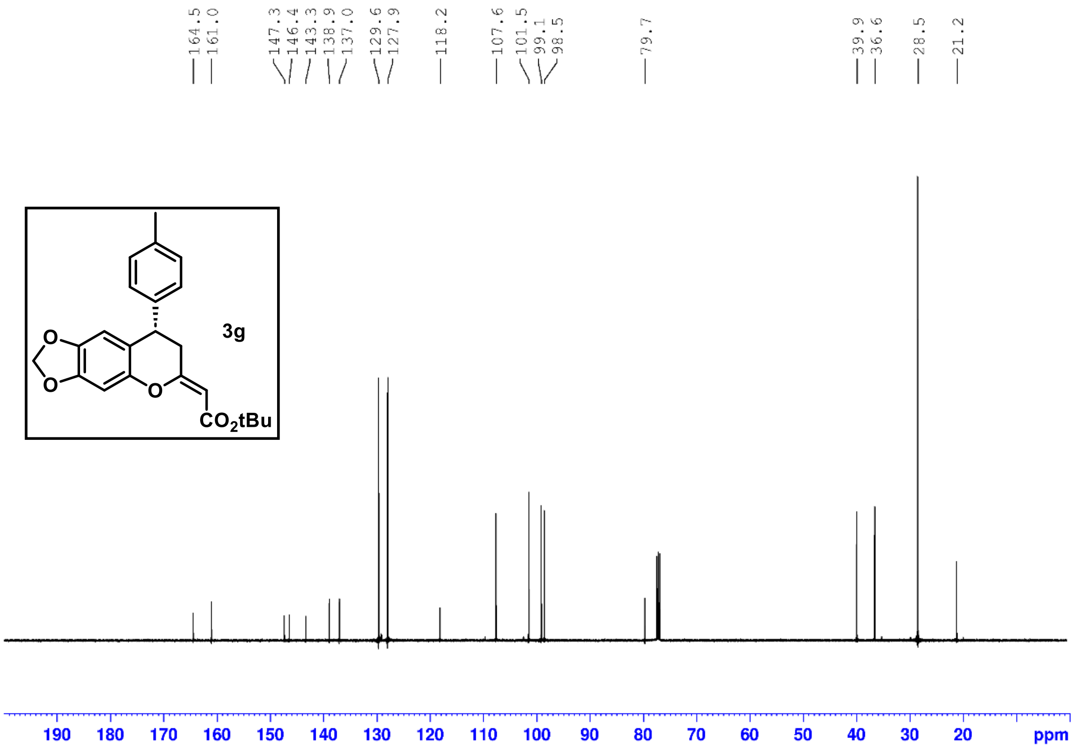
164.5
161.0
147.3
146.4
143.3
138.9
137.0
129.6
127.9
118.2
107.6
101.5
99.1
98.5
79.7
39.9
36.6
28.5
21.2
3g
O
O
O
CO_2tBu
190
180
170
160
150
140
130
120
110
100
90
80
70
60
50
40
30
20
ppm

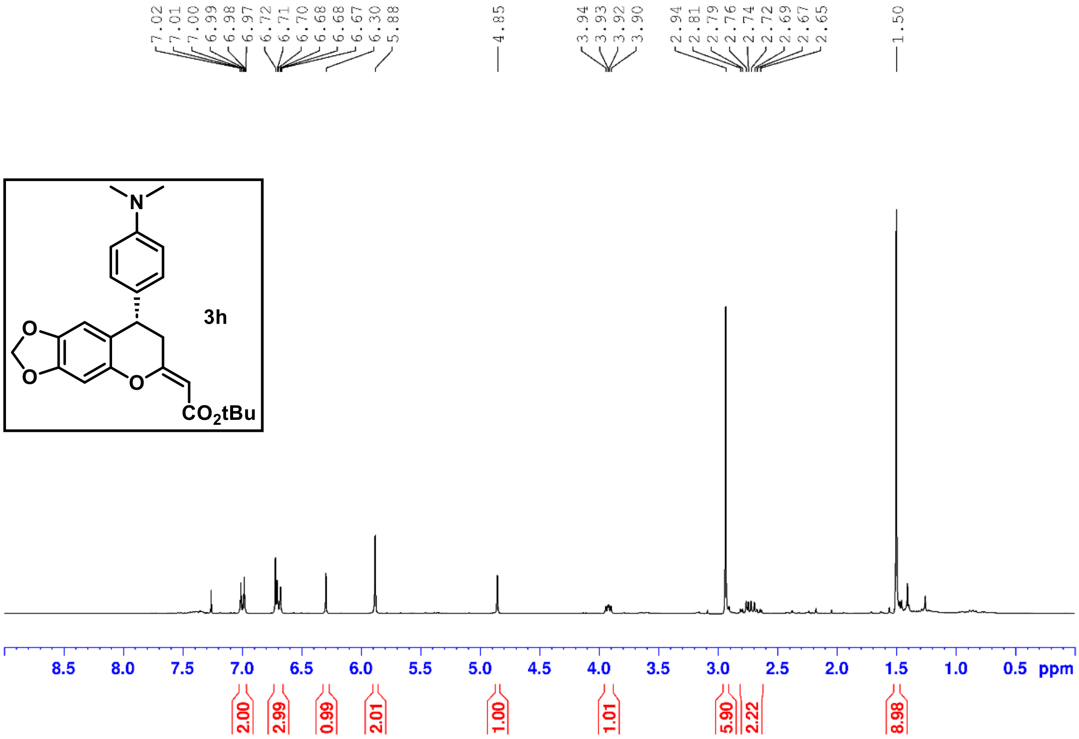
7.02
7.01
7.00
6.99
6.98
6.97
6.72
6.71
6.70
6.68
6.67
6.30
5.88
4.85
3.94
3.93
3.92
3.90
2.94
2.81
2.79
2.76
2.74
2.72
2.69
2.67
2.65
1.50
3h
CO_2tBu
2.00
2.99
0.99
2.01
1.00
1.01
5.90
2.22
8.98
ppm

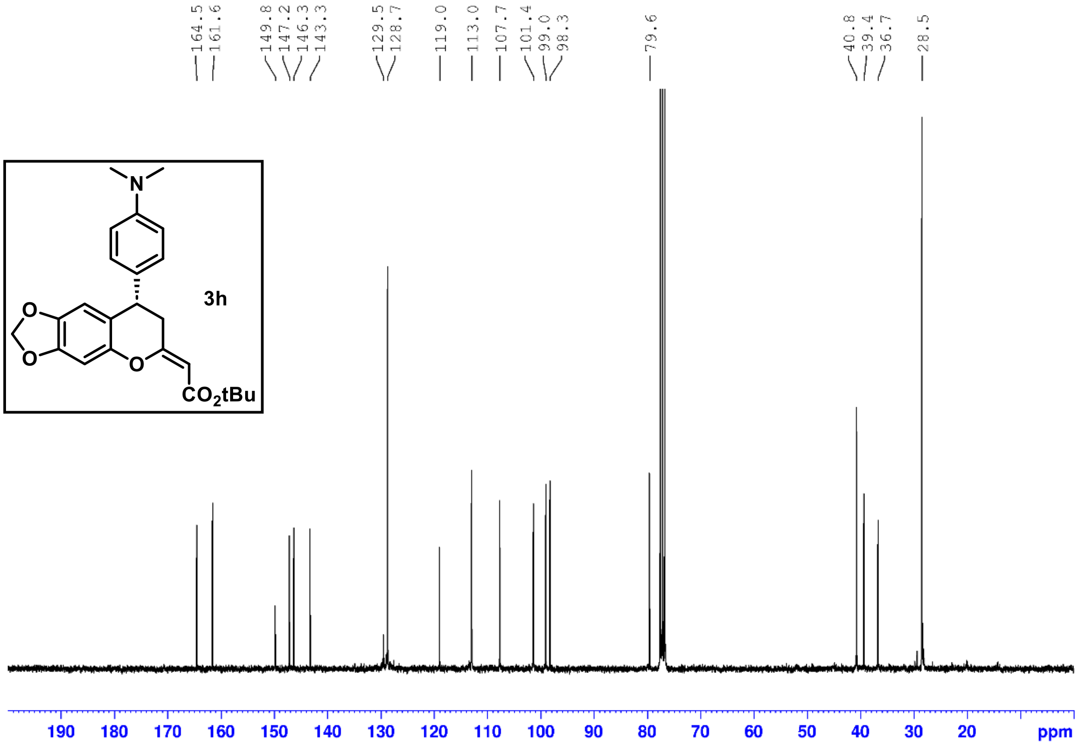
164.5
161.6
149.8
147.2
146.3
143.3
129.5
128.7
119.0
113.0
107.7
101.4
99.0
98.3
79.6
40.8
39.4
36.7
28.5
3h
CO_2tBu
ppm

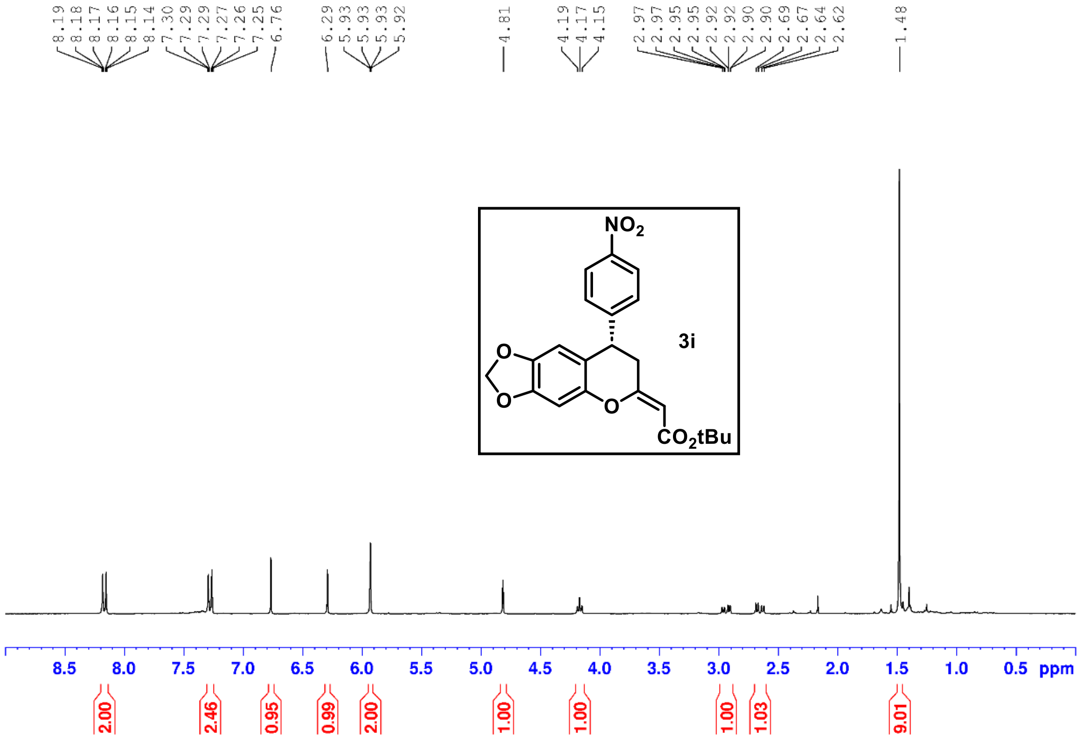
8.19
8.18
8.17
8.16
8.15
8.14
7.30
7.29
7.29
7.27
7.26
7.25
6.76
6.29
5.93
5.93
5.93
5.92
4.81
4.19
4.17
4.15
2.97
2.97
2.95
2.95
2.92
2.92
2.90
2.90
2.69
2.67
2.64
2.62
1.48
NO2
3i
O
O
O
CO2tBu
8.5
8.0
7.5
7.0
6.5
6.0
5.5
5.0
4.5
4.0
3.5
3.0
2.5
2.0
1.5
1.0
0.5
ppm
2.00
2.46
0.95
0.99
2.00
1.00
1.00
1.00
1.03
9.01

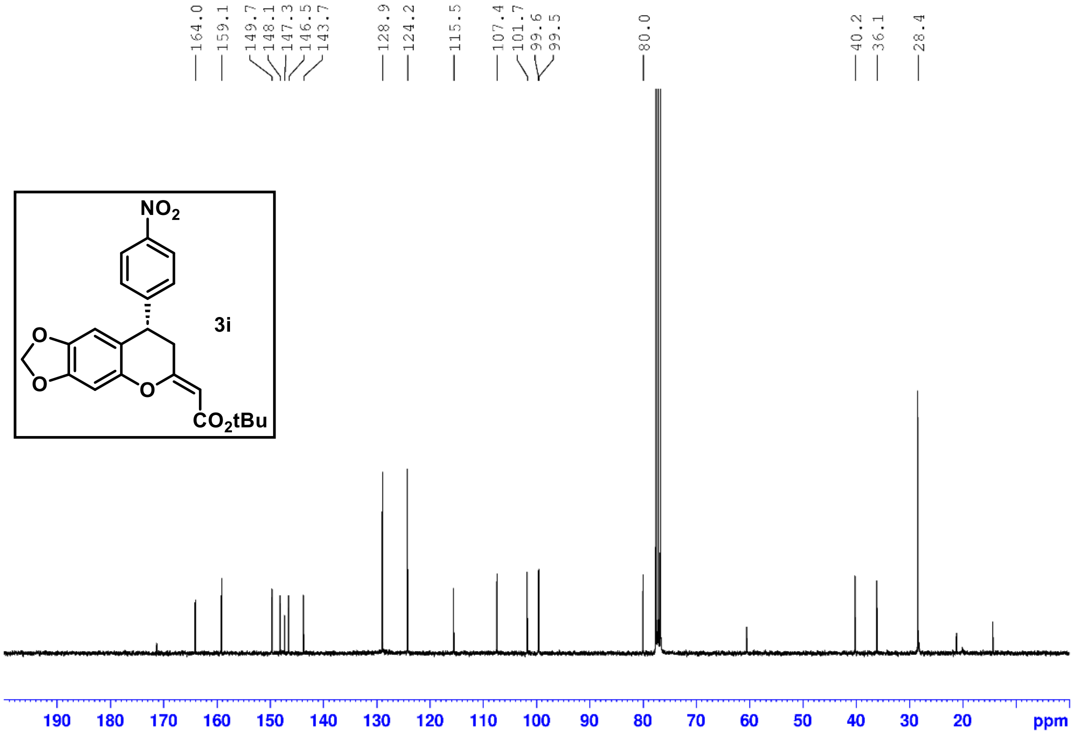
164.0
159.1
149.7
148.1
147.3
146.5
143.7
128.9
124.2
115.5
107.4
101.7
99.6
99.5
80.0
40.2
36.1
28.4
NO2
3i
O
O
O
CO2tBu
190
180
170
160
150
140
130
120
110
100
90
80
70
60
50
40
30
20
ppm

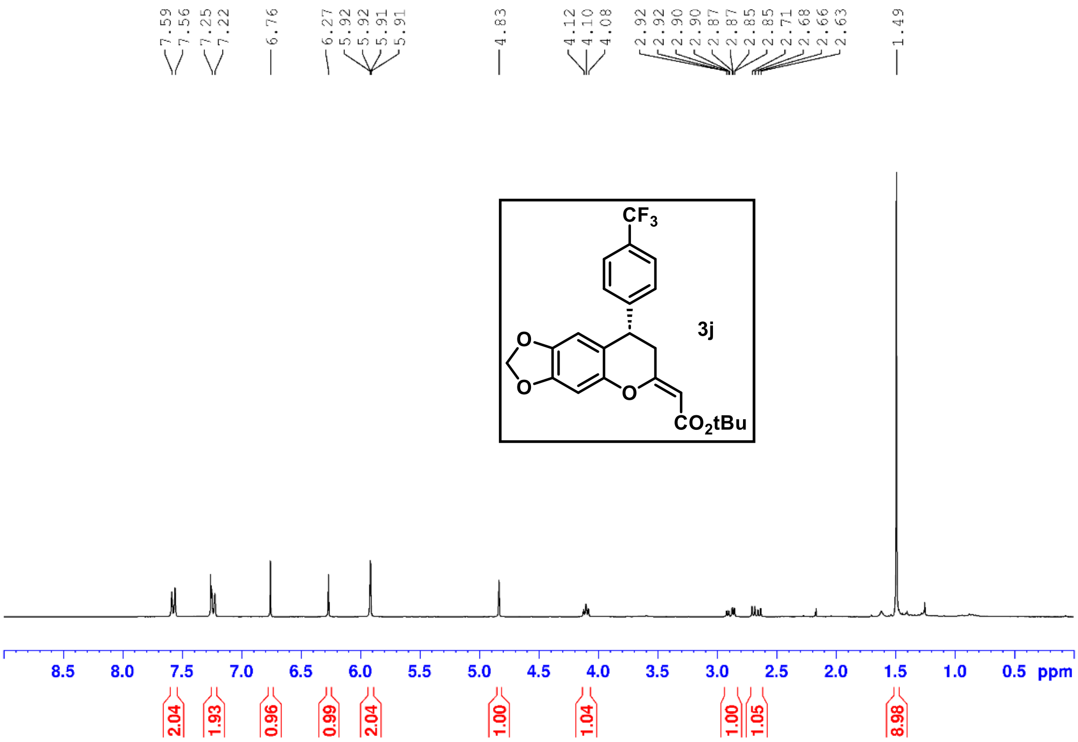
7.59
7.56
7.25
7.22
6.76
6.27
5.92
5.92
5.91
5.91
4.83
4.12
4.10
4.08
2.92
2.92
2.90
2.90
2.87
2.87
2.85
2.85
2.71
2.68
2.66
2.63
1.49
CF_3
3j
O
O
O
CO_2tBu
8.5 8.0 7.5 7.0 6.5 6.0 5.5 5.0 4.5 4.0 3.5 3.0 2.5 2.0 1.5 1.0 0.5 ppm
2.04
1.93
0.96
0.99
2.04
1.00
1.04
1.00
1.05
8.98

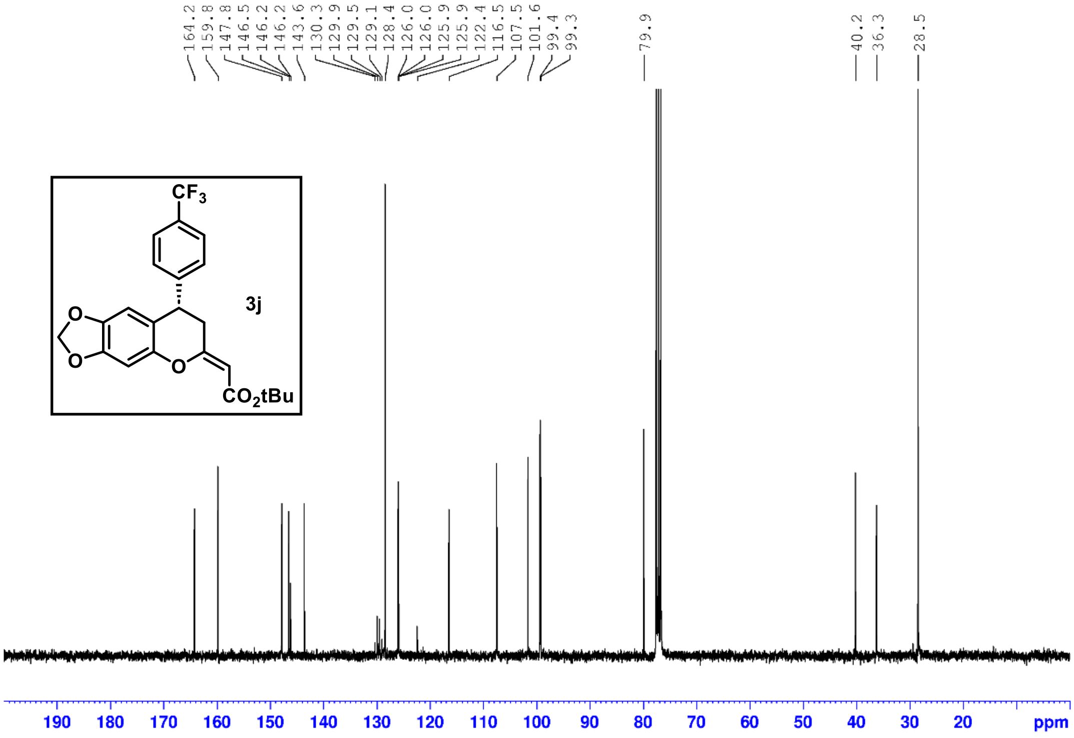
164.2
159.8
147.8
146.5
146.2
146.2
143.6
130.3
129.9
129.5
129.1
128.4
126.0
126.0
125.9
125.9
122.4
116.5
107.5
101.6
99.4
99.3
79.9
40.2
36.3
28.5
CF_3
3j
O
O
O
CO_2tBu
190 180 170 160 150 140 130 120 110 100 90 80 70 60 50 40 30 20 ppm

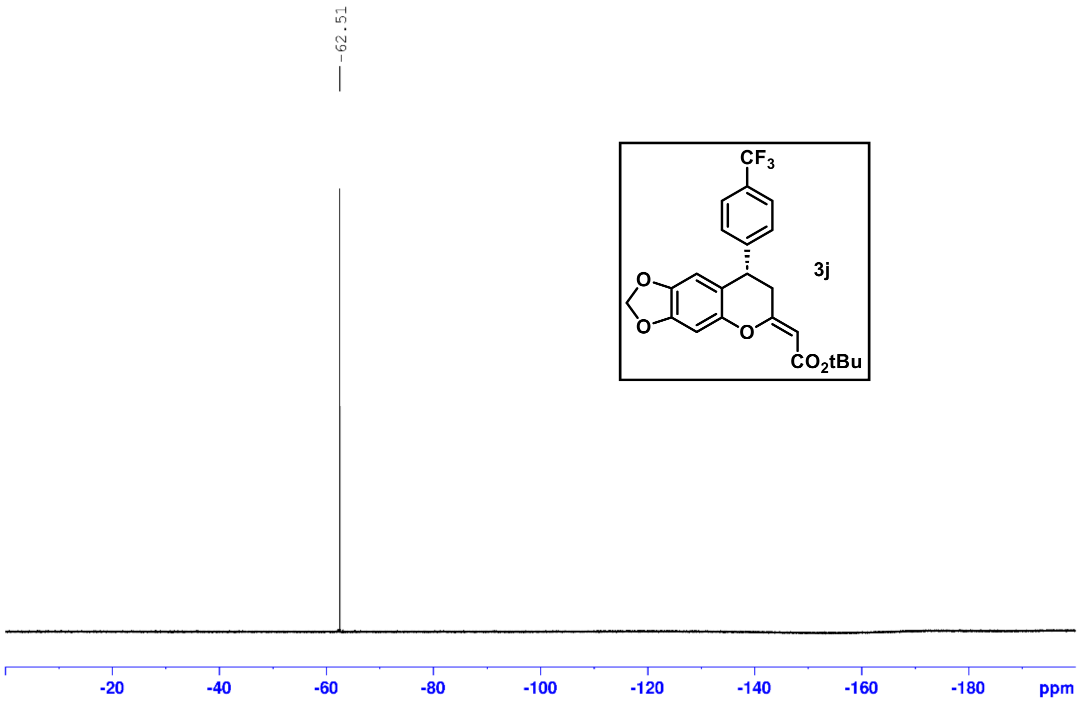
-62.51
CF_3
3j
O
O
O
CO_2tBu
-20
-40
-60
-80
-100
-120
-140
-160
-180
ppm

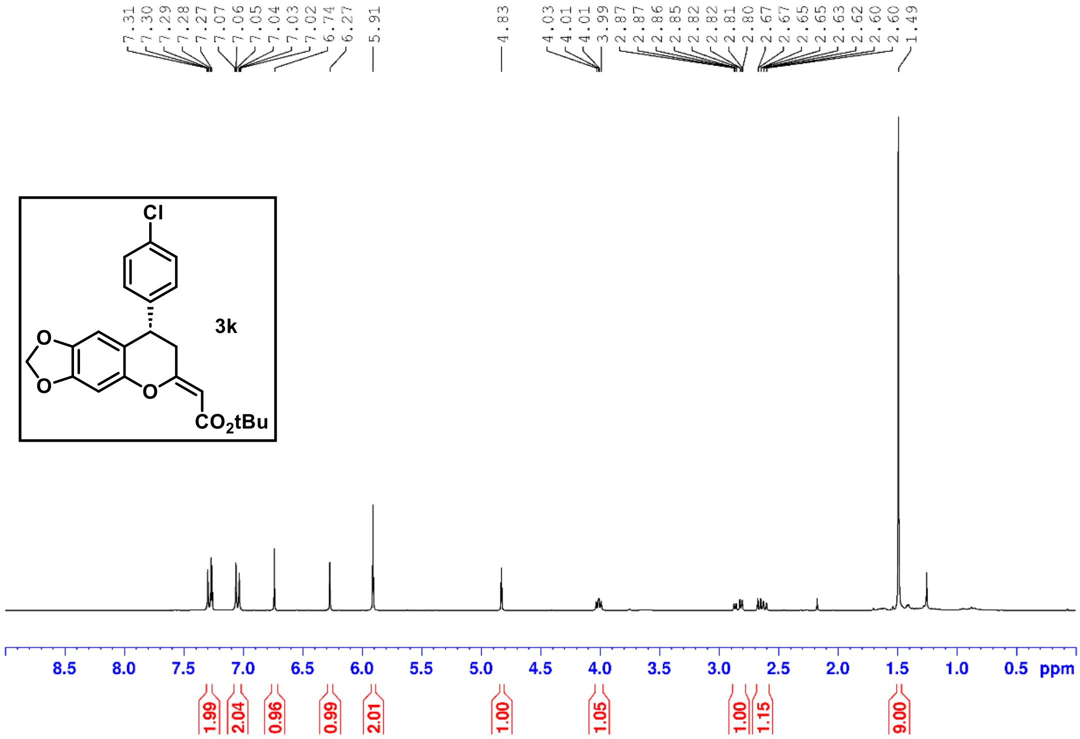
7.31 7.30 7.29 7.28 7.27 7.07 7.06 7.05 7.04 7.03 7.02 6.74 6.27 5.91 4.83 4.03 4.01 4.01 3.99 2.87 2.87 2.86 2.85 2.82 2.82 2.81 2.80 2.67 2.67 2.65 2.65 2.63 2.62 2.60 2.60 1.49
Cl
3k
O
O
O
CO2tBu
8.5 8.0 7.5 7.0 6.5 6.0 5.5 5.0 4.5 4.0 3.5 3.0 2.5 2.0 1.5 1.0 0.5 ppm
1.99 2.04 0.96 0.99 2.01 1.00 1.05 1.00 1.15 9.00

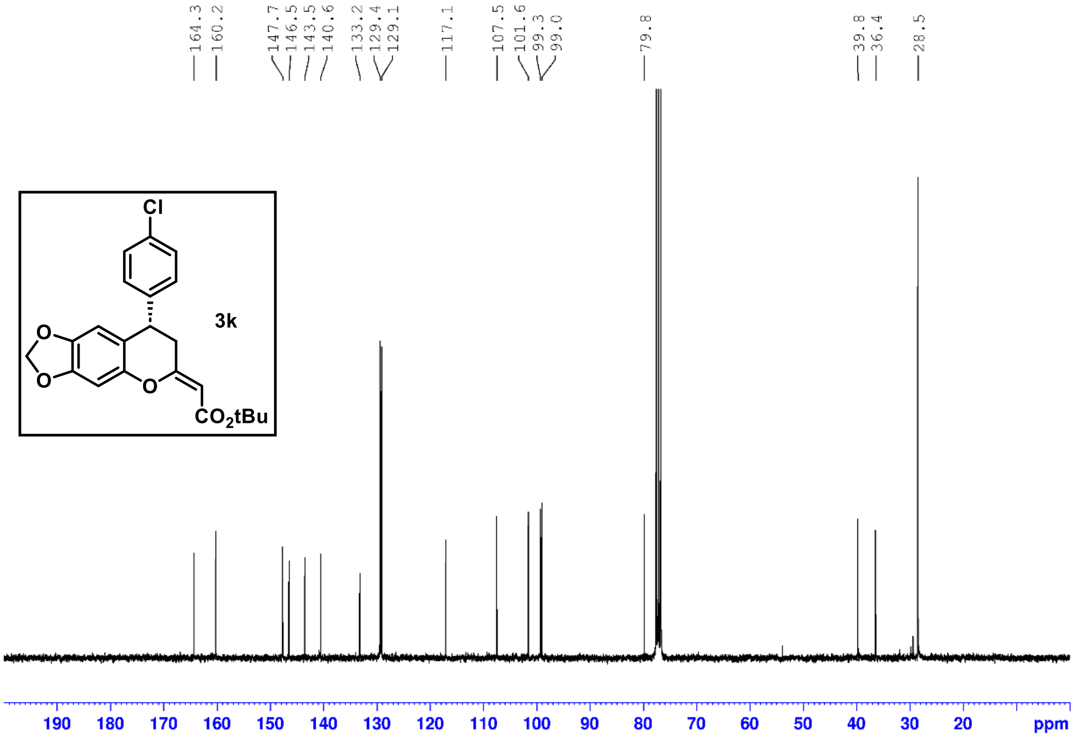
164.3 160.2 147.7 146.5 143.5 140.6 133.2 129.4 129.1 117.1 107.5 101.6 99.3 99.0 79.8 39.8 36.4 28.5
Cl
3k
O
O
O
CO2tBu
190 180 170 160 150 140 130 120 110 100 90 80 70 60 50 40 30 20 ppm

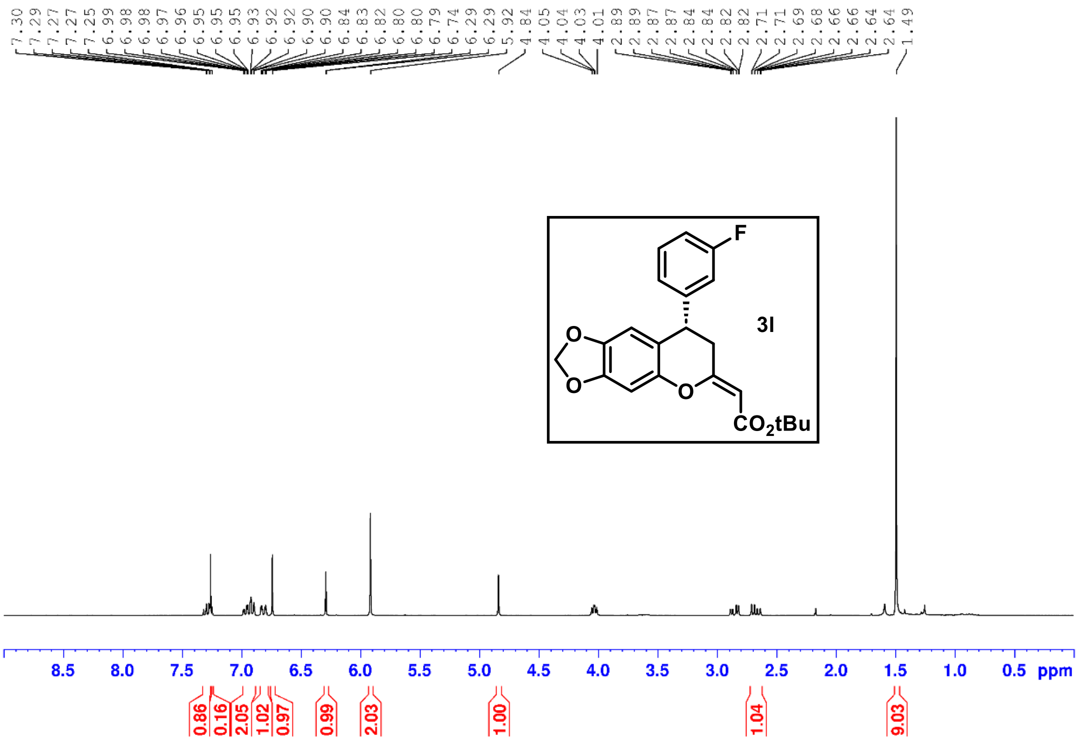
7.30 7.29 7.27 7.27 7.25 6.99 6.98 6.98 6.97 6.96 6.95 6.95 6.95 6.93 6.92 6.92 6.90 6.90 6.84 6.83 6.82 6.80 6.80 6.79 6.74 6.29 6.29 5.92 4.84 4.05 4.04 4.03 4.01 2.89 2.89 2.87 2.87 2.84 2.84 2.82 2.82 2.71 2.71 2.69 2.68 2.66 2.64 2.64 1.49
F
O
O
O
3l
CO2tBu
8.5 8.0 7.5 7.0 6.5 6.0 5.5 5.0 4.5 4.0 3.5 3.0 2.5 2.0 1.5 1.0 0.5 ppm
0.86 0.16 2.05 1.02 0.97 0.99 2.03 1.00 1.04 9.03

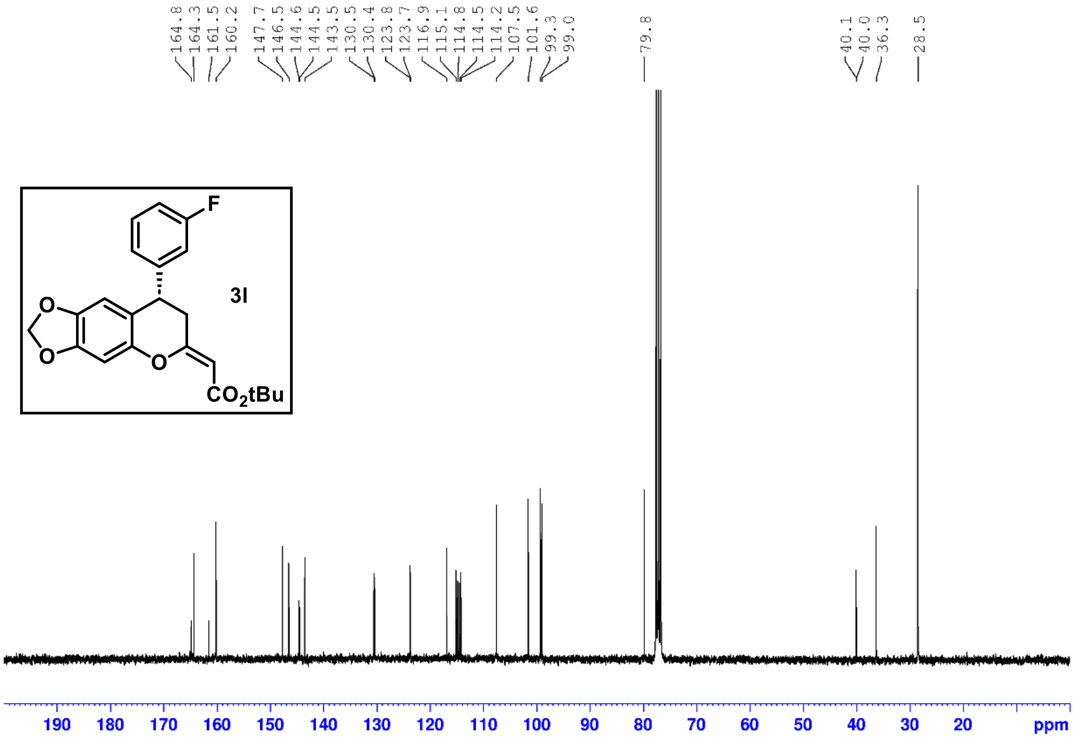
164.8 164.3 161.5 160.2 147.7 146.5 144.6 144.5 143.5 130.5 130.4 123.8 123.7 116.9 115.1 114.8 114.5 114.2 107.5 101.6 99.3 99.0 79.8 40.1 40.0 36.3 28.5
F
O
O
O
3l
CO2tBu
190 180 170 160 150 140 130 120 110 100 90 80 70 60 50 40 30 20 ppm

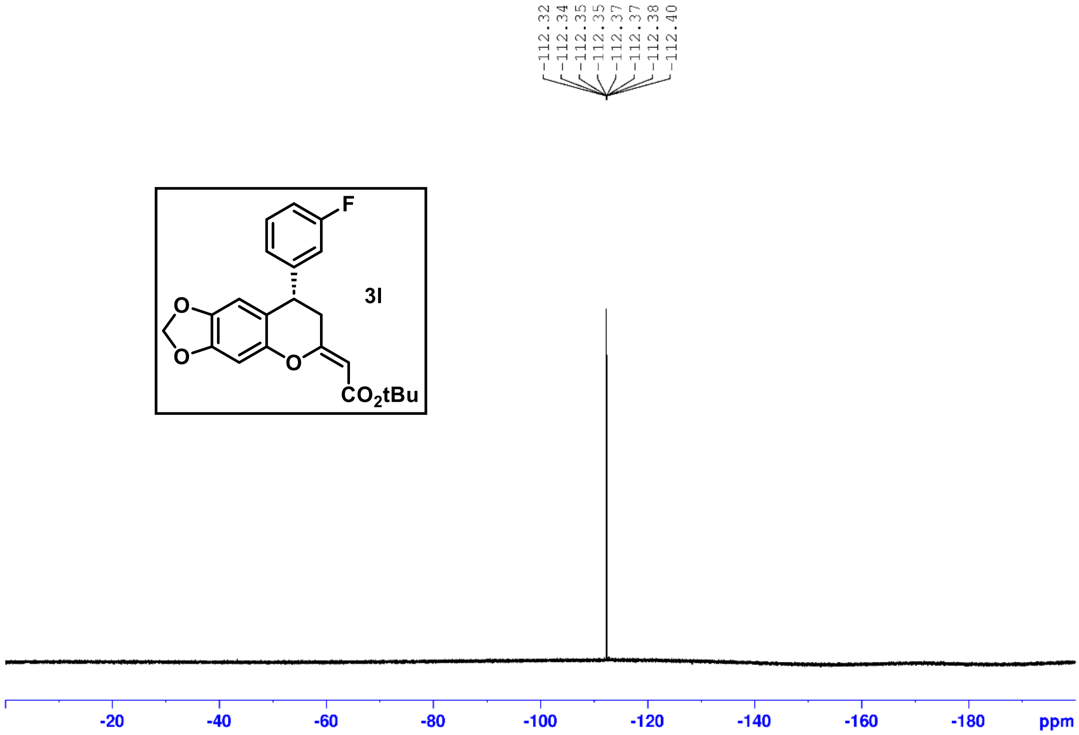
-112.32
-112.34
-112.35
-112.35
-112.37
-112.37
-112.38
-112.40
F
3l
O
O
O
CO2tBu
-20
-40
-60
-80
-100
-120
-140
-160
-180
ppm

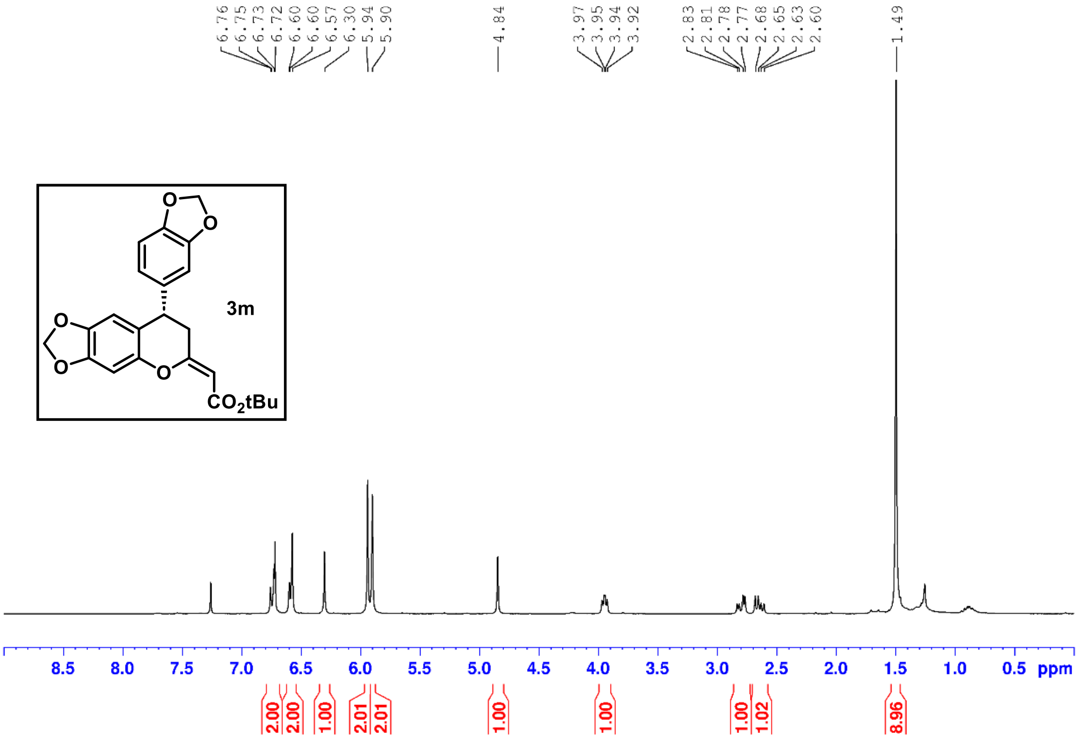
6.76
6.75
6.73
6.72
6.60
6.60
6.57
6.30
5.94
5.90
4.84
3.97
3.95
3.94
3.92
2.83
2.81
2.78
2.77
2.68
2.65
2.63
2.60
1.49
3m
CO_2tBu
8.5 8.0 7.5 7.0 6.5 6.0 5.5 5.0 4.5 4.0 3.5 3.0 2.5 2.0 1.5 1.0 0.5 ppm
2.00
2.00
1.00
2.01
2.01
1.00
1.00
1.00
1.02
8.96

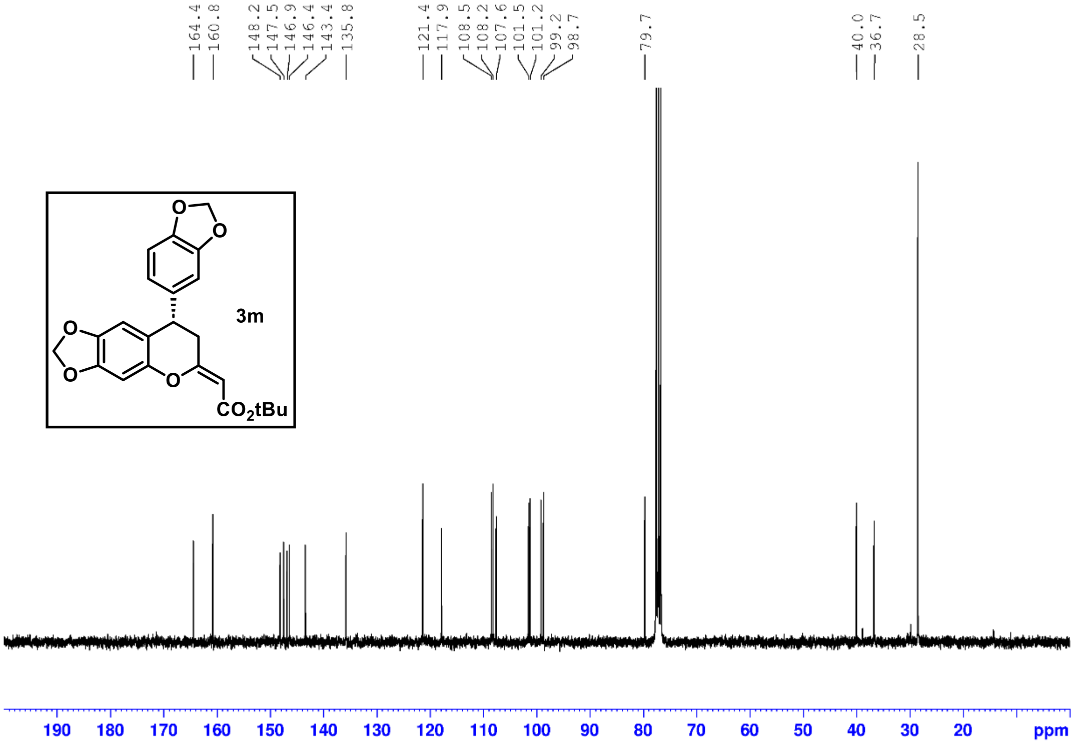
164.4
160.8
148.2
147.5
146.9
146.4
143.4
135.8
121.4
117.9
108.5
108.2
107.6
101.5
101.2
99.2
98.7
79.7
40.0
36.7
28.5
3m
CO_2tBu
190 180 170 160 150 140 130 120 110 100 90 80 70 60 50 40 30 20 ppm

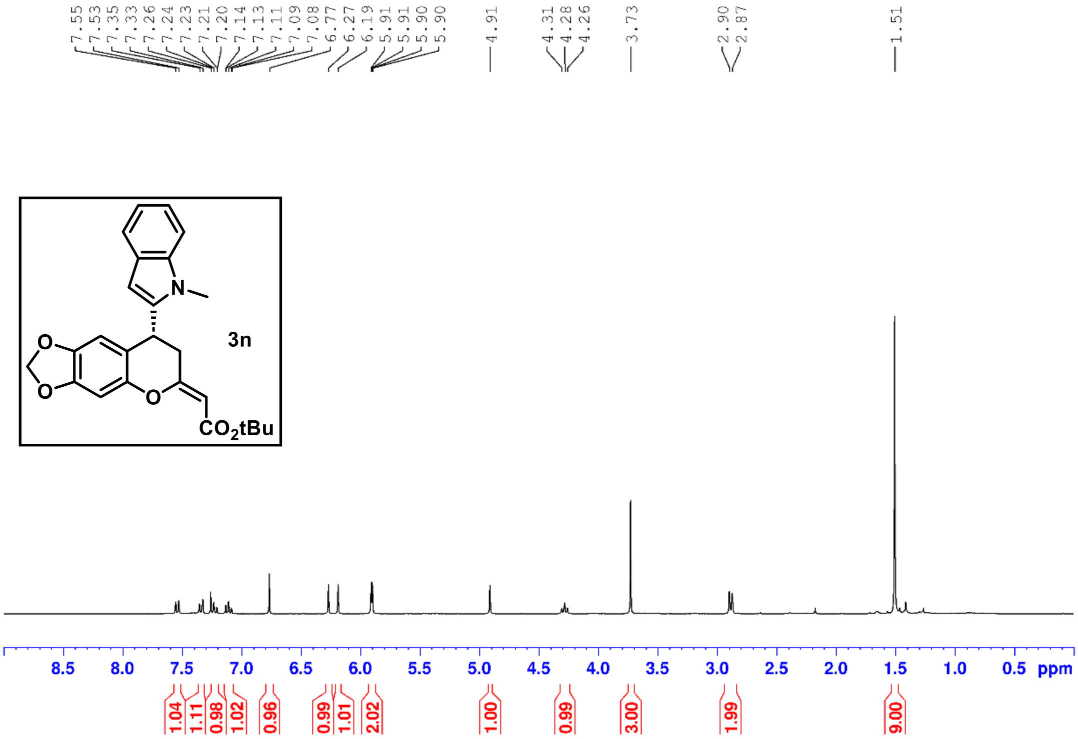
7.55
7.53
7.35
7.33
7.26
7.24
7.23
7.21
7.20
7.14
7.13
7.11
7.09
7.08
6.77
6.27
6.19
5.91
5.91
5.90
5.90
4.91
4.31
4.28
4.26
3.73
2.90
2.87
1.51
N
O
O
O
3n
CO2tBu
8.5
8.0
7.5
7.0
6.5
6.0
5.5
5.0
4.5
4.0
3.5
3.0
2.5
2.0
1.5
1.0
0.5
ppm
1.04
1.11
0.98
1.02
0.96
0.99
1.01
2.02
1.00
0.99
3.00
1.99
9.00

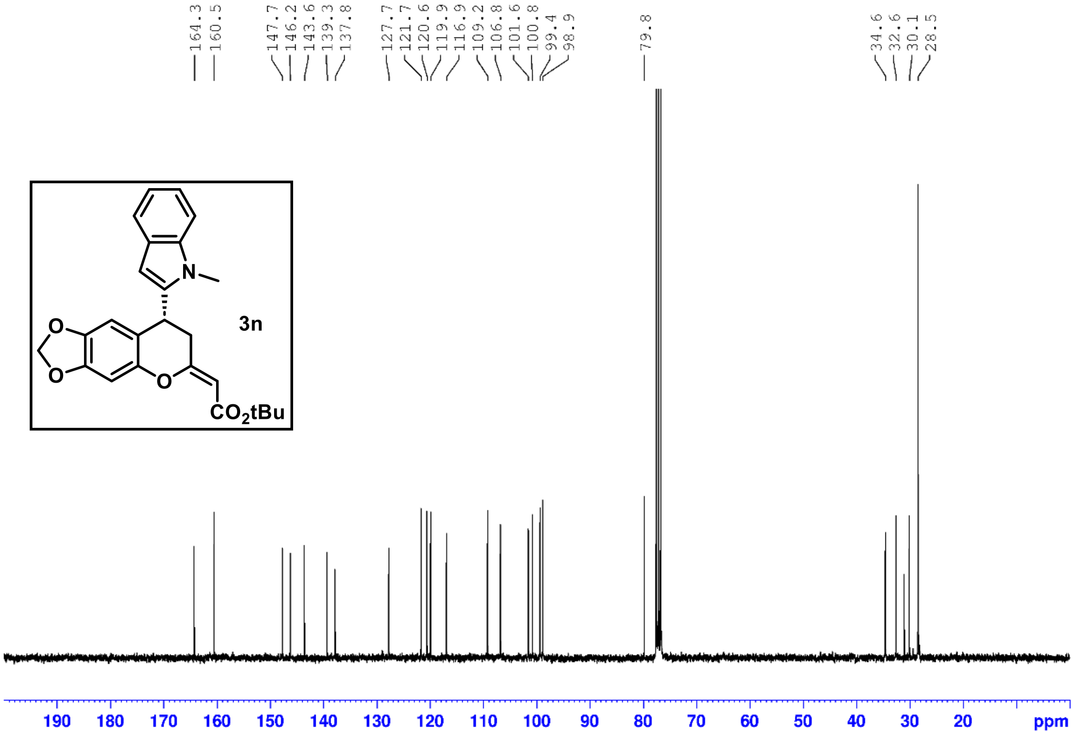
164.3
160.5
147.7
146.2
143.6
139.3
137.8
127.7
121.7
120.6
119.9
116.9
109.2
106.8
101.6
100.8
99.4
98.9
79.8
34.6
32.6
30.1
28.5
N
O
O
O
3n
CO2tBu
190
180
170
160
150
140
130
120
110
100
90
80
70
60
50
40
30
20
ppm

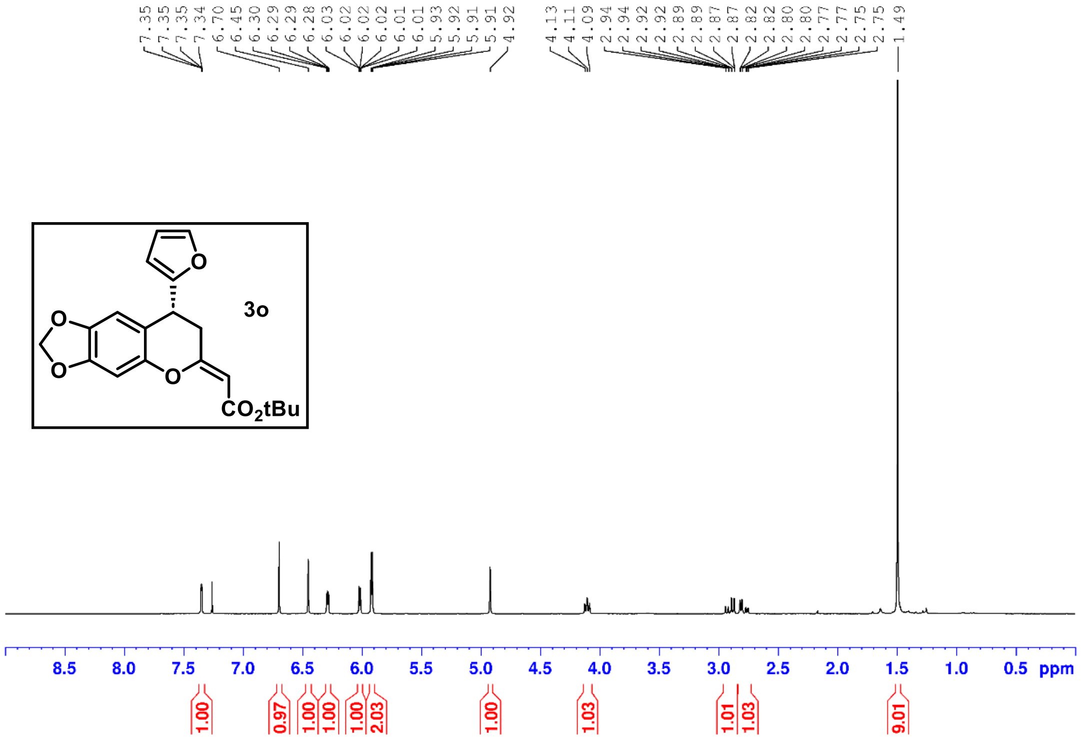
7.35 7.35 7.35 7.34 6.70 6.45 6.30 6.29 6.29 6.28 6.03 6.02 6.02 6.02 6.01 6.01 5.93 5.92 5.91 5.91 4.92 4.13 4.11 4.09 2.94 2.94 2.92 2.92 2.89 2.89 2.87 2.87 2.82 2.82 2.80 2.80 2.77 2.77 2.75 2.75 1.49
3o
CO_2tBu
8.5 8.0 7.5 7.0 6.5 6.0 5.5 5.0 4.5 4.0 3.5 3.0 2.5 2.0 1.5 1.0 0.5 ppm
1.00 0.97 1.00 1.00 1.00 2.03 1.00 1.03 1.01 1.03 9.01

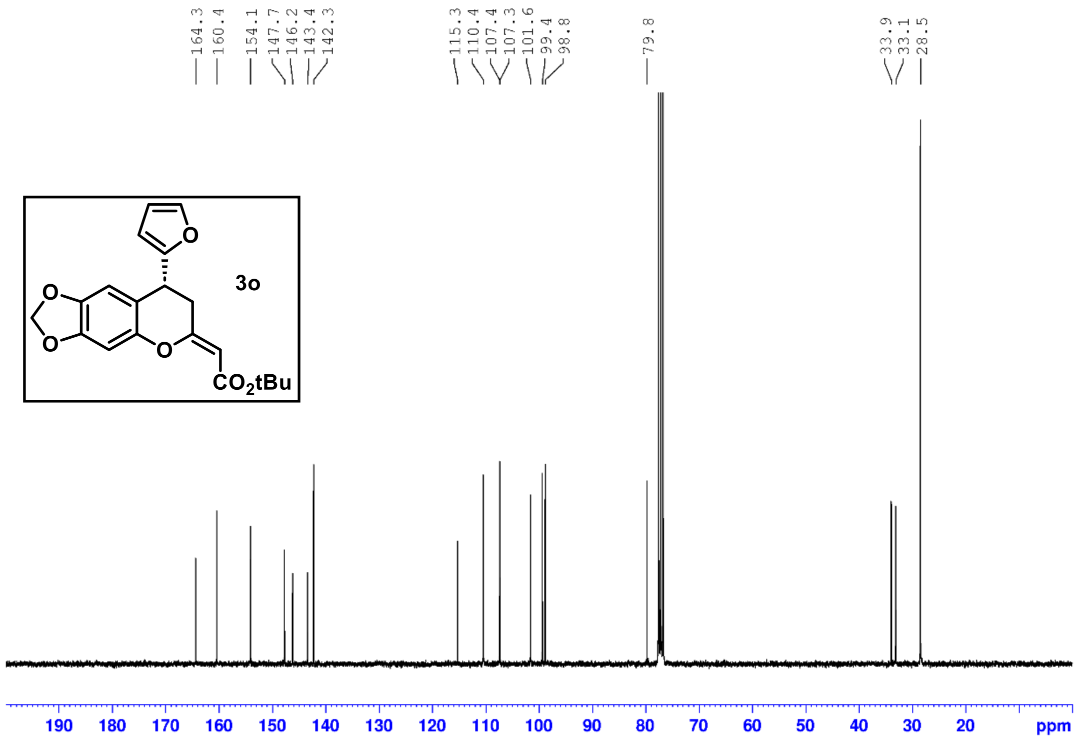
164.3 160.4 154.1 147.7 146.2 143.4 142.3 115.3 110.4 107.4 107.3 101.6 99.4 98.8 79.8 33.9 33.1 28.5
3o
CO_2tBu
190 180 170 160 150 140 130 120 110 100 90 80 70 60 50 40 30 20 ppm

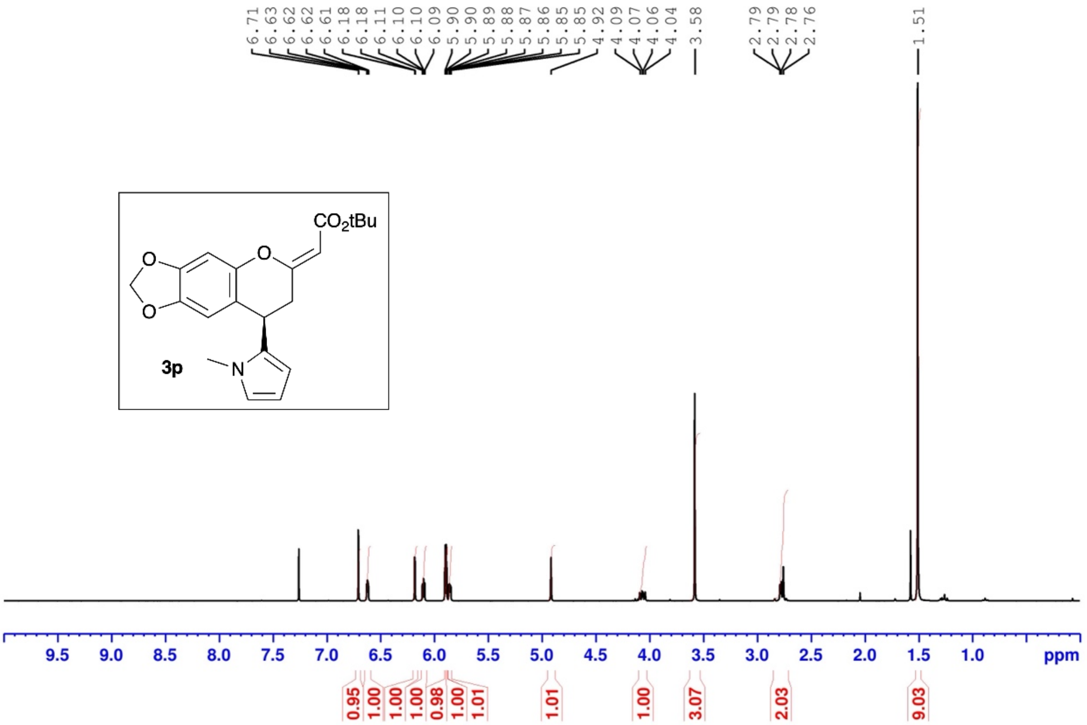
6.71
6.63
6.62
6.61
6.18
6.18
6.11
6.10
6.10
6.09
5.90
5.90
5.89
5.88
5.87
5.86
5.85
5.85
4.92
4.09
4.07
4.06
4.04
3.58
2.79
2.79
2.78
2.76
1.51
CO2tBu
O
O
O
3p
N
9.5 9.0 8.5 8.0 7.5 7.0 6.5 6.0 5.5 5.0 4.5 4.0 3.5 3.0 2.5 2.0 1.5 1.0 ppm
0.95
1.00
1.00
1.00
0.98
1.00
1.01
1.01
1.00
3.07
2.03
9.03

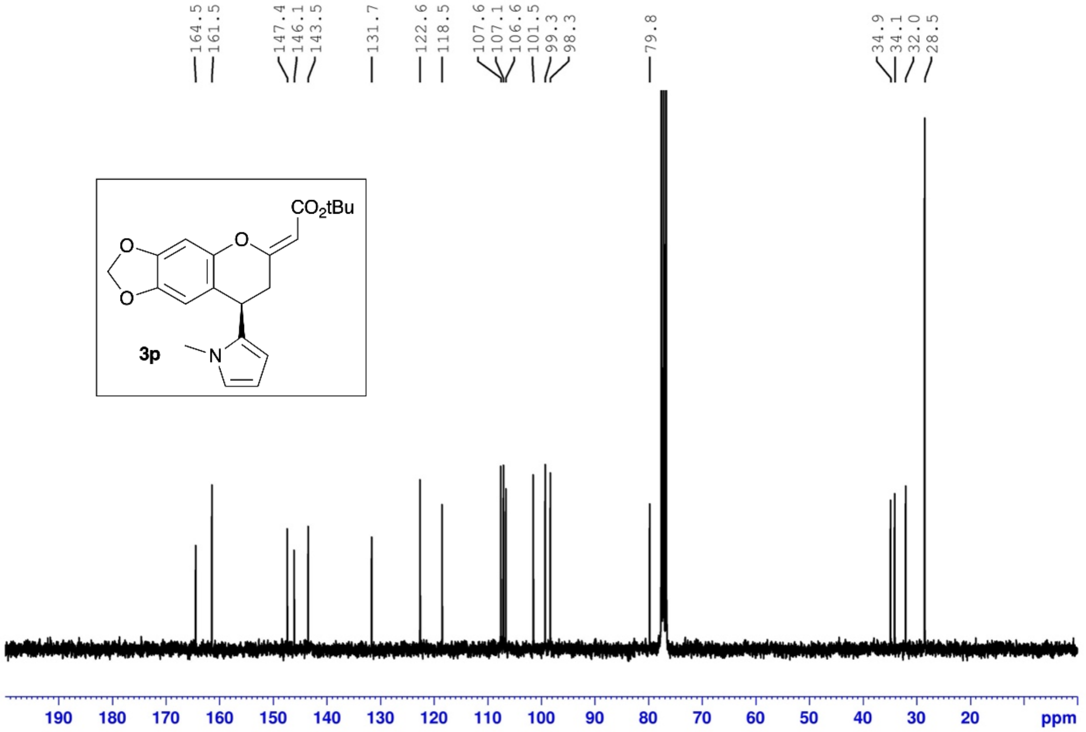
164.5
161.5
147.4
146.1
143.5
131.7
122.6
118.5
107.6
107.1
106.6
101.5
99.3
98.3
79.8
34.9
34.1
32.0
28.5
CO2tBu
O
O
O
3p
N
190 180 170 160 150 140 130 120 110 100 90 80 70 60 50 40 30 20 ppm

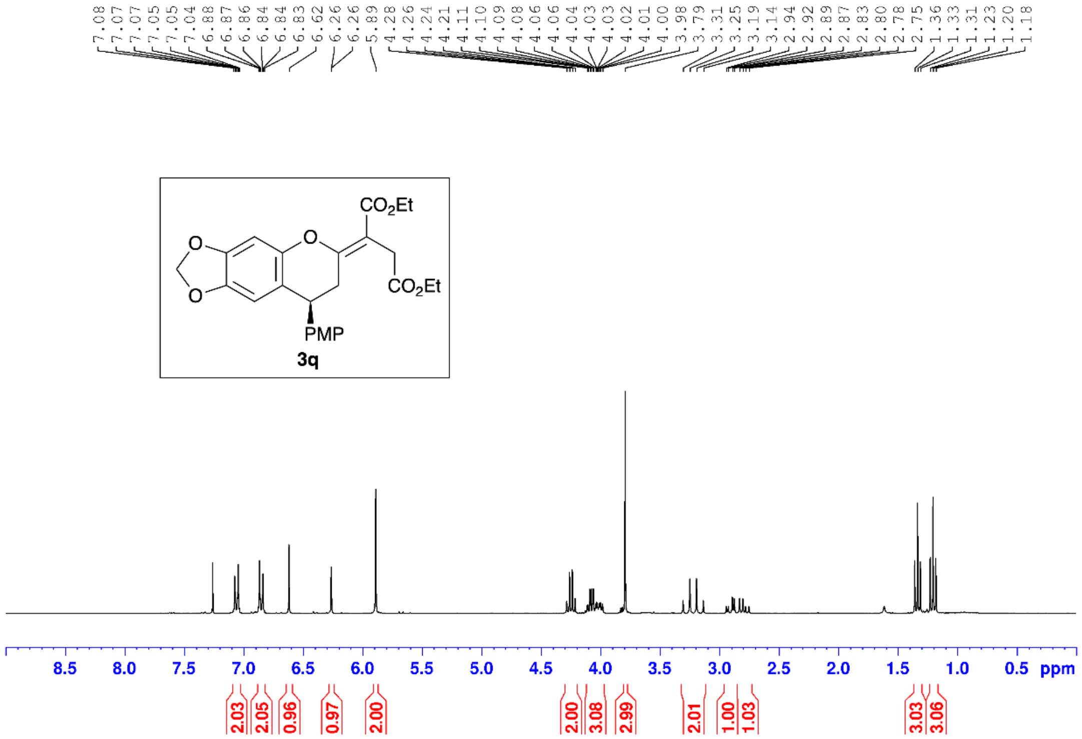
7.08 7.07 7.07 7.05 7.05 7.04 6.88 6.87 6.86 6.84 6.84 6.83 6.62 6.26 6.26 5.89 4.28 4.26 4.24 4.21 4.11 4.10 4.09 4.08 4.06 4.06 4.04 4.03 4.03 4.02 4.01 4.00 3.98 3.79 3.31 3.25 3.19 3.14 2.94 2.92 2.89 2.87 2.83 2.80 2.78 2.75 1.36 1.33 1.31 1.23 1.20 1.18
CO_2Et
CO_2Et
PMP
3q
8.5 8.0 7.5 7.0 6.5 6.0 5.5 5.0 4.5 4.0 3.5 3.0 2.5 2.0 1.5 1.0 0.5 ppm
2.03 2.05 0.96 0.97 2.00 2.00 3.08 2.99 2.01 1.00 1.03 3.03 3.06

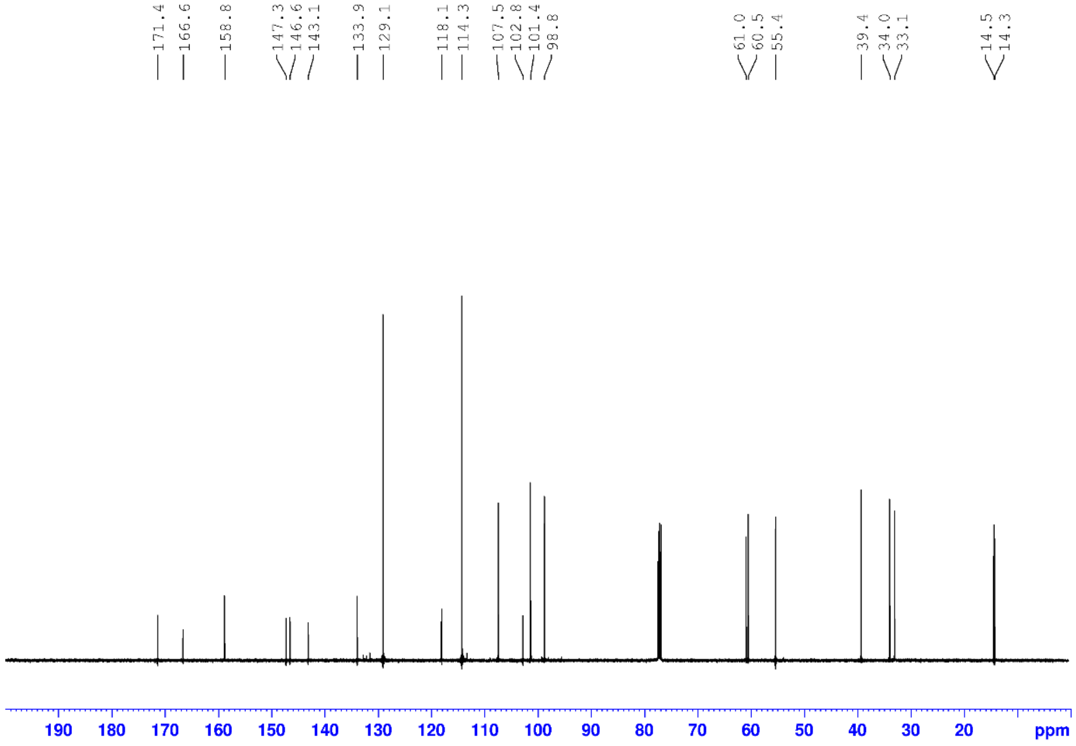
171.4 166.6 158.8 147.3 146.6 143.1 133.9 129.1 118.1 114.3 107.5 102.8 101.4 98.8 61.0 60.5 55.4 39.4 34.0 33.1 14.5 14.3
190 180 170 160 150 140 130 120 110 100 90 80 70 60 50 40 30 20 ppm

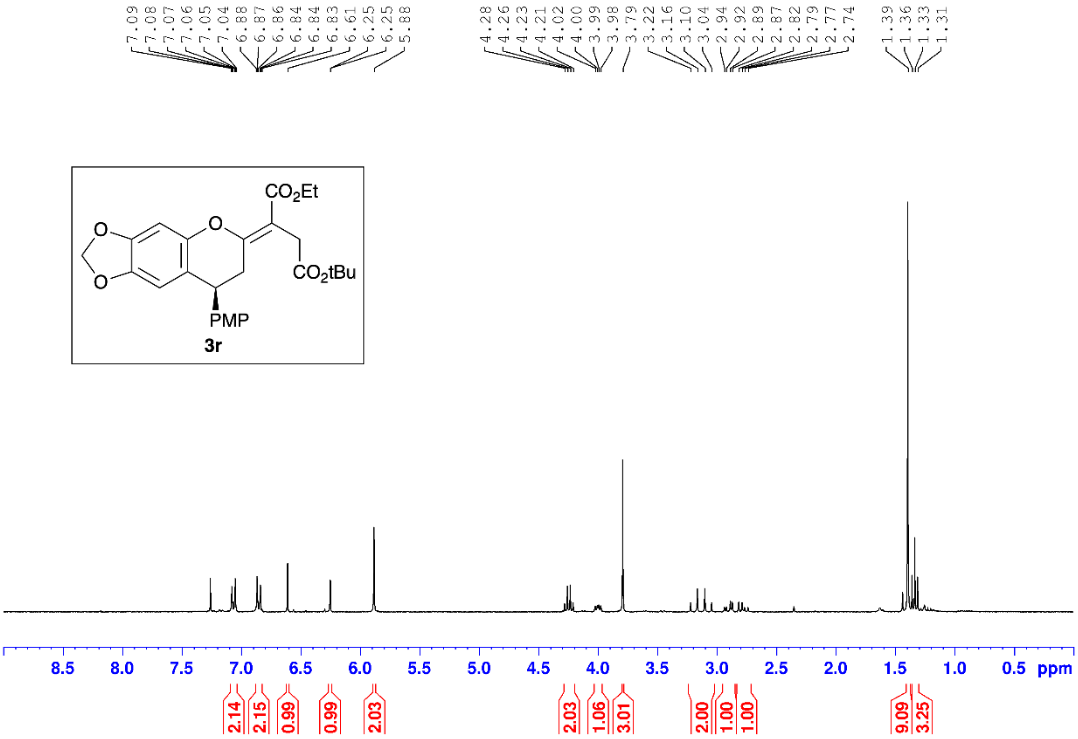

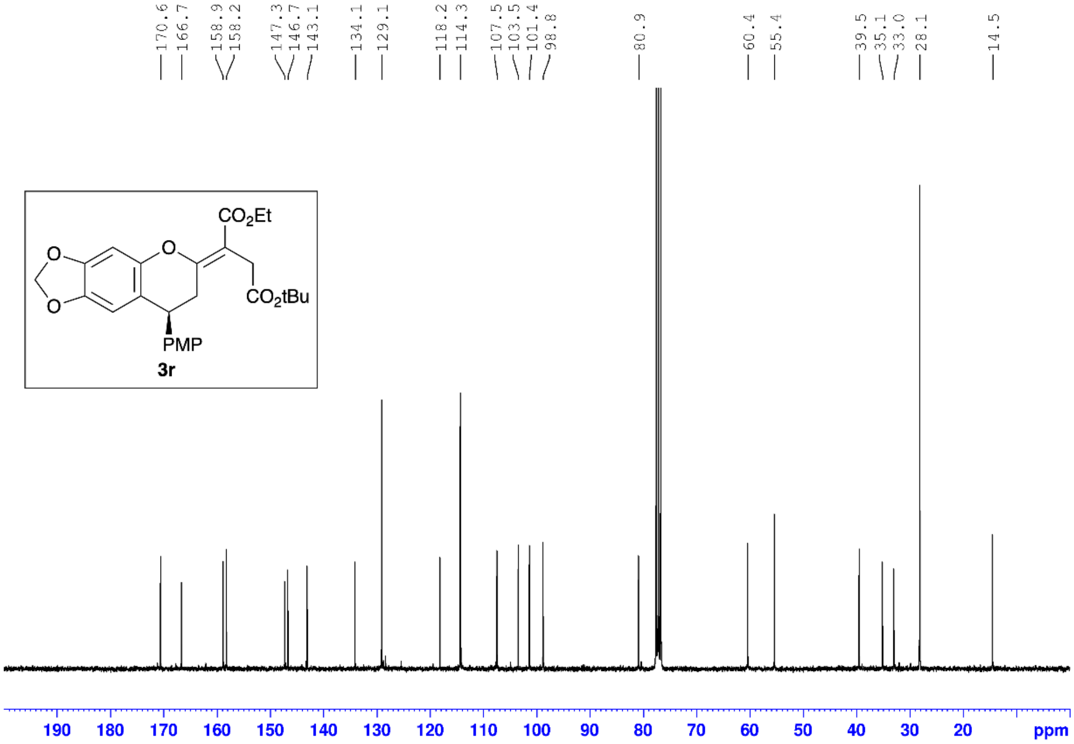

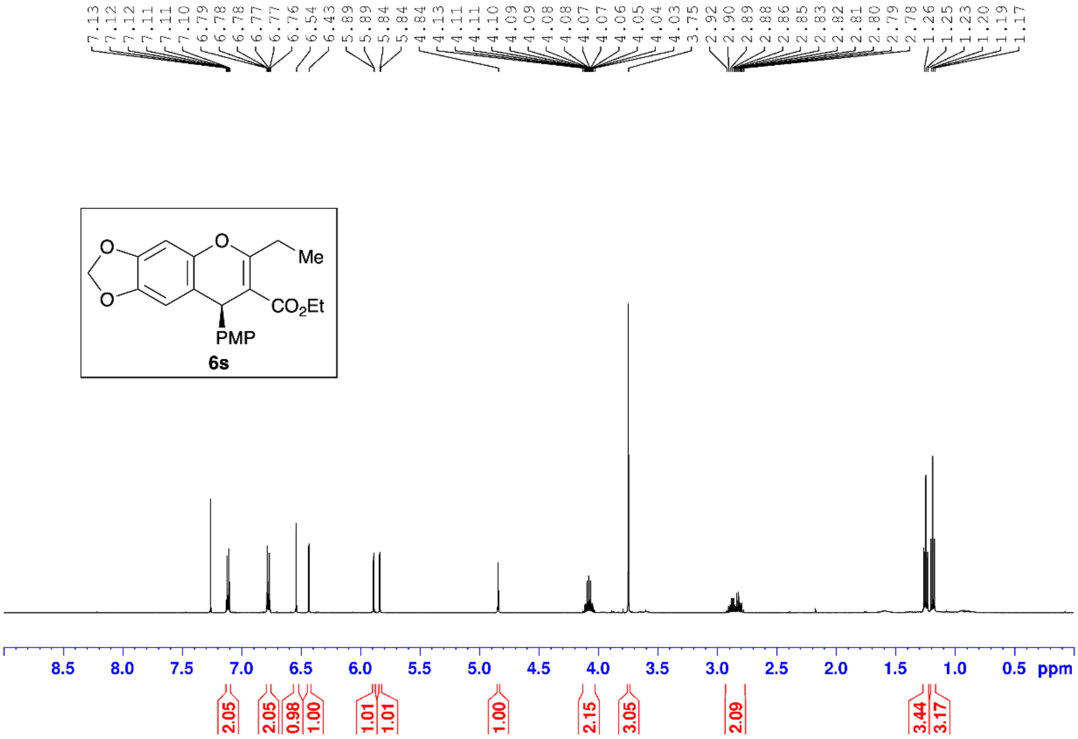
7.13
7.12
7.12
7.11
7.11
7.10
6.79
6.78
6.78
6.77
6.77
6.76
6.54
6.43
5.89
5.89
5.84
5.84
4.84
4.13
4.11
4.11
4.10
4.09
4.09
4.08
4.08
4.07
4.07
4.06
4.05
4.04
4.03
3.75
2.92
2.90
2.89
2.88
2.86
2.85
2.83
2.82
2.81
2.80
2.79
2.78
1.26
1.25
1.23
1.20
1.19
1.17
O
O
O
Me
CO_2Et
PMP
6s
8.5
8.0
7.5
7.0
6.5
6.0
5.5
5.0
4.5
4.0
3.5
3.0
2.5
2.0
1.5
1.0
0.5
ppm
2.05
2.05
0.98
1.00
1.01
1.01
1.00
2.15
3.05
2.09
3.44
3.17

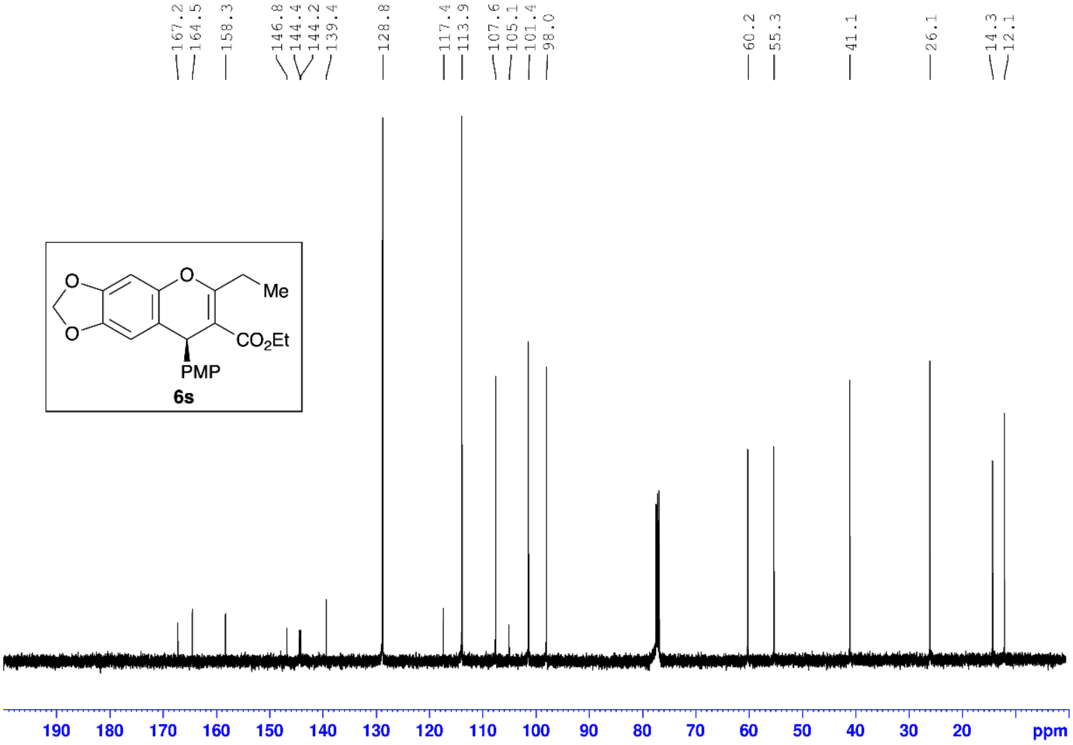
167.2
164.5
158.3
146.8
144.4
144.2
139.4
128.8
117.4
113.9
107.6
105.1
101.4
98.0
60.2
55.3
41.1
26.1
14.3
12.1
O
O
O
Me
CO_2Et
PMP
6s
190
180
170
160
150
140
130
120
110
100
90
80
70
60
50
40
30
20
ppm

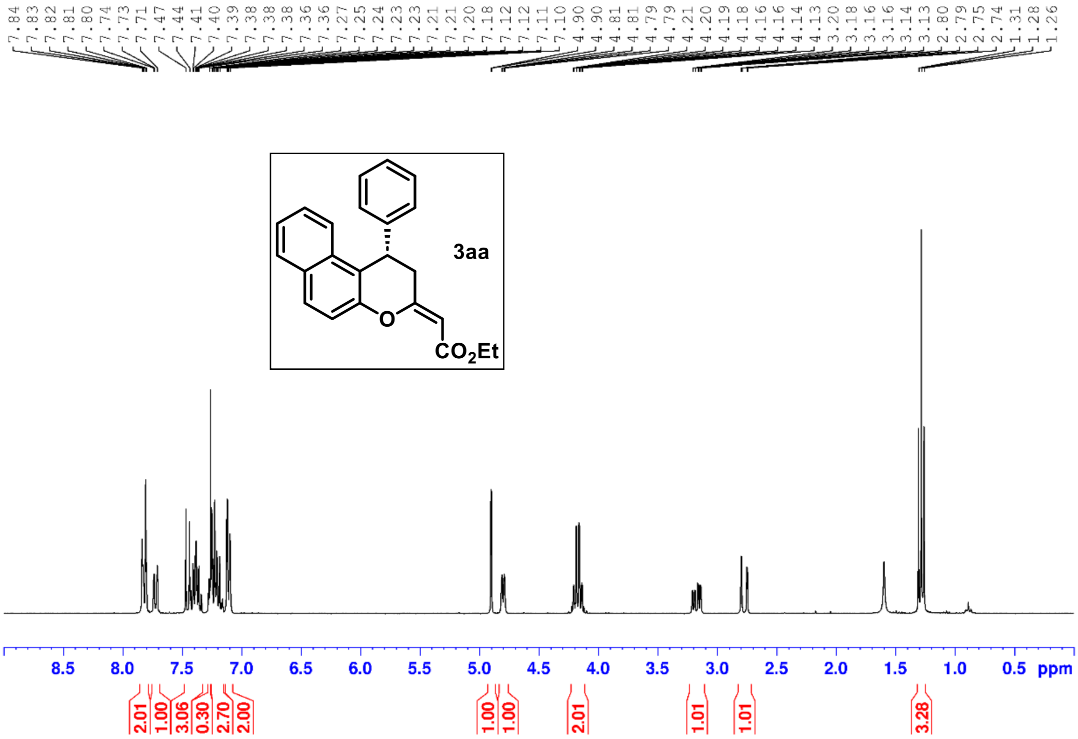
7.84 7.83 7.82 7.81 7.80 7.74 7.73 7.71 7.47 7.44 7.41 7.40 7.39 7.38 7.38 7.38 7.36 7.36 7.27 7.25 7.24 7.23 7.23 7.21 7.21 7.20 7.18 7.12 7.12 7.11 7.10 4.90 4.90 4.81 4.79 4.79 4.21 4.20 4.19 4.18 4.16 4.16 4.14 4.13 3.20 3.18 3.16 3.14 3.13 2.80 2.79 2.75 2.74 1.31 1.28 1.26
3aa
CO2Et
O
8.5 8.0 7.5 7.0 6.5 6.0 5.5 5.0 4.5 4.0 3.5 3.0 2.5 2.0 1.5 1.0 0.5 ppm
2.01 1.00 3.06 0.30 2.70 2.00 1.00 1.00 2.01 1.01 1.01 3.28

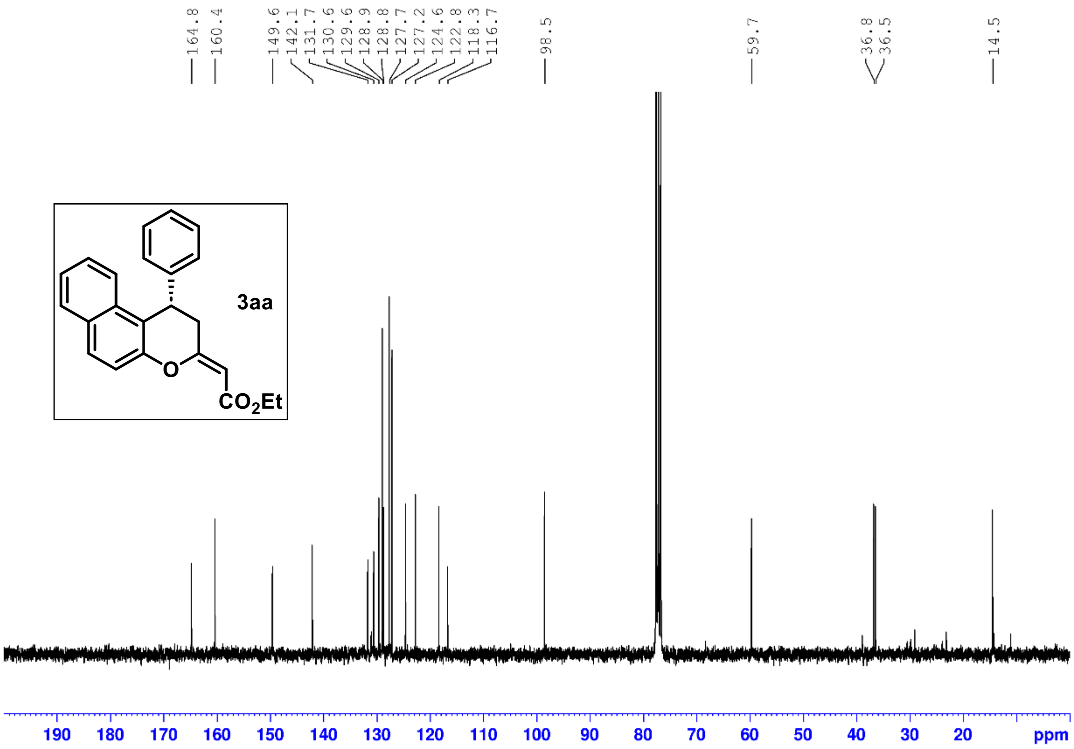
164.8 160.4 149.6 142.1 131.7 130.6 129.6 128.9 128.8 127.7 127.2 124.6 122.8 118.3 116.7 98.5 59.7 36.8 36.5 14.5
3aa
CO2Et
O
190 180 170 160 150 140 130 120 110 100 90 80 70 60 50 40 30 20 ppm

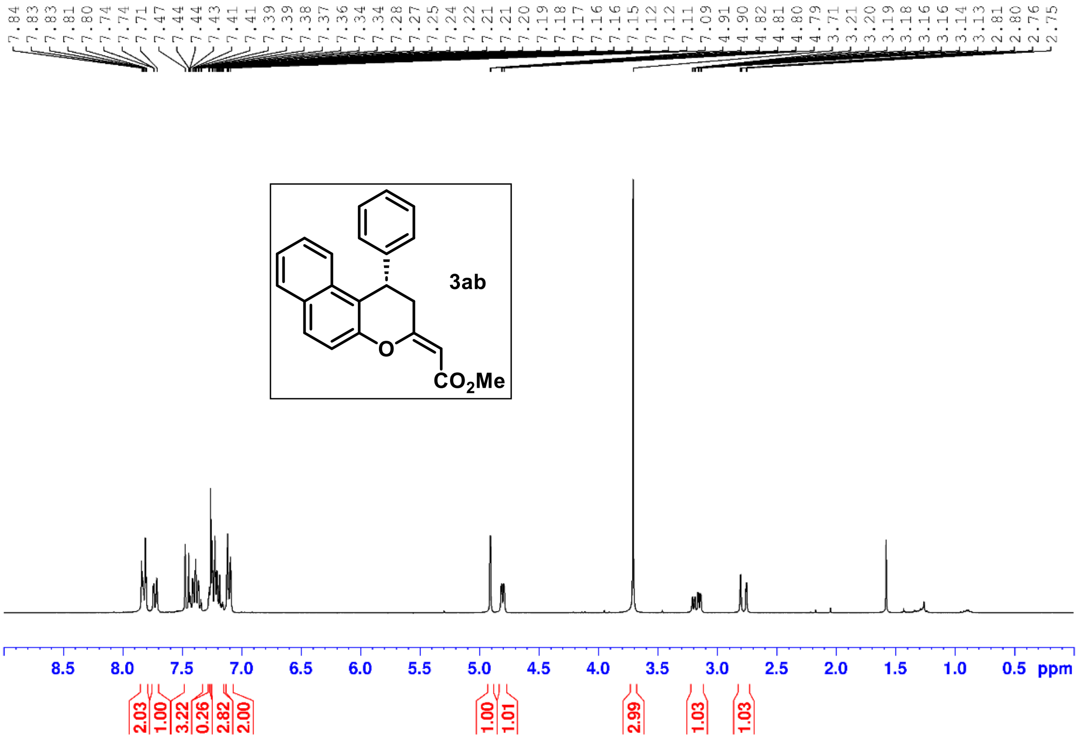
7.84 7.83 7.83 7.81 7.80 7.74 7.74 7.74 7.71 7.47 7.44 7.44 7.43 7.41 7.41 7.39 7.39 7.38 7.37 7.36 7.34 7.34 7.28 7.27 7.25 7.24 7.22 7.21 7.21 7.20 7.19 7.18 7.18 7.17 7.16 7.16 7.15 7.12 7.12 7.11 7.09 4.91 4.90 4.82 4.81 4.80 4.79 3.71 3.21 3.20 3.19 3.18 3.16 3.14 3.13 2.81 2.80 2.76 2.75
3ab
O
CO2Me
8.5 8.0 7.5 7.0 6.5 6.0 5.5 5.0 4.5 4.0 3.5 3.0 2.5 2.0 1.5 1.0 0.5 ppm
2.03 1.00 3.22 0.26 2.82 2.00 1.00 1.01 2.99 1.03 1.03

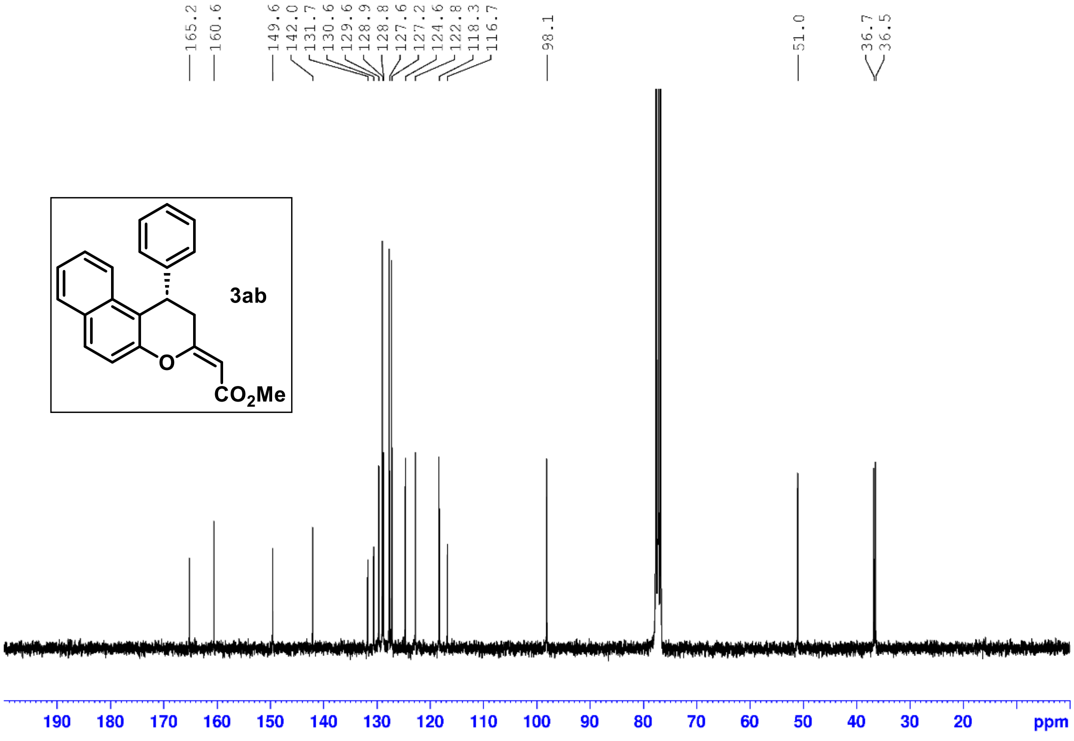
165.2 160.6 149.6 142.0 131.7 130.6 129.6 128.9 128.8 127.6 127.2 124.6 122.8 118.3 116.7 98.1 51.0 36.7 36.5
3ab
O
CO2Me
190 180 170 160 150 140 130 120 110 100 90 80 70 60 50 40 30 20 ppm

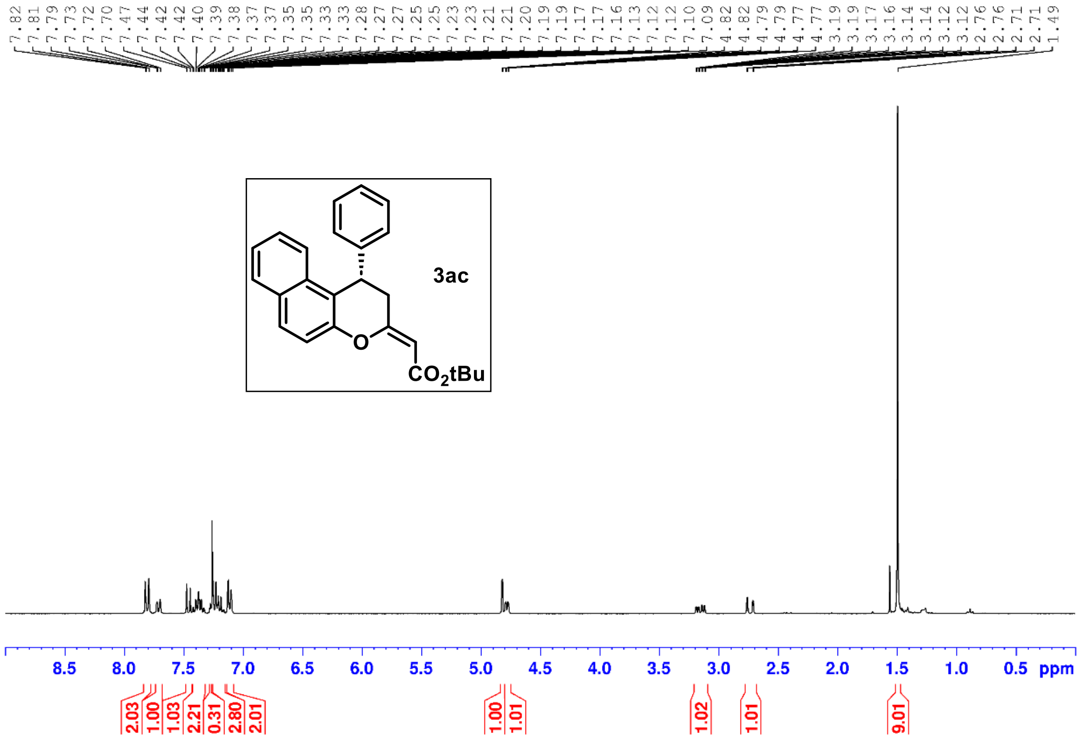

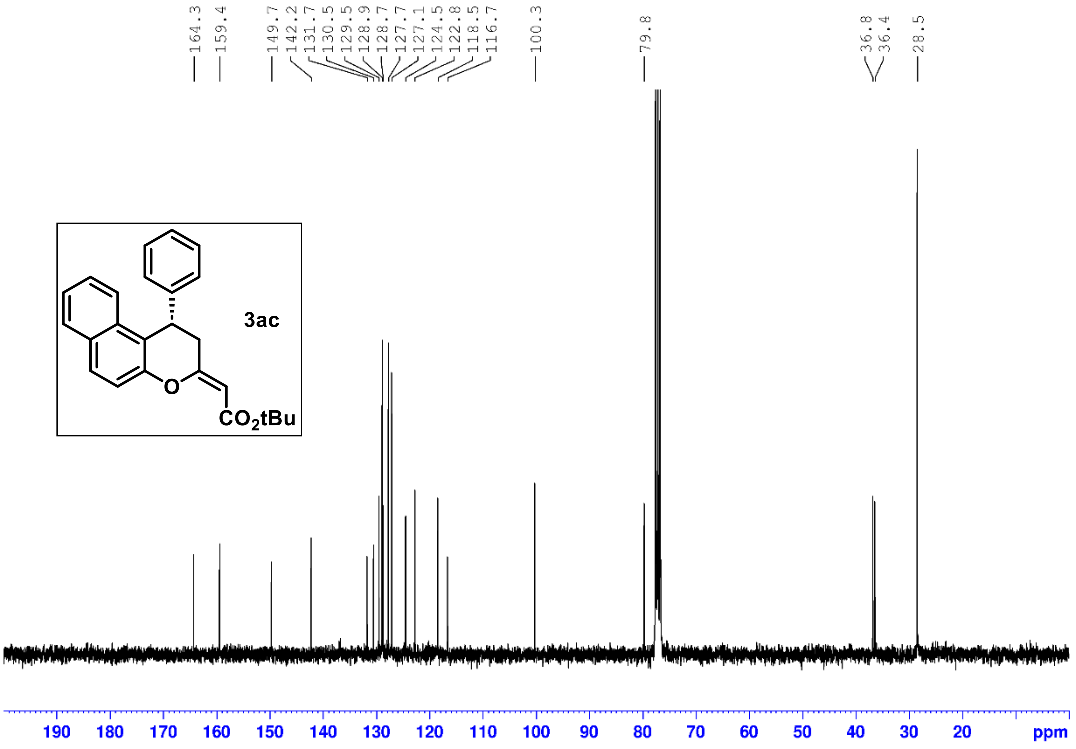

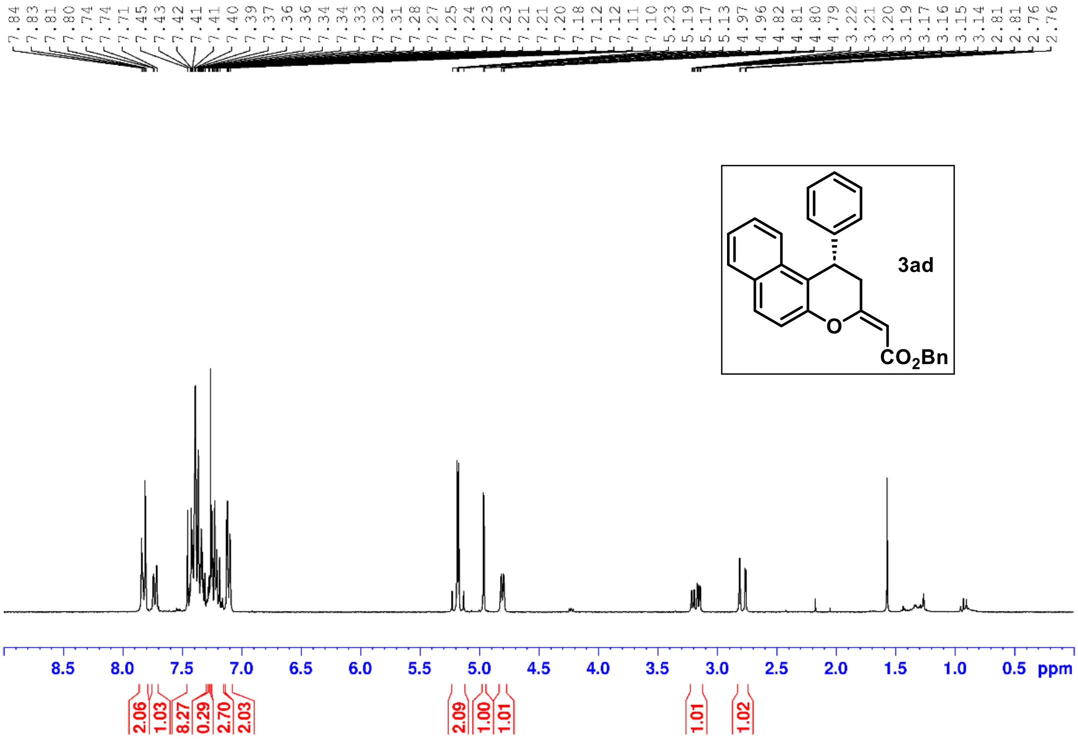
3ad
ppm

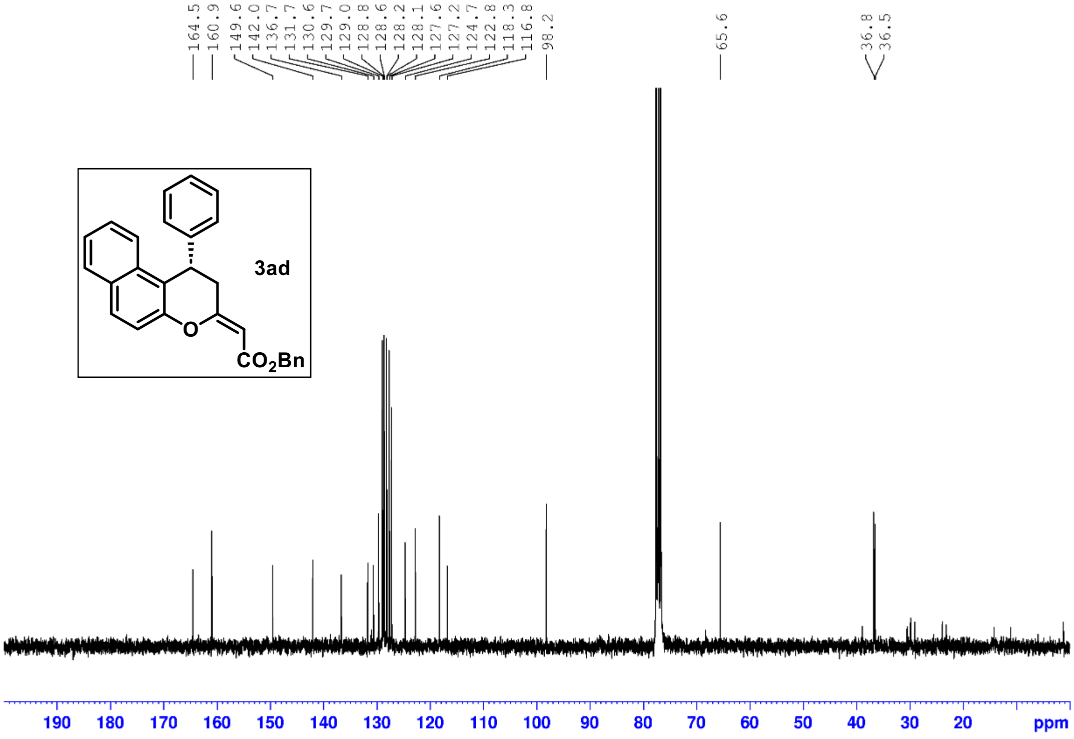
3ad
ppm

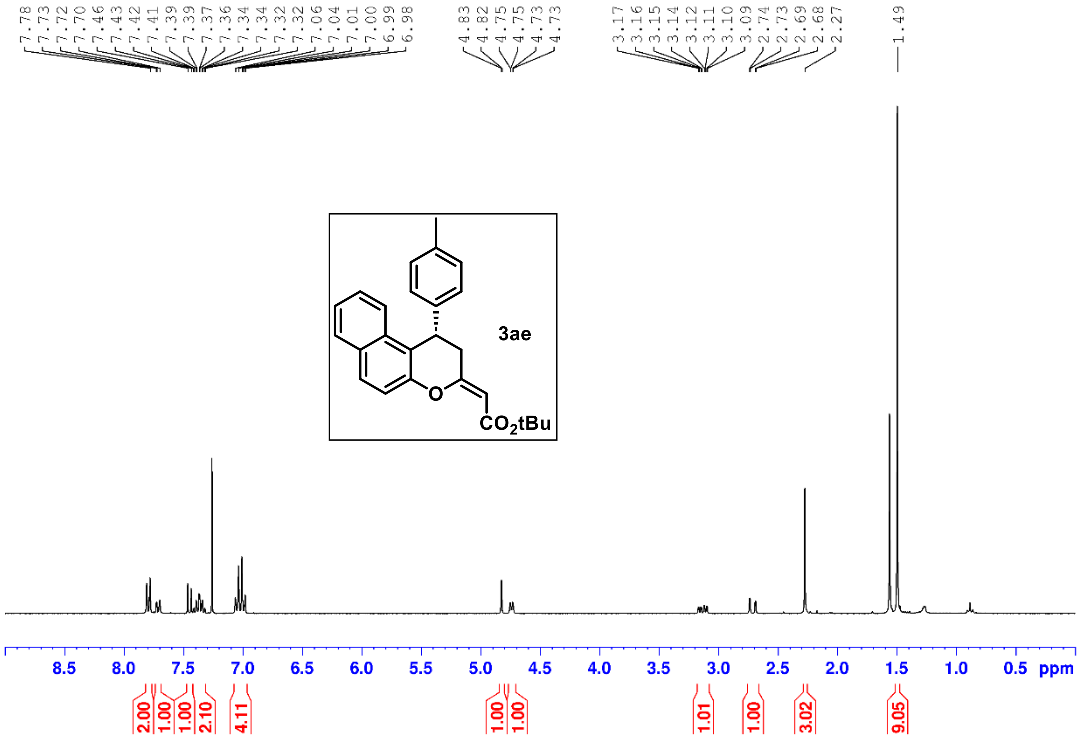
7.78 7.73 7.72 7.70 7.46 7.43 7.42 7.41 7.39 7.39 7.37 7.36 7.34 7.34 7.32 7.32 7.06 7.04 7.01 7.00 6.99 6.98
4.83 4.82 4.75 4.75 4.73 4.73
3.17 3.16 3.15 3.14 3.12 3.11 3.10 3.09 2.74 2.73 2.69 2.68 2.27
1.49
3ae
O
CO2tBu
8.5 8.0 7.5 7.0 6.5 6.0 5.5 5.0 4.5 4.0 3.5 3.0 2.5 2.0 1.5 1.0 0.5 ppm
2.00 1.00 1.00 2.10 4.11 1.00 1.00 1.01 1.00 3.02 9.05

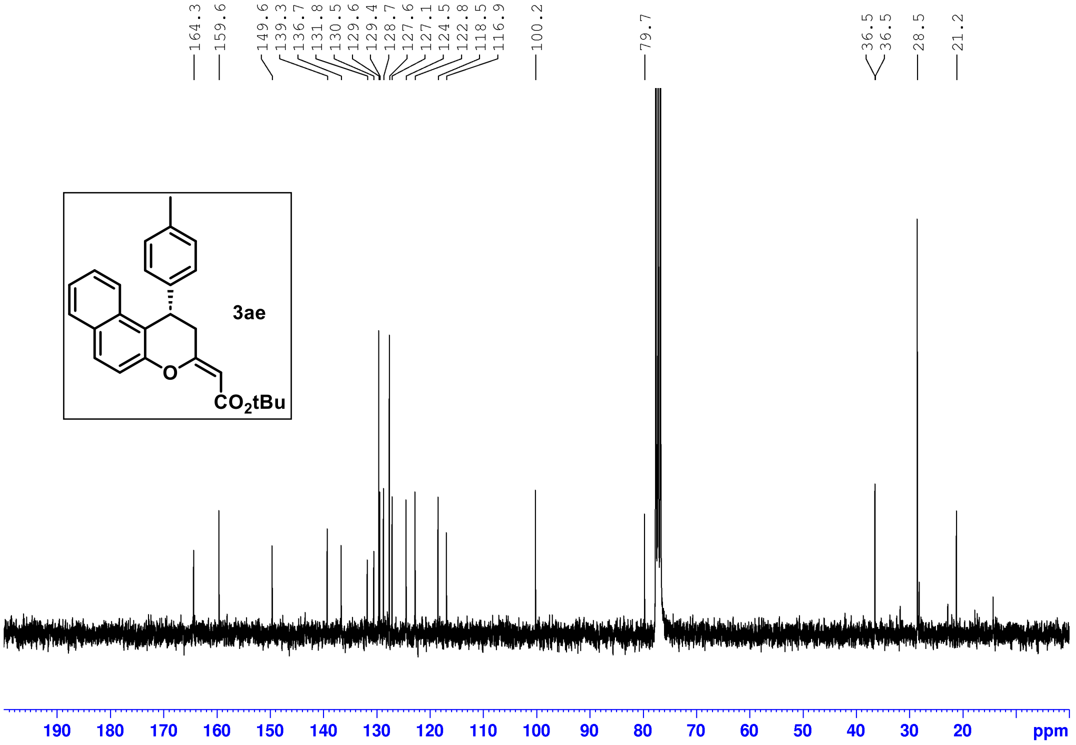
164.3 159.6 149.6 139.3 136.7 131.8 130.5 129.6 129.4 128.7 127.6 127.1 124.5 122.8 118.5 116.9 100.2 79.7 36.5 36.5 28.5 21.2
3ae
O
CO2tBu
190 180 170 160 150 140 130 120 110 100 90 80 70 60 50 40 30 20 ppm

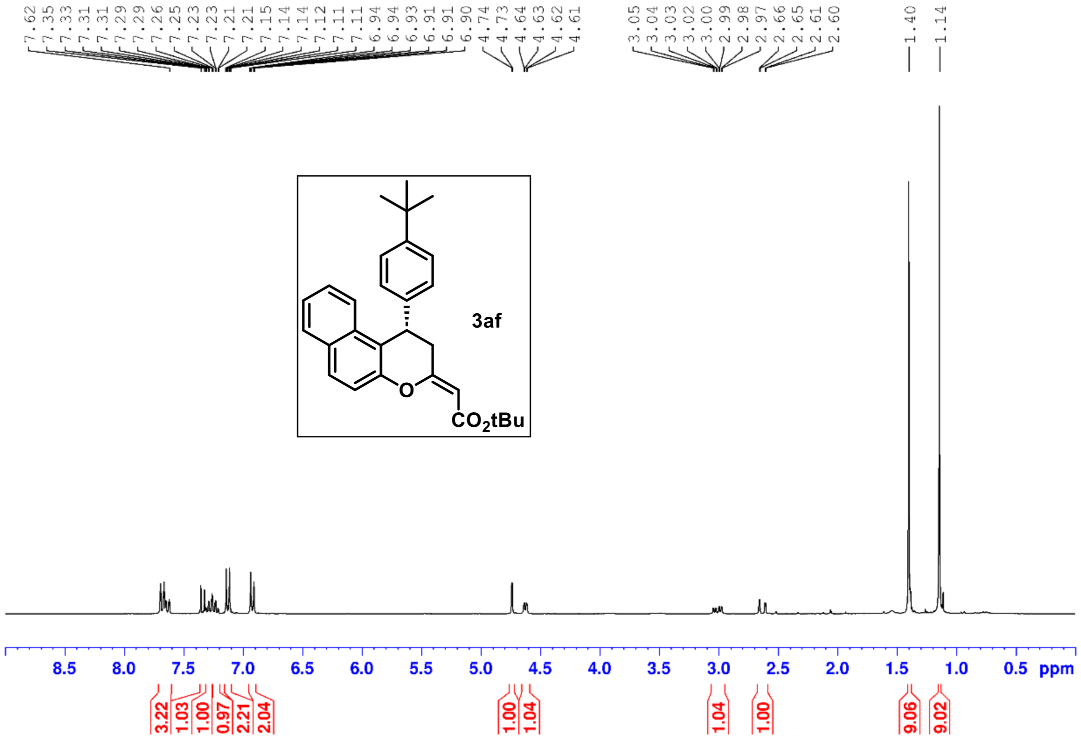
3af
CO_2tBu
7.62
7.35
7.33
7.31
7.31
7.29
7.29
7.26
7.25
7.23
7.23
7.21
7.21
7.21
7.15
7.14
7.14
7.12
7.11
7.11
6.94
6.94
6.93
6.91
6.91
6.90
4.74
4.73
4.64
4.63
4.62
4.61
3.05
3.04
3.03
3.02
3.00
2.99
2.98
2.97
2.66
2.65
2.61
2.60
1.40
1.14
3.22
1.03
1.00
0.97
2.21
2.04
1.00
1.04
1.04
1.00
9.06
9.02
ppm

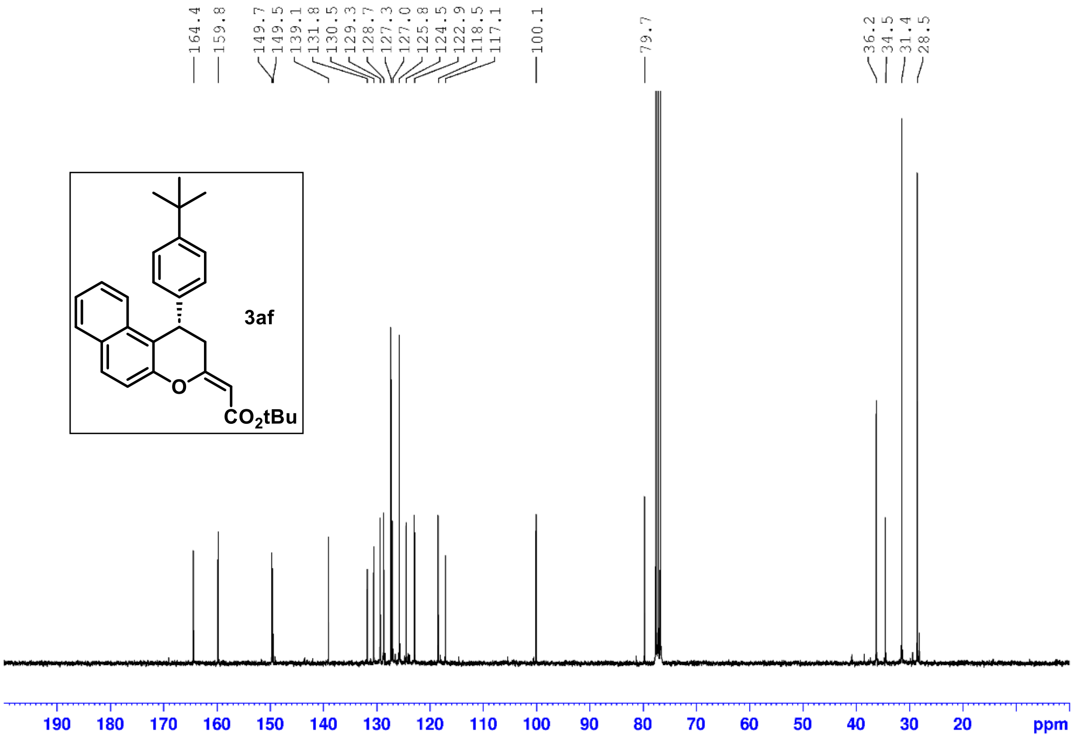
3af
CO_2tBu
164.4
159.8
149.7
149.5
139.1
131.8
130.5
129.3
128.7
127.3
127.0
125.8
124.5
122.9
118.5
117.1
100.1
79.7
36.2
34.5
31.4
28.5
ppm

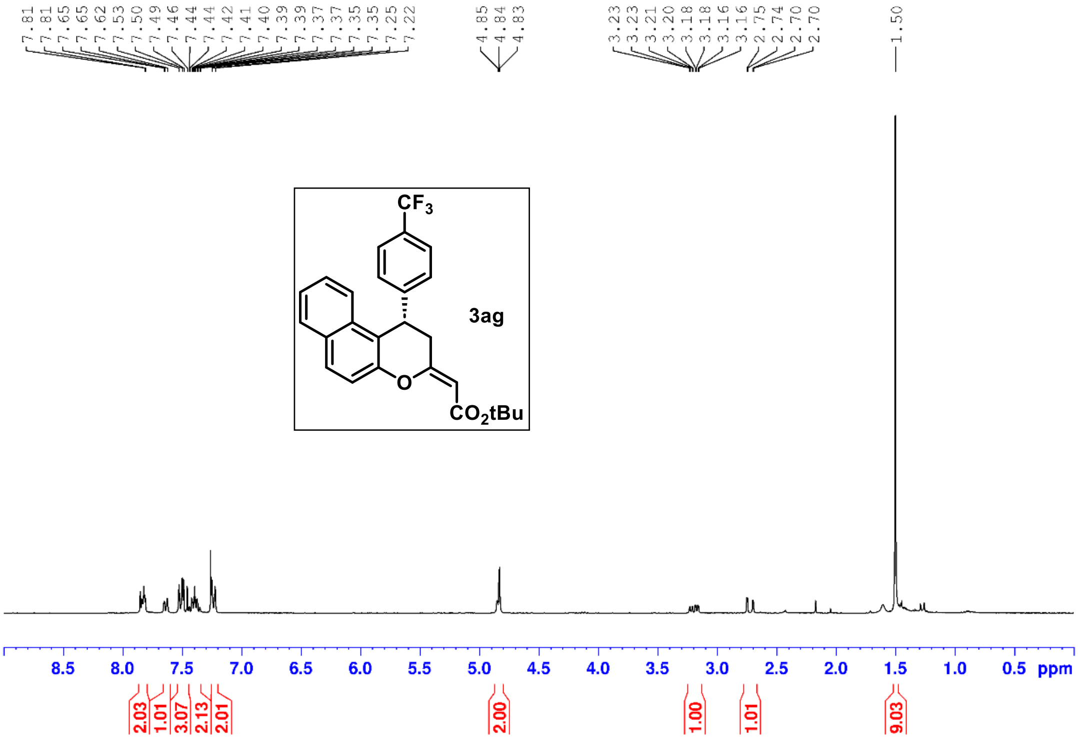
7.81
7.81
7.65
7.65
7.62
7.53
7.50
7.49
7.46
7.44
7.44
7.42
7.41
7.40
7.39
7.39
7.37
7.37
7.35
7.35
7.25
7.22
4.85
4.84
4.83
3.23
3.23
3.21
3.20
3.18
3.18
3.16
3.16
2.75
2.74
2.70
2.70
1.50
CF3
3ag
O
CO2tBu
8.5
8.0
7.5
7.0
6.5
6.0
5.5
5.0
4.5
4.0
3.5
3.0
2.5
2.0
1.5
1.0
0.5
ppm
2.03
1.01
3.07
2.13
2.01
2.00
1.00
1.01
9.03

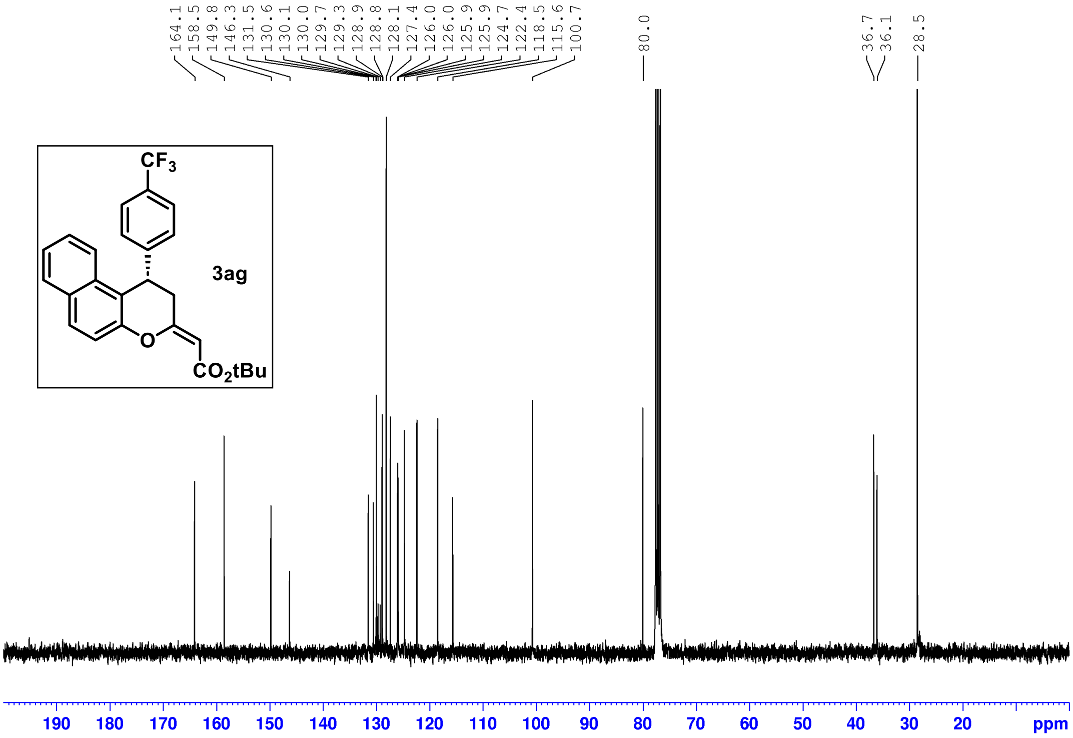
164.1
158.5
149.8
146.3
131.5
130.6
130.1
130.0
129.7
129.3
128.9
128.8
128.1
127.4
126.0
126.0
125.9
125.9
124.7
122.4
118.5
115.6
100.7
80.0
36.7
36.1
28.5
CF3
3ag
O
CO2tBu
190
180
170
160
150
140
130
120
110
100
90
80
70
60
50
40
30
20
ppm

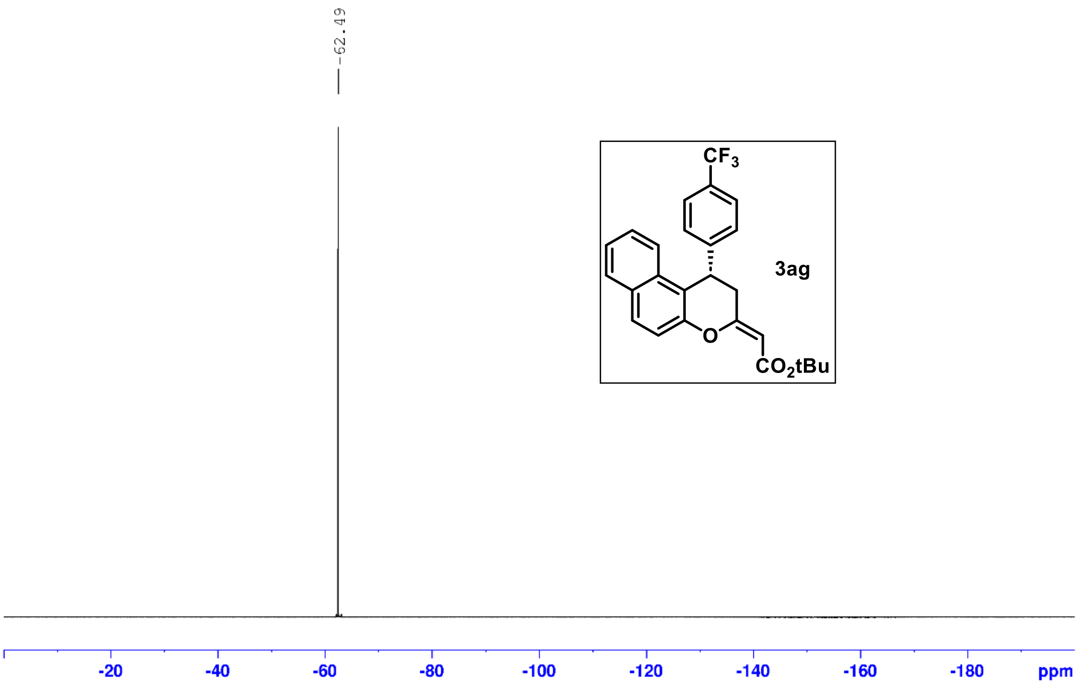
-62.49
CF3
O
CO2tBu
3ag
-20
-40
-60
-80
-100
-120
-140
-160
-180
ppm

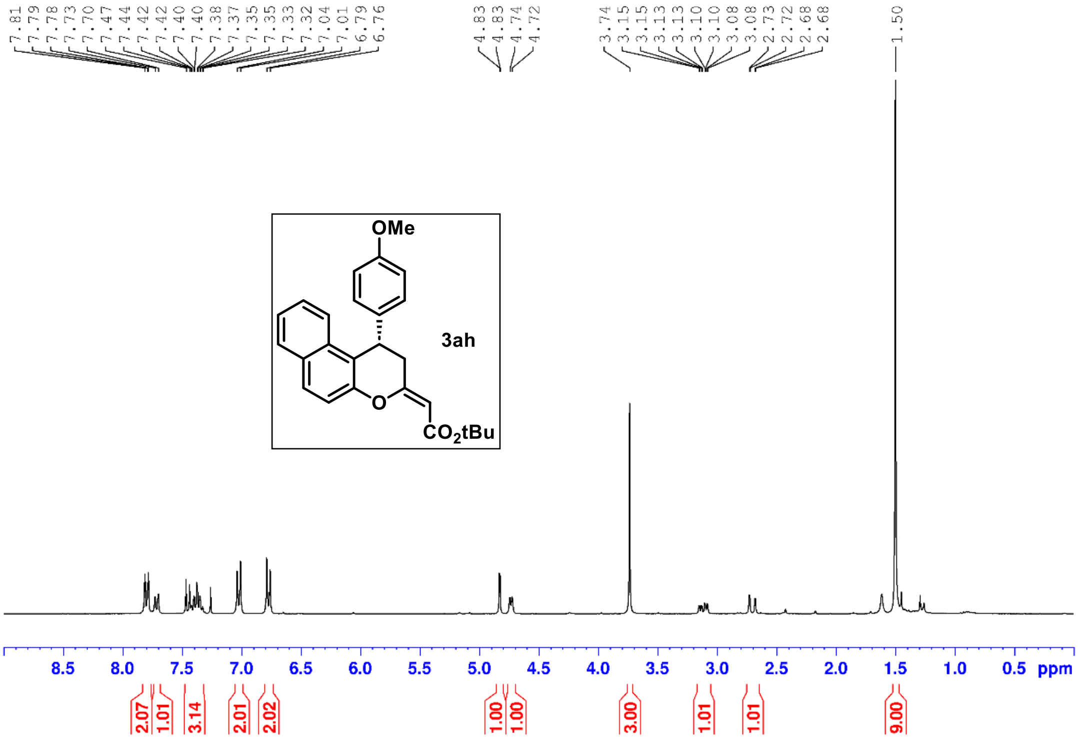
7.81 7.79 7.78 7.73 7.70 7.47 7.44 7.42 7.42 7.40 7.40 7.38 7.37 7.35 7.35 7.33 7.32 7.04 7.01 6.79 6.76
4.83 4.83 4.74 4.72
3.74 3.15 3.15 3.13 3.13 3.10 3.10 3.08 3.08 2.73 2.72 2.68 2.68
1.50
OMe
3ah
O
CO2tBu
8.5 8.0 7.5 7.0 6.5 6.0 5.5 5.0 4.5 4.0 3.5 3.0 2.5 2.0 1.5 1.0 0.5 ppm
2.07 1.01 3.14 2.01 2.02 1.00 1.00 3.00 1.01 1.01 9.00

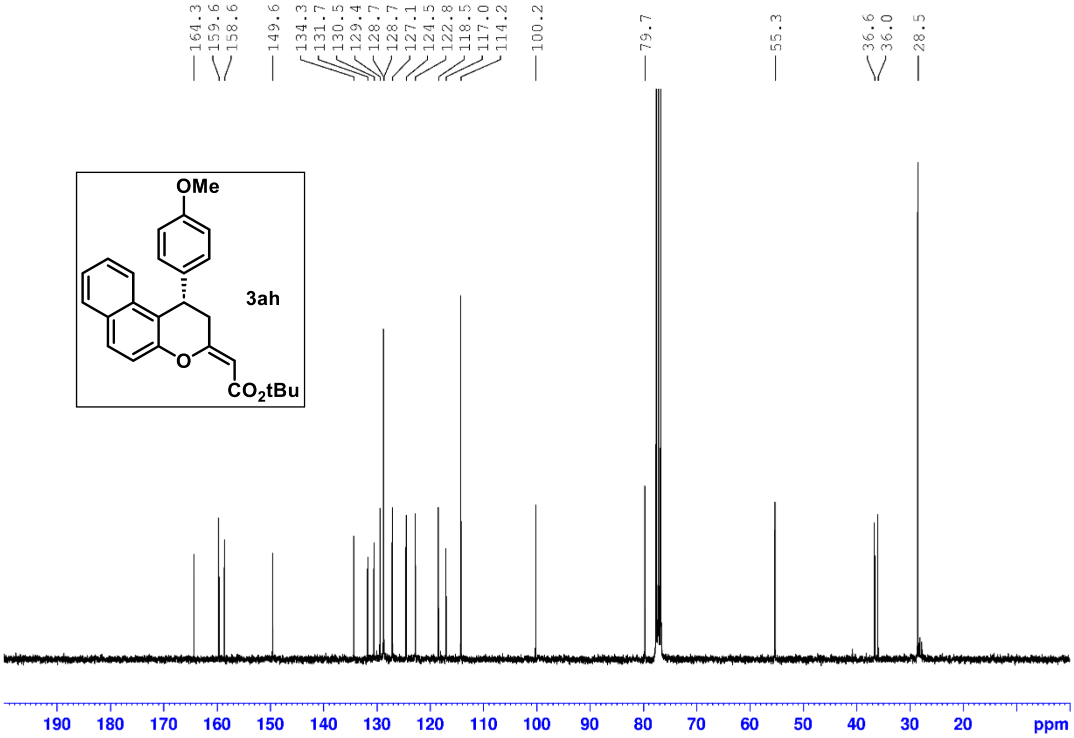
164.3 159.6 158.6 149.6 134.3 131.7 130.5 129.4 128.7 128.7 127.1 124.5 122.8 118.5 117.0 114.2 100.2 79.7 55.3 36.6 36.0 28.5
OMe
3ah
O
CO2tBu
190 180 170 160 150 140 130 120 110 100 90 80 70 60 50 40 30 20 ppm

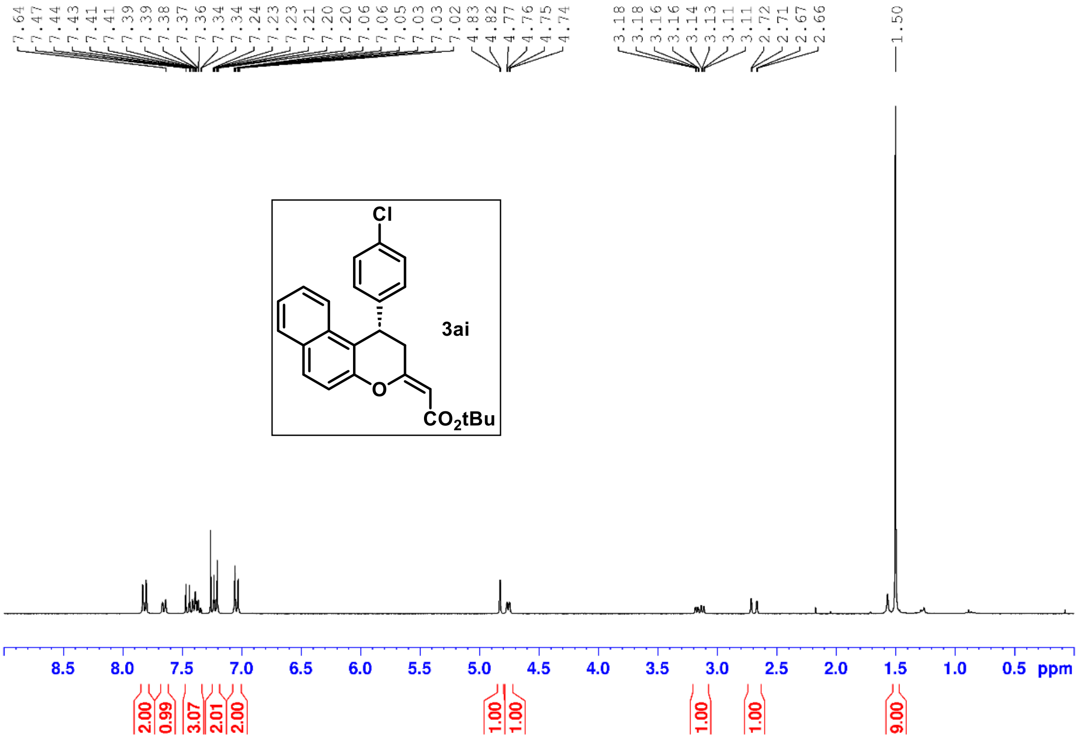

7.64
7.47
7.44
7.43
7.41
7.41
7.39
7.39
7.38
7.37
7.36
7.34
7.34
7.24
7.23
7.23
7.21
7.20
7.20
7.06
7.06
7.05
7.03
7.03
7.02
4.83
4.82
4.77
4.76
4.75
4.74
3.18
3.18
3.16
3.14
3.13
3.11
2.72
2.71
2.67
2.66
1.50
Cl
3ai
O
CO2tBu
8.5
8.0
7.5
7.0
6.5
6.0
5.5
5.0
4.5
4.0
3.5
3.0
2.5
2.0
1.5
1.0
0.5
ppm
2.00
0.99
3.07
2.01
2.00
1.00
1.00
1.00
1.00
9.00

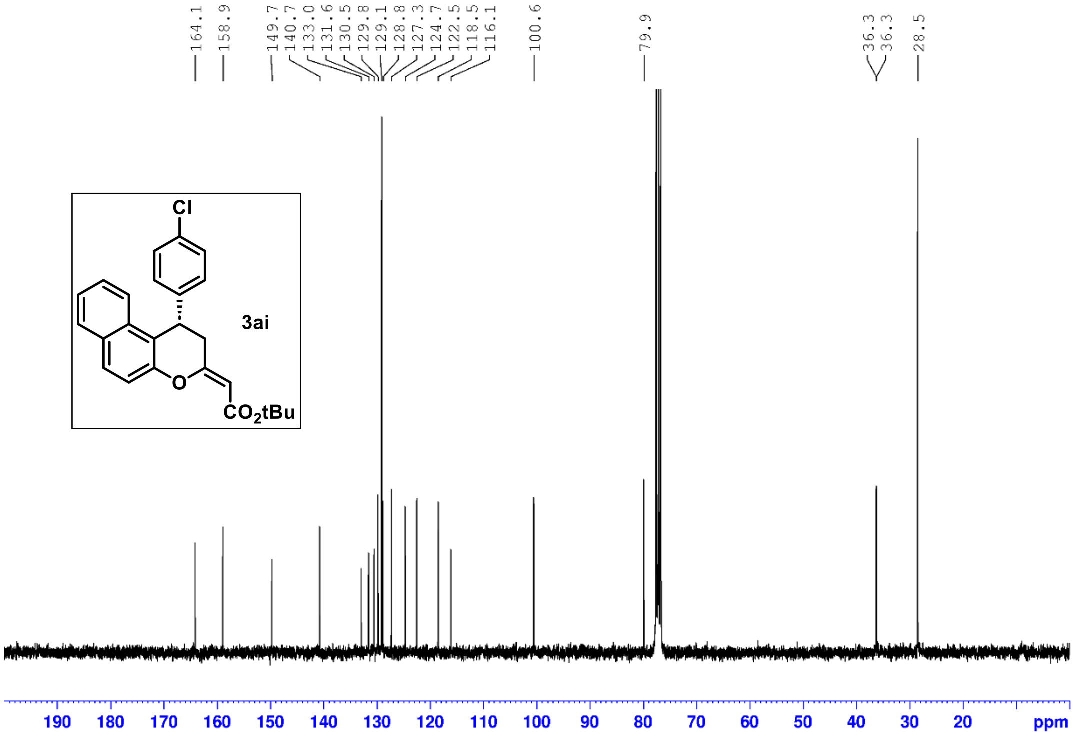

164.1
158.9
149.7
140.7
133.0
131.6
130.5
129.8
129.1
128.8
127.3
124.7
122.5
118.5
116.1
100.6
79.9
36.3
36.3
28.5
Cl
3ai
O
CO2tBu
190
180
170
160
150
140
130
120
110
100
90
80
70
60
50
40
30
20
ppm

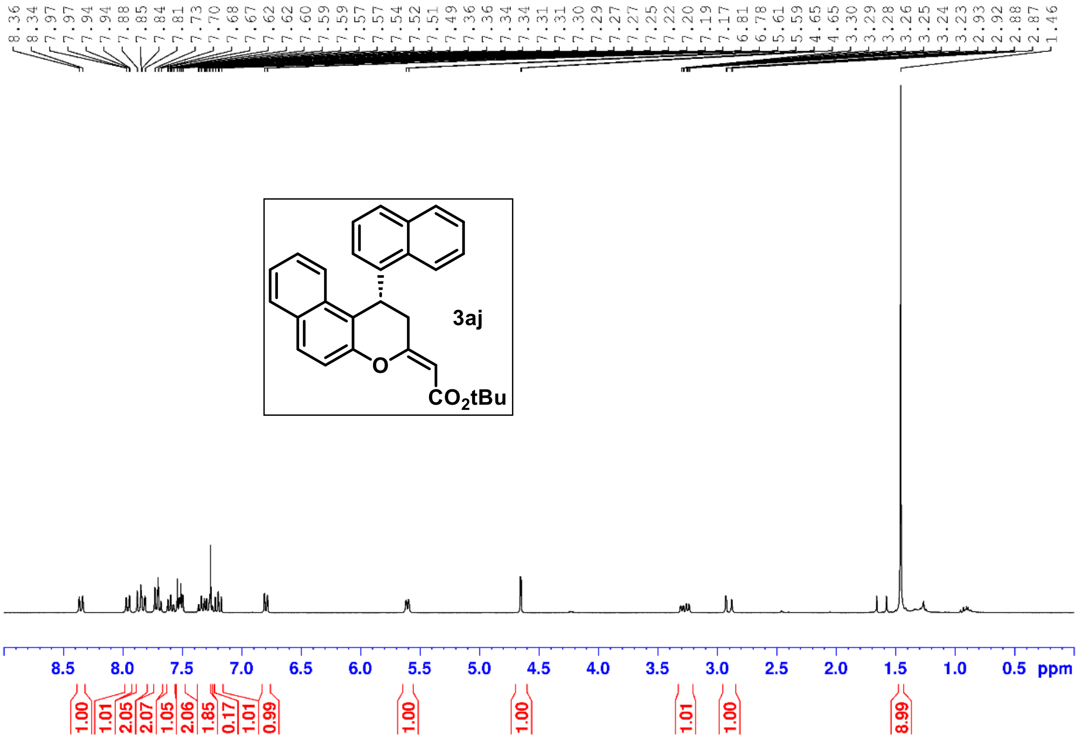

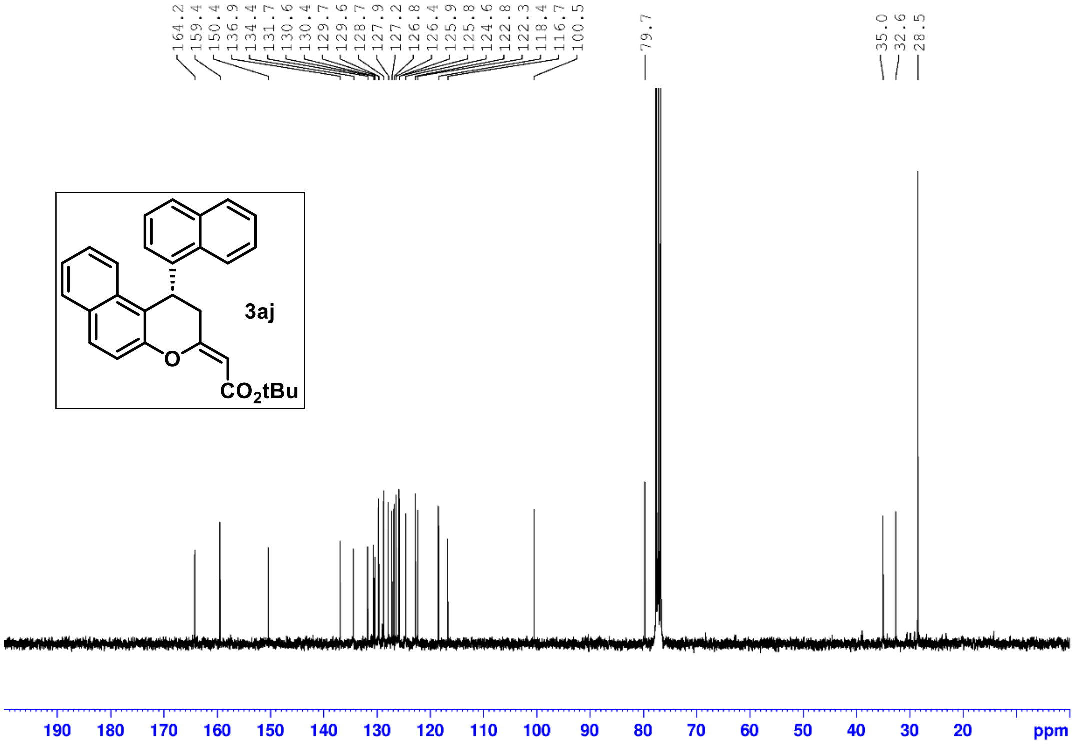

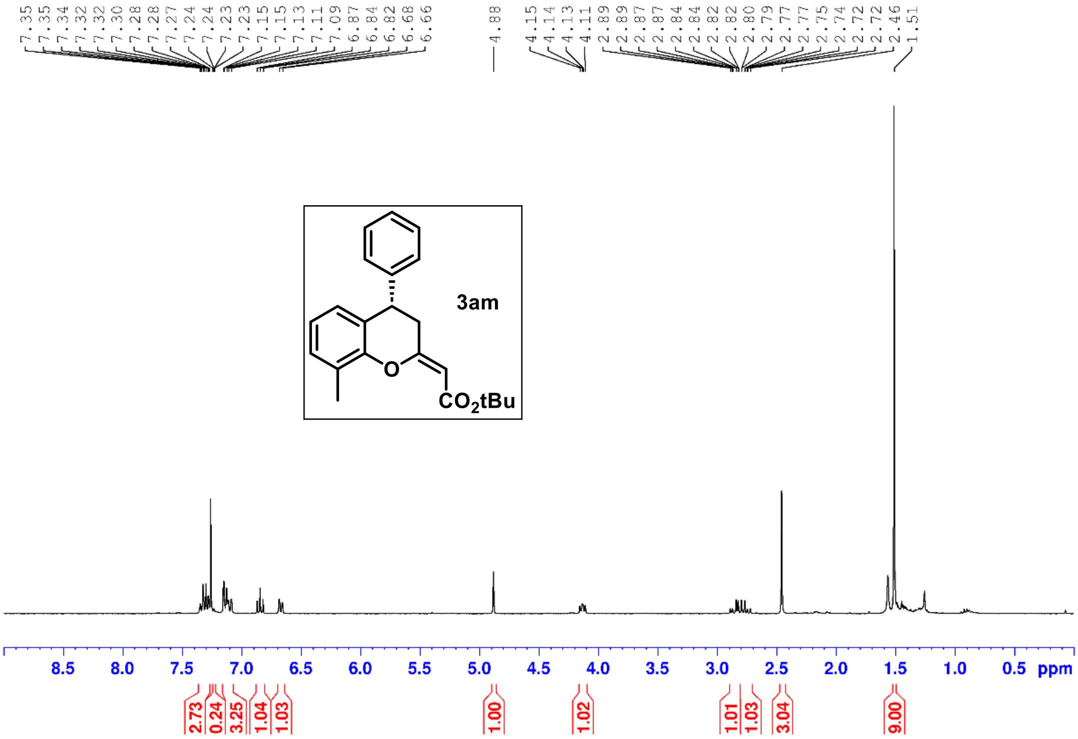
7.35
7.35
7.34
7.32
7.32
7.30
7.28
7.28
7.27
7.24
7.24
7.23
7.23
7.15
7.15
7.13
7.11
7.09
6.87
6.84
6.82
6.68
6.66
4.88
4.15
4.14
4.13
4.11
2.89
2.89
2.87
2.87
2.84
2.84
2.82
2.82
2.80
2.79
2.77
2.77
2.75
2.74
2.72
2.72
2.46
1.51
3am
O
CO2tBu
8.5
8.0
7.5
7.0
6.5
6.0
5.5
5.0
4.5
4.0
3.5
3.0
2.5
2.0
1.5
1.0
0.5
ppm
2.73
0.24
3.25
1.04
1.03
1.00
1.02
1.01
1.03
3.04
9.00

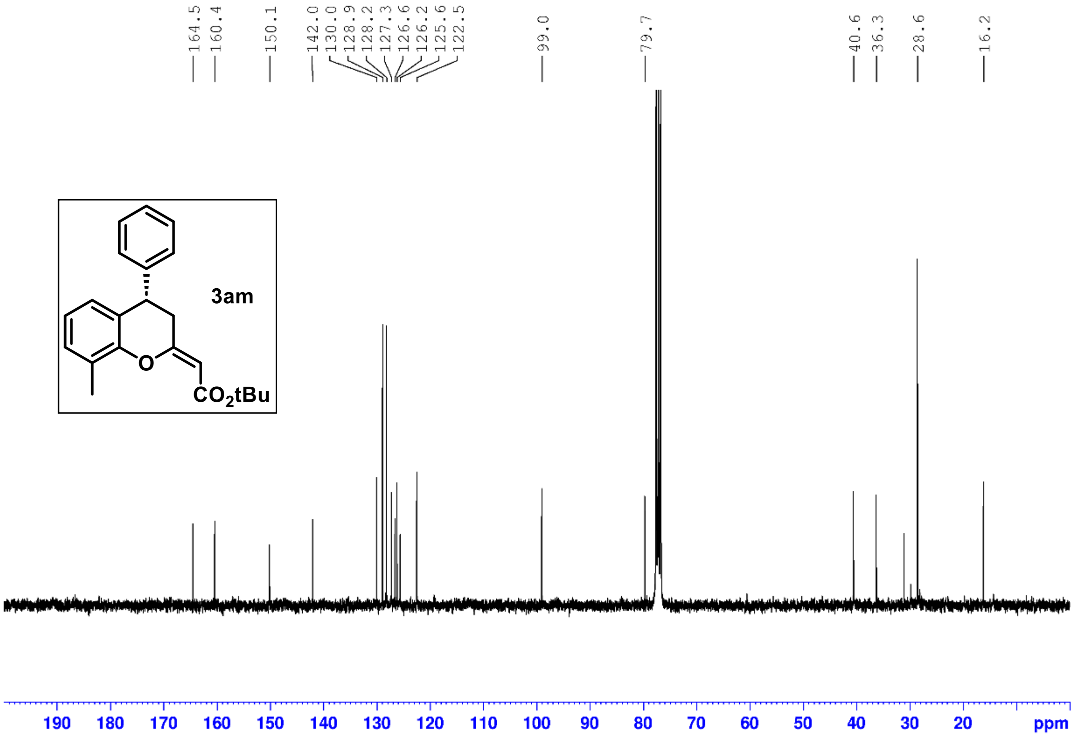
164.5
160.4
150.1
142.0
130.0
128.9
128.2
127.3
126.6
126.2
125.6
122.5
99.0
79.7
40.6
36.3
28.6
16.2
3am
O
CO2tBu
190
180
170
160
150
140
130
120
110
100
90
80
70
60
50
40
30
20
ppm

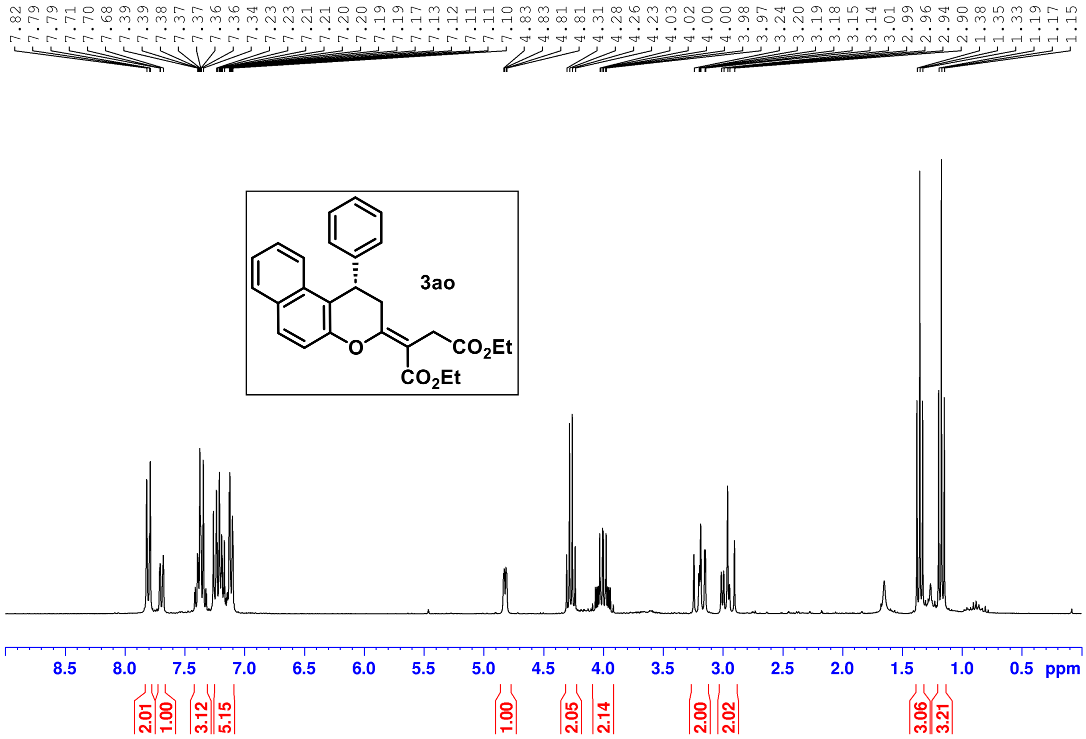
7.82 7.79 7.79 7.71 7.70 7.68 7.39 7.39 7.38 7.37 7.37 7.36 7.36 7.34 7.23 7.23 7.23 7.21 7.21 7.20 7.20 7.19 7.19 7.19 7.17 7.13 7.12 7.11 7.11 7.10 4.83 4.83 4.81 4.81 4.31 4.28 4.26 4.23 4.03 4.02 4.00 4.00 3.98 3.97 3.24 3.20 3.19 3.18 3.15 3.14 3.01 2.99 2.96 2.94 2.90 1.38 1.35 1.33 1.19 1.17 1.15
3ao
CO2Et
O
CO2Et
8.5 8.0 7.5 7.0 6.5 6.0 5.5 5.0 4.5 4.0 3.5 3.0 2.5 2.0 1.5 1.0 0.5 ppm
2.01 1.00 3.12 5.15 1.00 2.05 2.14 2.00 2.02 3.06 3.21

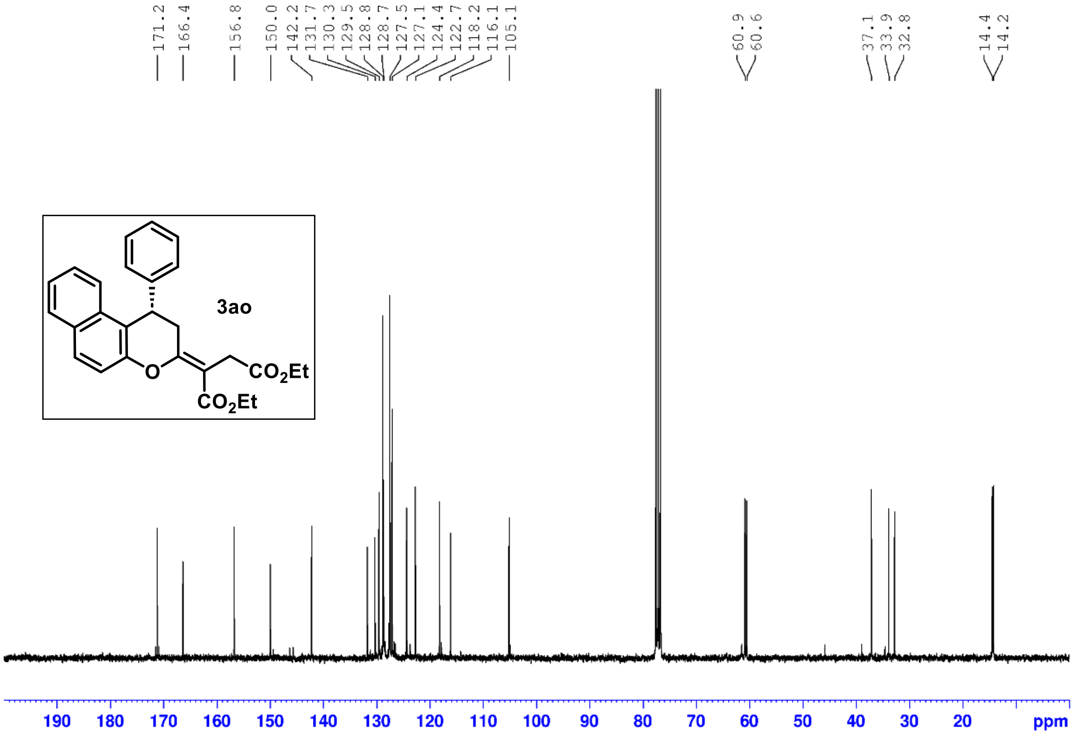
171.2 166.4 156.8 150.0 142.2 131.7 130.3 129.5 128.8 128.7 127.5 127.1 124.4 122.7 118.2 116.1 105.1 60.9 60.6 37.1 33.9 32.8 14.4 14.2
3ao
CO2Et
O
CO2Et
190 180 170 160 150 140 130 120 110 100 90 80 70 60 50 40 30 20 ppm

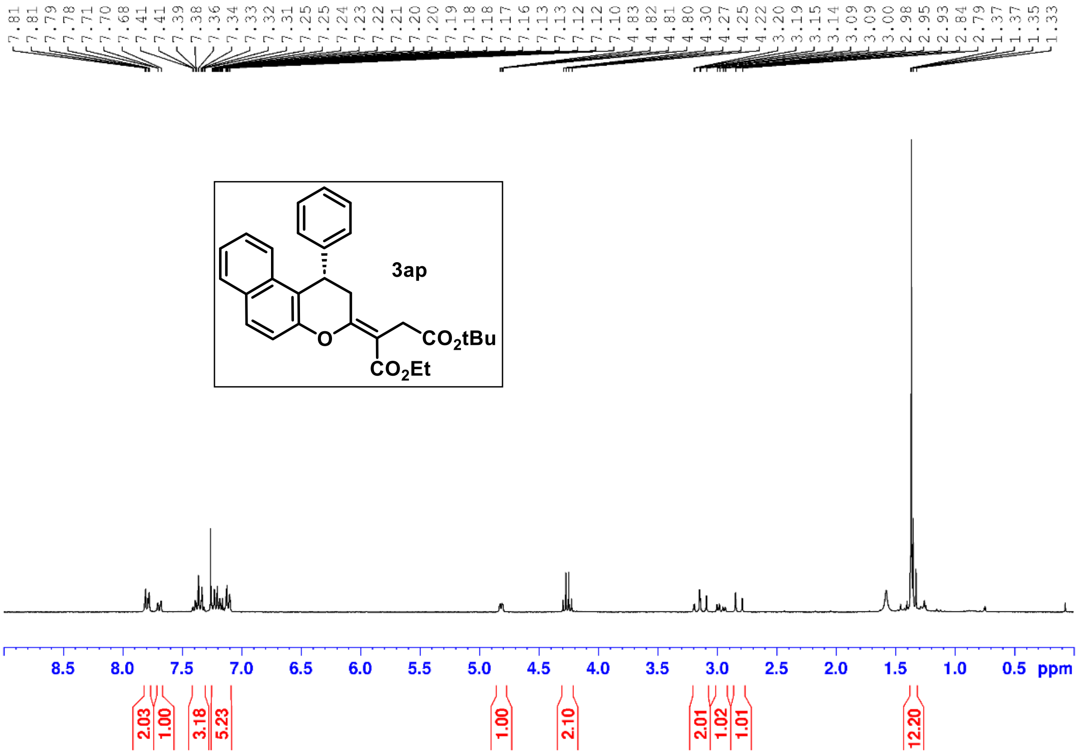
7.81 7.81 7.79 7.78 7.71 7.70 7.68 7.41 7.41 7.39 7.38 7.36 7.34 7.33 7.32 7.31 7.25 7.25 7.24 7.23 7.22 7.21 7.20 7.20 7.19 7.18 7.18 7.17 7.16 7.13 7.13 7.12 7.12 7.10 4.83 4.82 4.81 4.80 4.30 4.27 4.25 4.22 3.20 3.19 3.15 3.14 3.09 3.09 3.00 2.98 2.95 2.93 2.84 2.79 1.37 1.37 1.35 1.33
3ap
O
CO2tBu
CO2Et
8.5 8.0 7.5 7.0 6.5 6.0 5.5 5.0 4.5 4.0 3.5 3.0 2.5 2.0 1.5 1.0 0.5 ppm
2.03 1.00 3.18 5.23 1.00 2.10 2.01 1.02 1.01 12.20

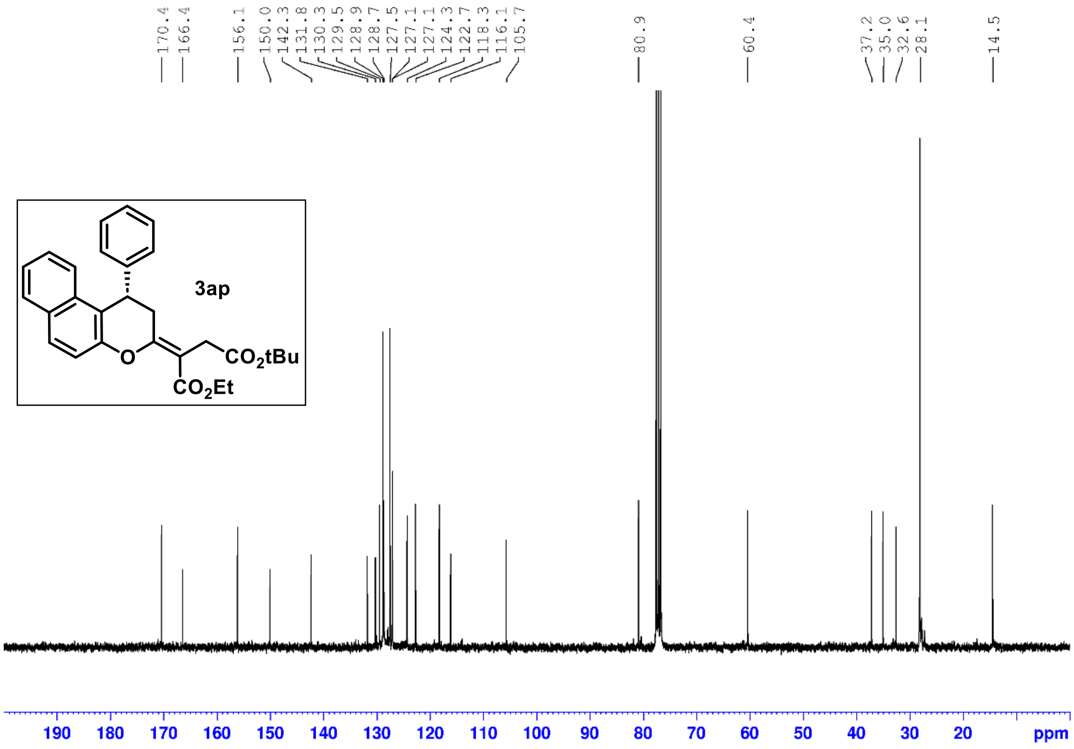
170.4 166.4 156.1 150.0 142.3 131.8 130.3 129.5 128.9 128.7 127.5 127.1 127.1 124.3 122.7 118.3 116.1 105.7 80.9 60.4 37.2 35.0 32.6 28.1 14.5
3ap
O
CO2tBu
CO2Et
190 180 170 160 150 140 130 120 110 100 90 80 70 60 50 40 30 20 ppm

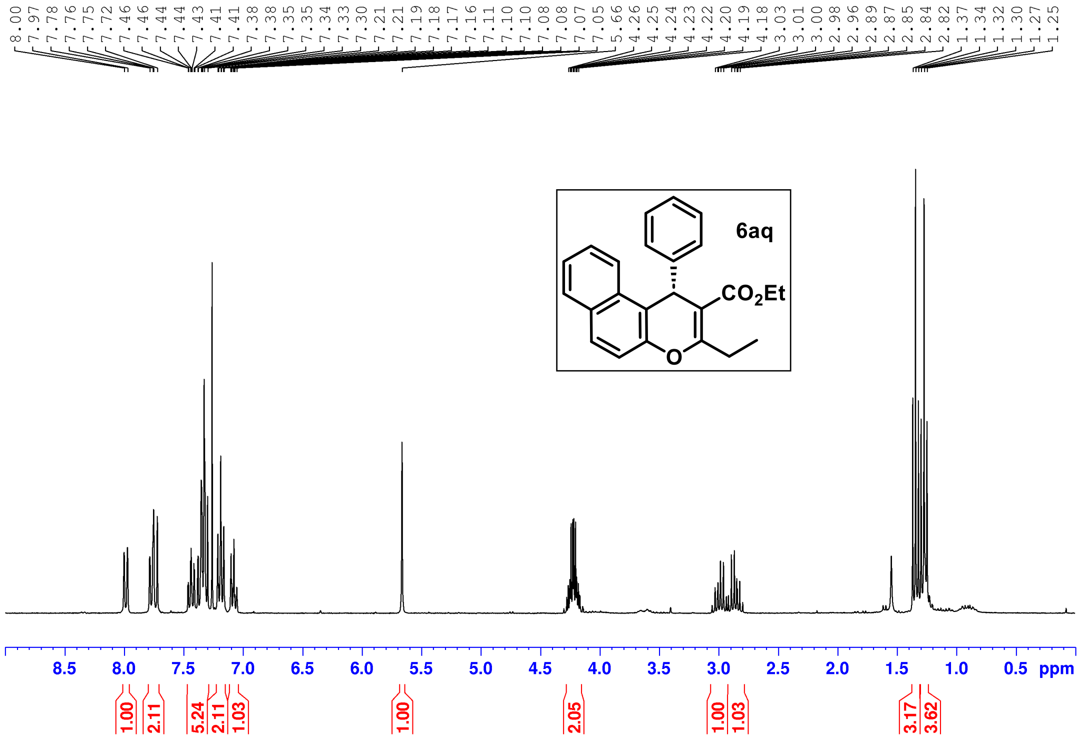
8.00 7.97 7.78 7.76 7.75 7.72 7.46 7.46 7.44 7.44 7.43 7.41 7.41 7.38 7.38 7.35 7.34 7.33 7.30 7.21 7.21 7.19 7.18 7.17 7.16 7.11 7.10 7.10 7.08 7.08 7.07 7.05 5.66 4.26 4.25 4.24 4.23 4.22 4.20 4.19 4.18 3.03 3.01 3.00 2.98 2.96 2.89 2.87 2.85 2.84 2.82 1.37 1.34 1.32 1.30 1.27 1.25
6aq
CO_2Et
O
8.5 8.0 7.5 7.0 6.5 6.0 5.5 5.0 4.5 4.0 3.5 3.0 2.5 2.0 1.5 1.0 0.5 ppm
1.00 2.11 5.24 2.11 1.03 1.00 2.05 1.00 1.03 3.17 3.62

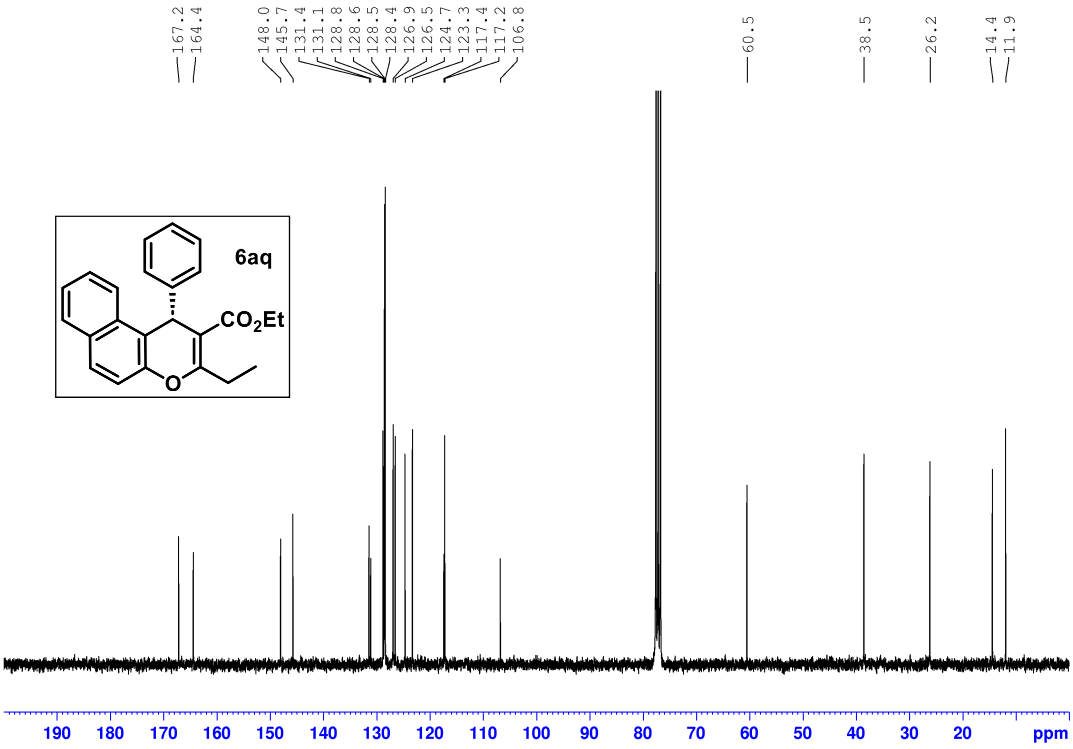
167.2 164.4 148.0 145.7 131.4 131.1 128.8 128.6 128.5 128.4 126.9 126.5 124.7 123.3 117.4 117.2 106.8 60.5 38.5 26.2 14.4 11.9
6aq
CO_2Et
O
190 180 170 160 150 140 130 120 110 100 90 80 70 60 50 40 30 20 ppm

14.3 NMR spectra of products of follow-up transformations

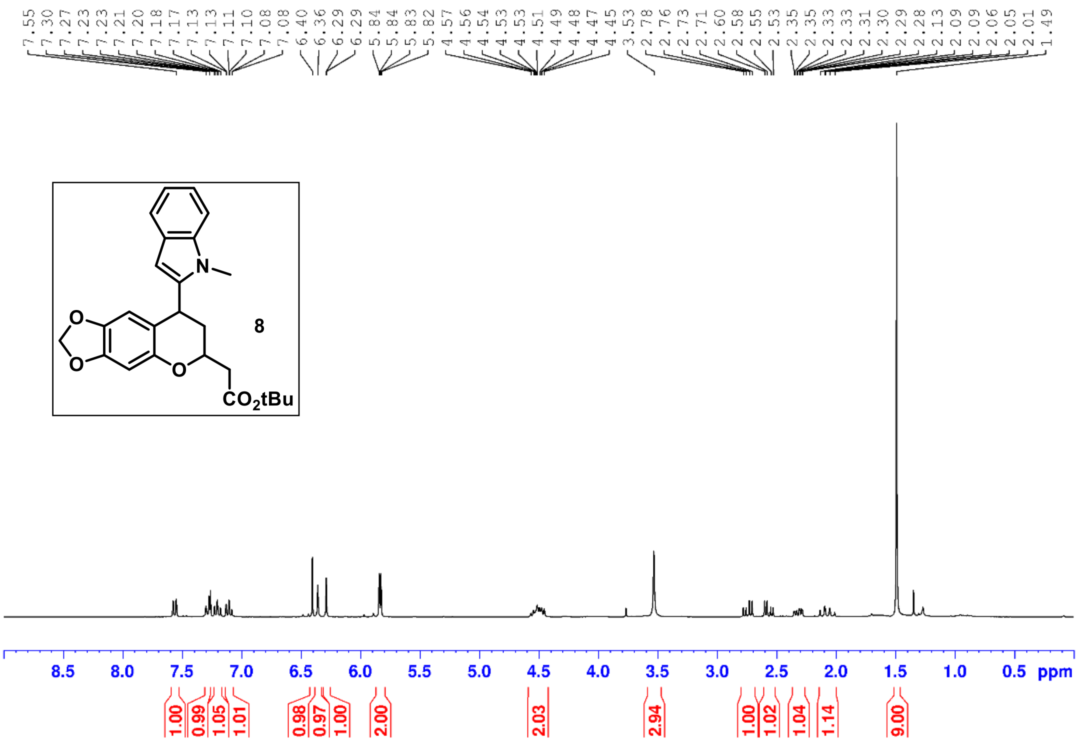

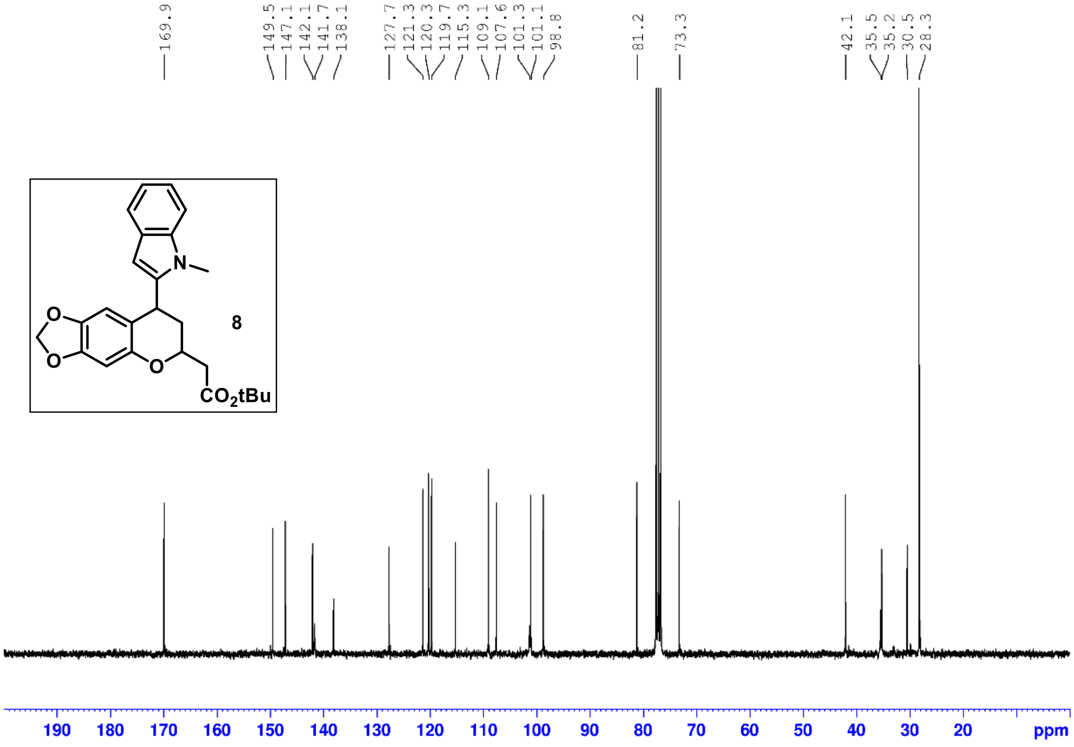

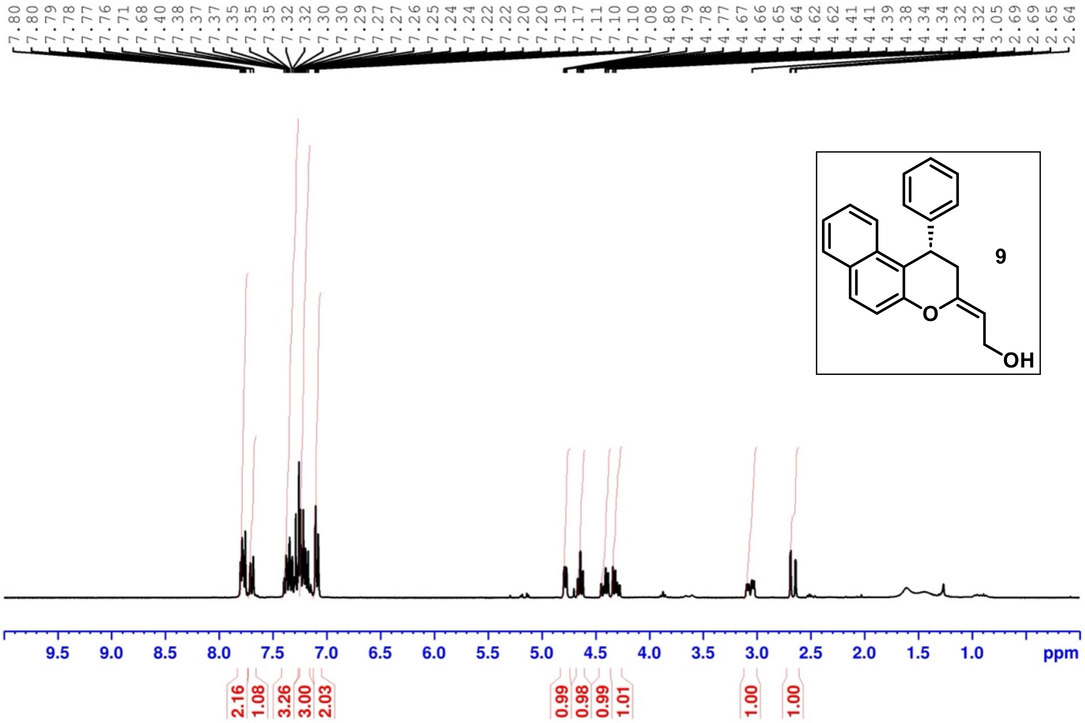
7.80 7.79 7.78 7.77 7.76 7.71 7.68 7.40 7.38 7.37 7.35 7.35 7.32 7.32 7.30 7.29 7.27 7.27 7.26 7.25 7.24 7.24 7.22 7.22 7.20 7.20 7.19 7.17 7.11 7.10 7.10 7.08 4.80 4.79 4.78 4.77 4.67 4.66 4.65 4.64 4.62 4.62 4.41 4.41 4.39 4.38 4.34 4.34 4.32 4.32 3.05 2.69 2.69 2.65 2.64
9
O
OH
9.5 9.0 8.5 8.0 7.5 7.0 6.5 6.0 5.5 5.0 4.5 4.0 3.5 3.0 2.5 2.0 1.5 1.0 ppm
2.16 1.08 3.26 3.00 2.03 0.99 0.98 0.99 1.01 1.00 1.00

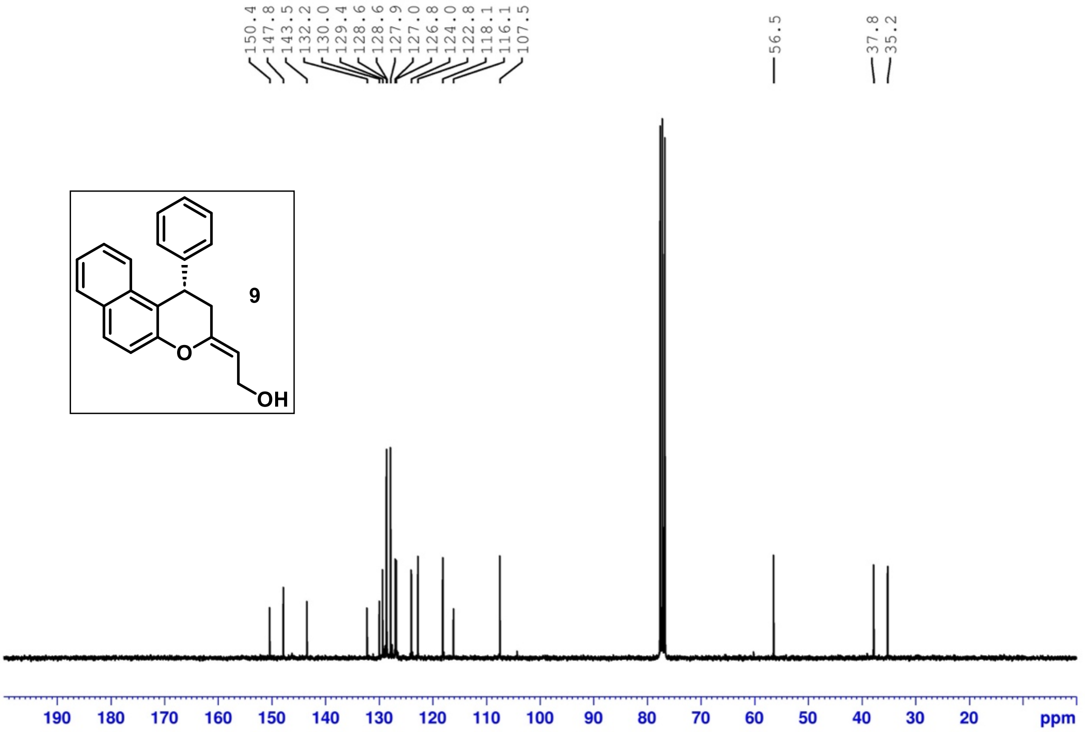
150.4 147.8 143.5 132.2 130.0 129.4 128.6 127.9 127.0 126.8 124.0 122.8 118.1 116.1 107.5
56.5
37.8 35.2
9
O
OH
190 180 170 160 150 140 130 120 110 100 90 80 70 60 50 40 30 20 ppm

14.4 NMR spectra of products from reactions of oQMs with reference nucleophiles

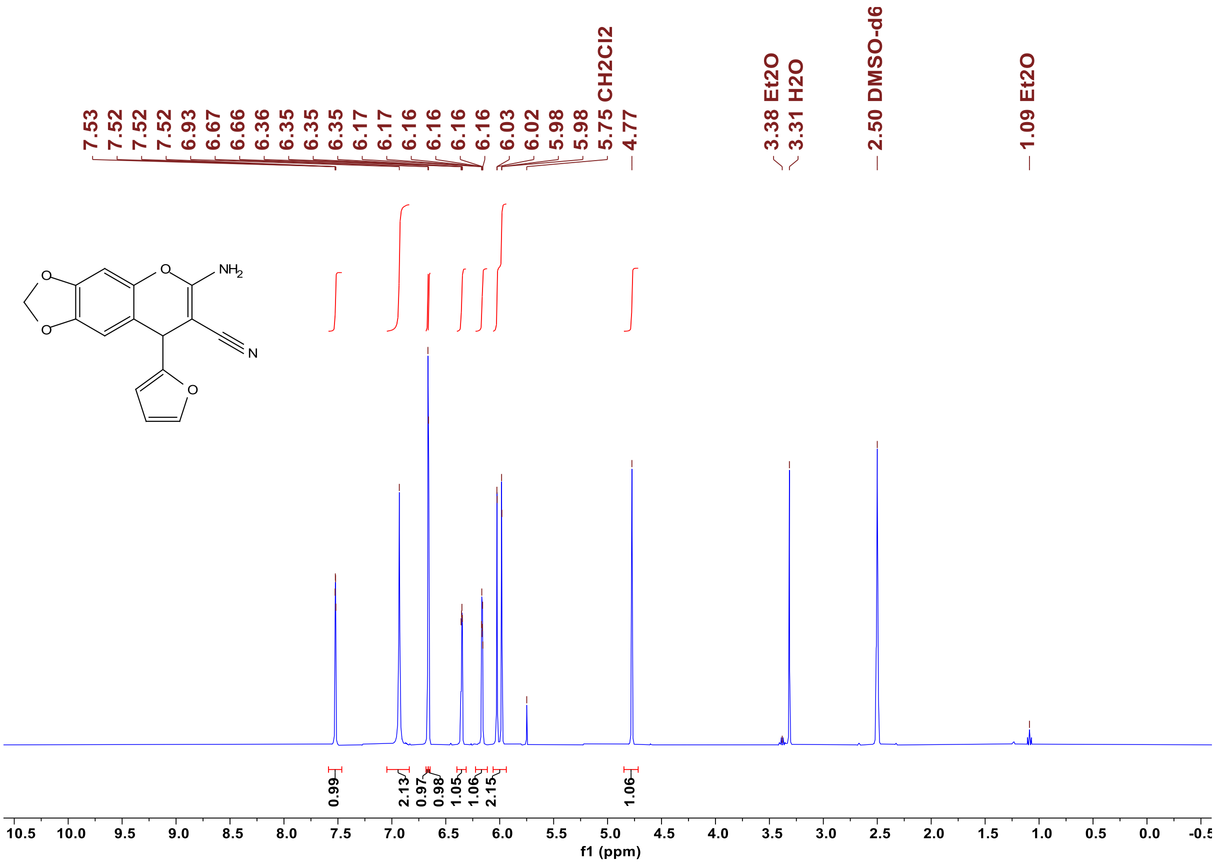

Fig. S10 ^{1}H NMR spectrum of **P1** in d_6-DMSO (400 MHz) CG585

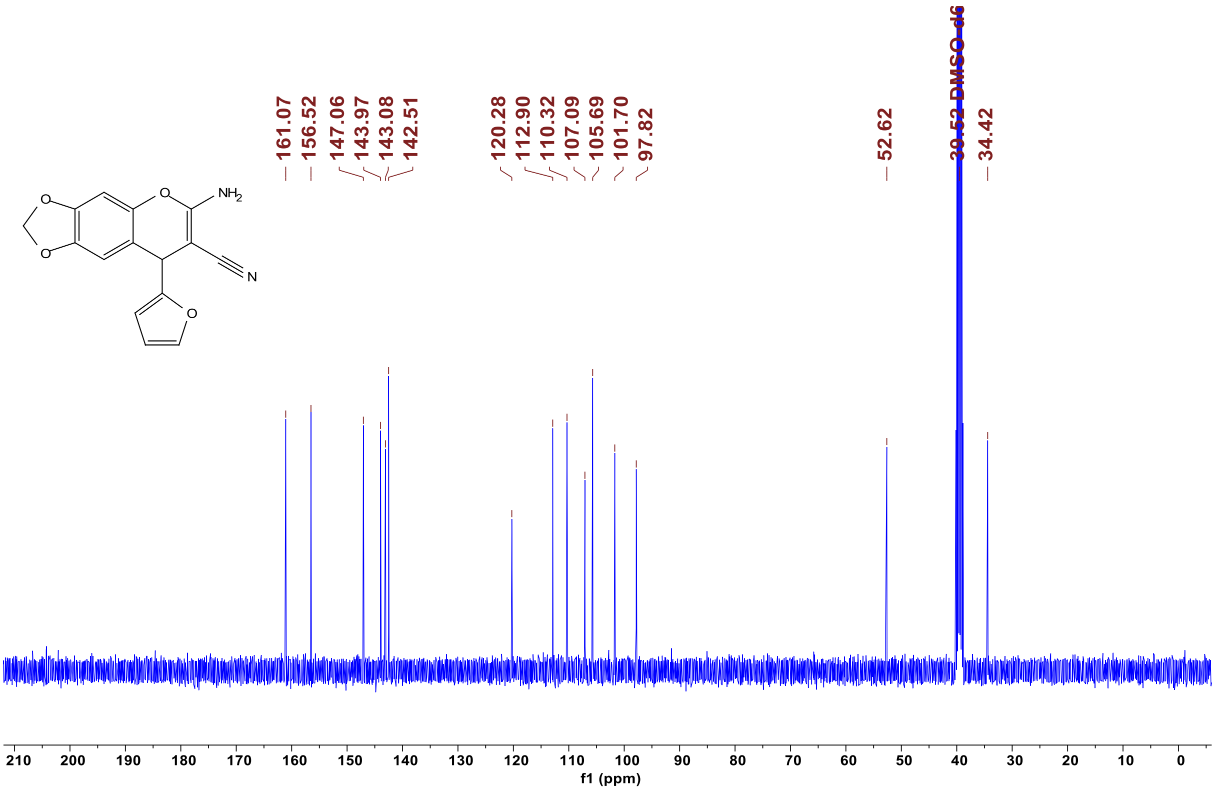

Fig. S38 ^{13}C{^{1}H} NMR spectrum of **P1** in d_6-DMSO (101 MHz) CG585

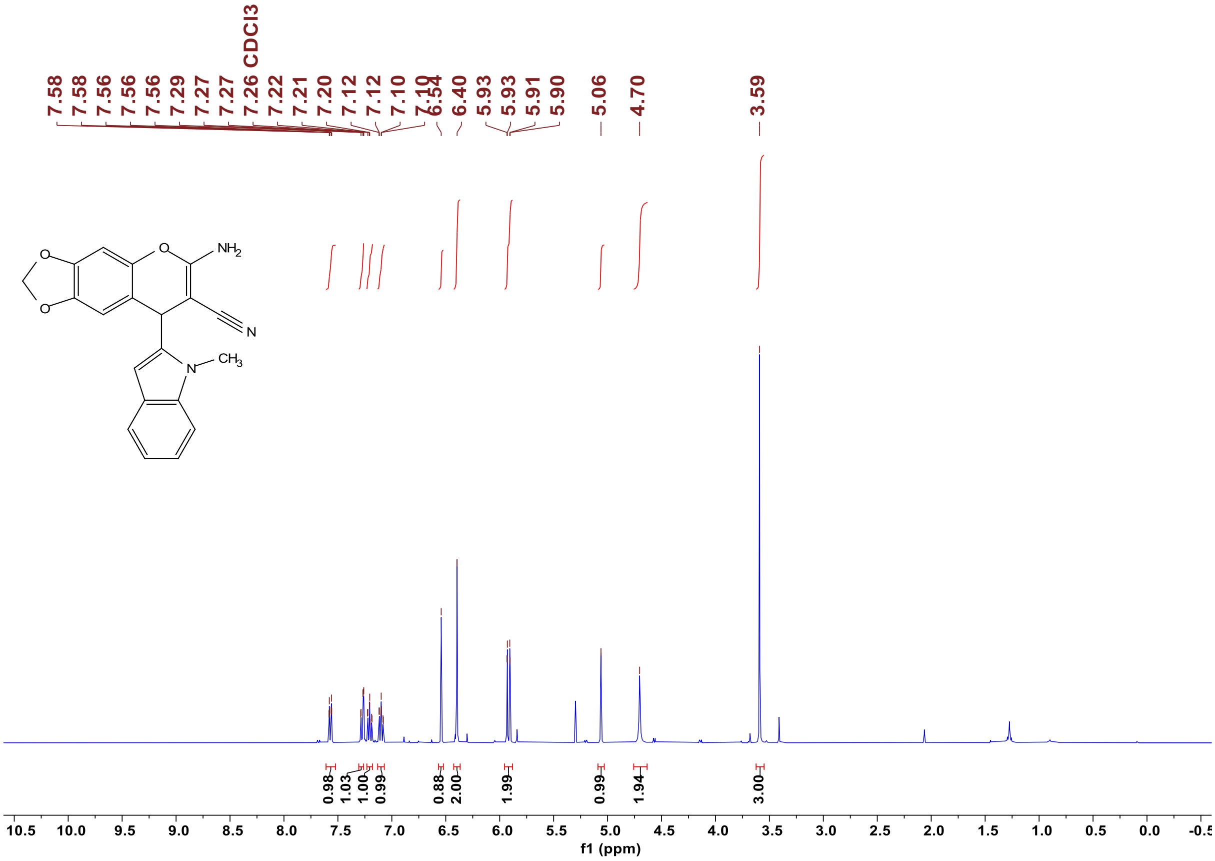

Fig. S39 ^{1}H NMR spectrum of **P2** in $CDCl_3$ (400 MHz) CG504

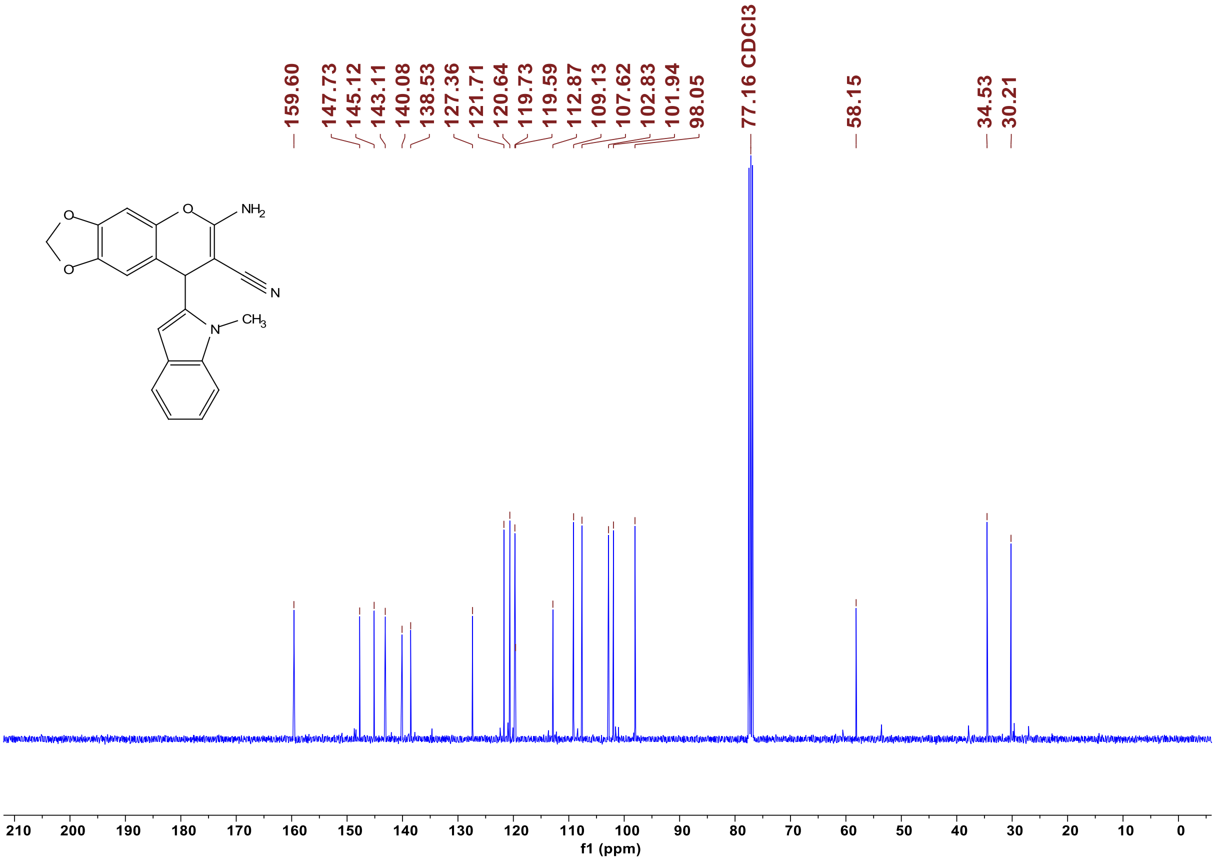

Fig. S40 $^{13}C\{^{1}H\}$ NMR spectrum of **P2** in $CDCl_3$ (101 MHz) CG504

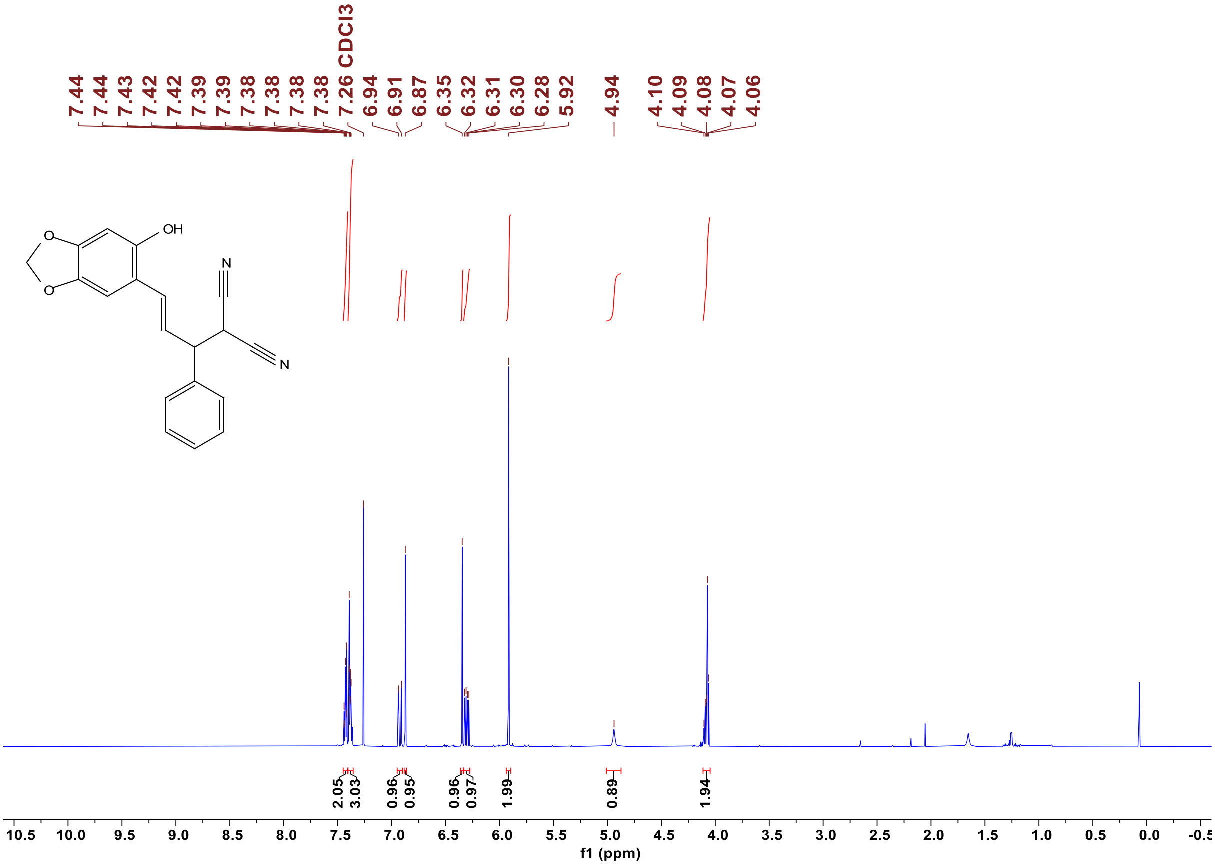

Fig. S41 ^{1}H NMR spectrum of **P3’** in $CDCl_3$ (600 MHz) CG380F1

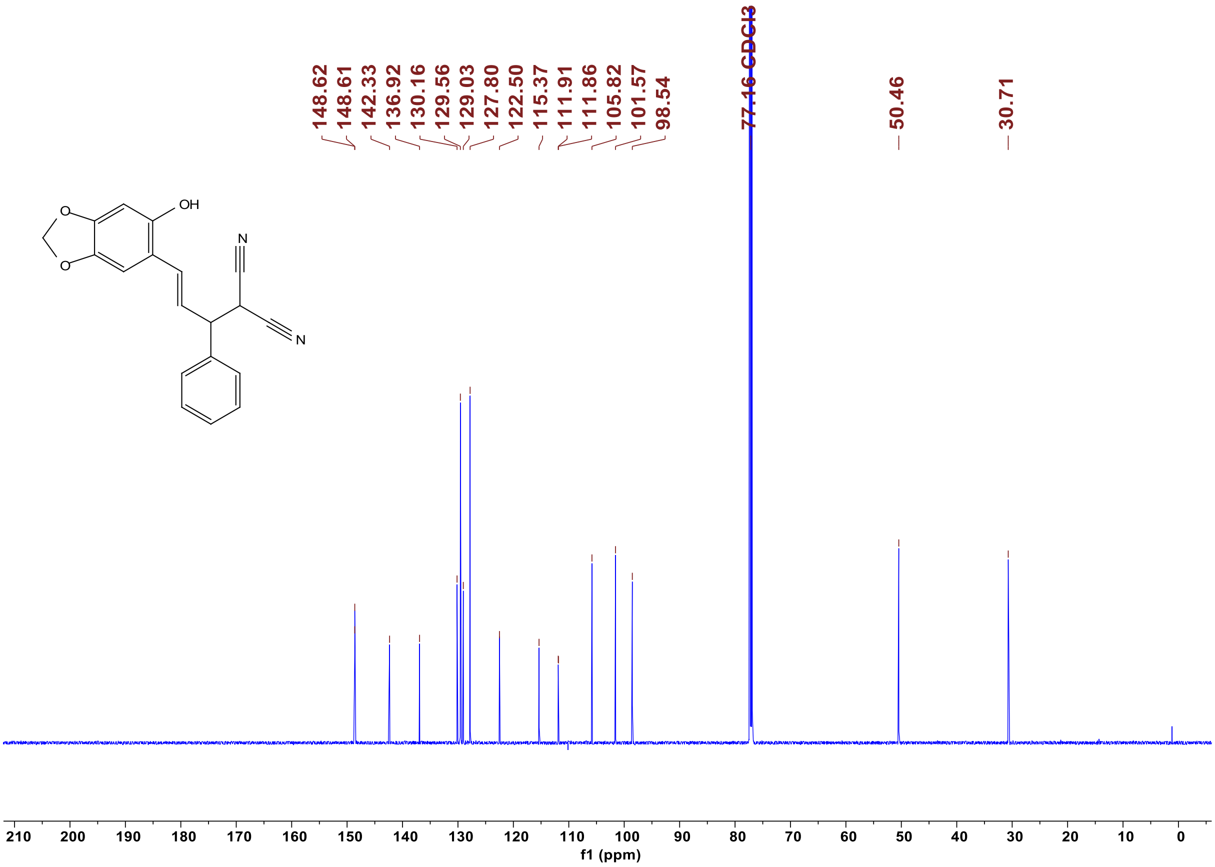

Fig. S42 $^{13}C\{^{1}H\}$ NMR spectrum of **P3’** in $CDCl_3$ (151 MHz) CG380F1

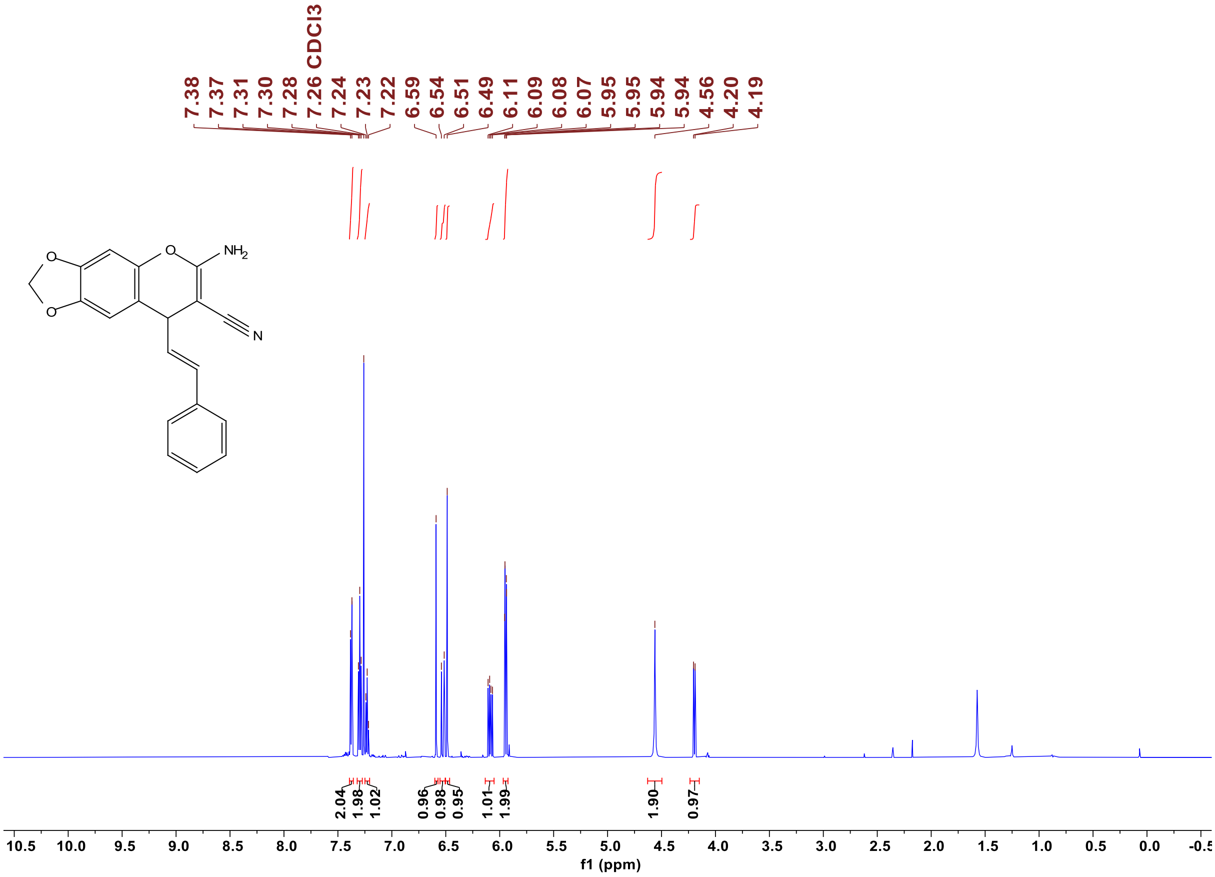

Fig. S43 ^{1}H NMR spectrum of **P3** in $CDCl_3$ (600 MHz) CG380F2

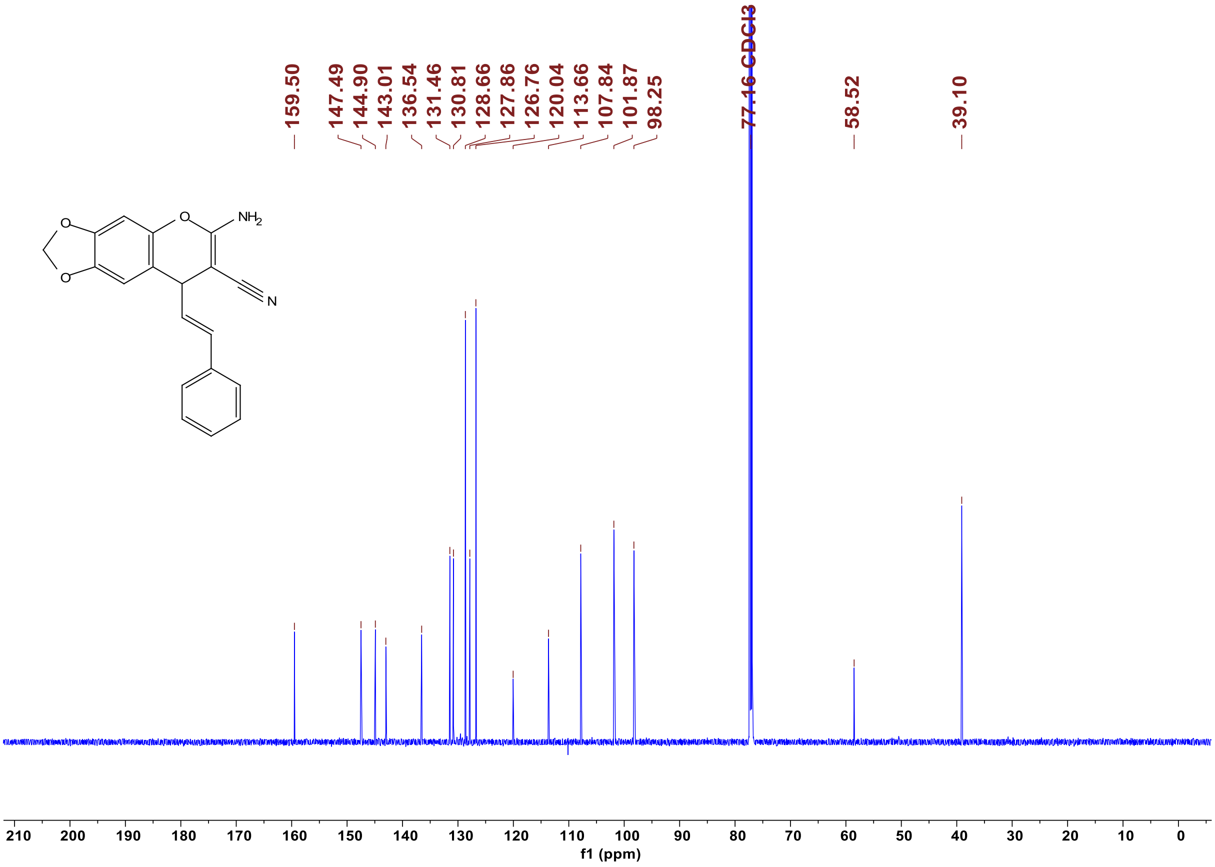

Fig. S44 $^{13}C\{^{1}H\}$ NMR spectrum of **P3** in $CDCl_3$ (151 MHz) CG380F2

14.5 HPLC chromatograms of the (4+2)-cycloaddition products

Chromatogram

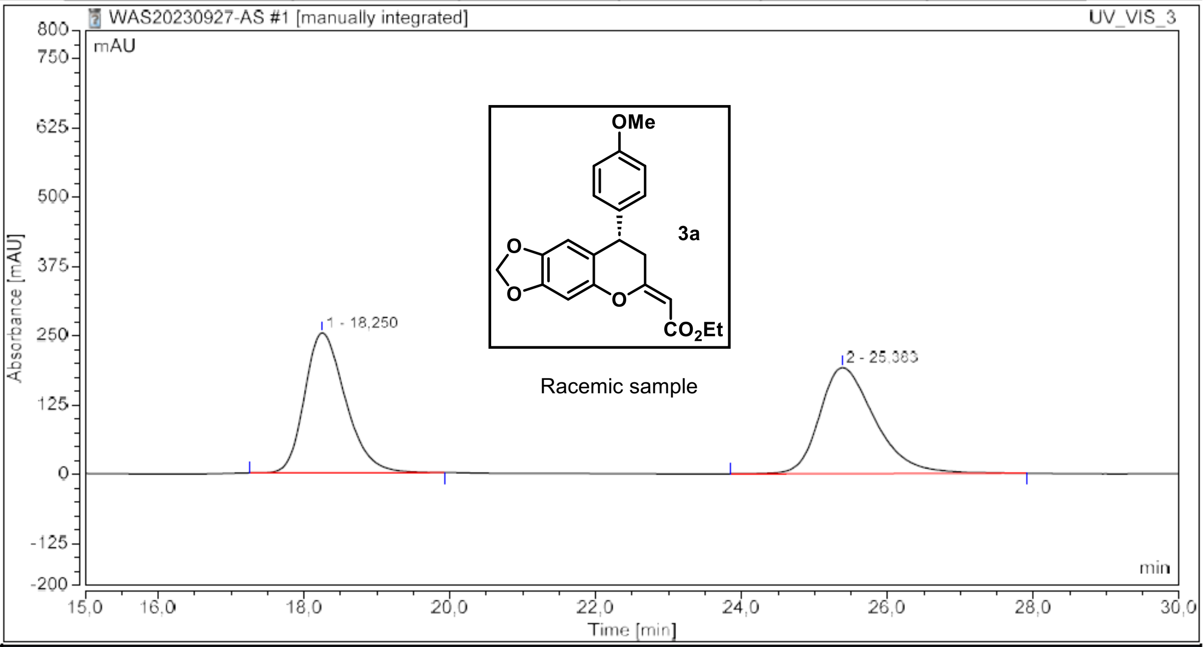

Integration Results

No.	Peak Name	Retention Time<br>min	Area<br>mAU*min	Height<br>mAU	Relative Area<br>%	Relative Height<br>%	Amount<br>n.a.
1		18,250	163,621	252,194	49,29	56,87	n.a.
2		25,383	168,315	191,262	50,71	43,13	n.a.
**Total:**			**331,936**	**443,456**	**100,00**	**100,00**	

Chromatogram

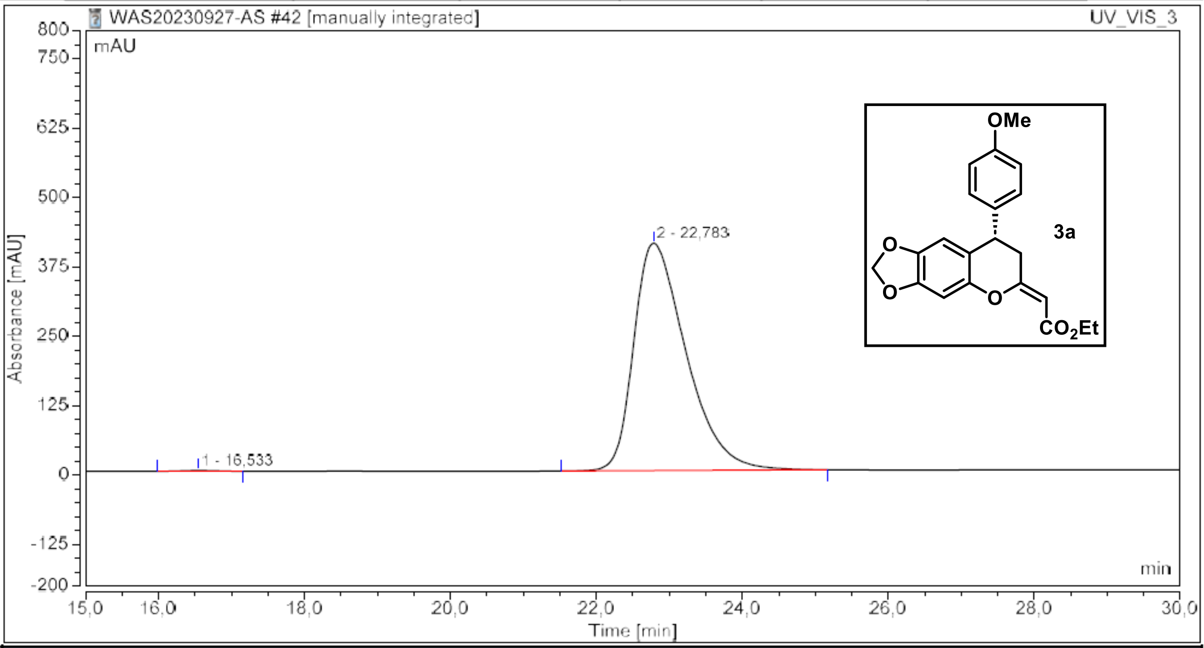

Integration Results

No.	Peak Name	Retention Time<br>min	Area<br>mAU*min	Height<br>mAU	Relative Area<br>%	Relative Height<br>%	Amount<br>n.a.
1		16,533	0,748	1,393	0,22	0,34	n.a.
2		22,783	340,239	409,608	99,78	99,66	n.a.
**Total:**			**340,987**	**411,001**	**100,00**	**100,00**	

Chromatogram

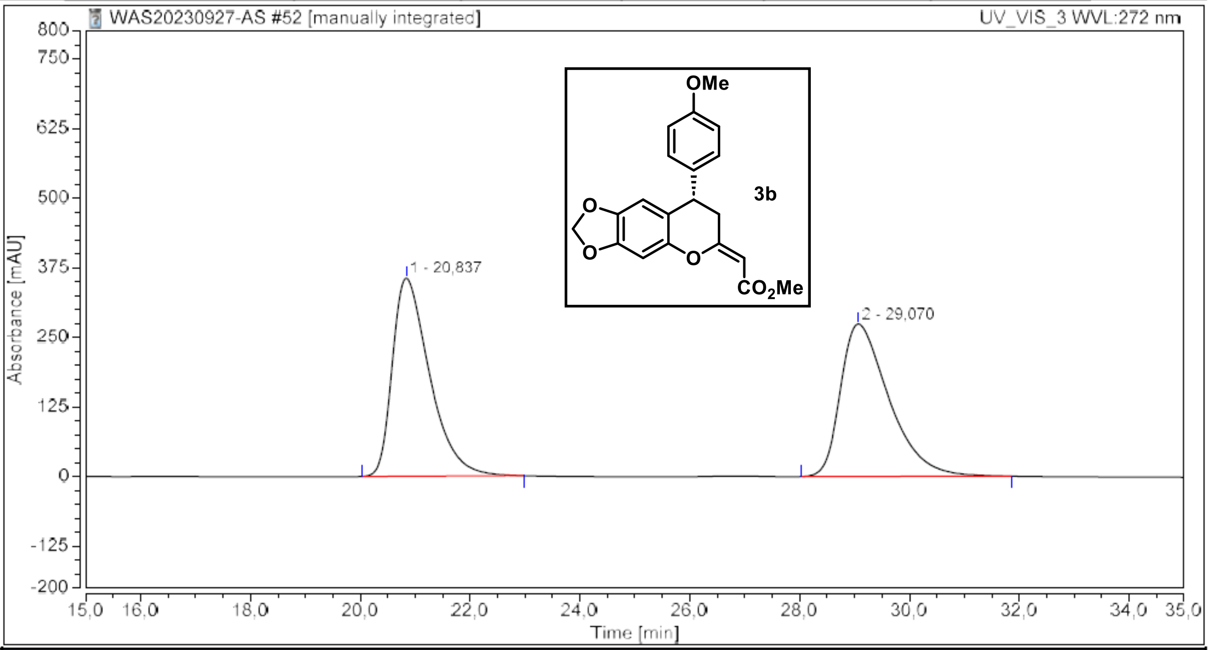

Integration Results

No.	Peak Name	Retention Time min	Area mAU*min	Height mAU	Relative Area %	Relative Height %	Amount n.a.
1		20,837	279,611	355,800	49,97	56,52	n.a.
2		29,070	279,956	273,696	50,03	43,48	n.a.
**Total:**			**559,567**	**629,496**	**100,00**	**100,00**	

Chromatogram

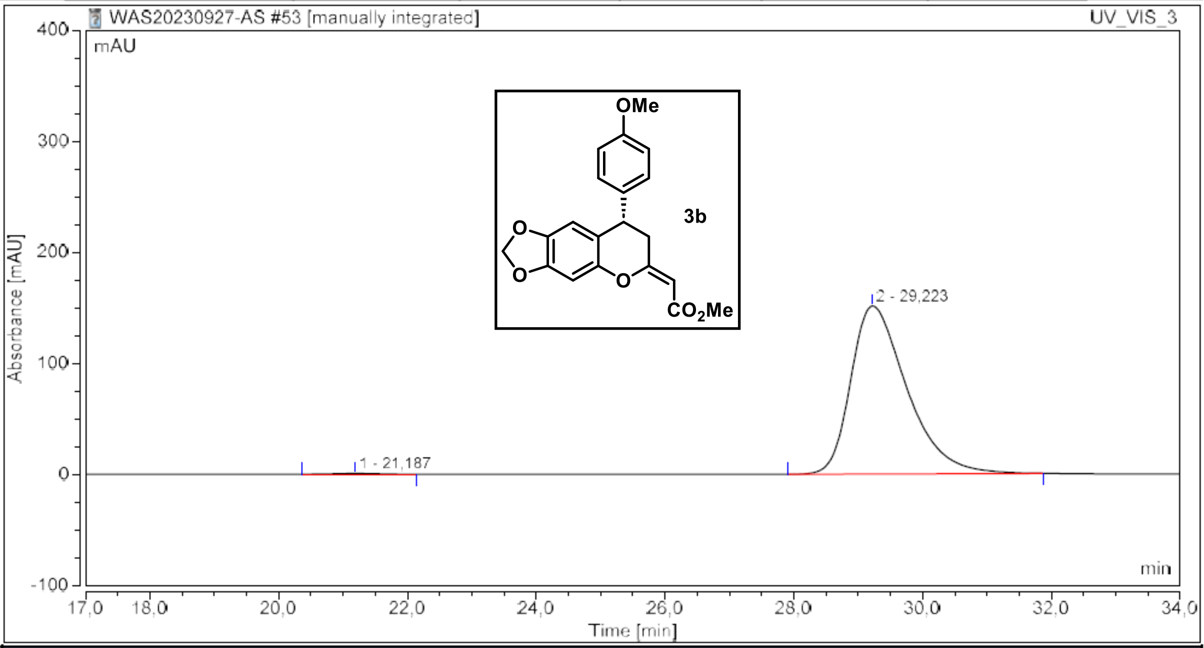

Integration Results

No.	Peak Name	Retention Time min	Area mAU*min	Height mAU	Relative Area %	Relative Height %	Amount n.a.
1		21,187	0,536	0,671	0,35	0,44	n.a.
2		29,223	152,645	151,296	99,65	99,56	n.a.
**Total:**			**153,181**	**151,967**	**100,00**	**100,00**	

Chromatogram

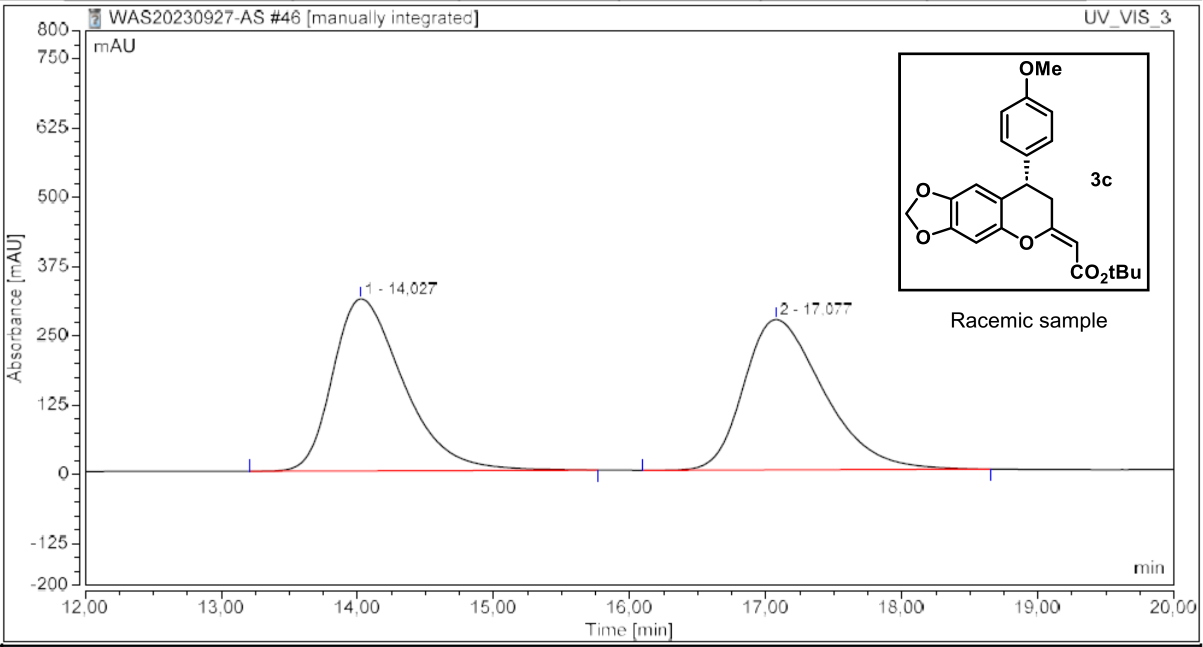

Integration Results

No.	Peak Name	Retention Time min	Area mAU*min	Height mAU	Relative Area %	Relative Height %	Amount n.a.
1		14,027	186,381	310,030	50,26	53,34	n.a.
2		17,077	184,479	271,161	49,74	46,66	n.a.
**Total:**			**370,860**	**581,192**	**100,00**	**100,00**	

Chromatogram

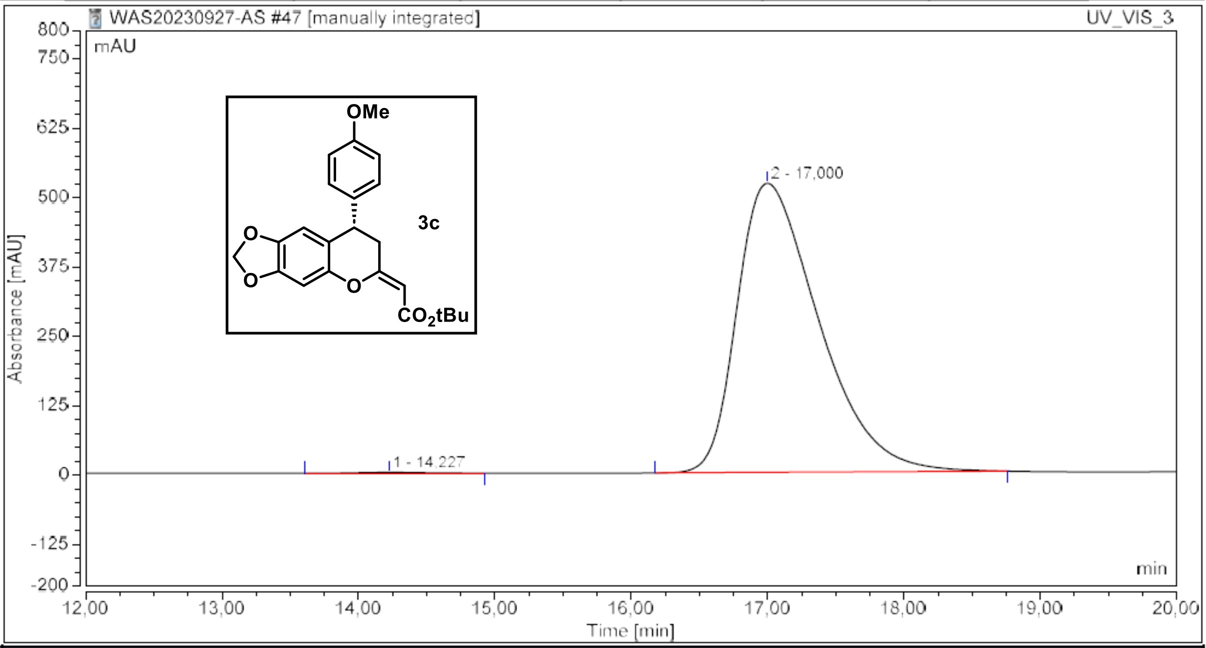

Integration Results

No.	Peak Name	Retention Time min	Area mAU*min	Height mAU	Relative Area %	Relative Height %	Amount n.a.
1		14,227	0,732	1,347	0,20	0,26	n.a.
2		17,000	361,126	520,692	99,80	99,74	n.a.
**Total:**			**361,858**	**522,038**	**100,00**	**100,00**	

Chromatogram

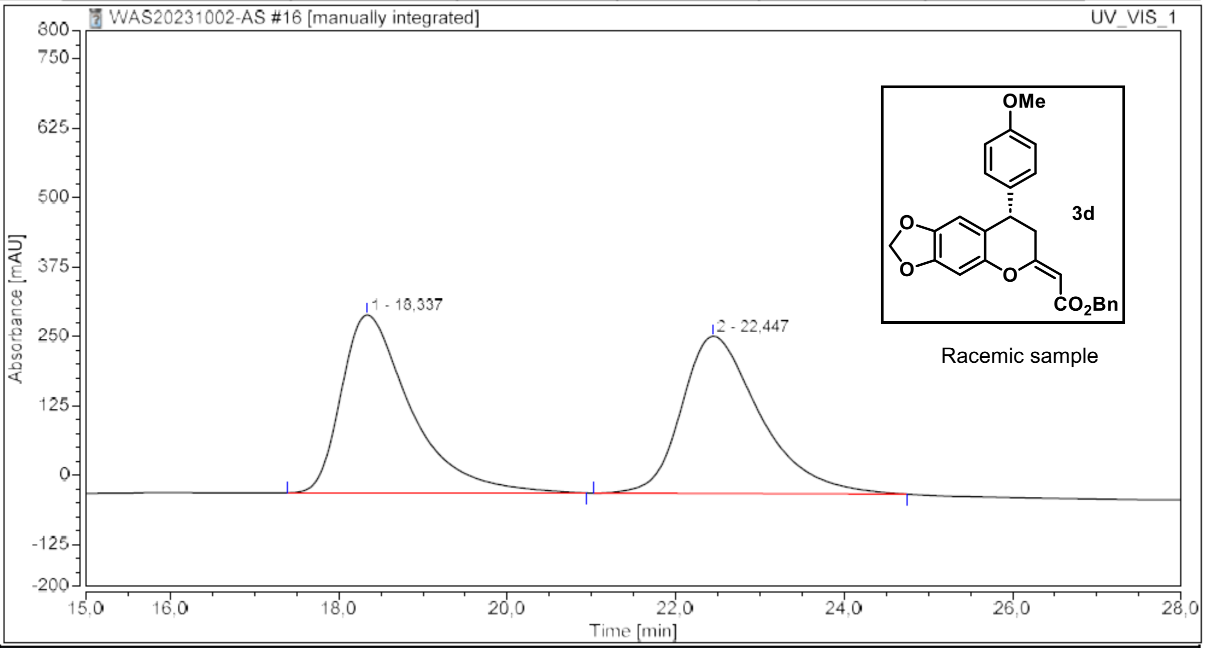

Integration Results

No.	Peak Name	Retention Time<br>min	Area<br>mAU*min	Height<br>mAU	Relative Area<br>%	Relative Height<br>%	Amount<br>n.a.
1		18,337	305,521	319,969	49,94	53,08	n.a.
2		22,447	306,271	282,866	50,06	46,92	n.a.
**Total:**			**611,792**	**602,835**	**100,00**	**100,00**	

Chromatogram

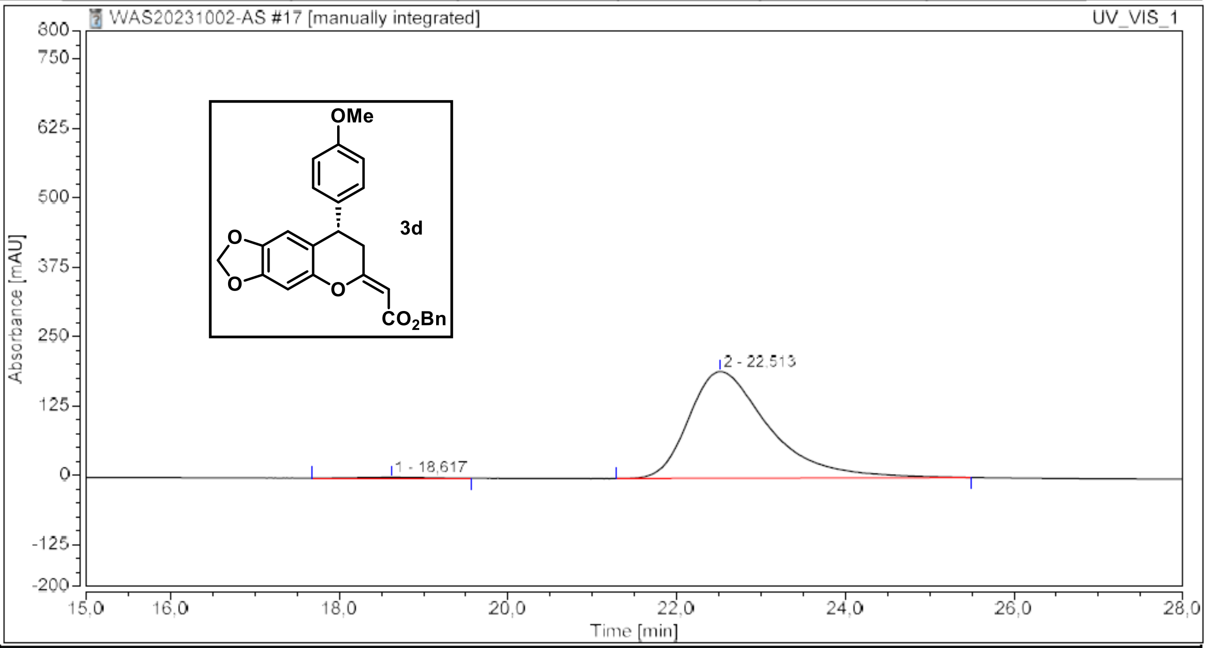

Integration Results

No.	Peak Name	Retention Time<br>min	Area<br>mAU*min	Height<br>mAU	Relative Area<br>%	Relative Height<br>%	Amount<br>n.a.
1		18,617	1,175	1,344	0,55	0,69	n.a.
2		22,513	211,193	192,039	99,45	99,31	n.a.
**Total:**			**212,367**	**193,383**	**100,00**	**100,00**	

Chromatogram

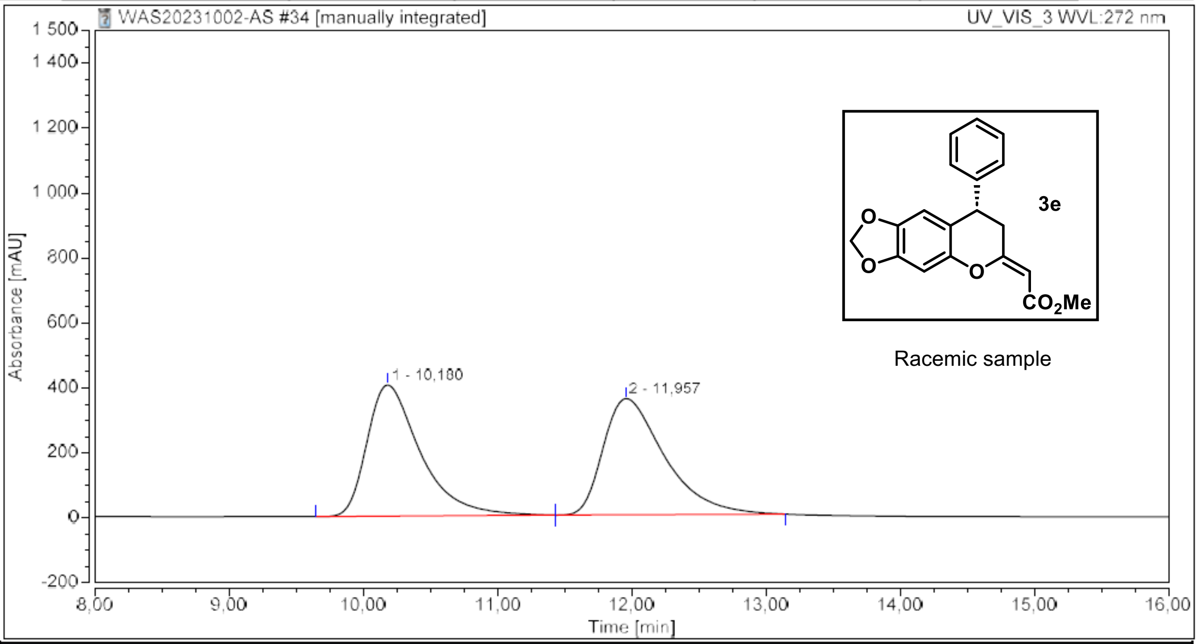

Integration Results

No.	Peak Name	Retention Time min	Area mAU*min	Height mAU	Relative Area %	Relative Height %	Amount n.a.
1		10,180	186,313	403,957	49,54	53,04	n.a.
2		11,957	189,740	357,685	50,46	46,96	n.a.
**Total:**			**376,054**	**761,642**	**100,00**	**100,00**	

Chromatogram

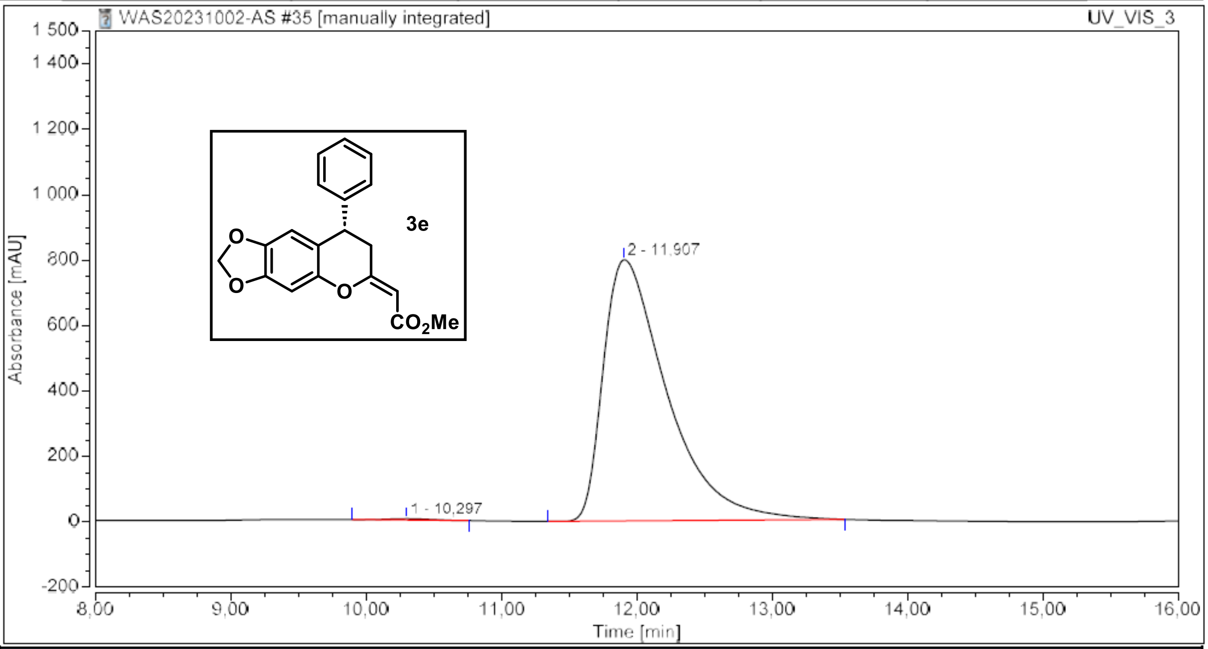

Integration Results

No.	Peak Name	Retention Time min	Area mAU*min	Height mAU	Relative Area %	Relative Height %	Amount n.a.
1		10,297	1,685	4,065	0,39	0,51	n.a.
2		11,907	434,873	798,805	99,61	99,49	n.a.
**Total:**			**436,558**	**802,869**	**100,00**	**100,00**	

Chromatogram

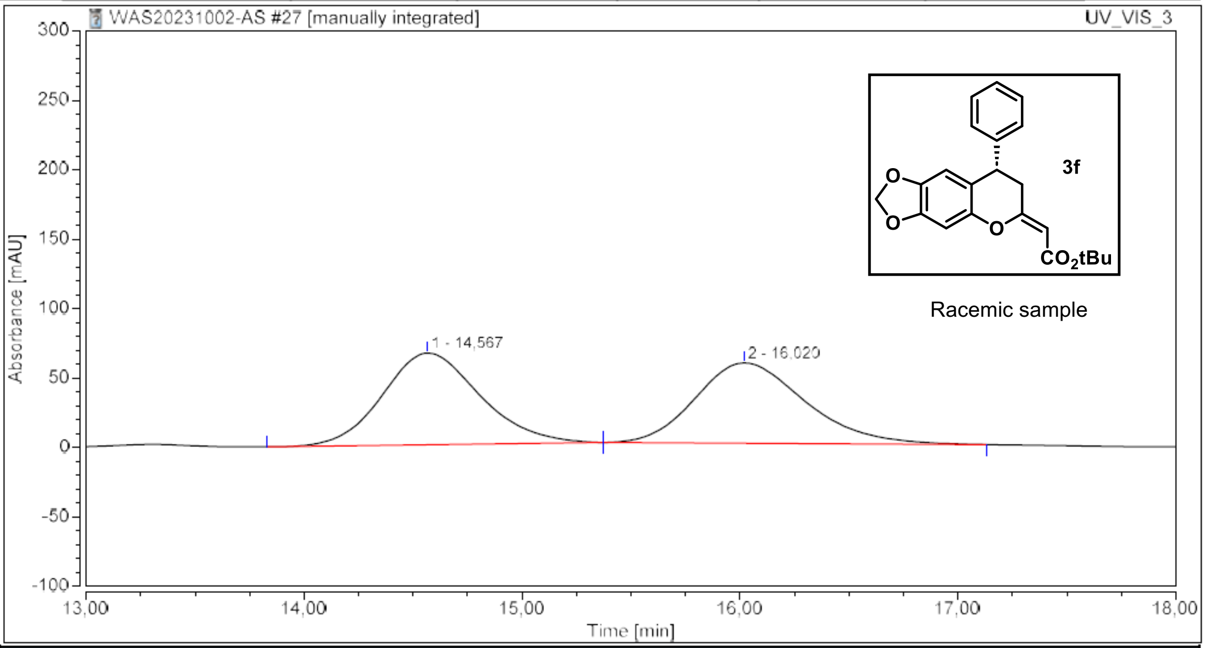

Integration Results

No.	Peak Name	Retention Time min	Area mAU*min	Height mAU	Relative Area %	Relative Height %	Amount n.a.
1		14,567	34,223	65,978	50,37	53,29	n.a.
2		16,020	33,719	57,826	49,63	46,71	n.a.
**Total:**			**67,942**	**123,804**	**100,00**	**100,00**	

Chromatogram

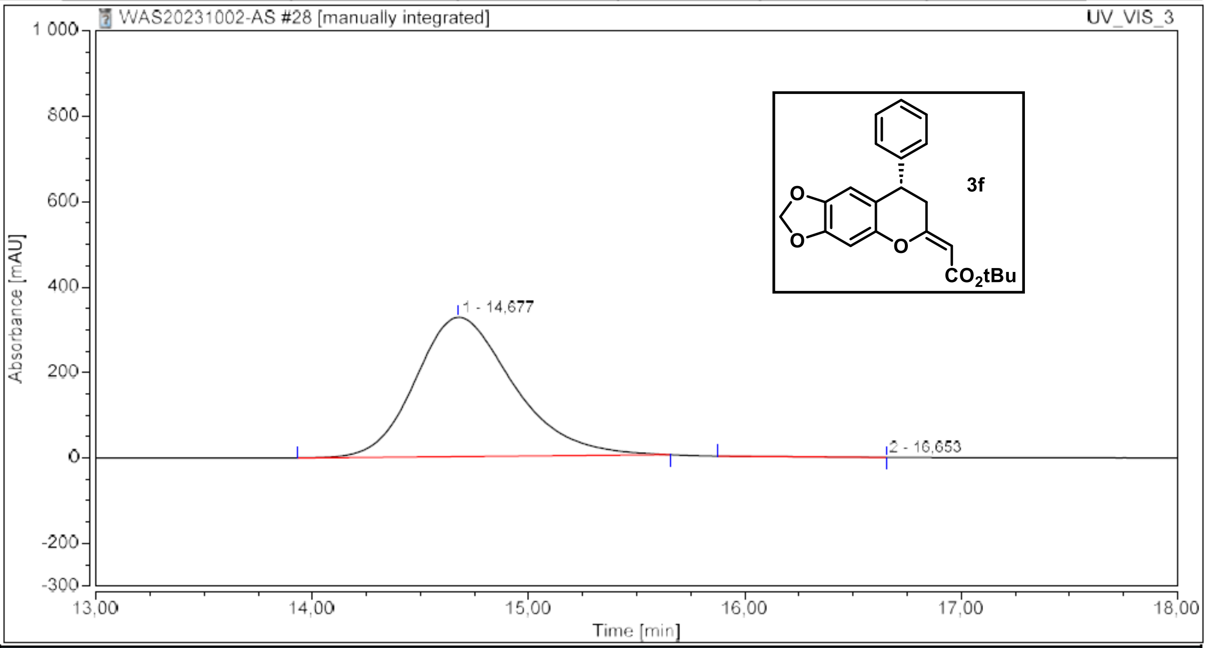

Integration Results

No.	Peak Name	Retention Time min	Area mAU*min	Height mAU	Relative Area %	Relative Height %	Amount n.a.
1		14,677	171,098	325,768	99,90	100,00	n.a.
2		16,653	0,166	0,000	0,10	0,00	n.a.
**Total:**			**171,264**	**325,768**	**100,00**	**100,00**	

Chromatogram

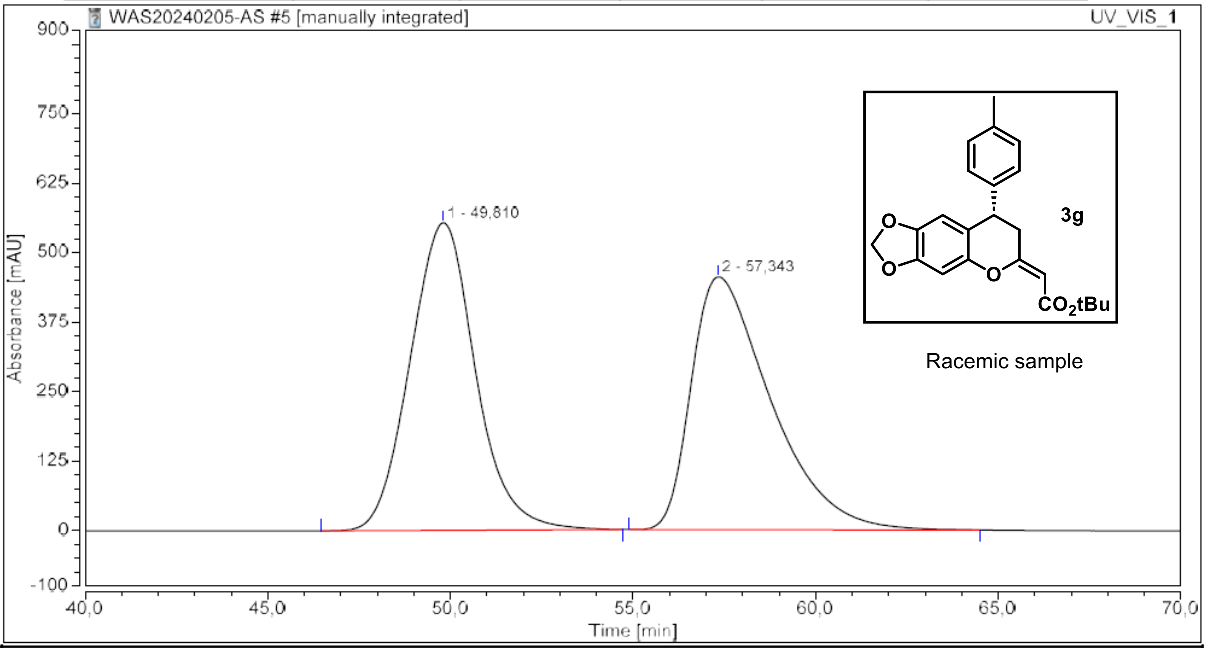

Integration Results

No.	Peak Name	Retention Time min	Area mAU*min	Height mAU	Relative Area %	Relative Height %	Amount n.a.
1		49,810	1146,349	553,373	49,99	54,88	n.a.
2		57,343	1146,827	454,960	50,01	45,12	n.a.
**Total:**			**2293,176**	**1008,333**	**100,00**	**100,00**	

Chromatogram

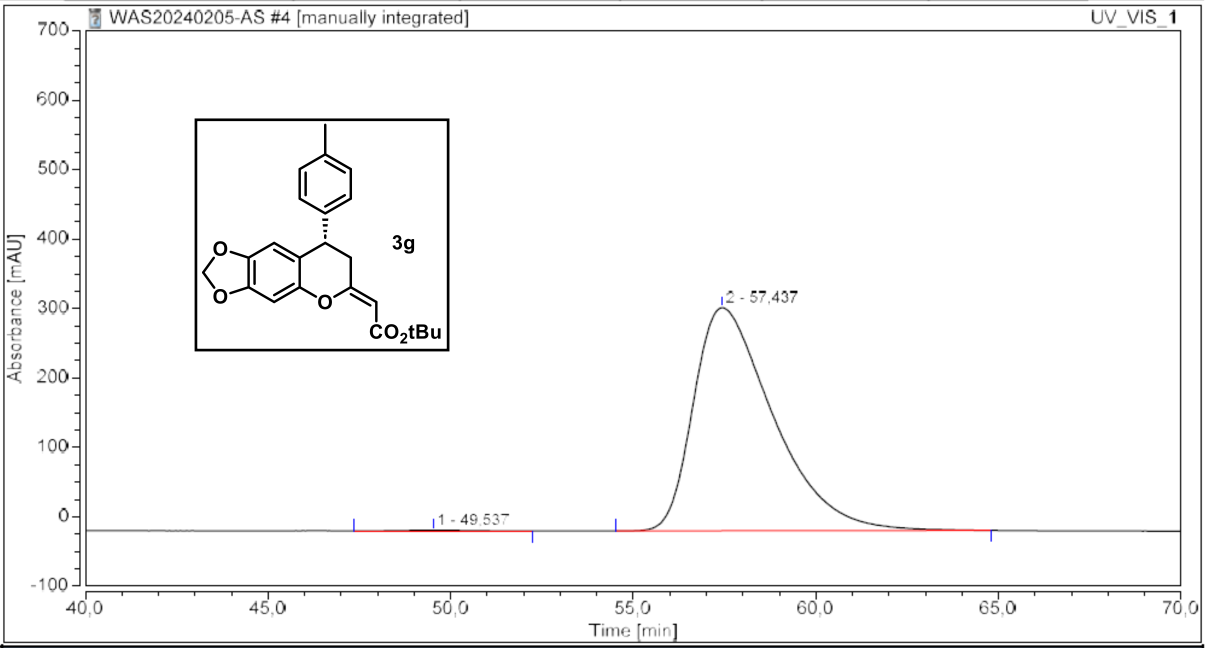

Integration Results

No.	Peak Name	Retention Time min	Area mAU*min	Height mAU	Relative Area %	Relative Height %	Amount n.a.
1		49,537	1,618	0,873	0,20	0,27	n.a.
2		57,437	814,630	321,364	99,80	99,73	n.a.
**Total:**			**816,248**	**322,237**	**100,00**	**100,00**	

Chromatogram

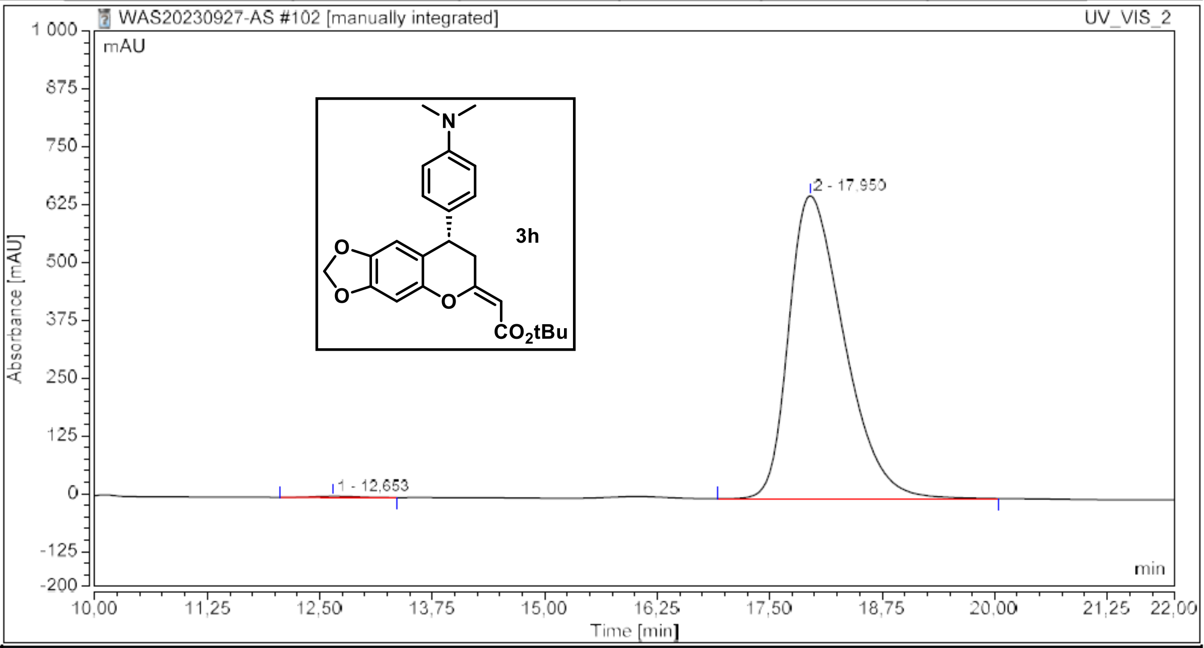

Integration Results

No.	Peak Name	Retention Time min	Area mAU*min	Height mAU	Relative Area %	Relative Height %	Amount n.a.
1		12,653	1,408	2,865	0,30	0,44	n.a.
2		17,950	465,528	654,359	99,70	99,56	n.a.
**Total:**			**466,936**	**657,224**	**100,00**	**100,00**	

Chromatogram

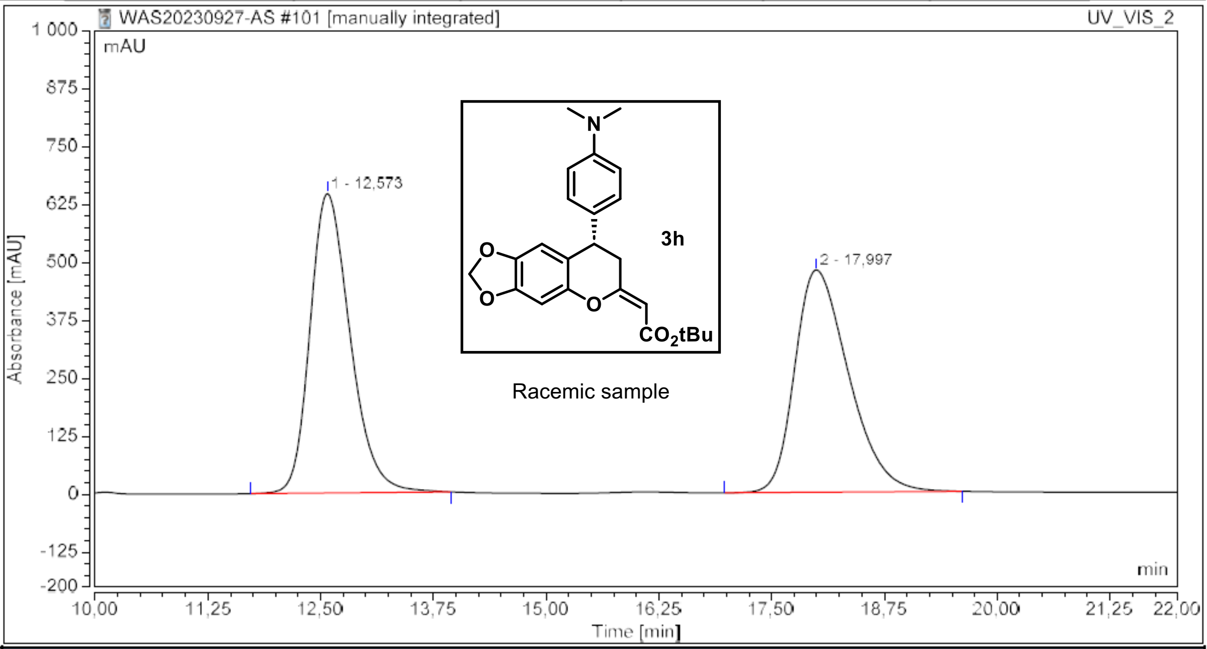

Integration Results

No.	Peak Name	Retention Time min	Area mAU*min	Height mAU	Relative Area %	Relative Height %	Amount n.a.
1		12,573	333,310	646,311	50,09	57,39	n.a.
2		17,997	332,076	479,863	49,91	42,61	n.a.
**Total:**			**665,387**	**1126,175**	**100,00**	**100,00**	

Chromatogram

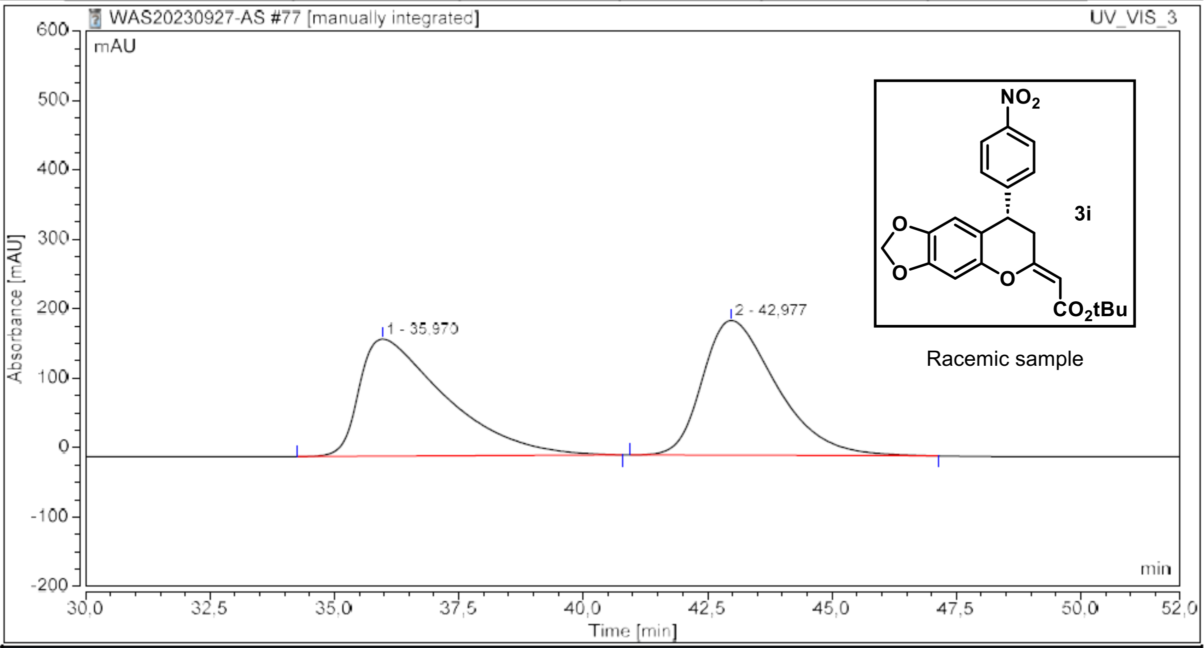

Integration Results

No.	Peak Name	Retention Time min	Area mAU*min	Height mAU	Relative Area %	Relative Height %	Amount n.a.
1		35,970	338,425	168,625	50,21	46,48	n.a.
2		42,977	335,581	194,148	49,79	53,52	n.a.
**Total:**			**674,006**	**362,773**	**100,00**	**100,00**	

Chromatogram

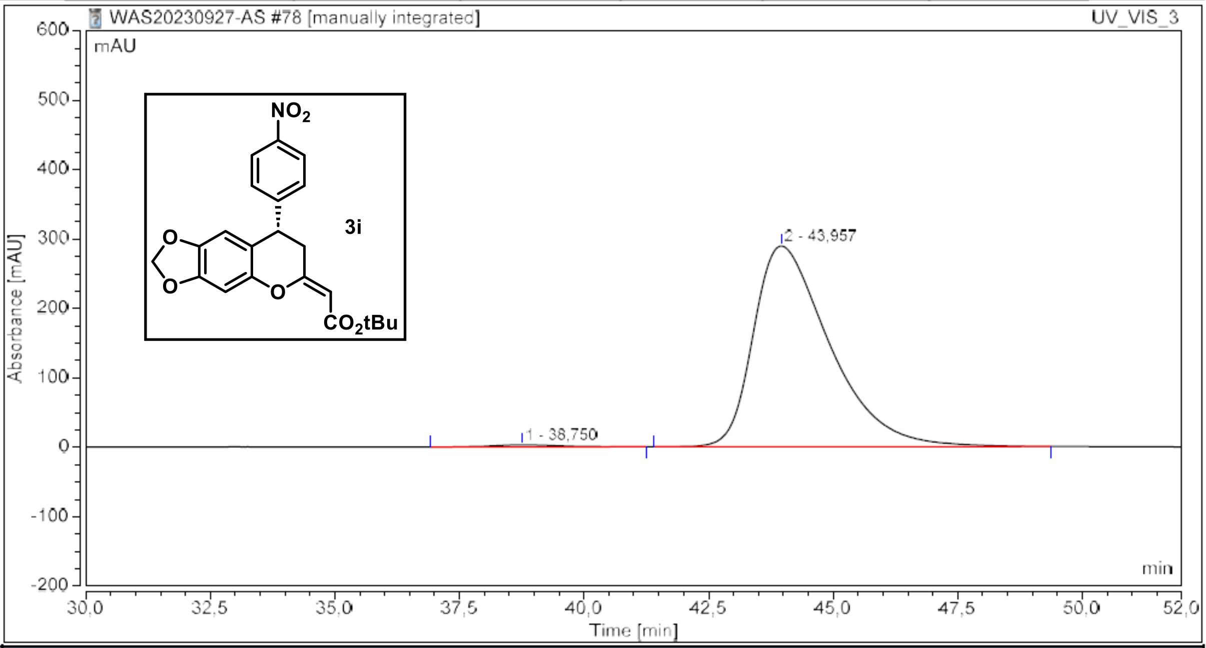

Integration Results

No.	Peak Name	Retention Time min	Area mAU*min	Height mAU	Relative Area %	Relative Height %	Amount n.a.
1		38,750	5,059	3,095	0,97	1,06	n.a.
2		43,957	514,380	289,170	99,03	98,94	n.a.
**Total:**			**519,439**	**292,265**	**100,00**	**100,00**	

Chromatogram

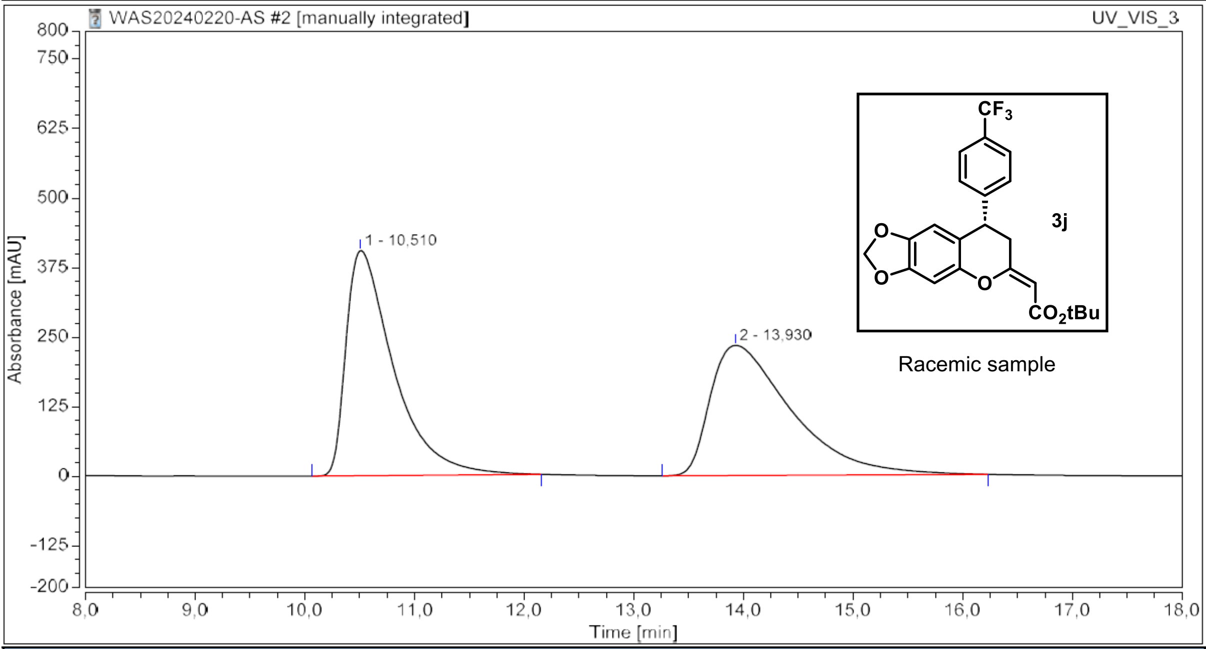

Integration Results

No.	Peak Name	Retention Time<br>min	Area<br>mAU*min	Height<br>mAU	Relative Area<br>%	Relative Height<br>%	Amount<br>n.a.
1		10,510	202,637	404,463	50,28	63,37	n.a.
2		13,930	200,389	233,824	49,72	36,63	n.a.
**Total:**			**403,026**	**638,287**	**100,00**	**100,00**	

Chromatogram

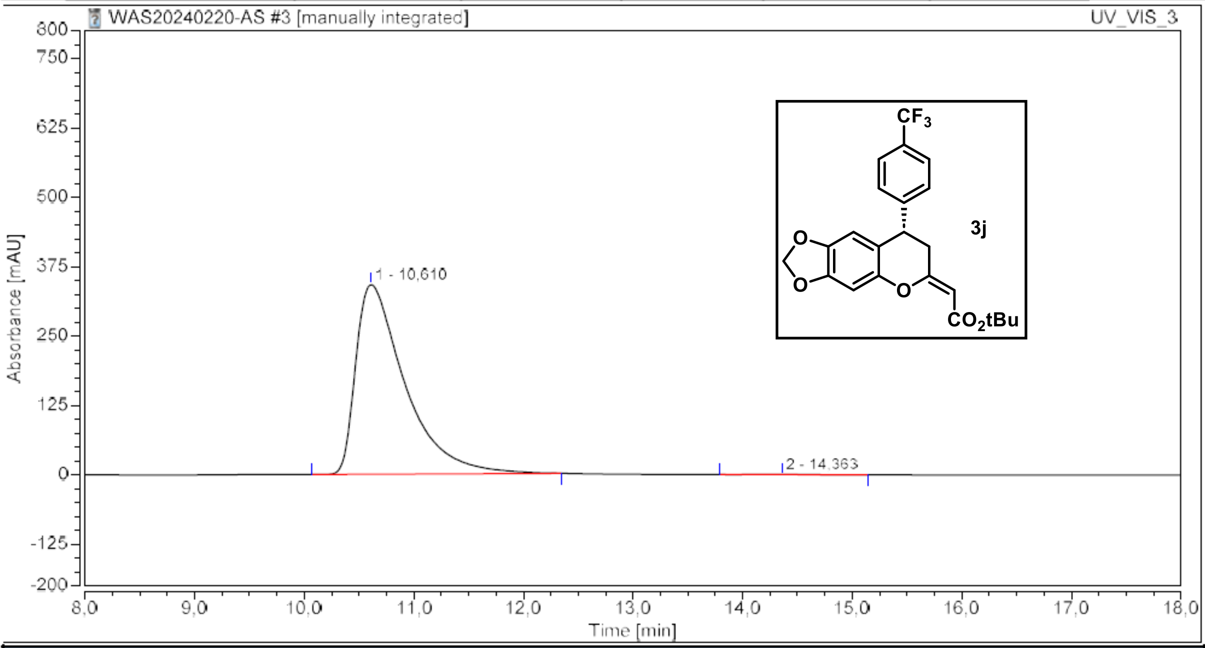

Integration Results

No.	Peak Name	Retention Time<br>min	Area<br>mAU*min	Height<br>mAU	Relative Area<br>%	Relative Height<br>%	Amount<br>n.a.
1		10,610	177,202	341,384	99,86	99,88	n.a.
2		14,363	0,245	0,409	0,14	0,12	n.a.
**Total:**			**177,447**	**341,793**	**100,00**	**100,00**	

Chromatogram

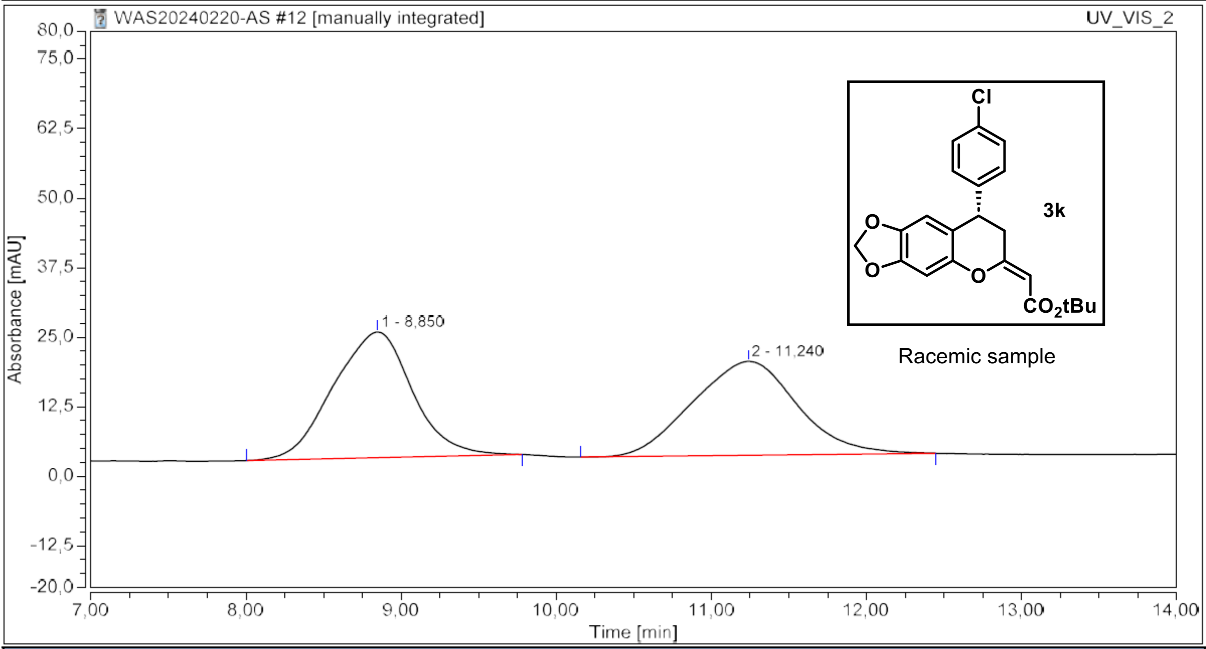

Integration Results

No.	Peak Name	Retention Time min	Area mAU*min	Height mAU	Relative Area %	Relative Height %	Amount n.a.
1		8,850	13,363	22,591	50,36	57,23	n.a.
2		11,240	13,172	16,881	49,64	42,77	n.a.
**Total:**			**26,535**	**39,472**	**100,00**	**100,00**	

Chromatogram

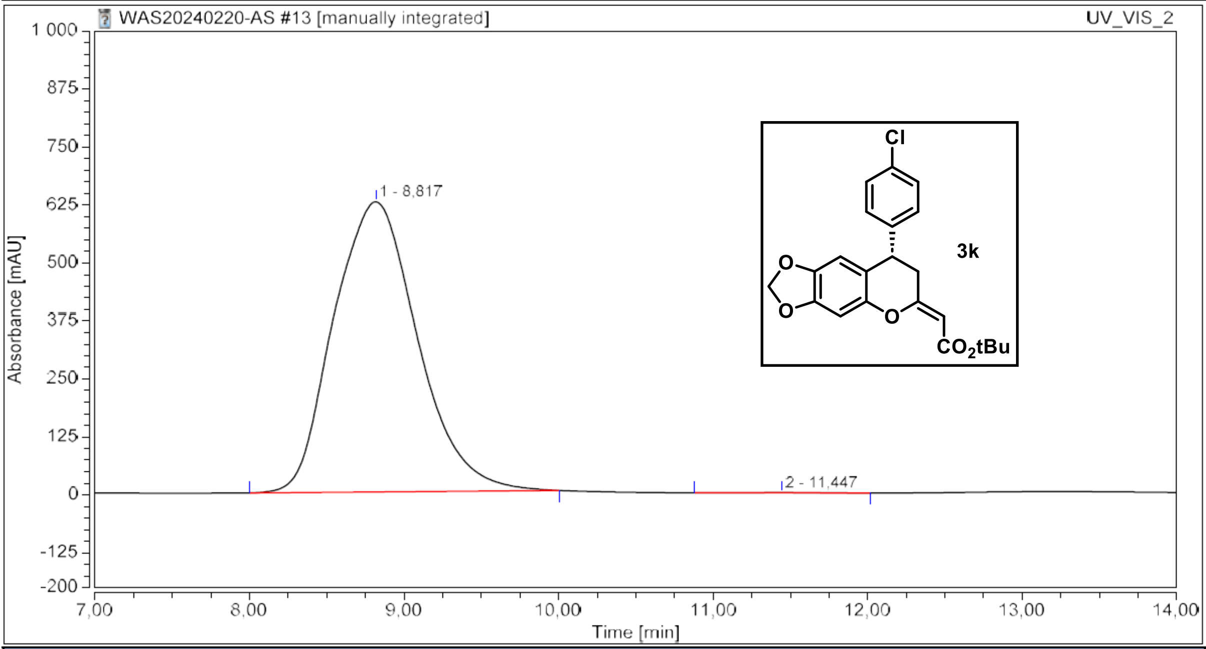

Integration Results

No.	Peak Name	Retention Time min	Area mAU*min	Height mAU	Relative Area %	Relative Height %	Amount n.a.
1		8,817	393,329	625,620	99,92	99,91	n.a.
2		11,447	0,320	0,559	0,08	0,09	n.a.
**Total:**			**393,648**	**626,179**	**100,00**	**100,00**	

Chromatogram

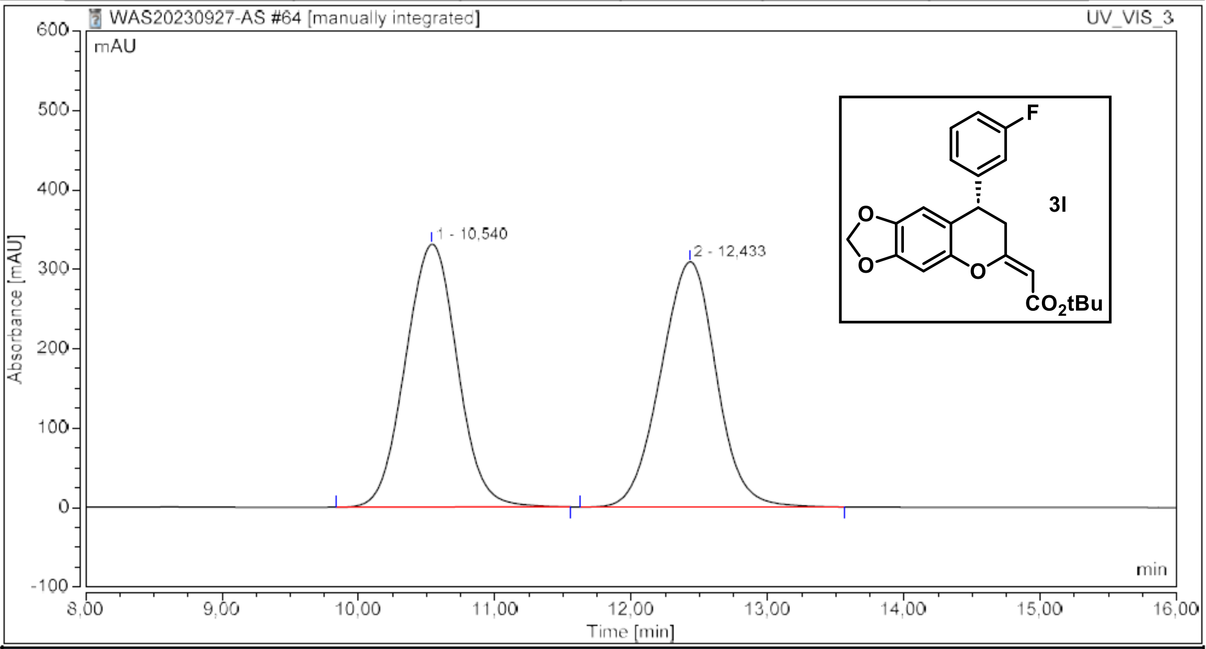

Integration Results

No.	Peak Name	Retention Time min	Area mAU*min	Height mAU	Relative Area %	Relative Height %	Amount n.a.
1		10,540	146,094	331,118	50,05	51,75	n.a.
2		12,433	145,791	308,676	49,95	48,25	n.a.
**Total:**			**291,885**	**639,794**	**100,00**	**100,00**	

Chromatogram

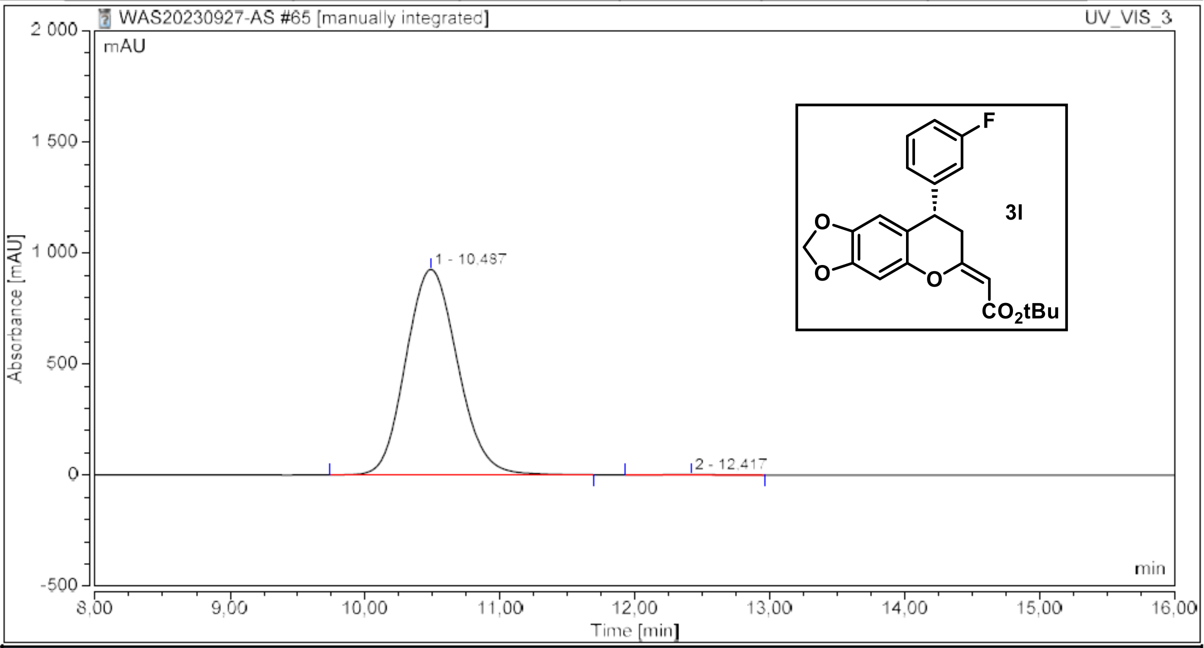

Integration Results

No.	Peak Name	Retention Time min	Area mAU*min	Height mAU	Relative Area %	Relative Height %	Amount n.a.
1		10,487	410,106	925,163	99,86	99,85	n.a.
2		12,417	0,583	1,427	0,14	0,15	n.a.
**Total:**			**410,688**	**926,590**	**100,00**	**100,00**	

Chromatogram

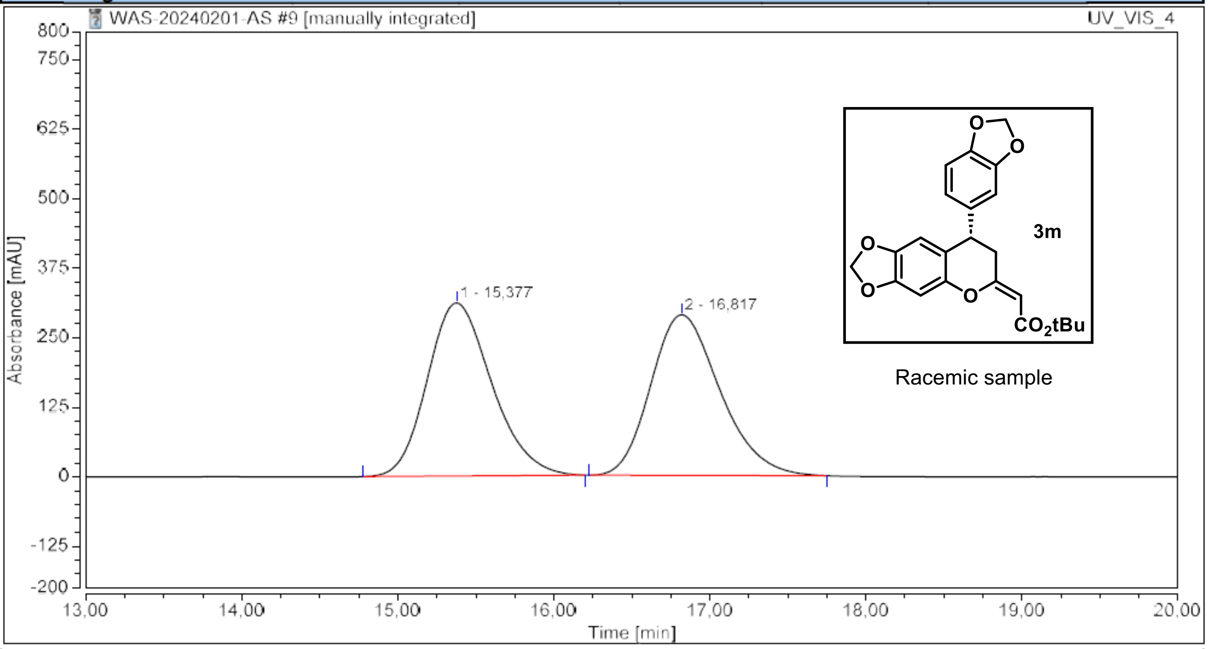

Integration Results

No.	Peak Name	Retention Time min	Area mAU*min	Height mAU	Relative Area %	Relative Height %	Amount n.a.
1		15,377	149,208	311,198	50,12	51,86	n.a.
2		16,817	148,485	288,852	49,88	48,14	n.a.
**Total:**			**297,693**	**600,049**	**100,00**	**100,00**	

Chromatogram

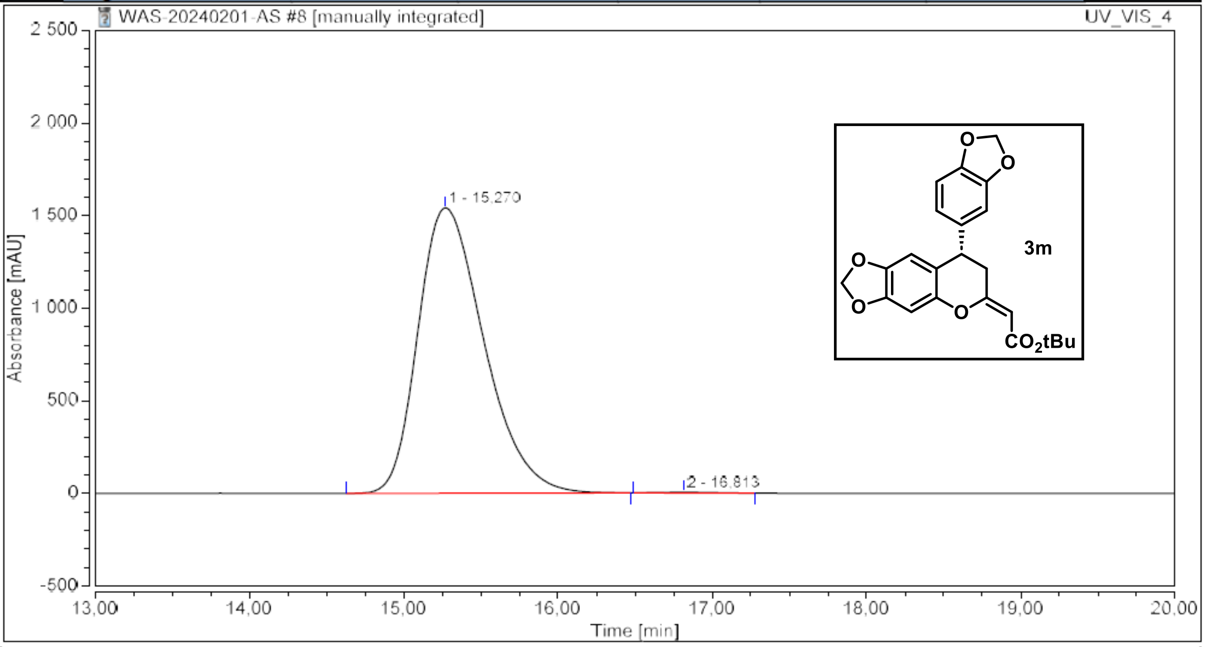

Integration Results

No.	Peak Name	Retention Time min	Area mAU*min	Height mAU	Relative Area %	Relative Height %	Amount n.a.
1		15,270	762,714	1540,000	99,83	99,80	n.a.
2		16,813	1,314	3,147	0,17	0,20	n.a.
**Total:**			**764,028**	**1543,148**	**100,00**	**100,00**	

Chromatogram

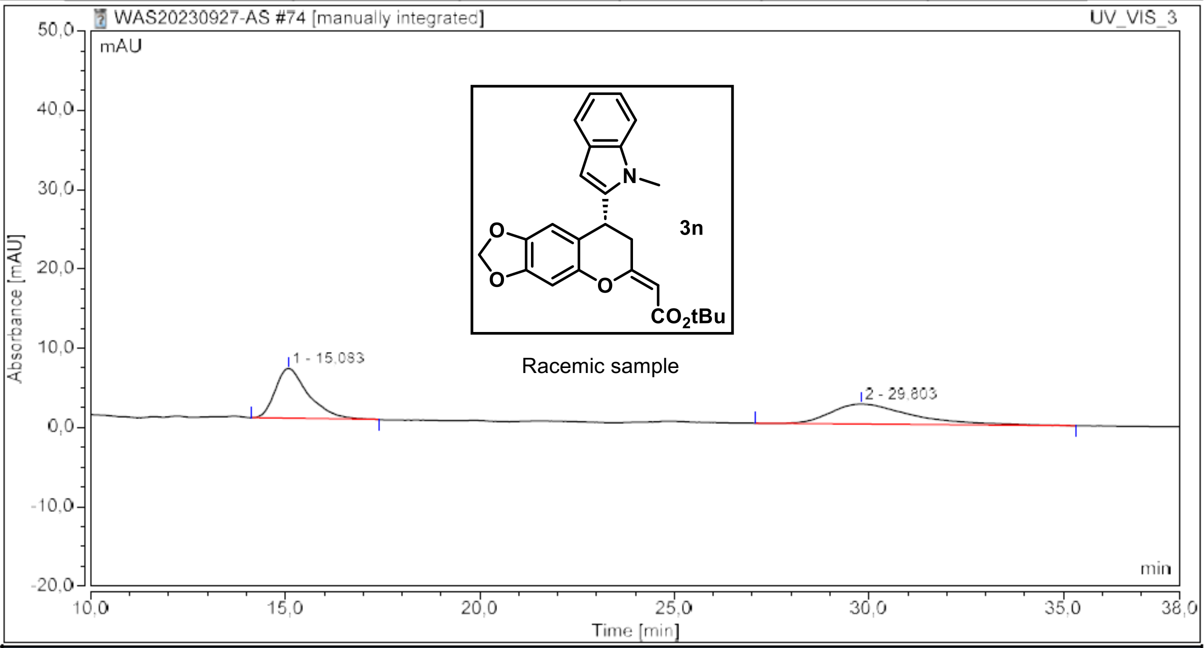

Integration Results

No.	Peak Name	Retention Time min	Area mAU*min	Height mAU	Relative Area %	Relative Height %	Amount n.a.
1		15,083	6,188	6,287	50,77	71,51	n.a.
2		29,803	5,999	2,504	49,23	28,49	n.a.
**Total:**			**12,187**	**8,791**	**100,00**	**100,00**	

Chromatogram

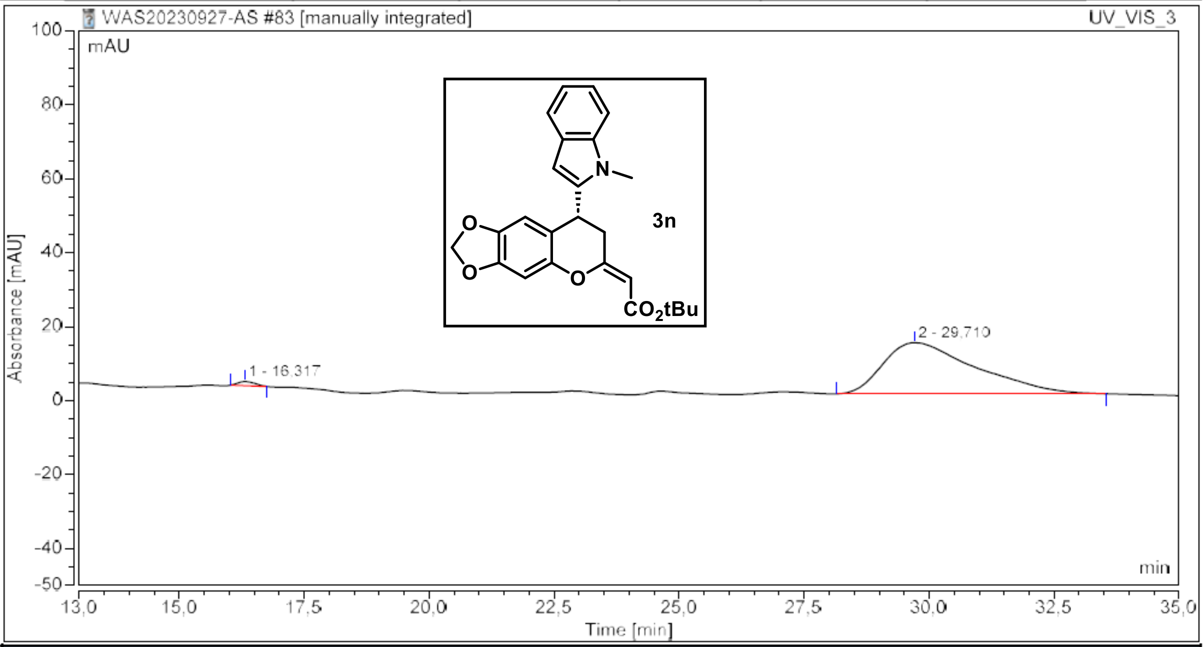

Integration Results

No.	Peak Name	Retention Time min	Area mAU*min	Height mAU	Relative Area %	Relative Height %	Amount n.a.
1		16,317	0,439	1,127	1,46	7,52	n.a.
2		29,710	29,705	13,872	98,54	92,48	n.a.
**Total:**			**30,145**	**14,999**	**100,00**	**100,00**	

Chromatogram

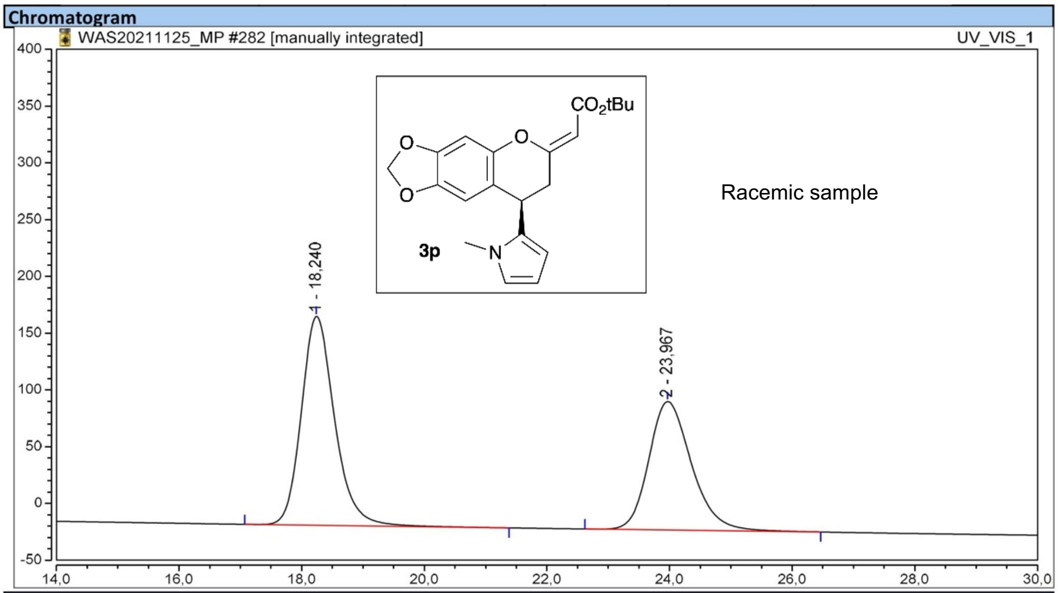

Integration Results

No.	Peak Name	Retention Time<br>min	Area<br>mAU*min	Height<br>mAU	Relative Area<br>%	Relative Height<br>%	Amount<br>n.a.
1		18,240	116,124	184,084	55,95	61,92	n.a.
2		23,967	91,425	113,212	44,05	38,08	n.a.
**Total:**			**207,550**	**297,296**	**100,00**	**100,00**	

Chromatogram

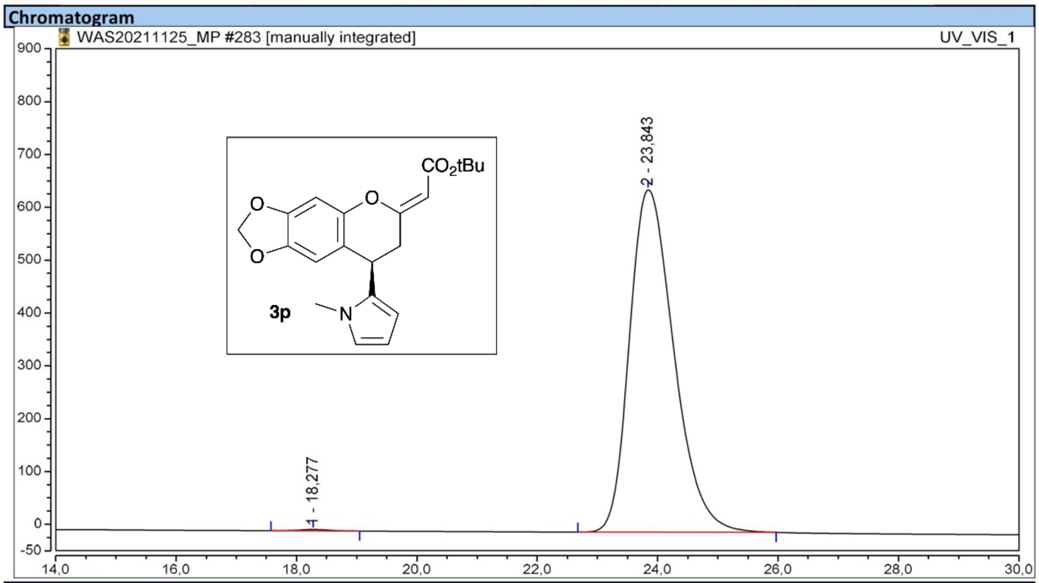

Integration Results

No.	Peak Name	Retention Time<br>min	Area<br>mAU*min	Height<br>mAU	Relative Area<br>%	Relative Height<br>%	Amount<br>n.a.
1		18,277	1,547	2,569	0,28	0,39	n.a.
2		23,843	553,032	648,124	99,72	99,61	n.a.
**Total:**			**554,579**	**650,692**	**100,00**	**100,00**	

Chromatogram

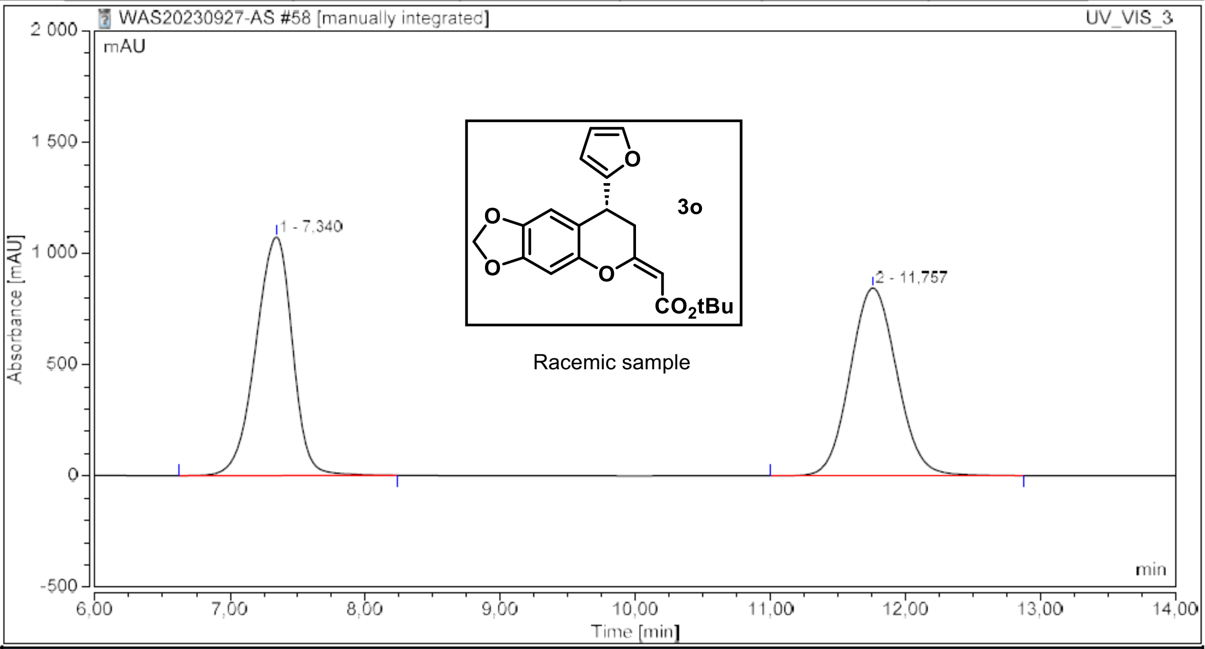

Integration Results

No.	Peak Name	Retention Time min	Area mAU*min	Height mAU	Relative Area %	Relative Height %	Amount n.a.
1		7,340	337,109	1073,291	49,90	55,98	n.a.
2		11,757	338,429	844,153	50,10	44,02	n.a.
**Total:**			**675,538**	**1917,444**	**100,00**	**100,00**	

Chromatogram

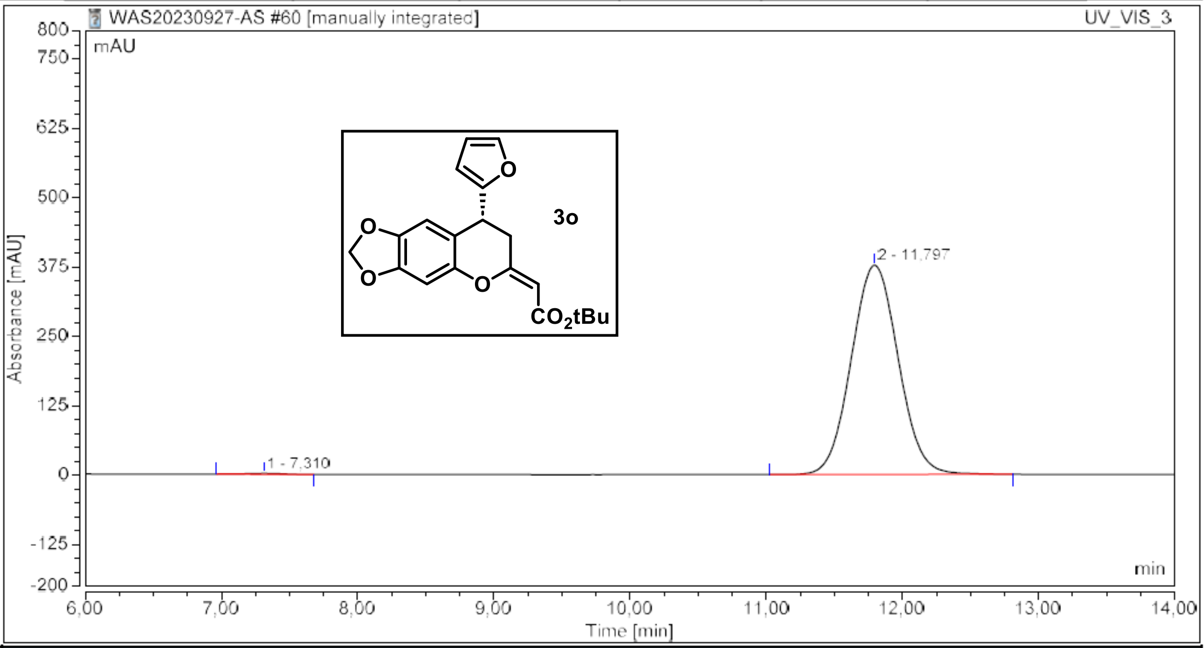

Integration Results

No.	Peak Name	Retention Time min	Area mAU*min	Height mAU	Relative Area %	Relative Height %	Amount n.a.
1		7,310	0,447	1,391	0,30	0,37	n.a.
2		11,797	150,656	376,819	99,70	99,63	n.a.
**Total:**			**151,103**	**378,209**	**100,00**	**100,00**	

Chromatogram

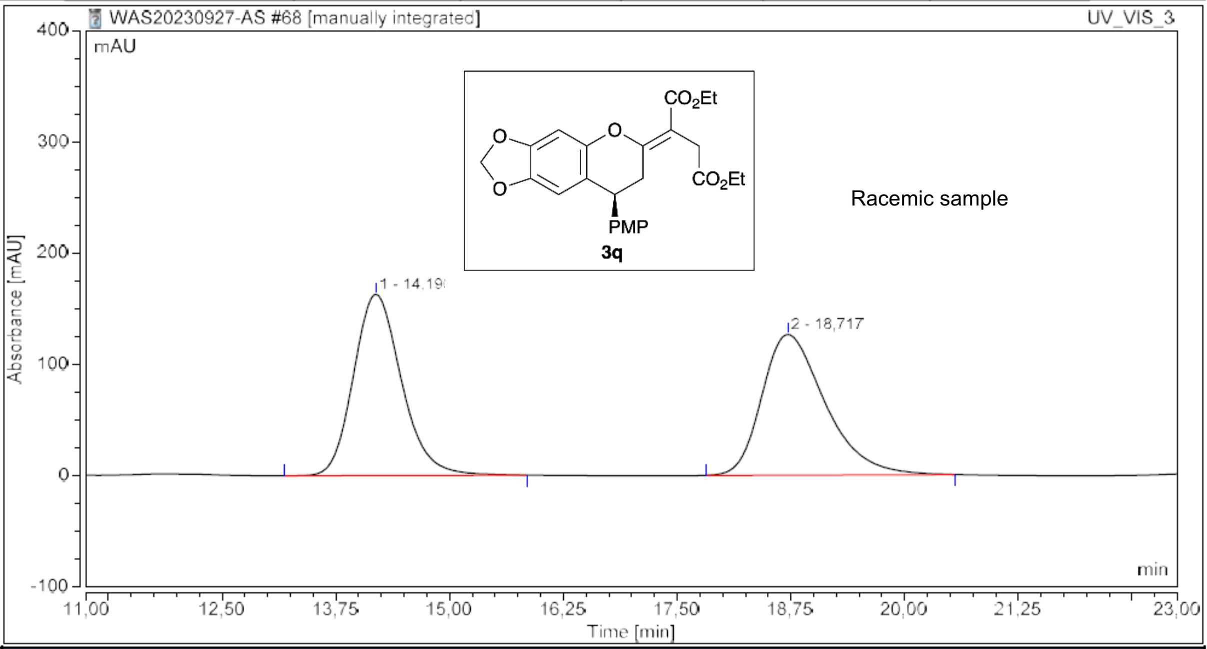

Integration Results

No.	Peak Name	Retention Time min	Area mAU*min	Height mAU	Relative Area %	Relative Height %	Amount n.a.
1		14,190	99,523	162,852	49,43	56,29	n.a.
2		18,717	101,807	126,437	50,57	43,71	n.a.
**Total:**			**201,329**	**289,289**	**100,00**	**100,00**	

Chromatogram

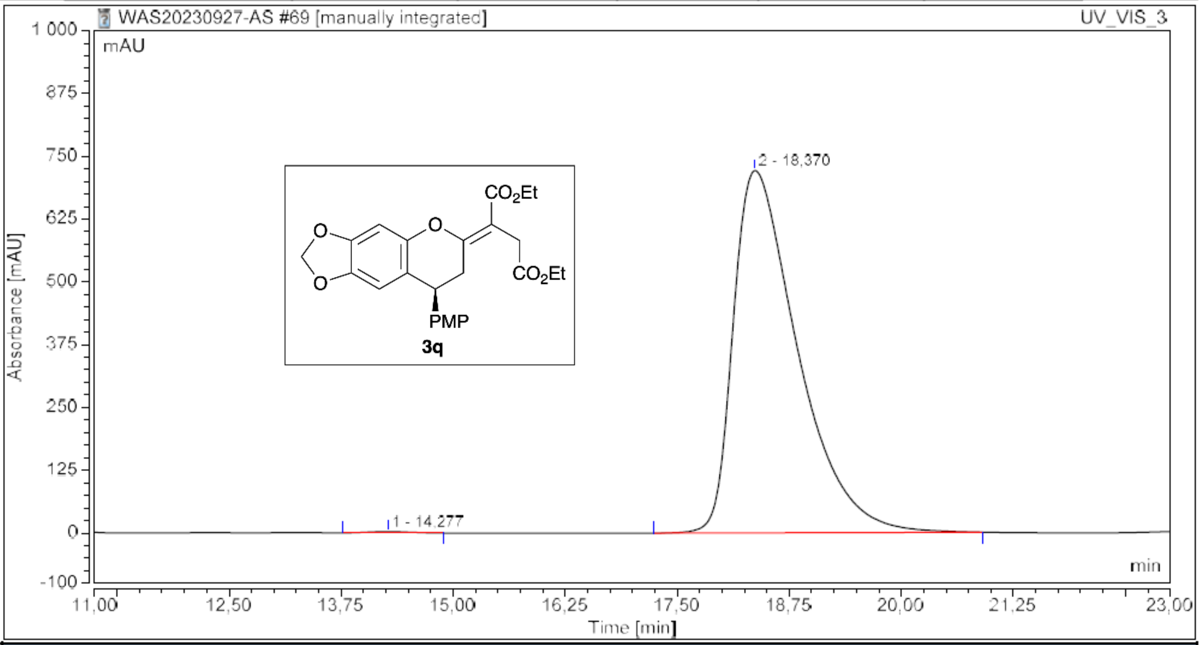

Integration Results

No.	Peak Name	Retention Time min	Area mAU*min	Height mAU	Relative Area %	Relative Height %	Amount n.a.
1		14,277	1,319	2,440	0,22	0,34	n.a.
2		18,370	599,064	720,885	99,78	99,66	n.a.
**Total:**			**600,383**	**723,325**	**100,00**	**100,00**	

Chromatogram

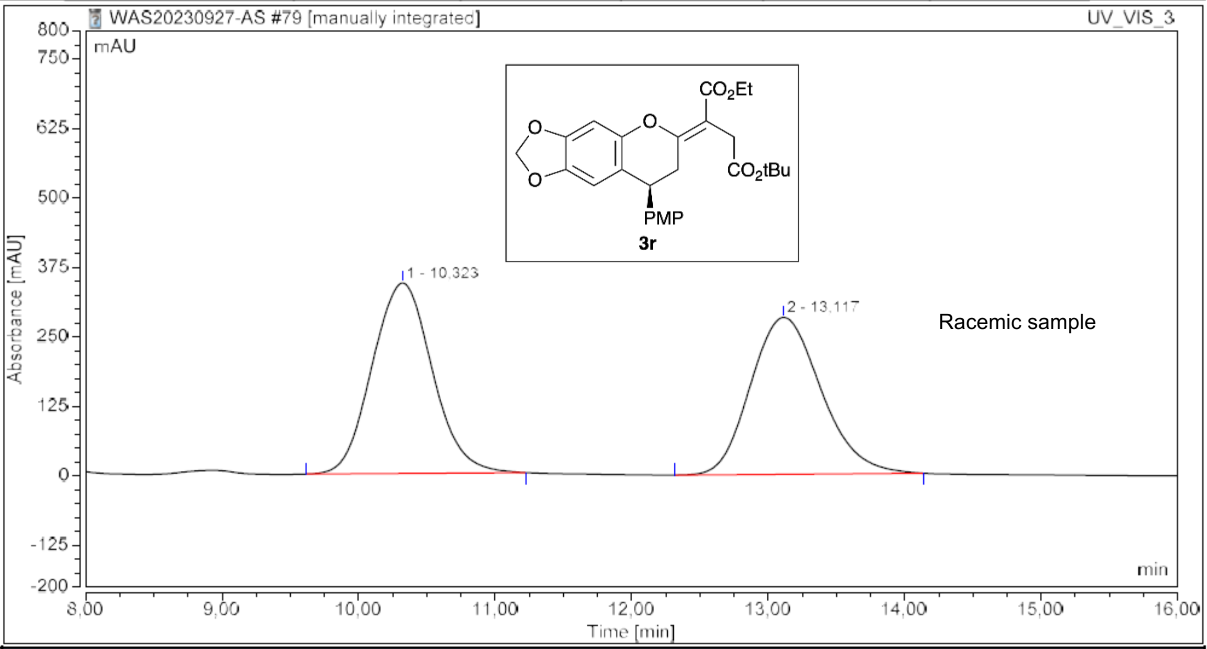

Integration Results

No.	Peak Name	Retention Time min	Area mAU*min	Height mAU	Relative Area %	Relative Height %	Amount n.a.
1		10,323	174,999	342,279	50,87	54,82	n.a.
2		13,117	168,999	282,070	49,13	45,18	n.a.
**Total:**			**343,998**	**624,348**	**100,00**	**100,00**	

Chromatogram

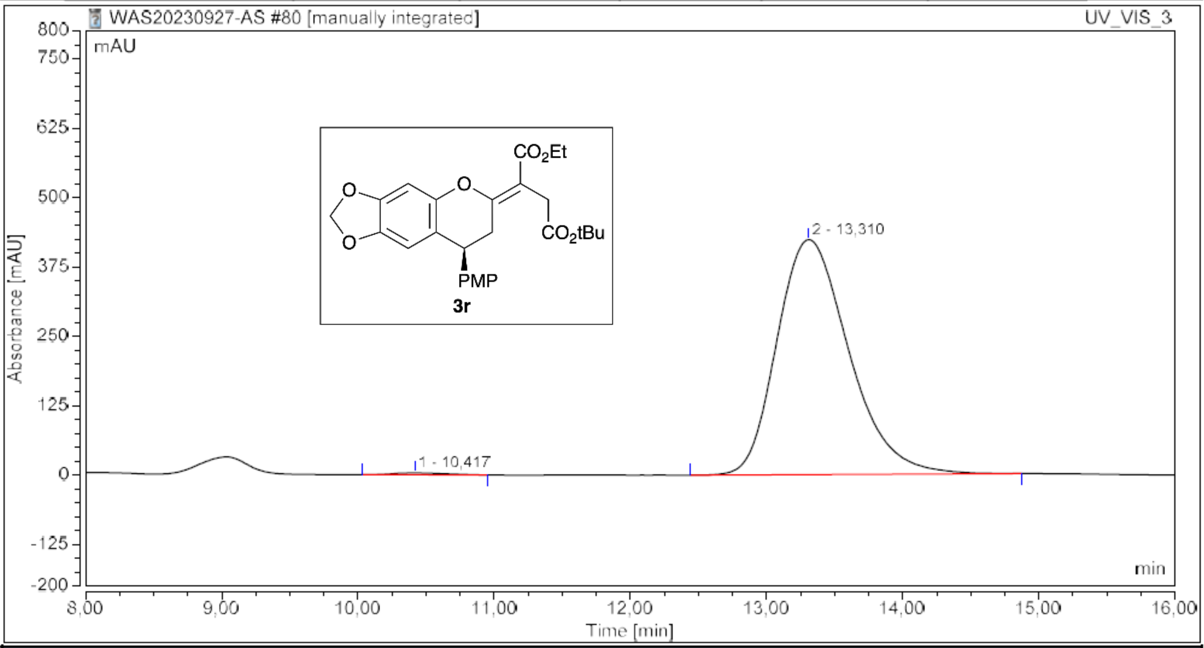

Integration Results

No.	Peak Name	Retention Time min	Area mAU*min	Height mAU	Relative Area %	Relative Height %	Amount n.a.
1		10,417	1,487	3,663	0,57	0,86	n.a.
2		13,310	258,952	423,316	99,43	99,14	n.a.
**Total:**			**260,439**	**426,979**	**100,00**	**100,00**	

Chromatogram

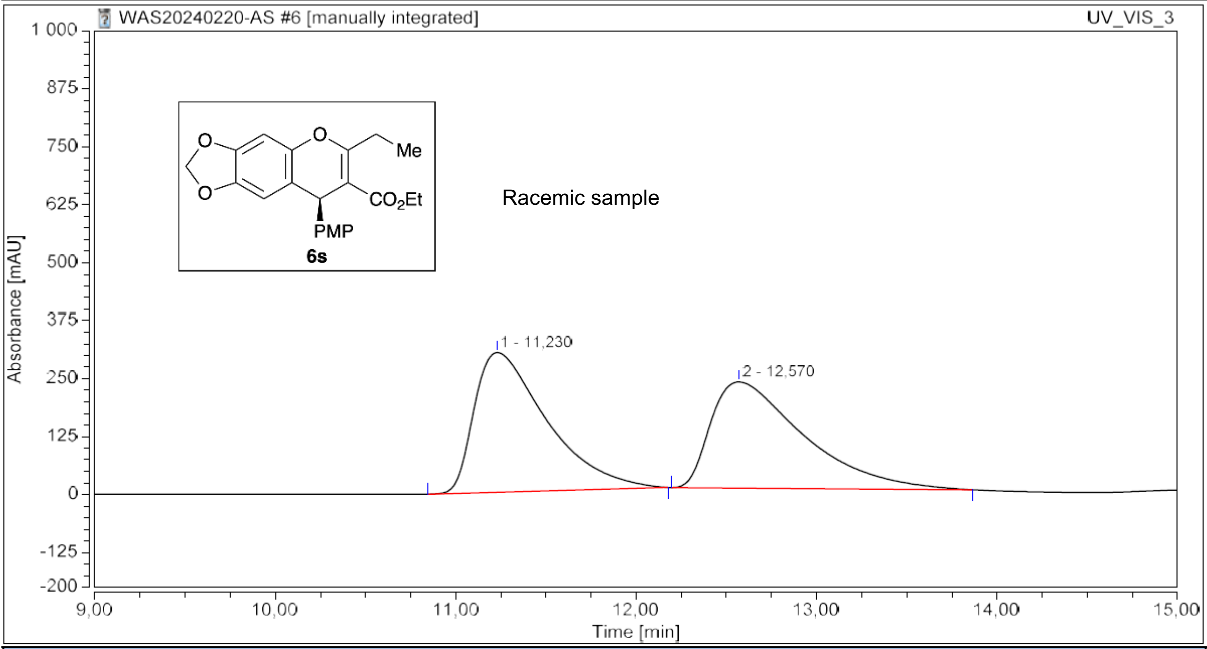

Integration Results

No.	Peak Name	Retention Time<br>min	Area<br>mAU*min	Height<br>mAU	Relative Area<br>%	Relative Height<br>%	Amount<br>n.a.
1		11,230	140,059	301,359	50,74	56,82	n.a.
2		12,570	136,001	229,050	49,26	43,18	n.a.
**Total:**			**276,060**	**530,409**	**100,00**	**100,00**	

Chromatogram

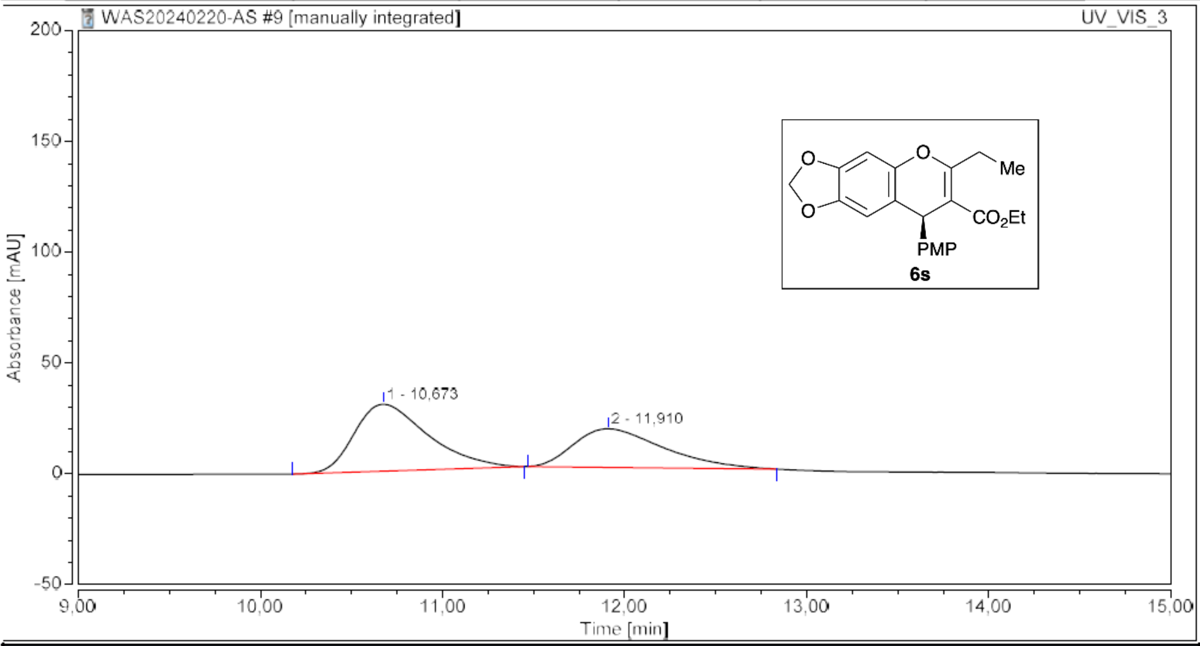

Integration Results

No.	Peak Name	Retention Time<br>min	Area<br>mAU*min	Height<br>mAU	Relative Area<br>%	Relative Height<br>%	Amount<br>n.a.
1		10,673	14,155	30,204	58,69	63,43	n.a.
2		11,910	9,965	17,416	41,31	36,57	n.a.
**Total:**			**24,120**	**47,621**	**100,00**	**100,00**	

Chromatogram

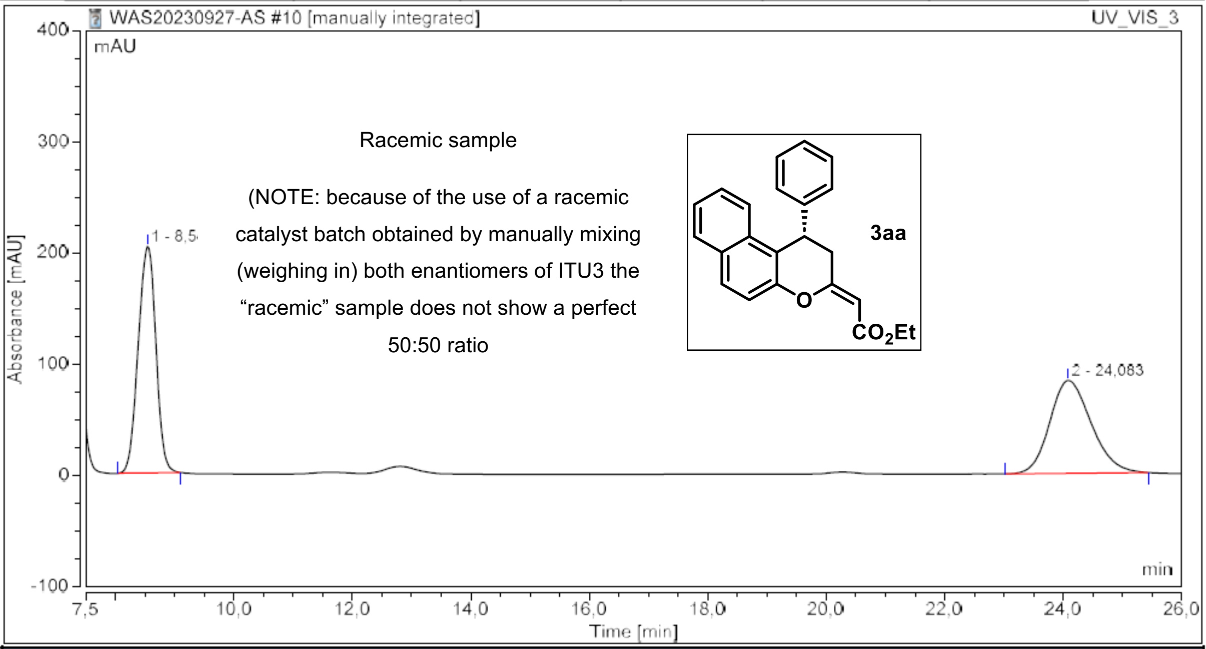

Integration Results

No.	Peak Name	Retention Time min	Area mAU*min	Height mAU	Relative Area %	Relative Height %	Amount n.a.
1		8,547	70,430	203,842	51,06	70,94	n.a.
2		24,083	67,515	83,489	48,94	29,06	n.a.
**Total:**			**137,944**	**287,331**	**100,00**	**100,00**	

Chromatogram

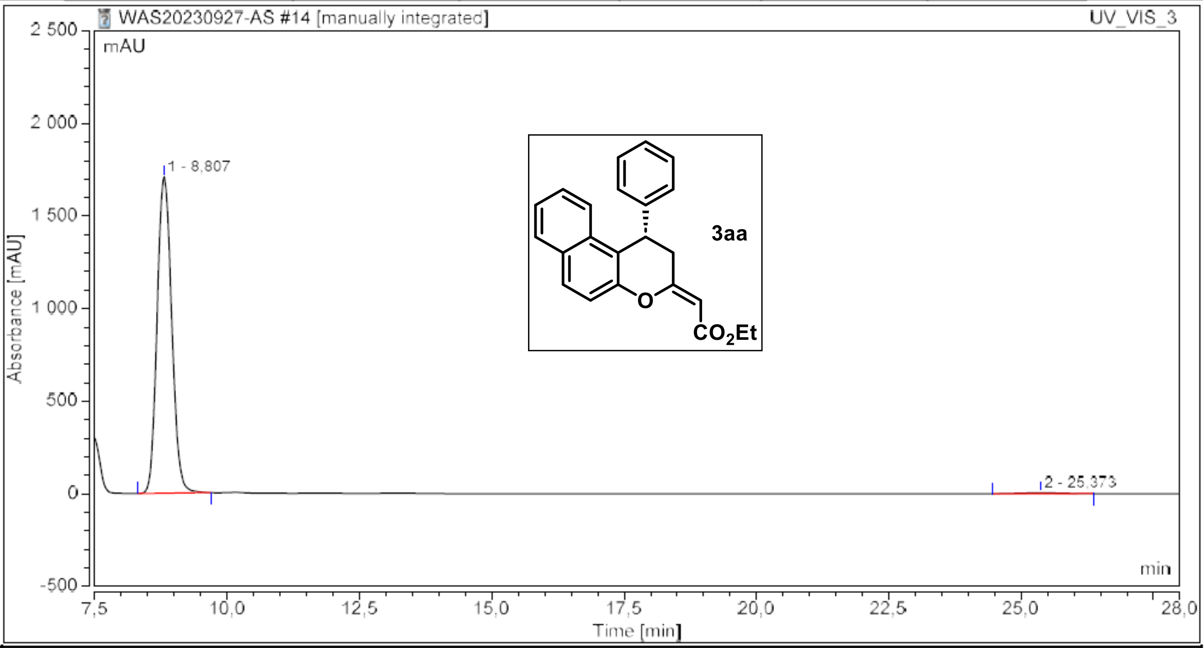

Integration Results

No.	Peak Name	Retention Time min	Area mAU*min	Height mAU	Relative Area %	Relative Height %	Amount n.a.
1		8,807	545,217	1709,461	99,10	99,65	n.a.
2		25,373	4,951	6,031	0,90	0,35	n.a.
**Total:**			**550,168**	**1715,492**	**100,00**	**100,00**	

Chromatogram

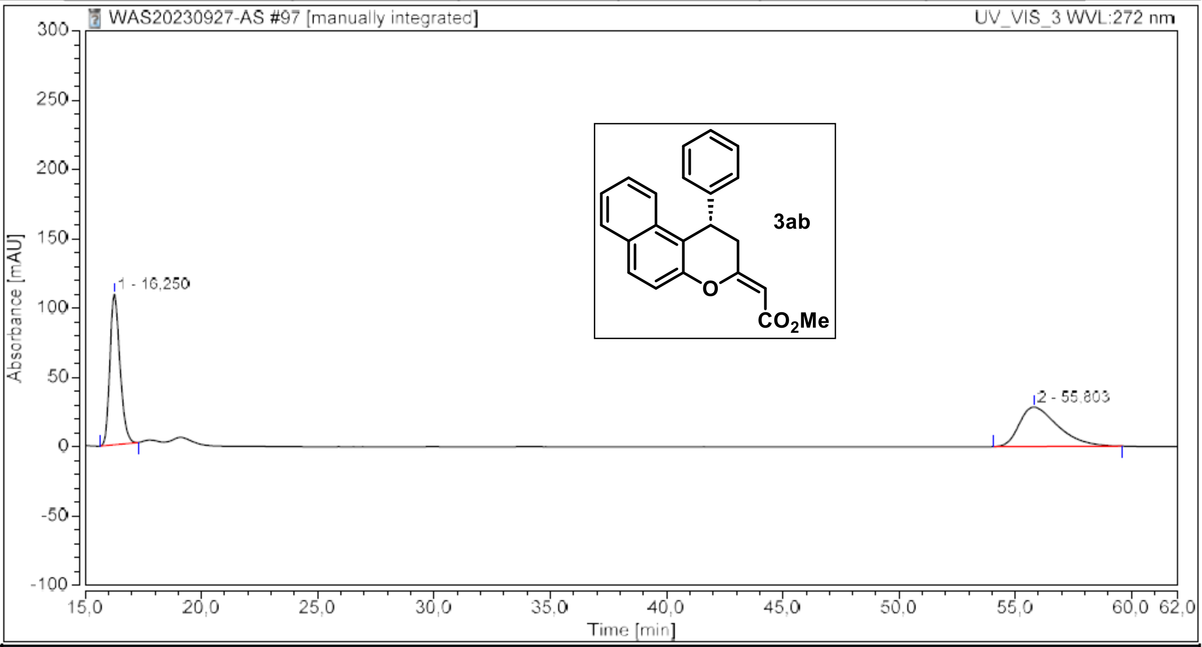

Integration Results

No.	Peak Name	Retention Time min	Area mAU*min	Height mAU	Relative Area %	Relative Height %	Amount n.a.
1		16,250	54,590	108,533	49,78	79,20	n.a.
2		55,803	55,081	28,499	50,22	20,80	n.a.
**Total:**			**109,670**	**137,032**	**100,00**	**100,00**	

Chromatogram

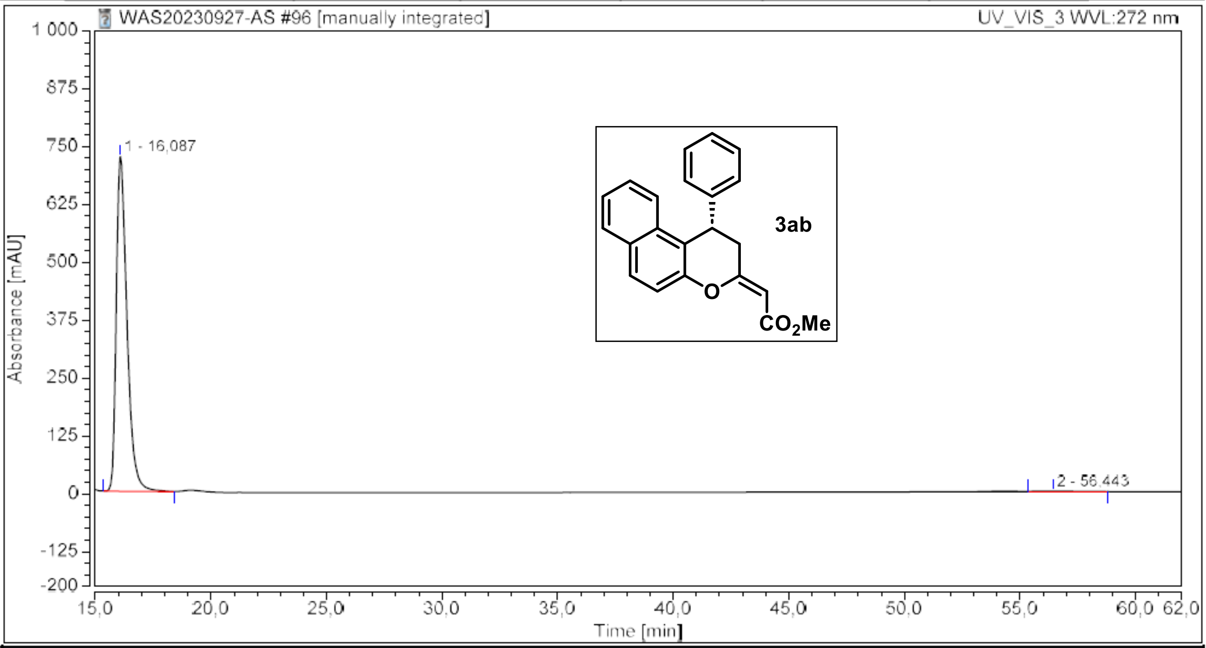

Integration Results

No.	Peak Name	Retention Time min	Area mAU*min	Height mAU	Relative Area %	Relative Height %	Amount n.a.
1		16,087	387,616	722,906	99,40	99,83	n.a.
2		56,443	2,330	1,225	0,60	0,17	n.a.
**Total:**			**389,946**	**724,131**	**100,00**	**100,00**	

Chromatogram

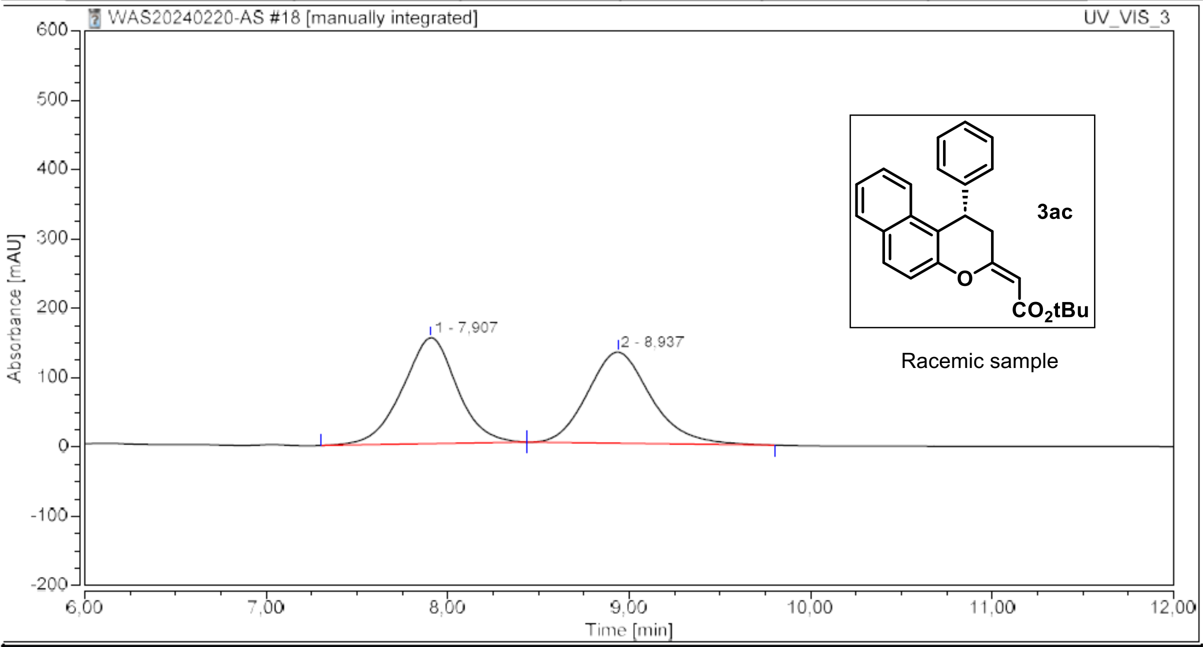

Integration Results

No.	Peak Name	Retention Time<br>min	Area<br>mAU*min	Height<br>mAU	Relative Area<br>%	Relative Height<br>%	Amount<br>n.a.
1		7,907	53,292	152,691	50,10	53,76	n.a.
2		8,937	53,083	131,346	49,90	46,24	n.a.
**Total:**			**106,376**	**284,037**	**100,00**	**100,00**	

Chromatogram

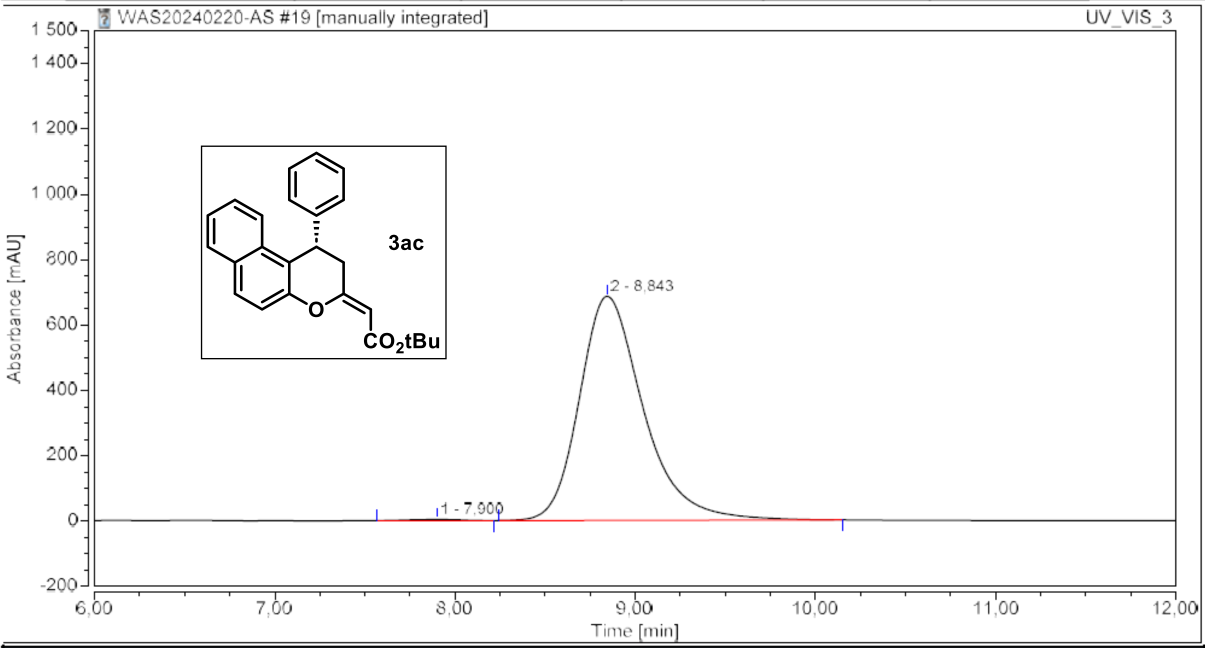

Integration Results

No.	Peak Name	Retention Time<br>min	Area<br>mAU*min	Height<br>mAU	Relative Area<br>%	Relative Height<br>%	Amount<br>n.a.
1		7,900	1,173	4,004	0,41	0,58	n.a.
2		8,843	282,186	685,976	99,59	99,42	n.a.
**Total:**			**283,359**	**689,980**	**100,00**	**100,00**	

Chromatogram

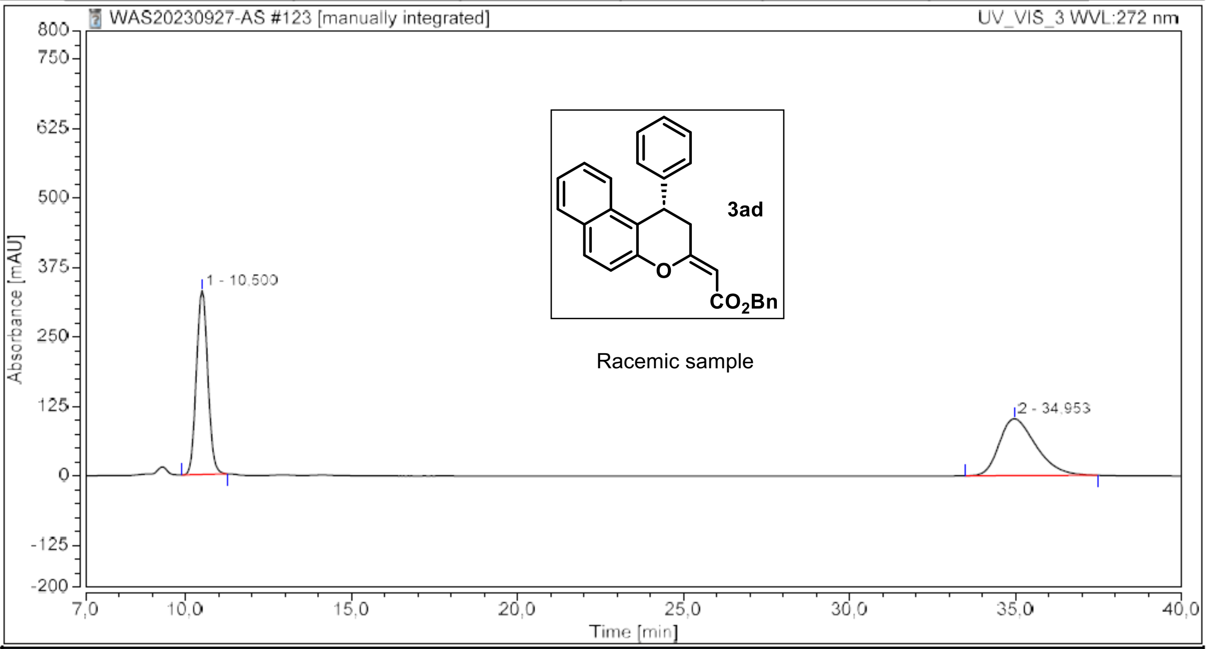

Integration Results

No.	Peak Name	Retention Time min	Area mAU*min	Height mAU	Relative Area %	Relative Height %	Amount n.a.
1		10,500	131,149	330,806	49,96	76,37	n.a.
2		34,953	131,338	102,357	50,04	23,63	n.a.
**Total:**			**262,487**	**433,163**	**100,00**	**100,00**	

Chromatogram

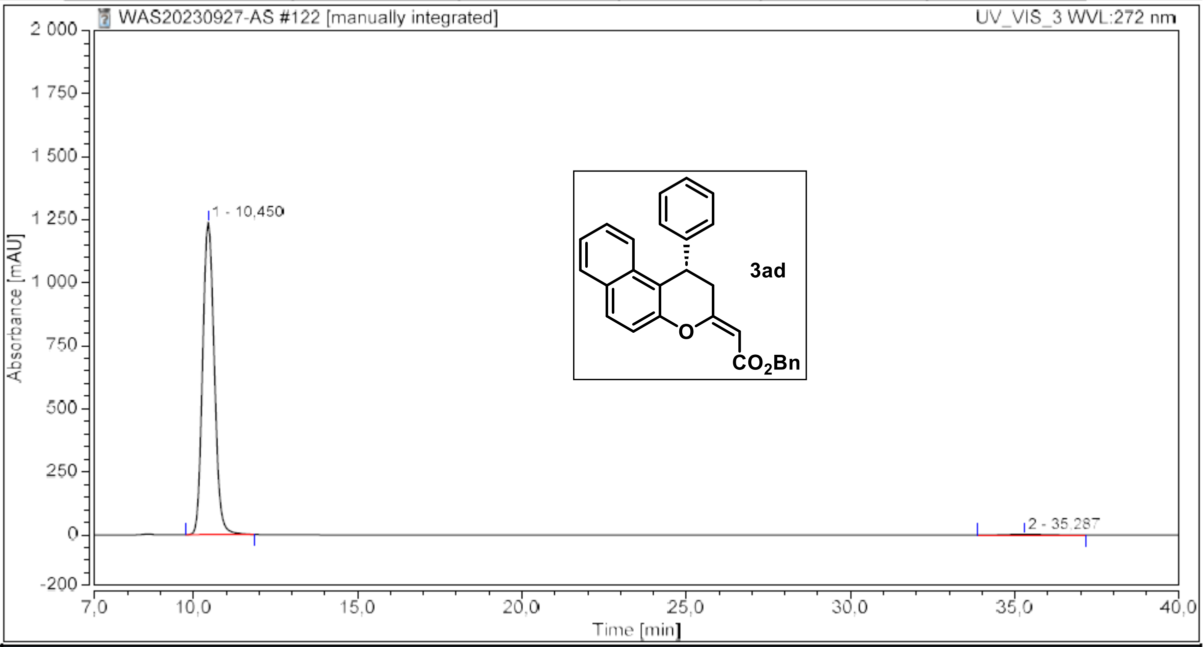

Integration Results

No.	Peak Name	Retention Time min	Area mAU*min	Height mAU	Relative Area %	Relative Height %	Amount n.a.
1		10,450	507,243	1239,995	99,38	99,80	n.a.
2		35,287	3,154	2,457	0,62	0,20	n.a.
**Total:**			**510,397**	**1242,453**	**100,00**	**100,00**	

Chromatogram

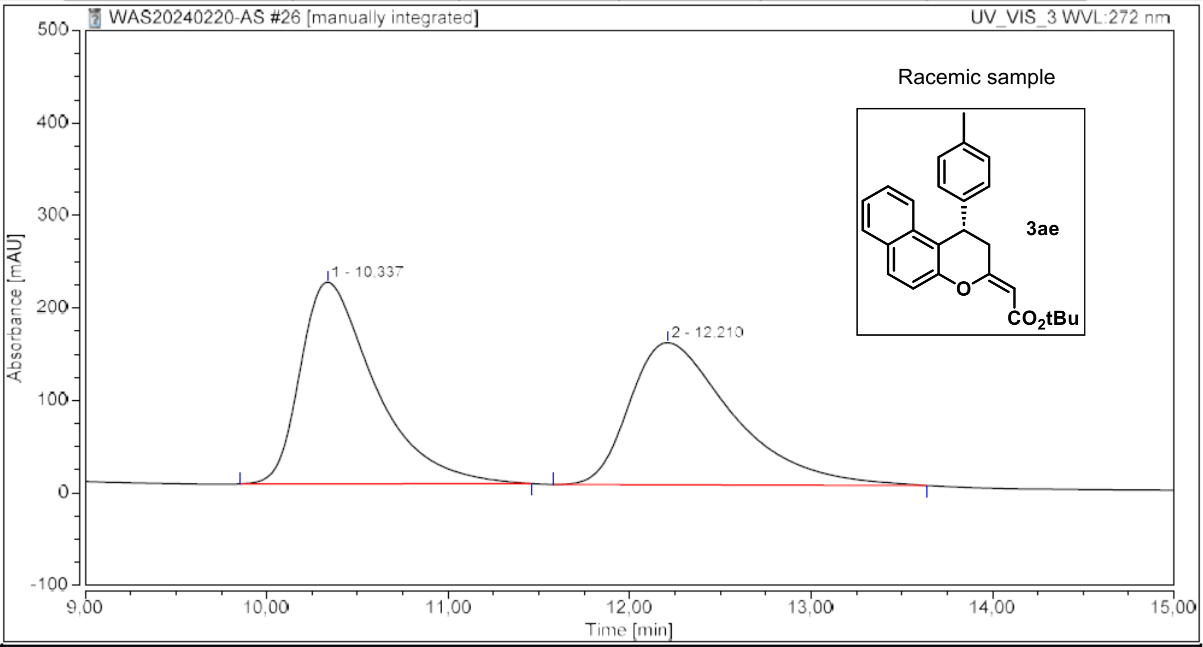

Integration Results

No.	Peak Name	Retention Time min	Area mAU*min	Height mAU	Relative Area %	Relative Height %	Amount n.a.
1		10,337	103,990	218,079	50,65	58,66	n.a.
2		12,210	101,301	153,717	49,35	41,34	n.a.
**Total:**			**205,291**	**371,797**	**100,00**	**100,00**	

Chromatogram

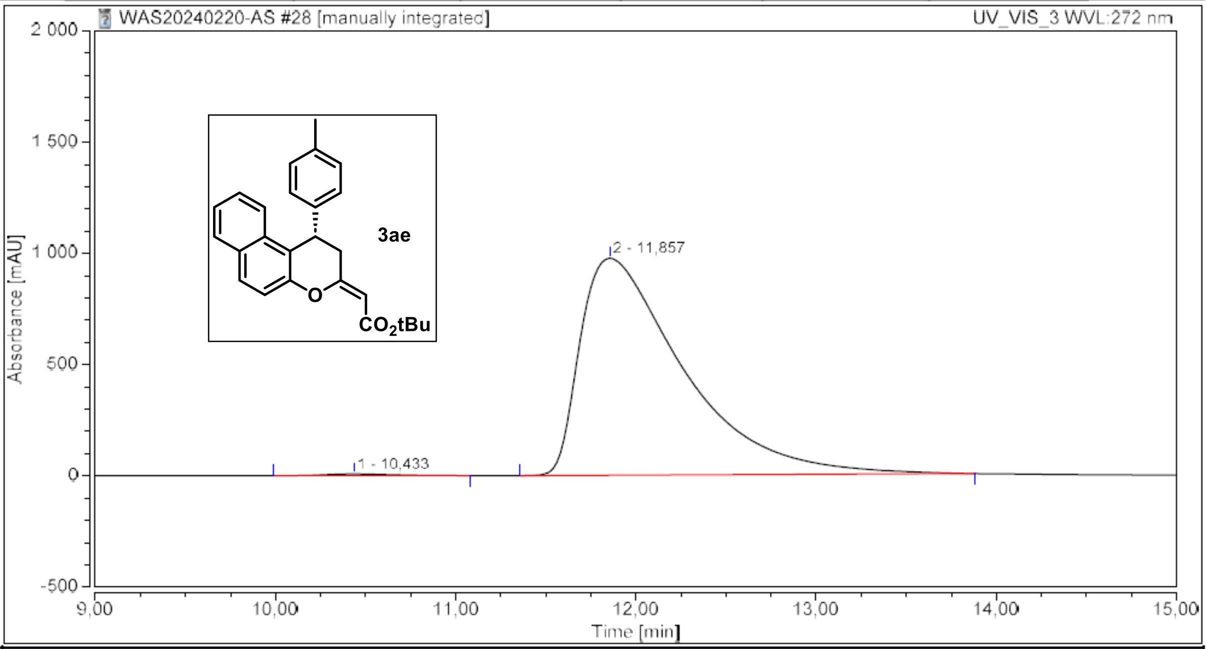

Integration Results

No.	Peak Name	Retention Time min	Area mAU*min	Height mAU	Relative Area %	Relative Height %	Amount n.a.
1		10,433	2,920	6,857	0,45	0,70	n.a.
2		11,857	648,013	974,889	99,55	99,30	n.a.
**Total:**			**650,933**	**981,746**	**100,00**	**100,00**	

Chromatogram

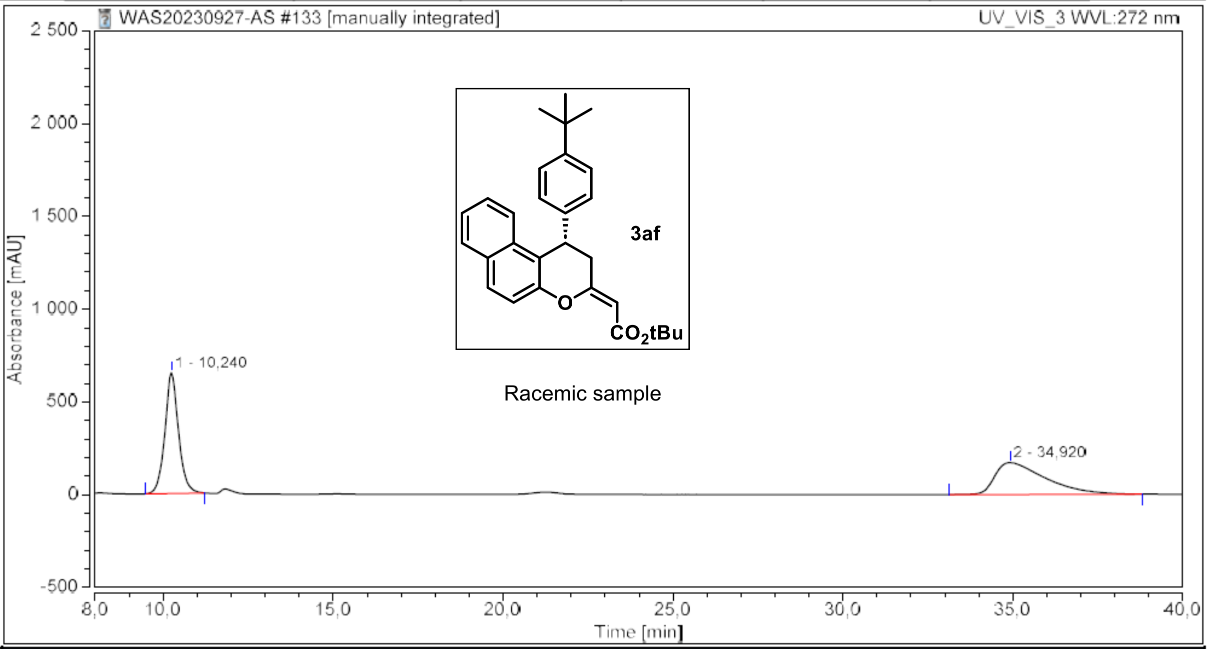

Racemic sample

Integration Results

No.	Peak Name	Retention Time min	Area mAU*min	Height mAU	Relative Area %	Relative Height %	Amount n.a.
1		10,240	301,967	651,055	50,58	79,13	n.a.
2		34,920	295,051	171,680	49,42	20,87	n.a.
**Total:**			**597,018**	**822,735**	**100,00**	**100,00**	

Chromatogram

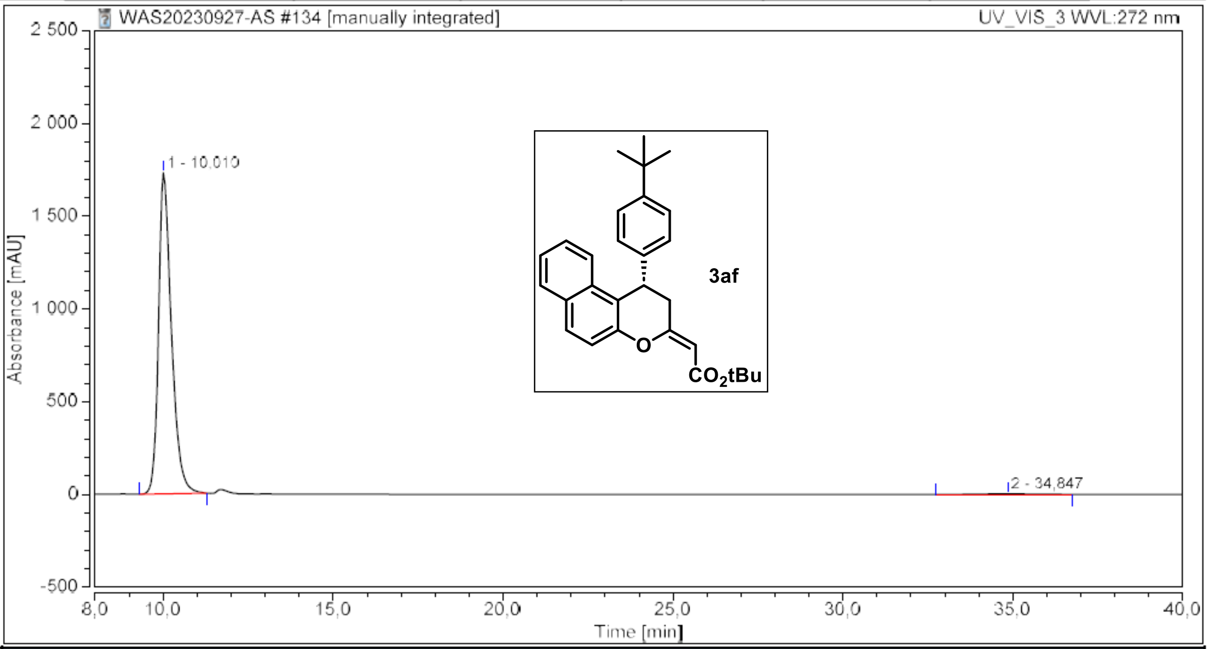

Integration Results

No.	Peak Name	Retention Time min	Area mAU*min	Height mAU	Relative Area %	Relative Height %	Amount n.a.
1		10,010	762,666	1731,771	99,50	99,86	n.a.
2		34,847	3,856	2,496	0,50	0,14	n.a.
**Total:**			**766,523**	**1734,267**	**100,00**	**100,00**	

Chromatogram

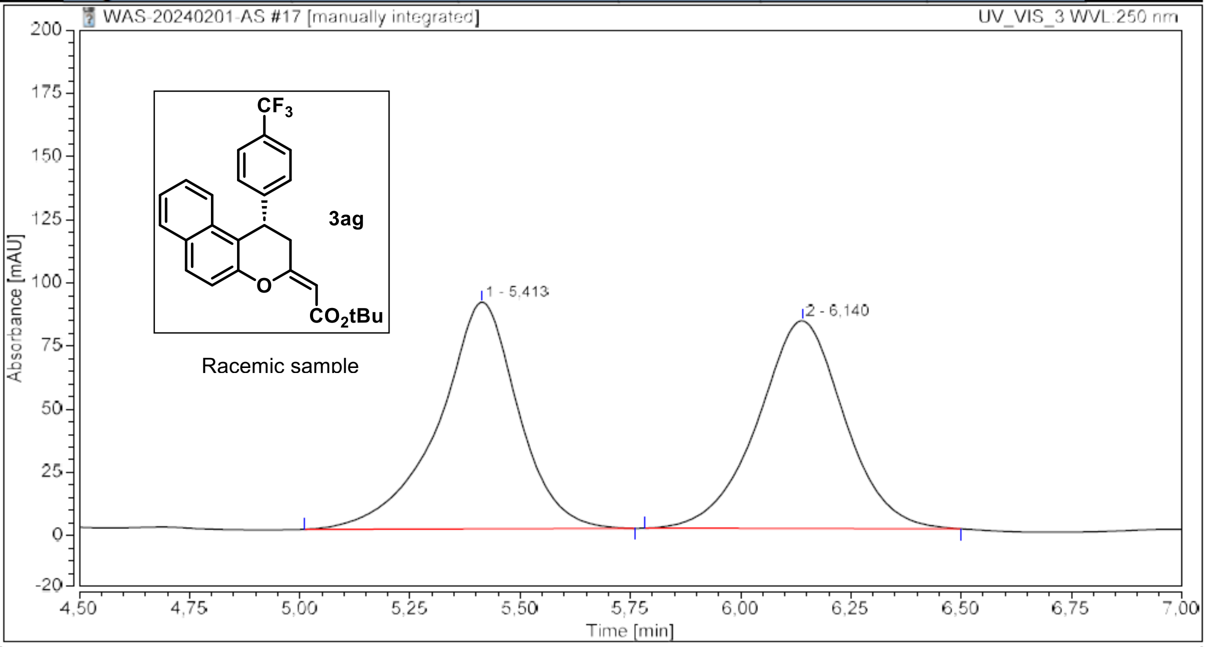

Integration Results

No.	Peak Name	Retention Time min	Area mAU*min	Height mAU	Relative Area %	Relative Height %	Amount n.a.
1		5,413	18,811	89,753	50,08	52,18	n.a.
2		6,140	18,754	82,244	49,92	47,82	n.a.
**Total:**			**37,565**	**171,997**	**100,00**	**100,00**	

Chromatogram

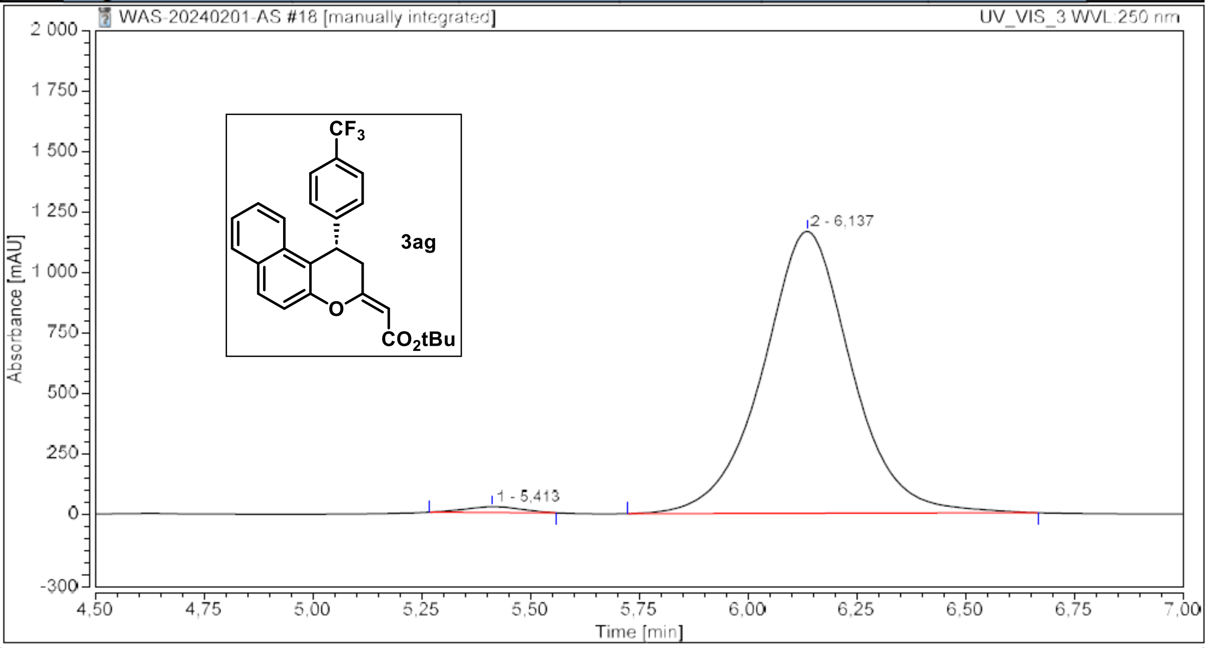

Integration Results

No.	Peak Name	Retention Time min	Area mAU*min	Height mAU	Relative Area %	Relative Height %	Amount n.a.
1		5,413	3,517	23,846	1,26	2,01	n.a.
2		6,137	276,100	1165,397	98,74	97,99	n.a.
**Total:**			**279,617**	**1189,243**	**100,00**	**100,00**	

Chromatogram

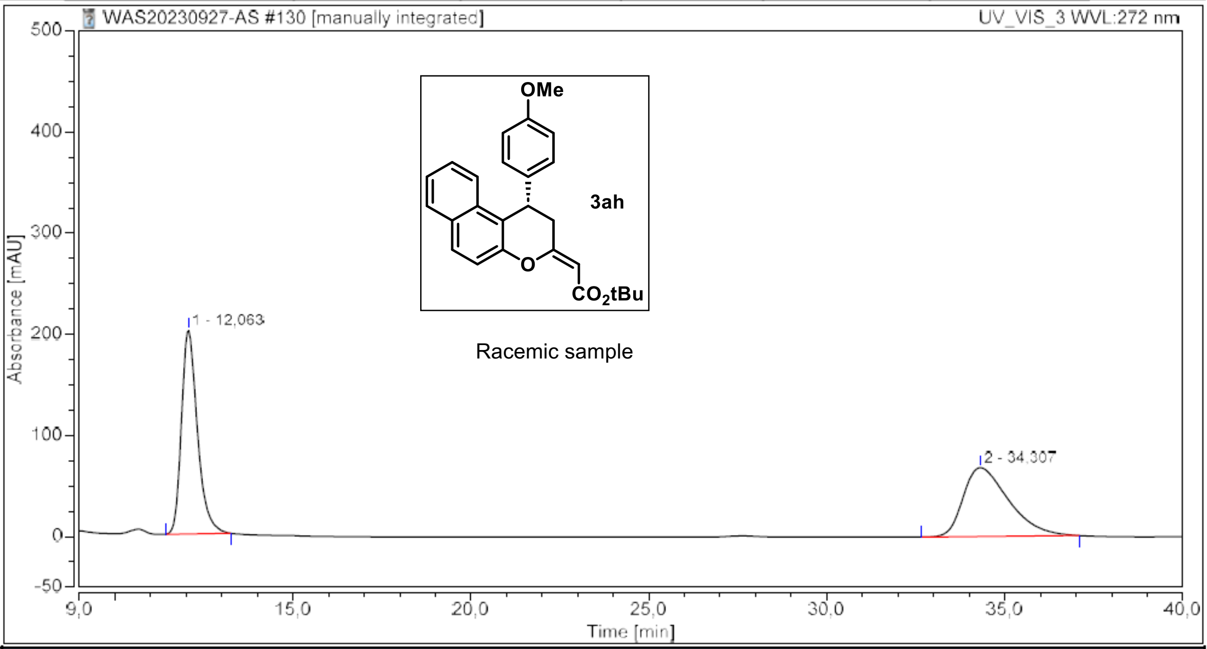

Integration Results

No.	Peak Name	Retention Time min	Area mAU*min	Height mAU	Relative Area %	Relative Height %	Amount n.a.
1		12,063	104,579	201,563	50,93	74,77	n.a.
2		34,307	100,741	68,016	49,07	25,23	n.a.
**Total:**			**205,320**	**269,579**	**100,00**	**100,00**	

Chromatogram

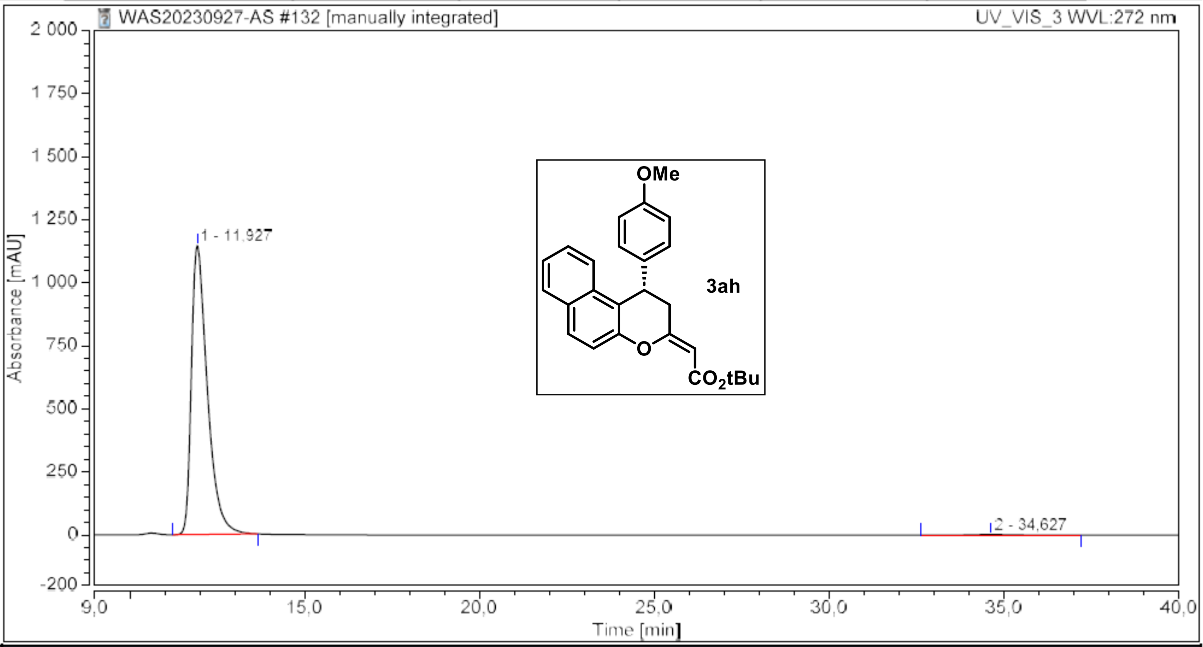

Integration Results

No.	Peak Name	Retention Time min	Area mAU*min	Height mAU	Relative Area %	Relative Height %	Amount n.a.
1		11,927	605,156	1145,262	99,46	99,81	n.a.
2		34,627	3,294	2,183	0,54	0,19	n.a.
**Total:**			**608,450**	**1147,445**	**100,00**	**100,00**	

Chromatogram

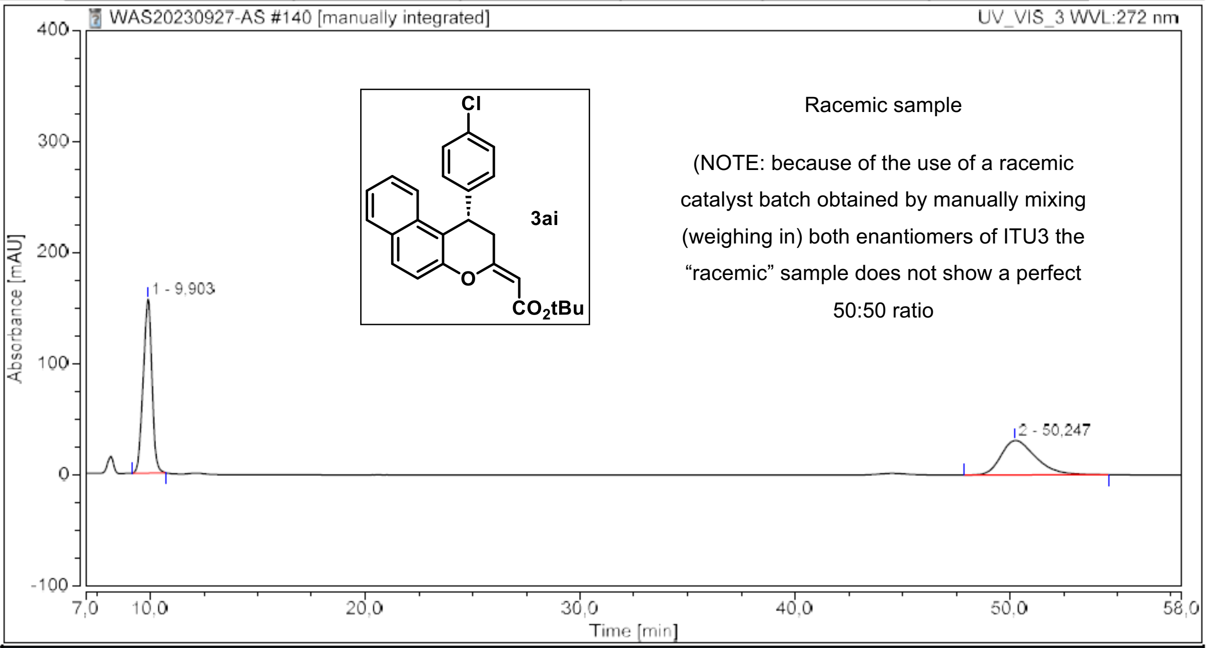

Integration Results

No.	Peak Name	Retention Time<br>min	Area<br>mAU*min	Height<br>mAU	Relative Area<br>%	Relative Height<br>%	Amount<br>n.a.
1		9,903	73,972	156,690	55,96	83,50	n.a.
2		50,247	58,215	30,973	44,04	16,50	n.a.
**Total:**			**132,187**	**187,663**	**100,00**	**100,00**	

Chromatogram

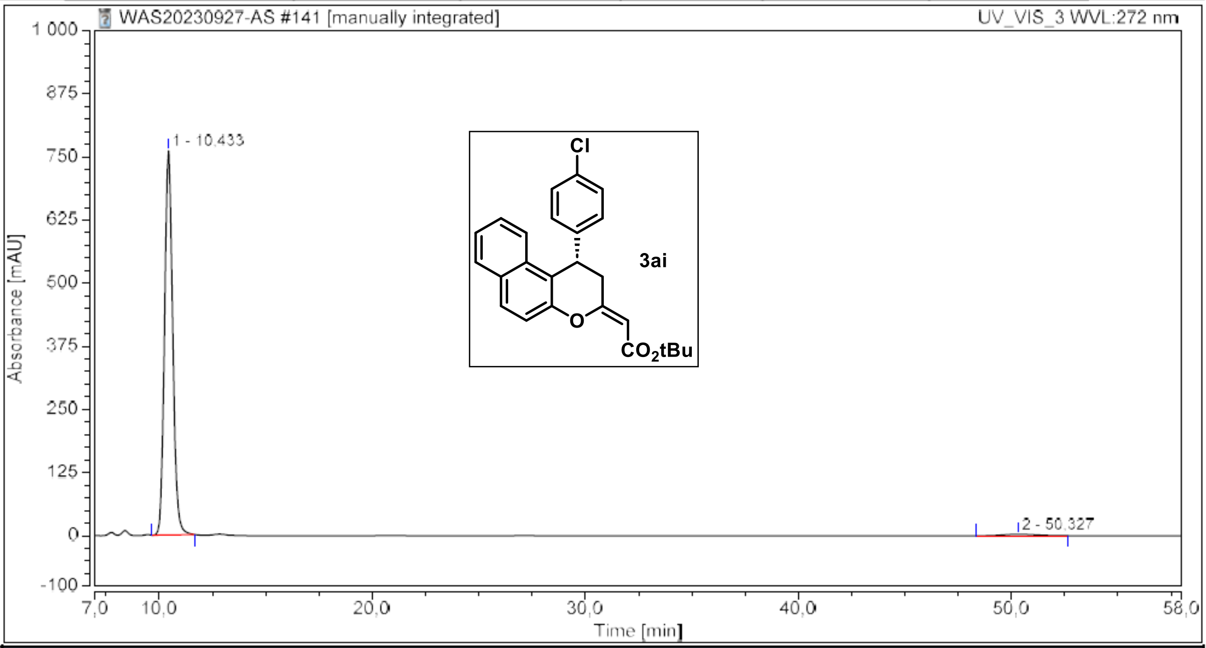

Integration Results

No.	Peak Name	Retention Time<br>min	Area<br>mAU*min	Height<br>mAU	Relative Area<br>%	Relative Height<br>%	Amount<br>n.a.
1		10,433	337,413	760,969	98,59	99,64	n.a.
2		50,327	4,832	2,735	1,41	0,36	n.a.
**Total:**			**342,246**	**763,704**	**100,00**	**100,00**	

Chromatogram

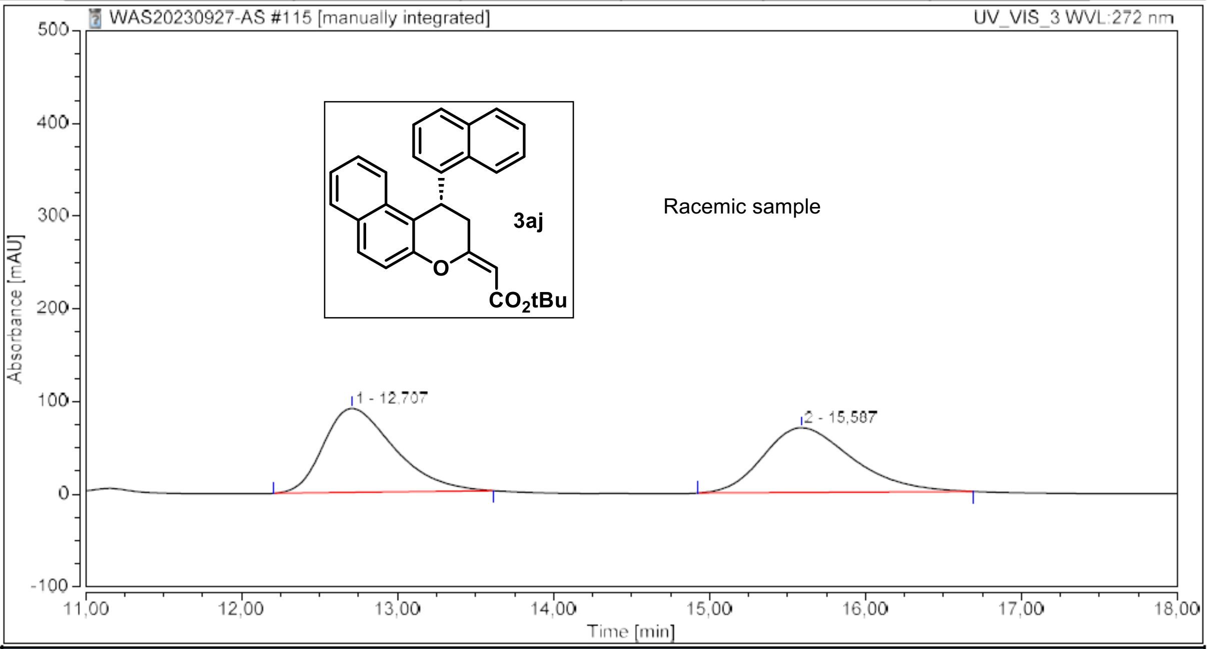

Integration Results

No.	Peak Name	Retention Time<br>min	Area<br>mAU*min	Height<br>mAU	Relative Area<br>%	Relative Height<br>%	Amount<br>n.a.
1		12,707	47,602	90,574	50,75	56,52	n.a.
2		15,587	46,202	69,688	49,25	43,48	n.a.
**Total:**			**93,804**	**160,262**	**100,00**	**100,00**	

Chromatogram

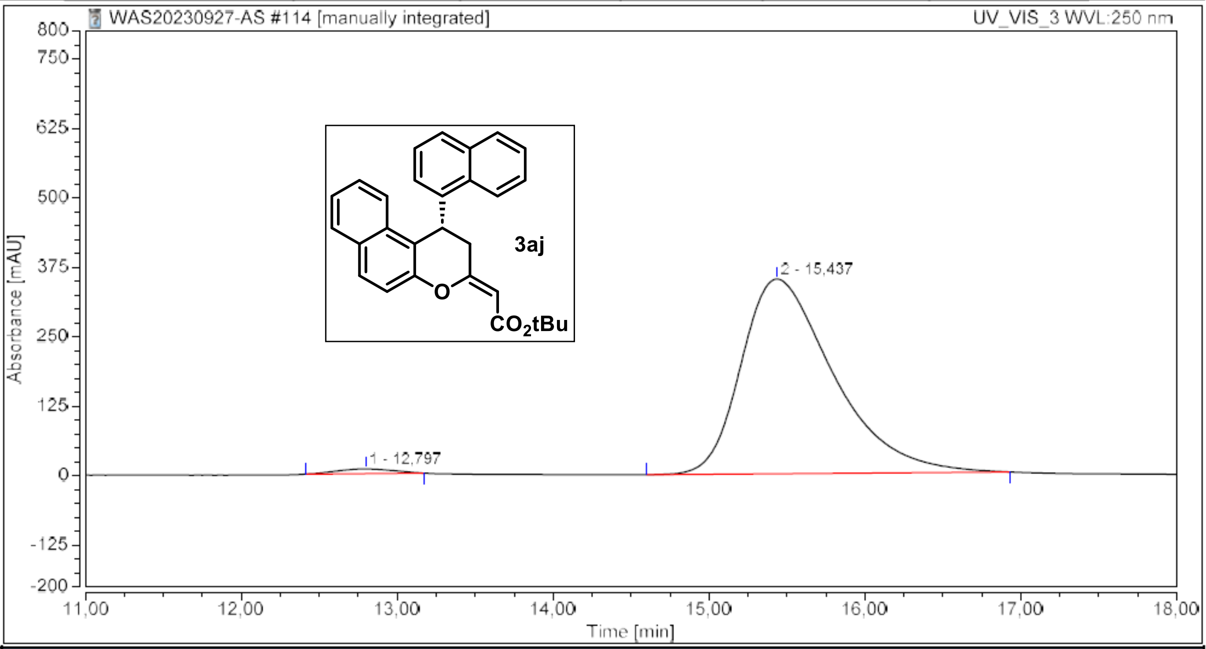

Integration Results

No.	Peak Name	Retention Time<br>min	Area<br>mAU*min	Height<br>mAU	Relative Area<br>%	Relative Height<br>%	Amount<br>n.a.
1		12,797	3,537	8,414	1,45	2,35	n.a.
2		15,437	240,135	350,064	98,55	97,65	n.a.
**Total:**			**243,672**	**358,478**	**100,00**	**100,00**	

Chromatogram

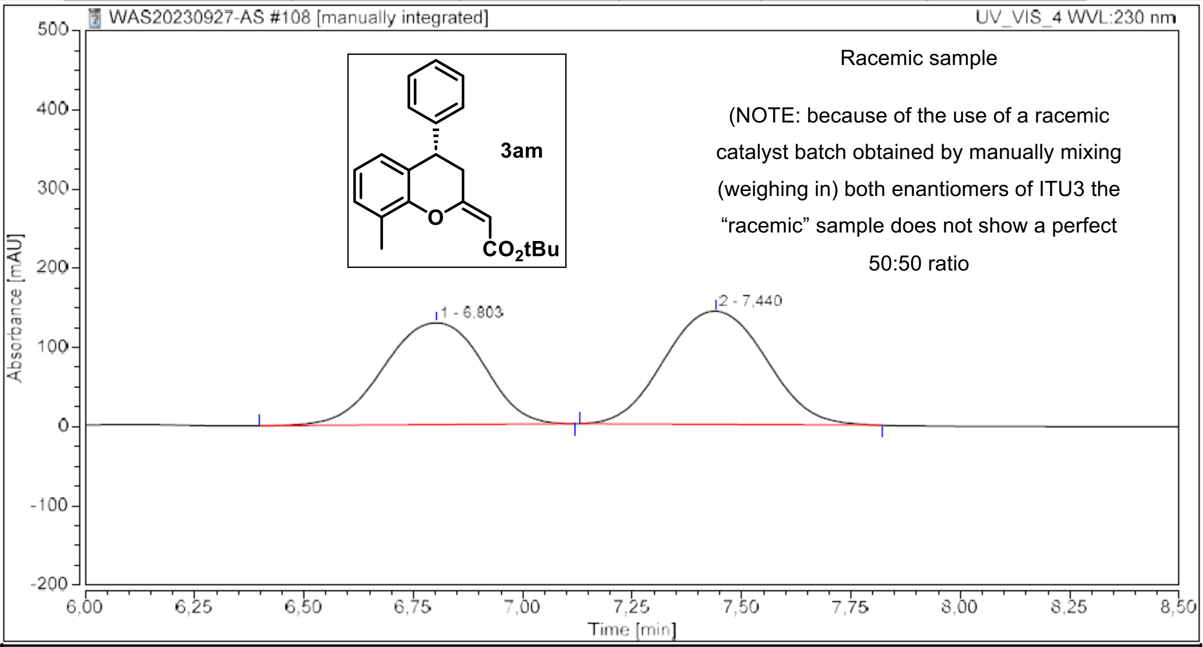

Integration Results

No.	Peak Name	Retention Time min	Area mAU*min	Height mAU	Relative Area %	Relative Height %	Amount n.a.
1		6,803	33,612	128,500	46,78	47,36	n.a.
2		7,440	38,240	142,852	53,22	52,64	n.a.
**Total:**			**71,851**	**271,352**	**100,00**	**100,00**	

Chromatogram

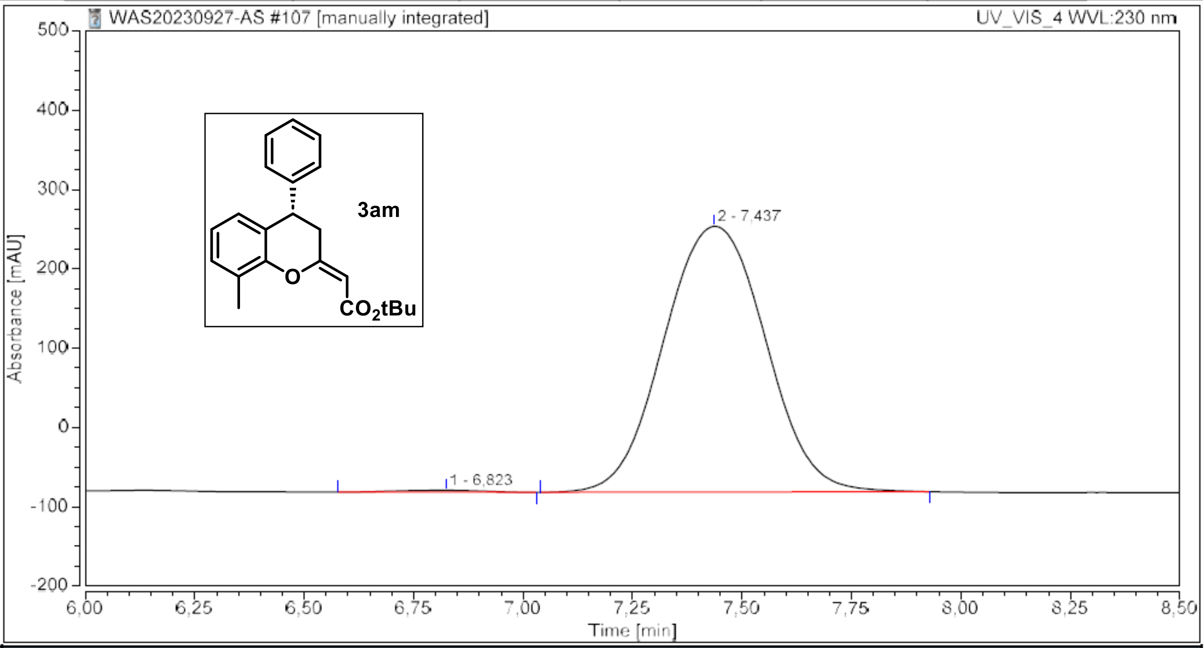

Integration Results

No.	Peak Name	Retention Time min	Area mAU*min	Height mAU	Relative Area %	Relative Height %	Amount n.a.
1		6,823	0,521	2,247	0,57	0,67	n.a.
2		7,437	90,141	335,096	99,43	99,33	n.a.
**Total:**			**90,661**	**337,343**	**100,00**	**100,00**	

Chromatogram

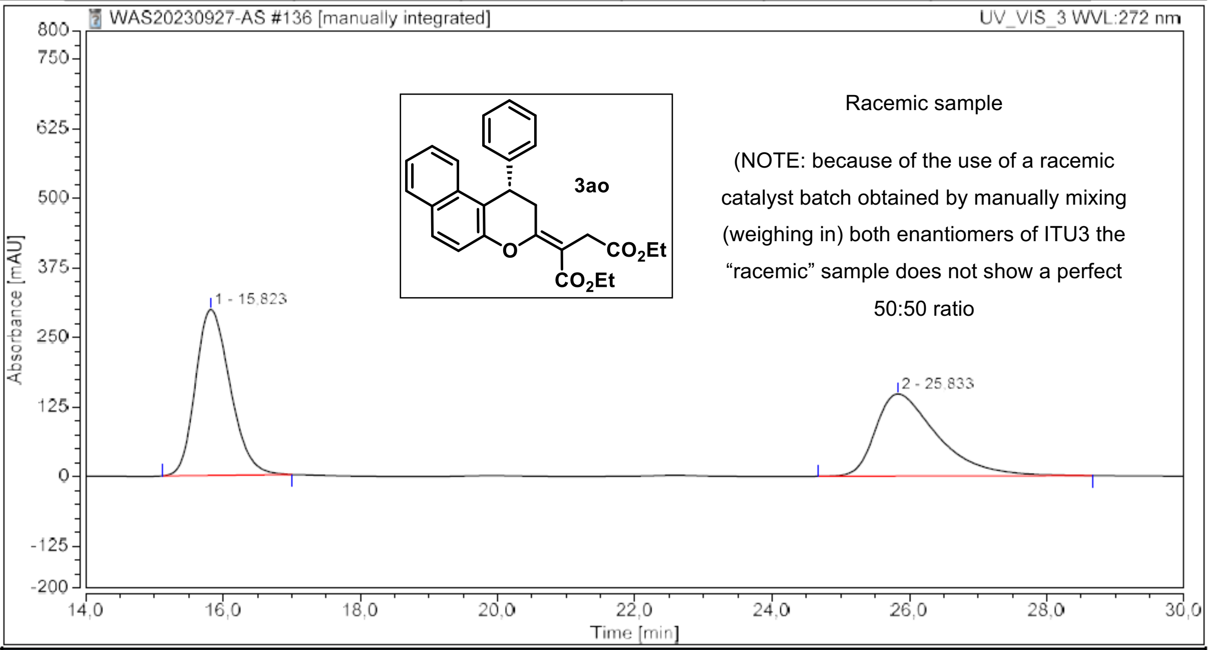

Integration Results

No.	Peak Name	Retention Time<br>min	Area<br>mAU*min	Height<br>mAU	Relative Area<br>%	Relative Height<br>%	Amount<br>n.a.
1		15,823	174,334	298,016	53,09	66,89	n.a.
2		25,833	154,011	147,512	46,91	33,11	n.a.
**Total:**			**328,345**	**445,527**	**100,00**	**100,00**	

Chromatogram

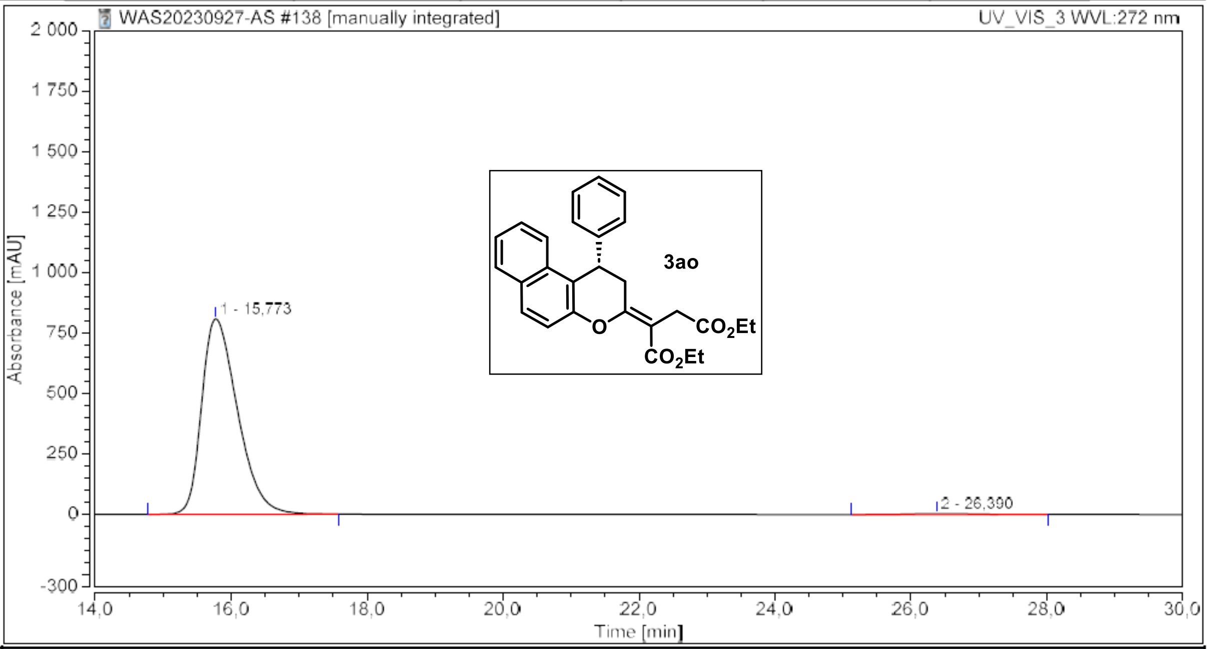

Integration Results

No.	Peak Name	Retention Time<br>min	Area<br>mAU*min	Height<br>mAU	Relative Area<br>%	Relative Height<br>%	Amount<br>n.a.
1		15,773	494,764	808,960	99,26	99,57	n.a.
2		26,390	3,692	3,453	0,74	0,43	n.a.
**Total:**			**498,456**	**812,413**	**100,00**	**100,00**	

Chromatogram

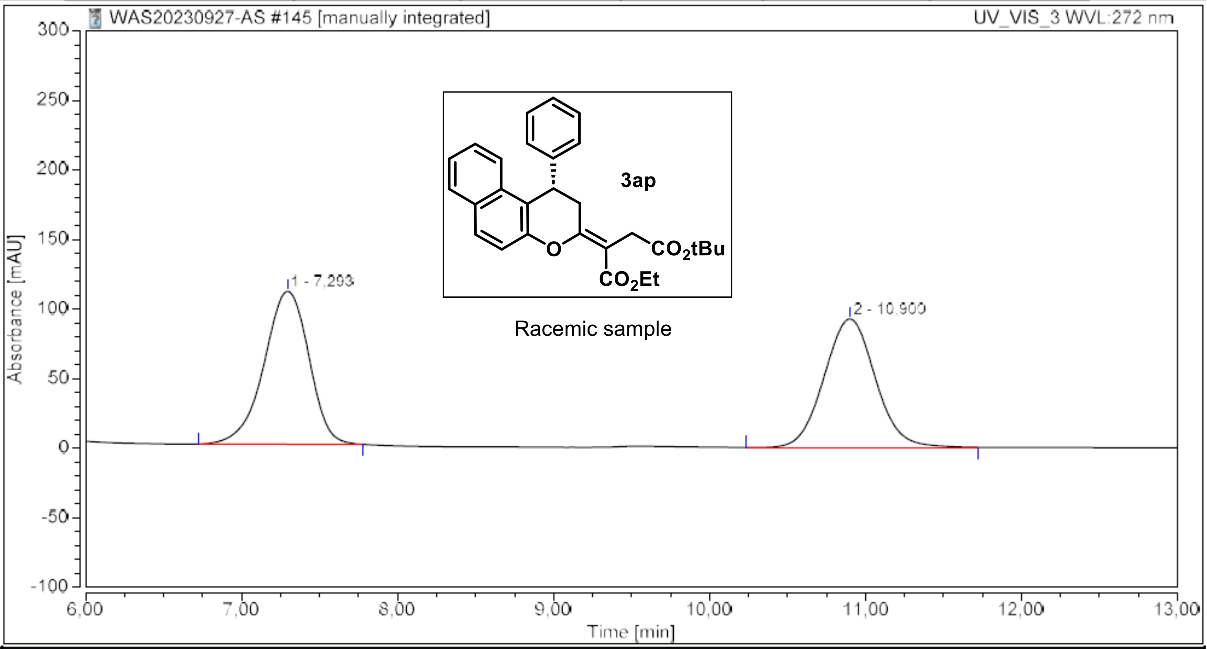

Integration Results

No.	Peak Name	Retention Time min	Area mAU*min	Height mAU	Relative Area %	Relative Height %	Amount n.a.
1		7,293	36,677	109,887	51,19	54,35	n.a.
2		10,900	34,969	92,282	48,81	45,65	n.a.
**Total:**			**71,646**	**202,169**	**100,00**	**100,00**	

Chromatogram

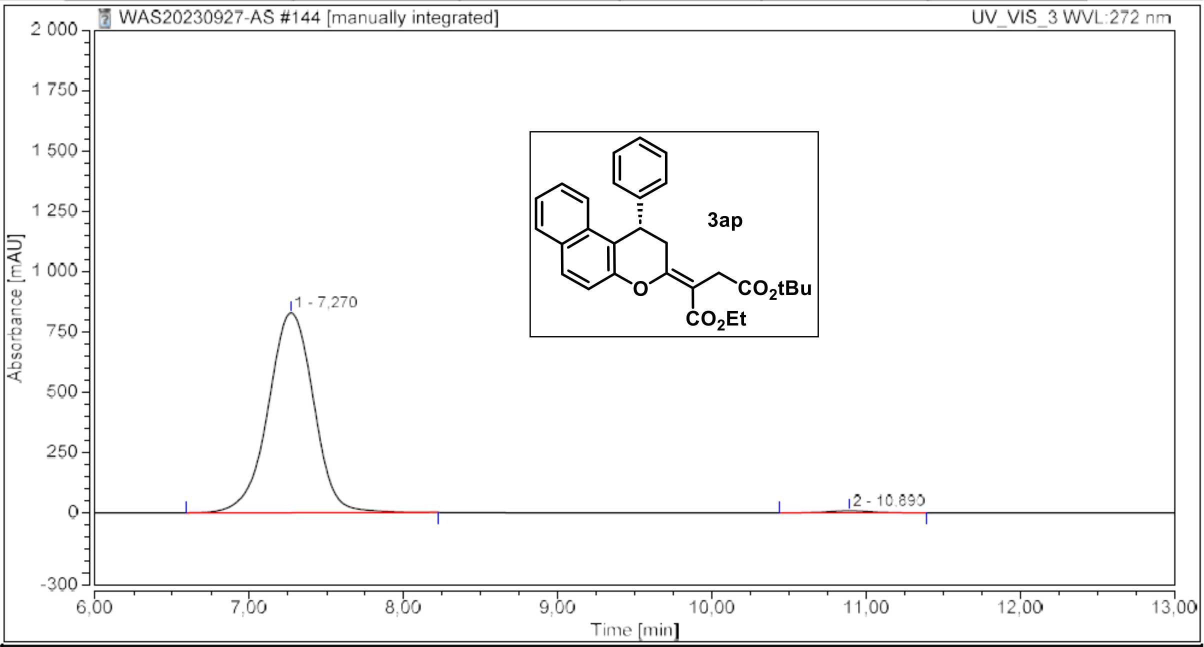

Integration Results

No.	Peak Name	Retention Time min	Area mAU*min	Height mAU	Relative Area %	Relative Height %	Amount n.a.
1		7,270	283,311	828,775	98,88	98,92	n.a.
2		10,890	3,223	9,031	1,12	1,08	n.a.
**Total:**			**286,534**	**837,807**	**100,00**	**100,00**	

Chromatogram

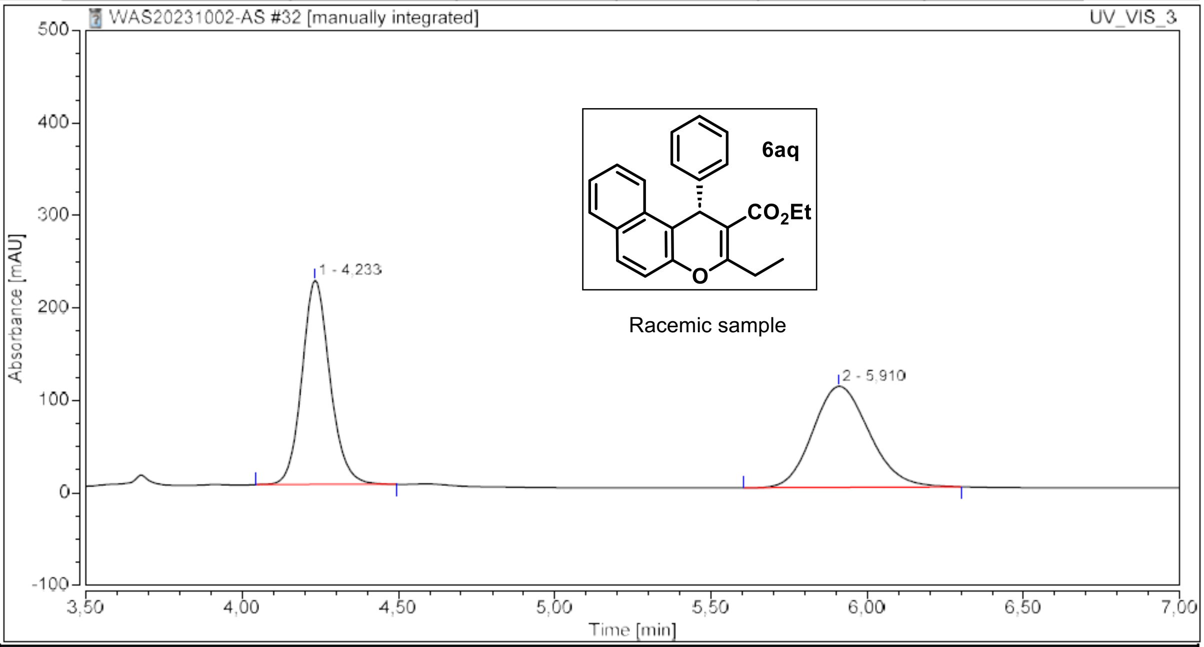

Integration Results

No.	Peak Name	Retention Time<br>min	Area<br>mAU*min	Height<br>mAU	Relative Area<br>%	Relative Height<br>%	Amount<br>n.a.
1		4,233	22,956	220,839	50,05	66,90	n.a.
2		5,910	22,907	109,274	49,95	33,10	n.a.
**Total:**			**45,863**	**330,113**	**100,00**	**100,00**	

Chromatogram

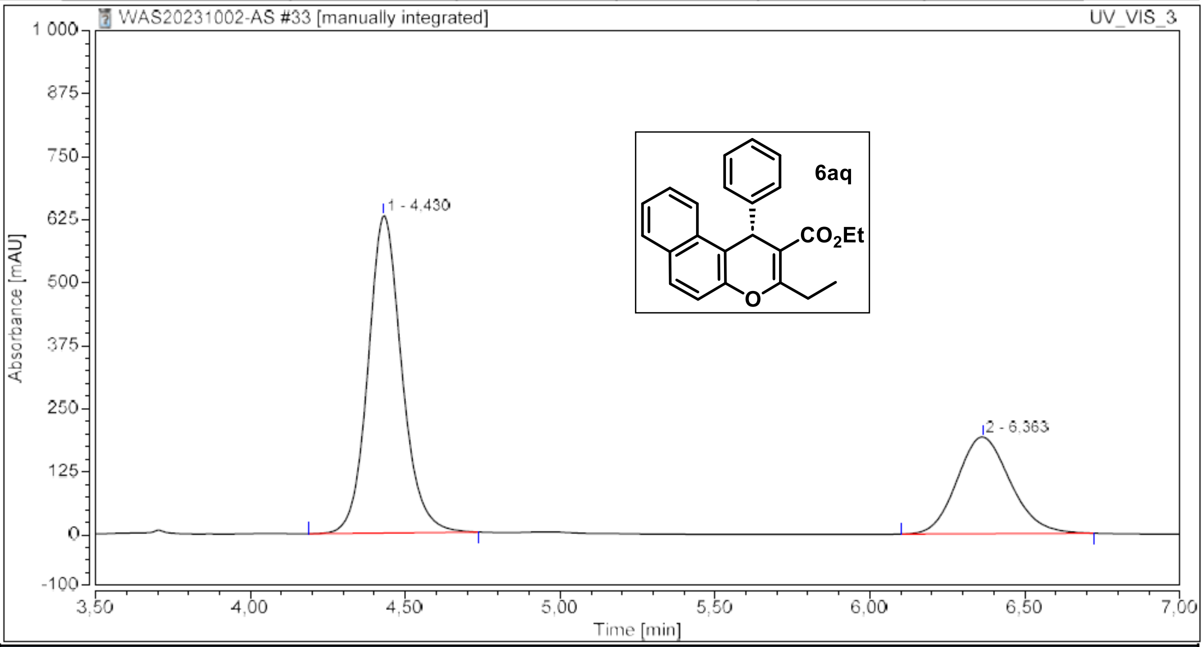

Integration Results

No.	Peak Name	Retention Time<br>min	Area<br>mAU*min	Height<br>mAU	Relative Area<br>%	Relative Height<br>%	Amount<br>n.a.
1		4,430	79,437	629,477	67,44	76,63	n.a.
2		6,363	38,348	192,014	32,56	23,37	n.a.
**Total:**			**117,785**	**821,491**	**100,00**	**100,00**	

14.6 HPLC chromatogram of the follow up transformation

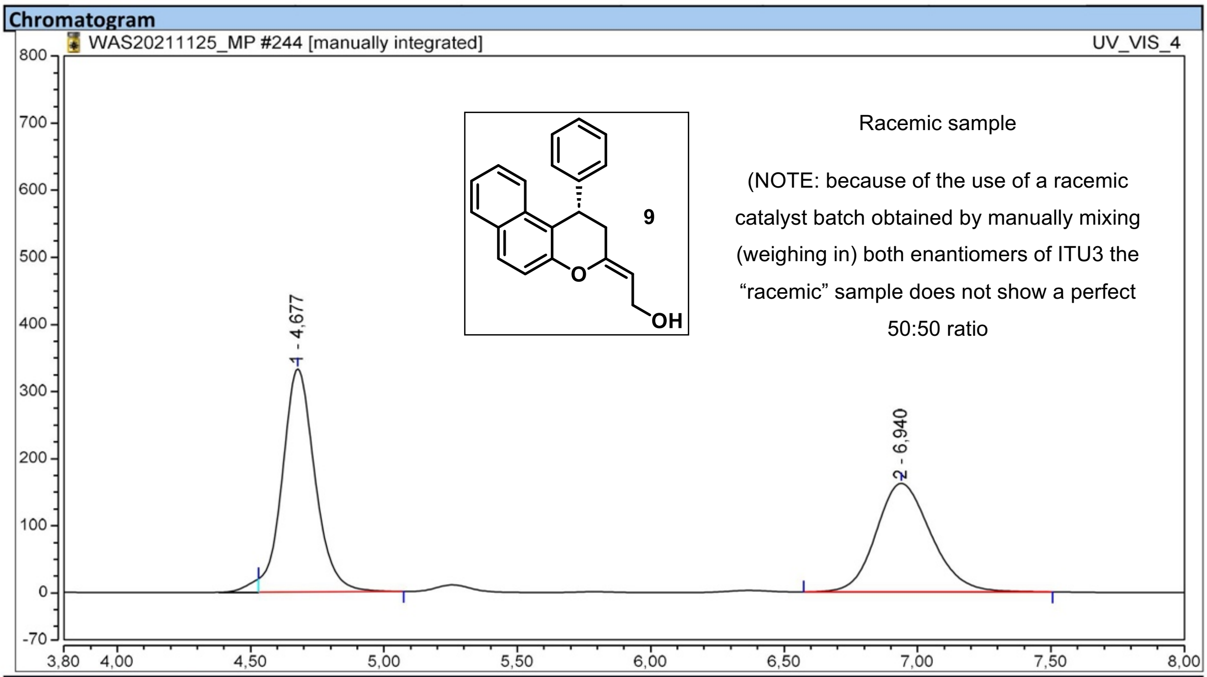

Integration Results

No.	Peak Name	Retention Time min	Area mAU*min	Height mAU	Relative Area %	Relative Height %	Amount n.a.
1		4,677	46,983	332,432	54,89	67,28	n.a.
2		6,940	38,608	161,669	45,11	32,72	n.a.
**Total:**			**85,591**	**494,101**	**100,00**	**100,00**	

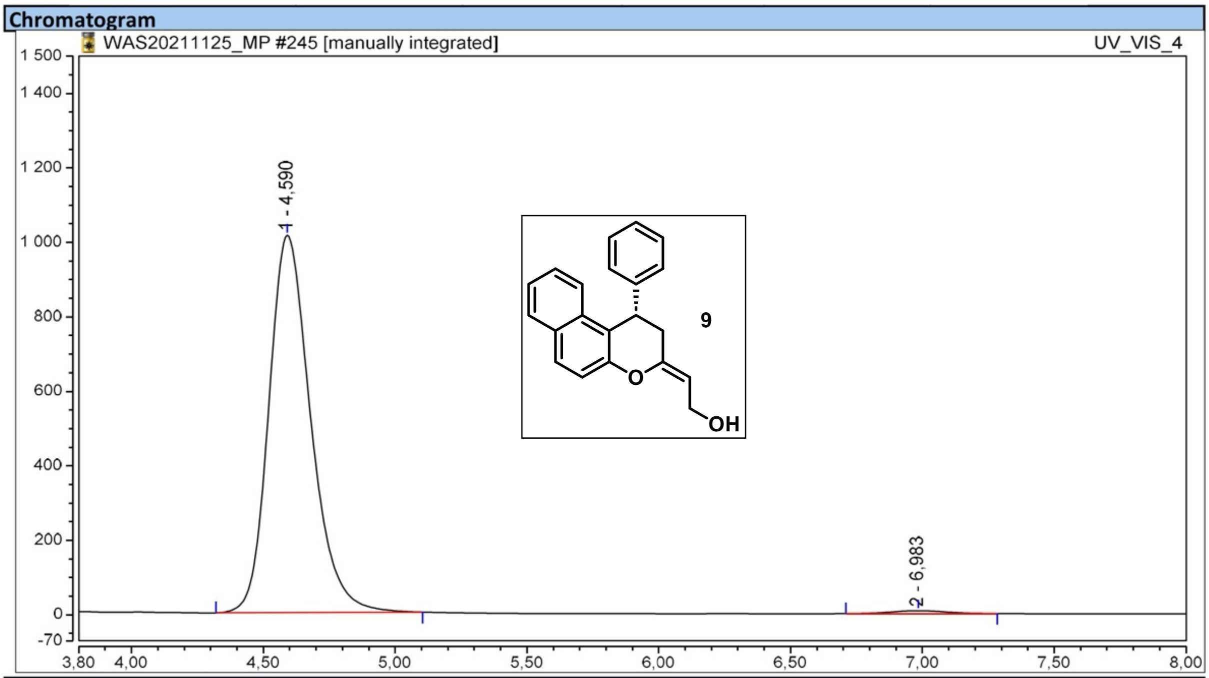

Integration Results

No.	Peak Name	Retention Time min	Area mAU*min	Height mAU	Relative Area %	Relative Height %	Amount n.a.
1		4,590	186,202	1013,432	98,98	99,23	n.a.
2		6,983	1,927	7,899	1,02	0,77	n.a.
**Total:**			**188,129**	**1021,332**	**100,00**	**100,00**	
